# Exploring the Pharmacological Mechanism of Radix Salvia Miltiorrhizae in the Treatment of Radiation Pneumonia by Using Network Pharmacology

**DOI:** 10.3389/fonc.2021.684315

**Published:** 2021-07-29

**Authors:** Peng Li, Xiaochun Xia, Jundong Zhou, Jinchang Wu

**Affiliations:** ^1^Department of Radiation Oncology, Huai’an Tumor Hospital & Huai’an Hospital of Huai’an City, Huai’an, China; ^2^Department of Radiation Oncology, Nantong Tumor Hospital, Affiliated Tumor Hospital of Nantong University, Nantong, China; ^3^Department of Radiation Oncology, Nanjing Medical University Affiliated Suzhou Hospital, Suzhou, China; ^4^Suzhou Cancer Center Core Laboratory, Nanjing Medical University Affiliated Suzhou Hospital, Suzhou, China; ^5^Department of Radiation Oncology, The Second Affiliated Hospital of Xuzhou Medical University, Xuzhou, China

**Keywords:** Radix Salviae Miltiorrhizae, radiation pneumonia, network pharmacology, thoracic neoplasms, traditional Chinese medicine

## Abstract

**Background:**

Radiation pneumonia (RP) is the most common complication of radiotherapy to the thorax and seriously affects the survival rate and quality of life of patients. Radix Salviae Miltiorrhizae (RSM) is an ancient Chinese medicine, whose main pharmacological effect is to promote blood circulation and remove stasis. A growing number of studies have proved that RSM has a good effect on RP. However, the underlying mechanism is still unclear and needs to be fully elucidated.

**Methods:**

The effective components and predictive targets of RSM were analyzed by Traditional Chinese Medicine Systems Pharmacology (TCMSP) database, and the related targets of RP were predicted by GeneCards database. The common targets of the two targets mentioned above were analyzed by protein-protein interaction on the STRING website, GO and KEGG analysis on the DAVID website, visualization by CytoScape3.7.0, and screening for Hubber gene by cytoHubber plug-in.

**Results:**

A search of the TCMSP database revealed that RSM contains 65 chemical constituents and 165 potential protein targets. A total of 2,162 protein targets were found to be associated with RP. The top 10 hub genes were obtained by MCC algorithm for 70 common genes, including TP53, CASP3, MAPK1, JUN, VEGFA, STAT3, PTGS2, IL6, AKT1, and FOS. By analyzing the Gene Ontology, The anti-radiation pneumonia effect of RSM is that it performs molecular functions (protein homodimerization activity) in the nucleus through three biological processes (positive regulation of transcription from RNA polymerase II promoter,Extrinsic apoptotic signaling pathway in absence of ligand and lipopolysaccharide-mediated signaling pathway). Through KEGG analysis, the mechanism of RSM treatment of radiation pneumonia may be through PI3K-Akt, HIF-1, TNF signaling pathways.

**Conclusions:**

Through network pharmacology analysis, we found the possible target genes of RSM on RP and revealed the most likely signaling pathway, providing theoretical basis for further elucidating the potential mechanism of RSM on RP.

## Introduction

Radiation therapy (RT) has become one of the main treatments for thorax malignancies (1). The lung is a radiation-sensitive organ, so radiation pneumonia (RP) is the most common complication of chest radiotherapy, which limits the dose of tumor target and affects the effect of RT (2). With the advancement of modern radiotherapy equipment and technology (including IGRT, proton, and heavy particle therapy), the survival rate of radiotherapy for thorax tumors continues to increase, and the survival time is prolonged, but the proportion of patients with RP cannot be eliminated and increased year by year. The incidence of asymptomatic radiation pneumonia diagnosed by imaging is as high as 43%, while the incidence of clinically diagnosed symptomatic radiation pneumonia is slightly lower at 5–15% (3). Treatment and prevention of radiation pneumonia are urgently needed. As the exact mechanism of radiation pneumonia is still unclear, high-dose steroid hormone therapy is the main treatment of choice, and serious side effects such as immunosuppression and osteoporosis caused by high-dose hormones have limited their application, and patients treated with corticosteroids alone often relapse. In recent years, many traditional Chinese medicines for Blood Activating Stasis Removing Drugs have obtained good curative effect and research progress in the prevention and treatment of RP (4).

Radix Salvia Miltiorrhizae (RSM, Danshen in Chinese) is one of the oldest traditional Chinese medicines in China, with a history of more than 1,000 years of clinical application (5, 6). RSM has been included in the Chinese Pharmacopoeia since 1963. It is a common blood rheological agent that promotes blood circulation, stops bleeding and stasis, and nourishes and calms the nerves. Modern pharmacological research shows that RSM has the ability to protect vascular endothelial cells, prevent arrhythmia, prevent atherosclerosis, improve microcirculation, protect the heart muscle, inhibit and release platelet aggregation, increase coronary flow, increase the body’s ability to resist hypoxia, and inhibit collagen fibers. It produces and promotes the degradation of fibrin, is anti-inflammatory, promotes anti-lipid peroxidation and scavenging free radicals, as well as protects liver cells and prevents pulmonary fibrosis (7–9). A growing number of studies have pointed out that RSM has a good effect on RP (10–15), but its underlying molecular mechanism is still unclear.

Network pharmacology came into being with the development of genomics, proteomics, and systems biology (16), by mining data on drugs and diseases in public databases, analyzing the active ingredients of drugs and related target genes of diseases, and studying the possible mechanism of action of drugs. Combining modern network pharmacology with traditional Chinese medicine is conducive to revealing the pharmacological effects of Chinese medicine from a complex network of traditional Chinese medicine with multiple components, multiple pathways, and multiple targets (17). In this study, the active ingredients of RSM were screened through the network pharmacology research method, and multiple targets and pathways for the treatment of radiation pneumonia were excavated to provide a reference for elucidating the mechanism of action of RSM.

## Materials and Methods

**Figure 1:**
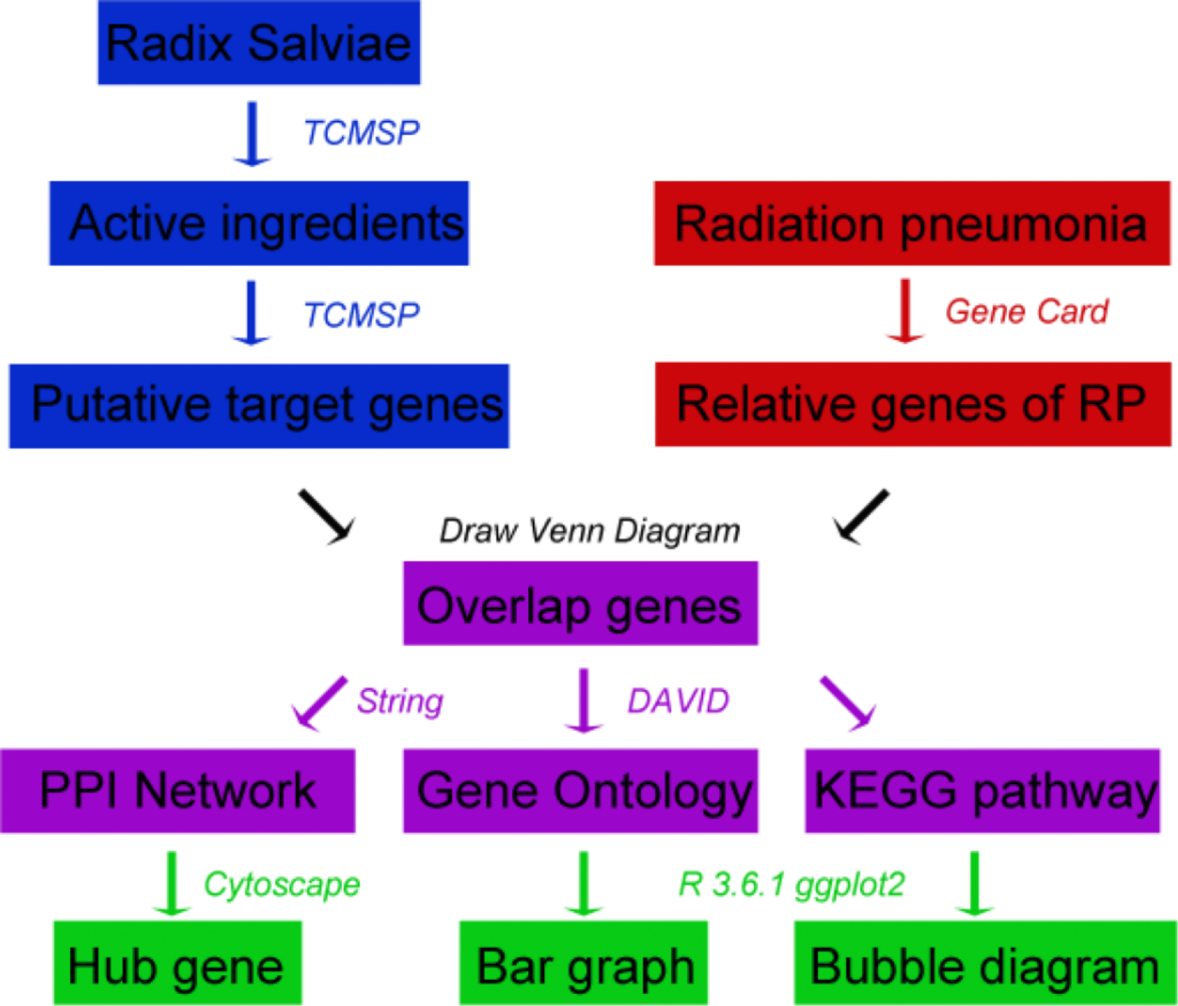
Flowchart of the main research process.

The main research process of this research is shown in the flowchart (**Figure 1**). First, we searched the active ingredients and putative target of RSM, and at the same time, we searched for the genes related to radiation pneumonia. The intersection of the above genes is used as the common genes for subsequent research. The protein-protein interaction network, hub gene, GO enrichment analysis, and KEGG analysis were analyzed separately.

### RSM Chemical Ingredients and Putative Target

We used the Traditional Chinese Medicine System Pharmacology Database (18) (TCMSP™, http://tcmspw.com/tcmsp.php) to retrieve the effective chemical ingredients of RSM and their putative targets. TCMSP is a systematic pharmacological platform for the study of herbal medicines. Its functions include the identification of the chemical constituents of herbal medicines and their corresponding targets. Absorption, distribution, metabolism, and excretion (ADME) are key processes that need to be considered in screening compounds for TCM. Oral bioavailability (OB) and drug similarity (DL) were the most important pharmacokinetic parameters. OB is the dose and rate at which the active ingredient of a drug enters the human bloodstream by mouth, and DL is used to evaluate the structural similarity between a drug and clinical treatment drugs in the drugbank database. The higher the OB and DL, the more likely it is to be an active ingredient in the drug. On the website, we searched for effective compounds by inputting Chinese pinyin “Danshen” and screened out effective compounds according to the criteria of oral bioavailability (OB) >30% and drug likeness (DL) >0.18, and we found their corresponding target genes.

### Genes Related to Radiation Pneumonia

We searched genes related to radiation pneumonia through the Genecards website (GeneCards, https://www.genecards.org/) (19), which is an integrative database that includes genomic, transcriptomic, proteomic, genetic, clinical, and functional information. The current version is 4.12 and includes 13,878 disease genes. Using “radiation pneumonia” as the keyword, we established the disease target database of RP.

### Intersection of Drug Target Genes and Disease Target Genes

We used Bioinformatics & Evolutionary Genomics website (http://bioinformatics.psb.ugent.be/webtools/Venn/) to intersect the targets that match the active ingredients of RSM and the targets related to radiation pneumonia. The obtained overlapping genes are the key genes of RSM to act on radiation pneumonia.

### Traditional Chinese Medicine, Active Ingredient, Target Genes, Disease Network

We inputted the abovementioned network data on traditional Chinese medicine, active ingredients of traditional Chinese medicine, target genes, and diseases into the software Cytoscape (http://cytoscape.org/, version 3.7.0) to obtain the topological network between them. Cytoscape is an open software platform for visualizing networks of biological pathways and molecular interaction networks, and it has rich plug-in functions.

### Protein-Protein Interaction Network and Hub Genes

We used the String website and Cytoscape 3.7.1 software to analyze the key genes and build the network. The String database contains a large number of known or predicted PPI relationships. Cytoscape is a graph-oriented software for the analysis and visualization of genomic networks widely used in network pharmacology research. We uploaded the intersection gene to the String website (http://stringdb.org/, 10th edition) (20), with the restriction that the species selects “*Homo sapiens*” and the confidence is >0.4. The obtained protein-protein interaction network data were inputted into the software Cytoscape 3.7.0, and the Hub gene was obtained using the plug-in cytoHubber.

### Gene Ontology and KEGG Enrichment Analysis

We performed Gene Ontology (GO) enrichment analysis and KEGG signal pathway enrichment analysis of intersection genes through the Database for Annotation, Visualization and Integrated Discovery (DAVID, https://david.ncifcrf.gov/home.jsp, ver. 6.8) (21, 22). GO enrichment mainly analyses the biological process, cellular composition, and molecular function of the target, whereas KEGG (www.kegg.jp/kegg/kegg1.html) enrichment analyzes the potential biological pathways and functions associated with the target. The data were visualized by software R language 3.6.1 and GGplot2 package.

## Results

### RSM Chemical Ingredients and Putative Targets

We have screened 65 active compounds through the website according to the foregoing criteria. The detailed information are shown in **Table 1**, and 165 corresponding targets are shown in **Table S1**. Seven of the 65 compounds obtained had no corresponding target genes, and the final 58 compounds produced 165 putative targets.

**Table 1:** The information on the active ingredients of *Salvia miltiorrhiza*.

MOL ID	Molecule Name	OB	DL
MOL001601	1,2,5,6-tetrahydrotanshinone	38.75	0.36
MOL001659	Poriferasterol	43.83	0.76
MOL001771	poriferast-5-en-3beta-ol	36.91	0.75
MOL001942	isoimperatorin	45.46	0.23
MOL002222	sugiol	36.11	0.28
MOL002651	Dehydrotanshinone II A	43.76	0.4
MOL002776	Baicalin	40.12	0.75
MOL000569	digallate	61.85	0.26
MOL000006	luteolin	36.16	0.25
MOL006824	α-amyrin	39.51	0.76
MOL007036	5,6-dihydroxy-7-isopropyl-1,1-dimethyl-2,3-dihydrophenanthren-4-one	33.77	0.29
MOL007041	2-isopropyl-8-methylphenanthrene-3,4-dione	40.86	0.23
MOL007045	3α-hydroxytanshinone II a	44.93	0.44
MOL007048	(E)-3-[2-(3,4-dihydroxyphenyl)-7-hydroxy-benzofuran-4-yl]acrylic acid	48.24	0.31
MOL007049	4-methylenemiltirone	34.35	0.23
MOL007050	2-(4-hydroxy-3-methoxyphenyl)-5-(3-hydroxypropyl)-7-methoxy-3-benzofurancarboxaldehyde	62.78	0.4
MOL007051	6-o-syringyl-8-o-acetyl shanzhiside methyl ester	46.69	0.71
MOL007058	formyltanshinone	73.44	0.42
MOL007059	3-beta-Hydroxymethyllenetanshiquinone	32.16	0.41
MOL007061	Methylenetanshinquinone	37.07	0.36
MOL007063	przewalskin a	37.11	0.65
MOL007064	przewalskin b	110.32	0.44
MOL007068	Przewaquinone B	62.24	0.41
MOL007069	przewaquinone c	55.74	0.4
MOL007070	(6S,7R)-6,7-dihydroxy-1,6-dimethyl-8,9-dihydro-7H-naphtho[8,7-g]benzofuran-10,11-dione	41.31	0.45
MOL007071	przewaquinone f	40.31	0.46
MOL007077	sclareol	43.67	0.21
MOL007079	tanshinaldehyde	52.47	0.45
MOL007081	Danshenol B	57.95	0.56
MOL007082	Danshenol A	56.97	0.52
MOL007085	Salvilenone	30.38	0.38
MOL007088	cryptotanshinone	52.34	0.4
MOL007093	dan-shexinkum d	38.88	0.55
MOL007094	danshenspiroketallactone	50.43	0.31
MOL007098	deoxyneocryptotanshinone	49.4	0.29
MOL007100	dihydrotanshinlactone	38.68	0.32
MOL007101	dihydrotanshinone I	45.04	0.36
MOL007105	epidanshenspiroketallactone	68.27	0.31
MOL007107	C09092	36.07	0.25
MOL007108	isocryptotanshi-none	54.98	0.39
MOL007111	Isotanshinone II	49.92	0.4
MOL007115	manool	45.04	0.2
MOL007118	microstegiol	39.61	0.28
MOL007119	miltionone I	49.68	0.32
MOL007120	miltionone II	71.03	0.44
MOL007121	miltipolone	36.56	0.37
MOL007122	Miltirone	38.76	0.25
MOL007123	miltirone II	44.95	0.24
MOL007124	neocryptotanshinone ii	39.46	0.23
MOL007125	neocryptotanshinone	52.49	0.32
MOL007127	1-methyl-8,9-dihydro-7H-naphtho[5,6-g]benzofuran-6,10,11-trione	34.72	0.37
MOL007130	prolithospermic acid	64.37	0.31
MOL007132	(2R)-3-(3,4-dihydroxyphenyl)-2-[(Z)-3-(3,4-dihydroxyphenyl)acryloyl]oxy-propionic acid	109.38	0.35
MOL007140	(Z)-3-[2-[(E)-2-(3,4-dihydroxyphenyl)vinyl]-3,4-dihydroxy-phenyl]acrylic acid	88.54	0.26
MOL007141	salvianolic acid g	45.56	0.61
MOL007142	salvianolic acid j	43.38	0.72
MOL007143	salvilenone I	32.43	0.23
MOL007145	salviolone	31.72	0.24
MOL007149	NSC 122421	34.49	0.28
MOL007150	(6S)-6-hydroxy-1-methyl-6-methylol-8,9-dihydro-7H-naphtho[8,7-g]benzofuran-10,11-quinone	75.39	0.46
MOL007151	Tanshindiol B	42.67	0.45
MOL007152	Przewaquinone E	42.85	0.45
MOL007154	tanshinone iia	49.89	0.4
MOL007155	(6S)-6-(hydroxymethyl)-1,6-dimethyl-8,9-dihydro-7H-naphtho[8,7-g]benzofuran-10,11-dione	65.26	0.45
MOL007156	tanshinone VI	45.64	0.3

OB, Oral Bioavailability; DL, Drug-likeness.

### Genes Related to Radiation Pneumonia

We used the key word “radiation pneumonia” and screened more than 2,000 genes related to radiation pneumonia through the website (see **Table S2** for details).

### Traditional Chinese Medicine, Active Ingredient, Target Genes, Disease Network

**Figure 2:**
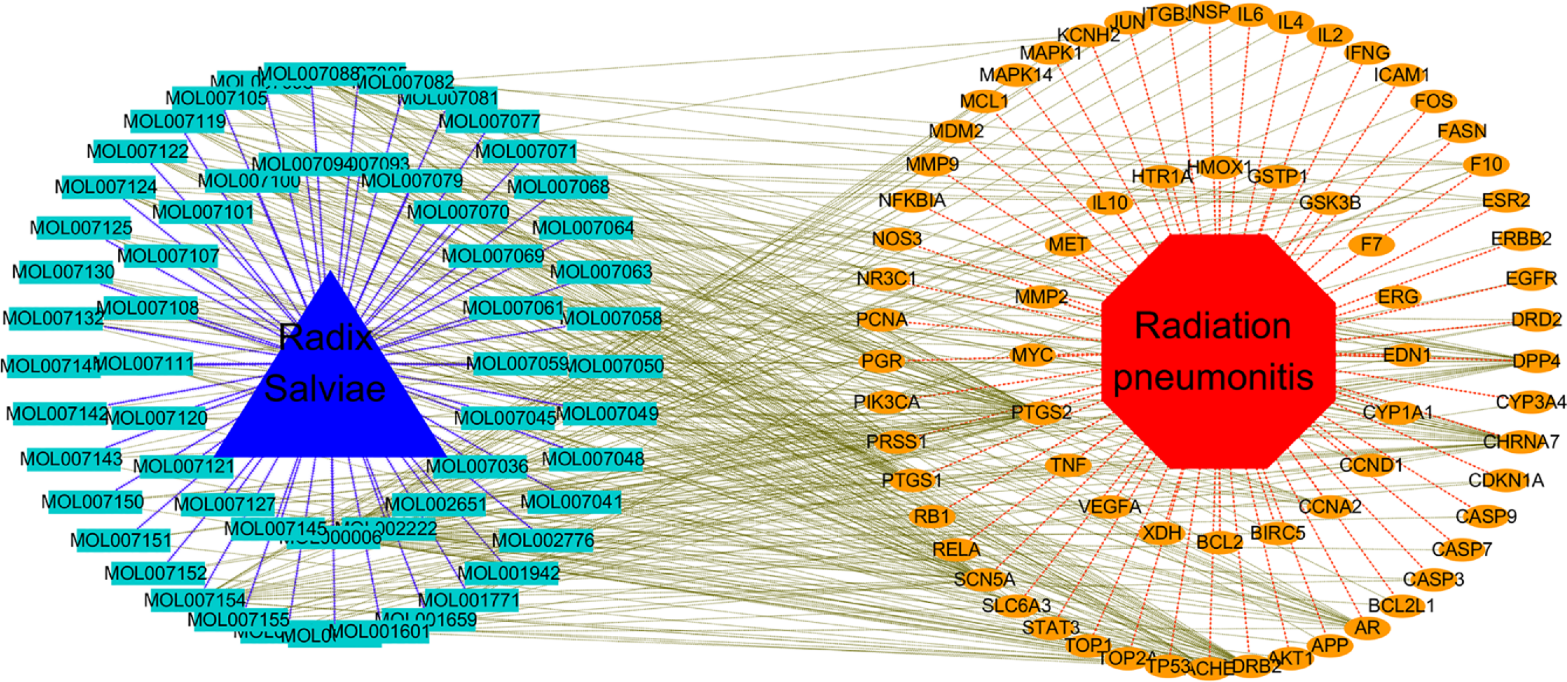
Topological network of Drug, Chemical components, Putative target, Disease. (The red octagon represents the disease, the orange ovals represent the putative targets, the blue triangle represents the drug, and the pale blue rectangles represent the chemical components).

Through the software cytoscape3.7.0, a topological network between drug, chemical components, targets, and disease is constructed, as shown in **Figure 2**. The red octagon represents radiation pneumonia, the orange ovals represent the putative targets, the blue triangle represents RSM, and the pale blue rectangles represent the active ingredients. RSM has 58 active ingredients (seven active ingredients are not related to radiation pneumonia) and 70 targets related to radiation pneumonia. There are 358 links between 58 active ingredients and 70 target genes, as shown in **Table S3**.

### Intersection of Drug Target Genes and Disease Target Genes

**Figure 3:**
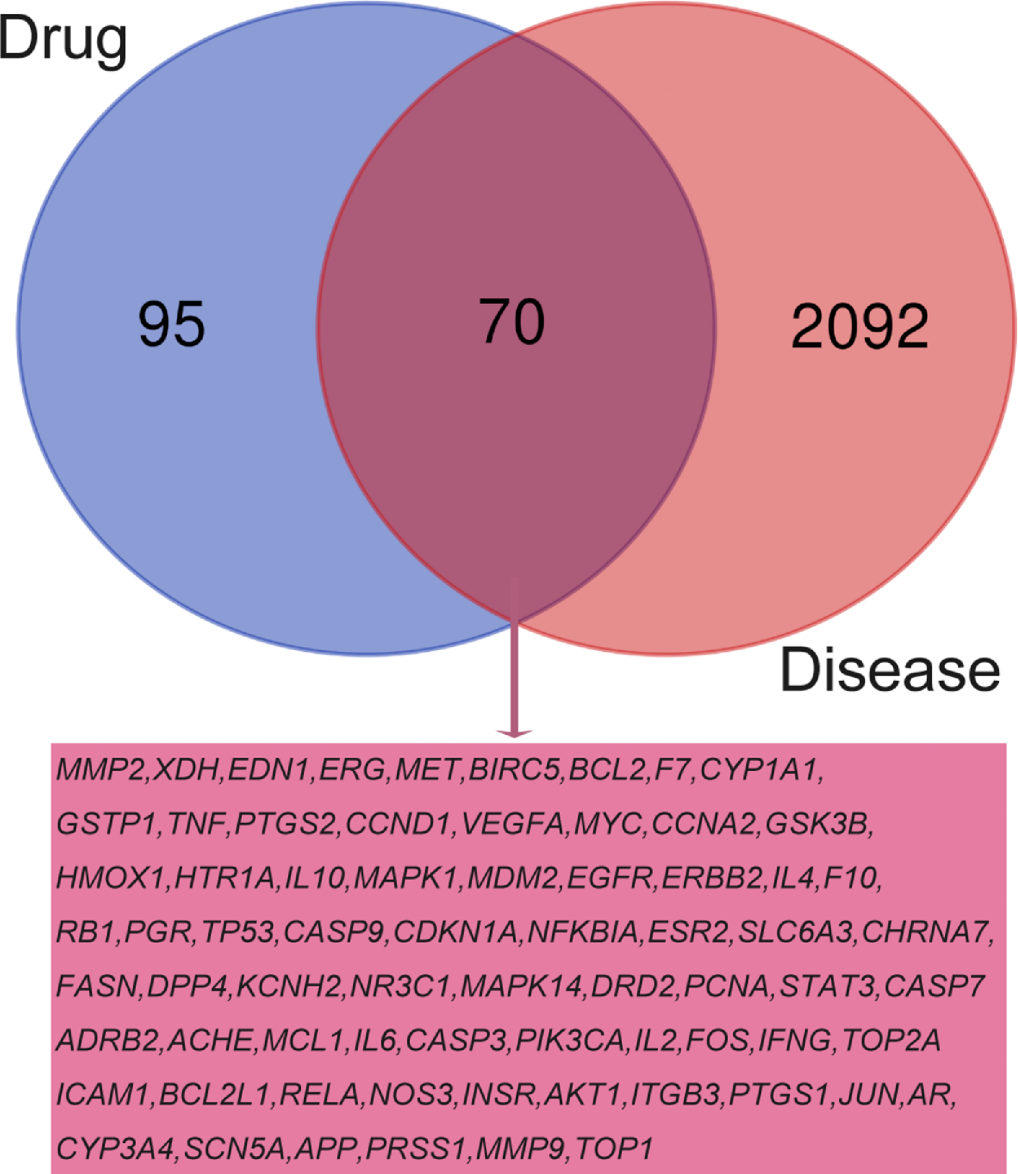
Venn diagram for drug prediction of target genes and disease-related genes.

In order to clarify the pharmacological role of RSM in RP, as shown in **Figure 3** of Venn, we matched the 165 genes predicted by RSM and the 2,162 genes predicted by RP to obtain 70 overlapping genes, which are probably the most critical genes for RSM to act on RP.

### Protein-Protein Interaction Network and Hub Genes

**Figure 4:**
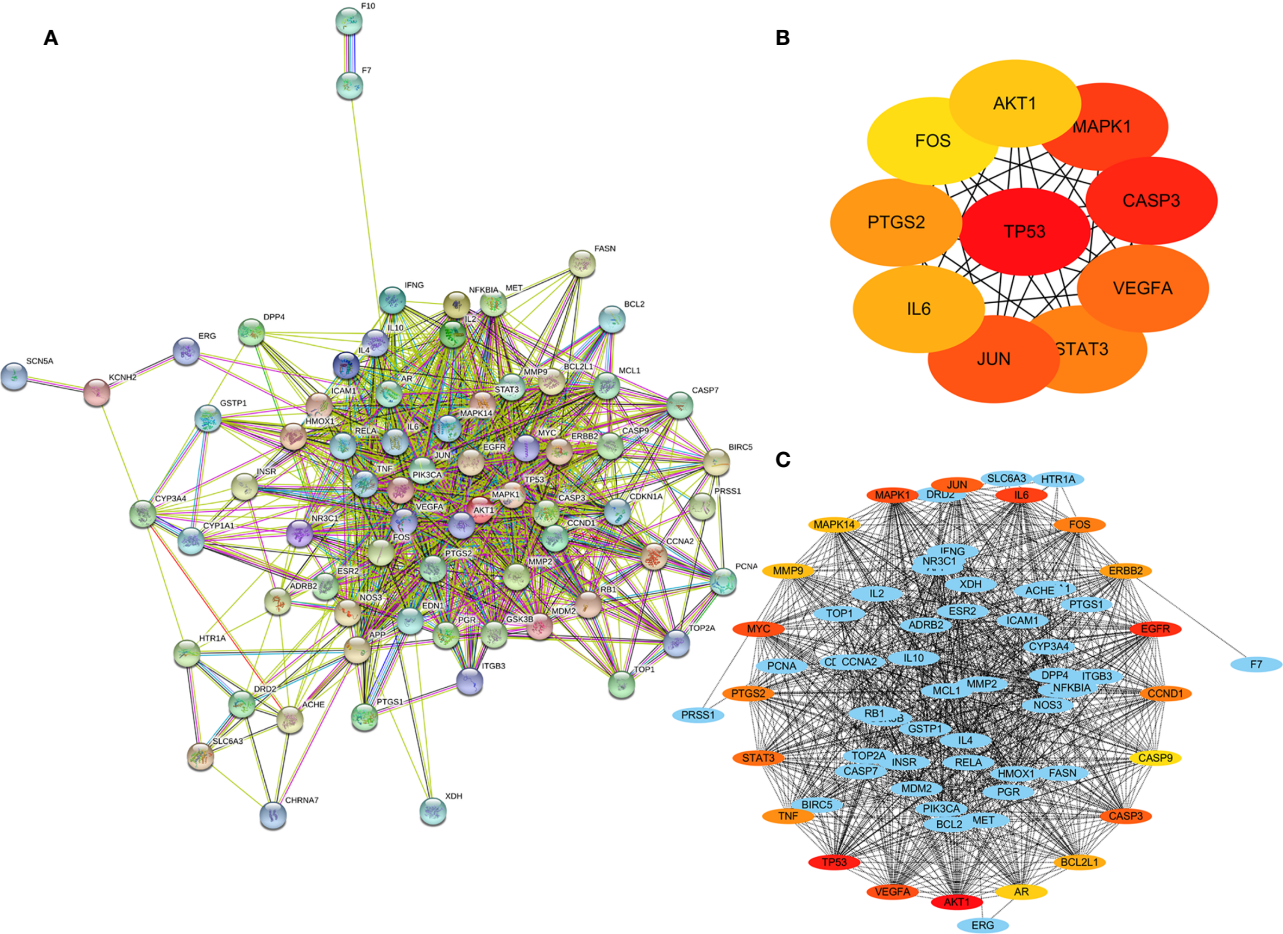
Protein-protein interaction (PPI) network of 70 common genes. **(A)** PPI network of common genes, **(B)** Top 10 hub genes of 70 common genes by MCC algorithm. **(C)** Top 20 hub genes of 70 common genes with expanded subnetwork.

The string website (STRING, https://string-db.org/) was used to build a network of 70 common genes, with 70 nodes and 903 edges (**Figure 4A**). By importing the above data into cytoscape3.7.0,and using the CytoHubber plug-in, the MCC algorithm yielded the top 10 hub genes (**Figure 4B**): *TP53, CASP3, MAPK1, JUN, VEGFA, STAT3, PTGS2, IL6, AKT1*, and *FOS*. The top 20 key genes obtained through the Degree algorithm are (**Figure 4C**) *AKT1, TP53, EGFR, IL6, VEGFA, MYC, MAPK1, CASP3, JUN, STAT3, PTGS2, FOS, CCND1, TNF, ERBB2, BCL2L1, MMP9, MAPK14, AR*, and *CASP9.* The above analysis suggests that these genes may be the key genes for RSM to act in RP.

### Gene Ontology and KEGG Enrichment Analysis

**Figure 5:**
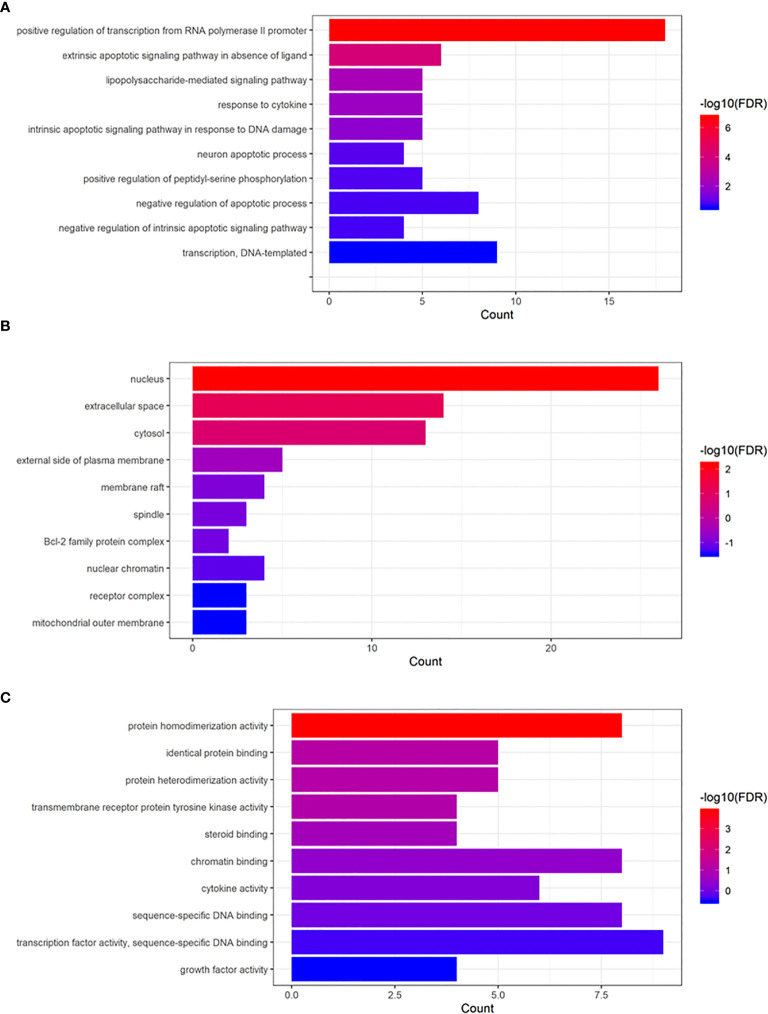
Gene Ontology enrichment analysis of common genes. **(A)** Biological process, **(B)** cellular component, **(C)** molecular function.

We uploaded 70 common genes of RSM and RP to DAVID’s website for GO analysis and KEGG analysis. Enrichment of Gene Oncology (molecular function [MF], biological process [BP], cellular components [CC]) is displayed in **Figure 5**. The p-values of these GO terms were less than 0.05, including protein homodimerization activity (MF), positive regulation of transcription from RNA polymerase II promoter (BP), extrinsic apoptotic signaling pathway in the absence of ligand (BP) and lipopolysaccharide-mediated signaling pathway (BP), and nucleus (CC).

**Figure 6:**
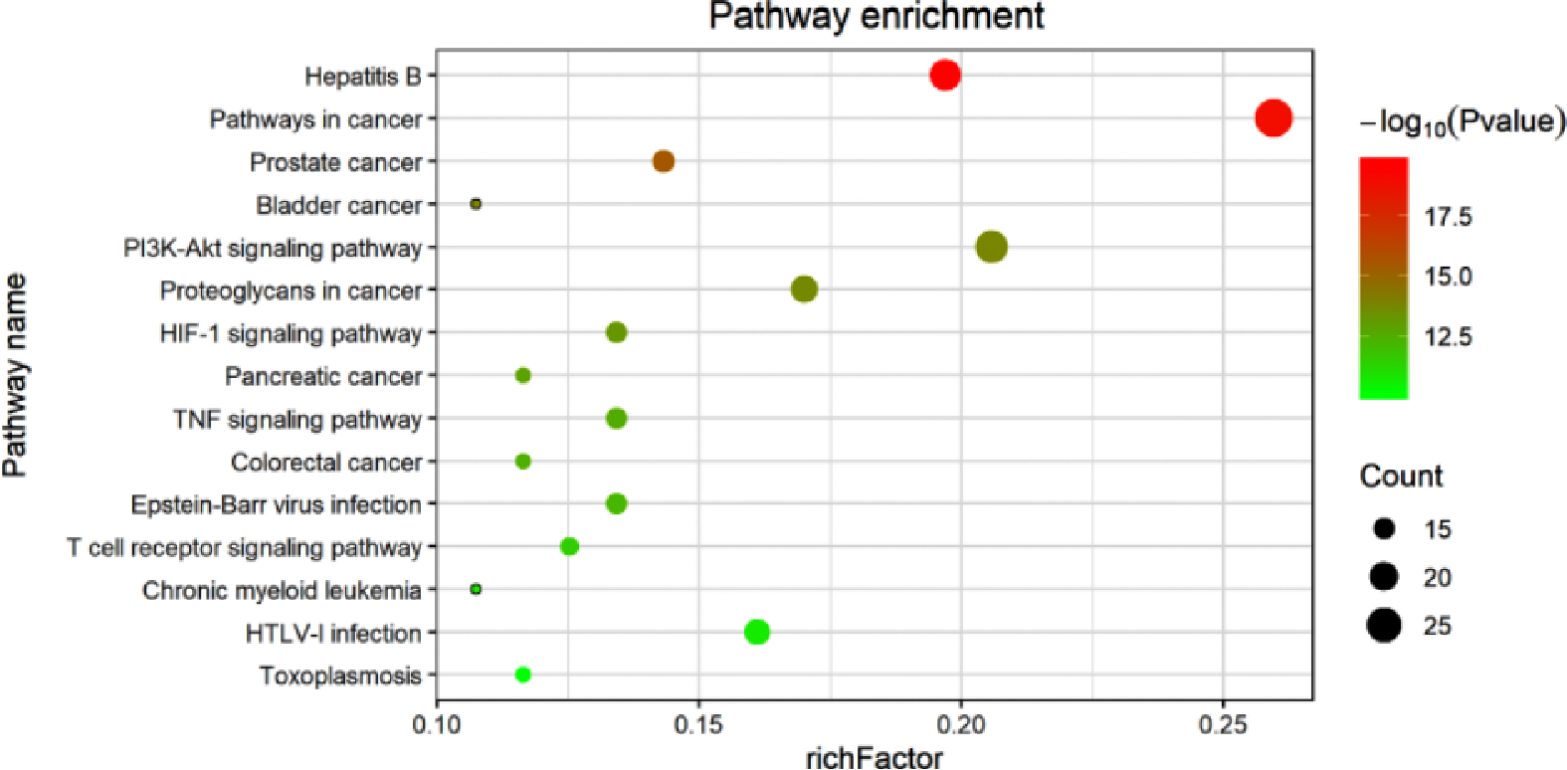
KEGG pathway analysis for common genes.

**Figure 7:**
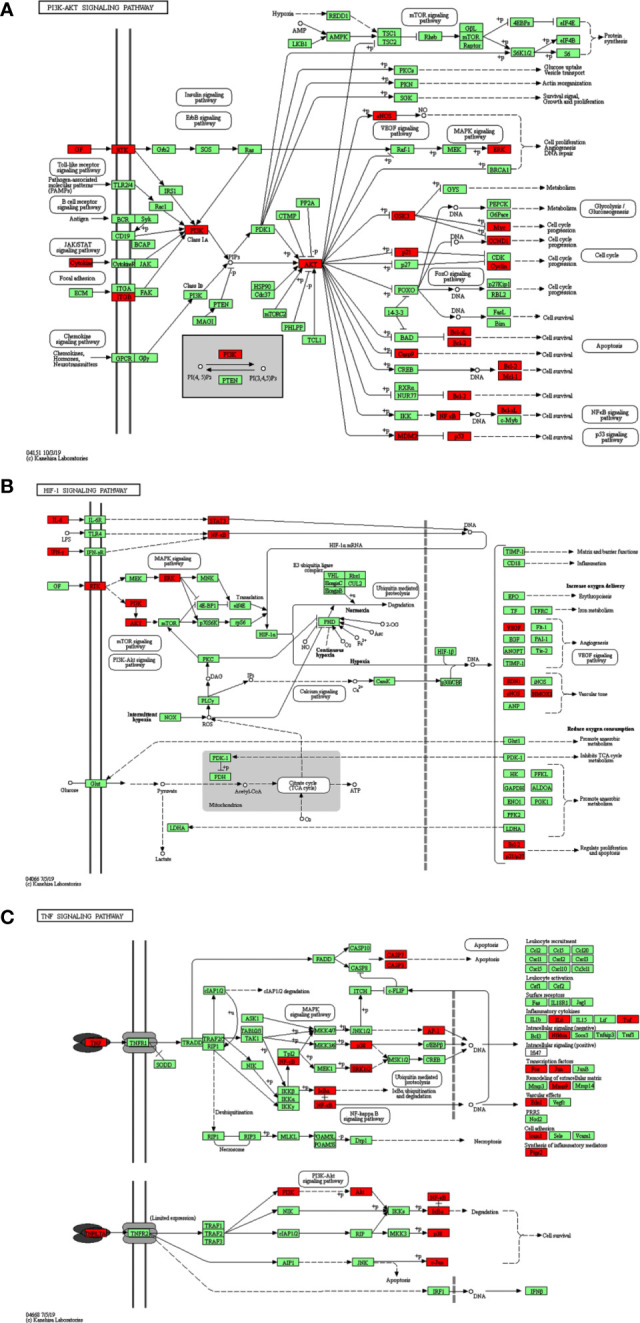
KEGG signaling pathway. **(A)** PI3K-AKT signaling pathway, **(B)** HIF-1 signaling pathway, **(C)** TNF signaling pathway.

Additionally, the results of the KEGG Pathway analysis showed that 70 genes were mapped in 50 signal pathways with significant differences (P<0.05), and the top 15 pathways are shown in **Figure 6**. Among them, 23, 15, and 15 genes were enriched in PI3K/AKT (*EGFR, IL4, IL6, MCL1, RELA, MET, TP53, ITGB3, BCL2L1, AKT1, MAPK1, CDKN1A, CCND1, CASP9, GSK3B, BCL2, VEGFA, PIK3CA, MDM2, NOS3, INSR, MYC, IL2*), HIF-1 (*EGFR, IL6, RELA, ERBB2, EDN1, STAT3, AKT1, MAPK1, CDKN1A, BCL2, VEGFA, IFNG, PIK3CA, NOS3, INSR*), and TNF (*ICAM1, IL6, TNF, PTGS2, RELA, MMP9, EDN1, NFKBIA, AKT1, FOS, MAPK1, CASP3, MAPK14, JUN, PIK3CA*) pathway, respectively. The diagram of the above signaling pathways is shown in **Figure 7**. These pathways are closely related to cell proliferation, differentiation, apoptosis, and adhesion, so we believe that RSM may improve the occurrence and development of radiation pneumonia through these pathways.

## Discussion

RP is one of the most challenging clinical complications of radiotherapy for lung malignancies. At present, the molecular mechanism of its pathogenesis mainly has the following points. First, the free radicals produced by radiotherapy can be treated with amphostine. Second, recruit inflammatory cells, which can be treated with the drug celecoxib. Third, cytokines and growth factors, therapeutic drugs TGF-β inhibitors (SM16) are still in basic research. Fourth, angiotensin-converting enzyme inhibitors (ACEI) have a potential role in reducing radiation-induced lung injury. However, the therapeutic effect is not good. The traditional Chinese medicine RSM has been widely used in the treatment of RP in China and many Chinese literature reports have been published, but the mechanism has not been clarified yet.

In our study, we obtained 65 effective compounds of RSM from the database and predicted 165 possible target genes, while we screened out 2,162 genes associated with radioactive pneumonia. By intersections of these genes, we obtained 70 common genes, which may regulate the pharmacological effects of these genes on radiation pneumonia. By establishing the network between drugs, effective compounds, target genes, and diseases (**Figure 2**), we were surprised to find that 50 active components of RP act on the target gene PTGS2, while the active component MOL000006 luteolin acts on 43 target genes simultaneously.

The full name of PTGS2 is prostaglandin peroxidase 2, also known as cyclooxygenase-2 (cox-2), which is a key enzyme in prostaglandin biosynthesis and has the dual function of dioxygenase and peroxidase. The expression of PTGS2 is positively correlated with the production of ROS and inflammatory signals in tissues, and inhibiting cox-2 can reduce inflammatory symptoms (23). The most studied cox-2 inhibitor, Celecoxib, has been shown to reduce the toxicity of radiation to the lungs (24). The possible mechanism by which this leads to radiation protection is activation of PTEN and inactivation of AKT (25). Luteolin is an active flavonoid compound with anti-oxidative, anti-inflammatory, and anti-fibrotic properties, which also alleviates collagen deposition, TGF-β1 expression, and lung fibrosis (26). Luteolin acts on hepatic stellate cells to anti-fibrosis *via* AKT/mTOR/p70S6K and TGFβ/Smad signaling pathways (27).

PTGS2 and Luteolin are only the tip of the iceberg in the mechanism of resistance of RSM against RP, and its efficacy must be the result of multi-target and multi-pathway action. In order to further reveal the mechanism, we uploaded the obtained 70 common genes to the STRING website to obtain their PPI network, and we obtained the hub genes through two algorithms (MMC and Degree), which were displayed in multiple ways (**Figure 4**).

Activation of the STAT3 pathway may play an important role in the pathogenesis of radiation lung injury. The protective effect of delayed treatment of WP1066 suggests that the STAT3 signal may be a therapeutic target for RP (28). Clarithromycin can prevent radiation pneumonia by PTGS2, TNF-antigen, TNF receptor 1, NF-B, vcam-1, and MMP9 (29). Interleukin 6 (IL6) has been reported as a risk factor for RP and contributes to the development of RP (30). FOS plays an important role in the radiation resistance mechanism of malignant glioma and may be a potential new target for the treatment of malignant glioma (31). Many of the above genes play an important role in radiation resistance, some have been reported in RP, and some have not been published yet, which suggests that we should carry out relevant research and could find potential key genes related to RP in them.

In addition, to further understand the interaction between these common genes, we enriched their GO function and analyzed their KEGG pathway. Through our research, we found that RSM may regulate protein homodimerization activity *via* positive regulation of transcription from RNA polymerase II promoter, extrinsic apoptotic signaling pathway in the absence of ligand- and lipopolysaccharide-mediated signaling pathway, and response to cytokine in the nucleus to anti-radiation pneumonia. Many proteins need to function as homologous dimers; affecting the activity of protein homologous dimer will lead to the loss of its function (32). The repair mechanism of DNA double-strand break includes homologous recombination and non-homologous end joining (33). The change of protein homodimerization activity must lead to the resistance or sensitivity of cells to radiation. Although not experimentally confirmed, there is reason to believe that RSM may improve the body’s ability to repair radiation damage through homologous recombination.

Through KEGG analysis, multiple signaling pathways may be the underlying mechanism. **Figure 7** shows the three pathways and highlights some of the 70 genes in common. PI3K-AKT, HIF, and TNF signaling pathways have all been reported in radiation pneumonia. Tang Y et al. reported that severe radiation pneumonia was associated with genetic variation in the PI3K/AKT pathway in patients with lung cancer after radiotherapy (34). Study of Toullec, A et al. has shown that loss of HIF in intestinal endothelial cells can reduce the severity of radiation enteritis, but similar loss of intestinal epithelial cells cannot (35). Loss of HIF-1α does not have a beneficial effect on lung injury in mice during stereotactic radiotherapy (36). Zhang, M et al. demonstrated the selective protective effect of TNF-α pathway inhibition on radiation lung injury by gene knockout and antisense oligonucleotide (ASO) silencing of TNF-α in mice model of lung metastasis of colon cancer (37). Multiple target genes of RSM are enriched in the three pathways mentioned above, and the roles of these pathways are mainly focused on cell survival, cell cycle progression, cell proliferation, angiogenesis, DNA repair, reducing oxygen consumption, regulating proliferation and apoptosis, and synthesis of inflammatory mediators. The complexity of its functions exactly reflects the multi-component, multi-target, and multi-pathway pharmacological mechanism of RSM. The above analysis points out the direction of our further research.

Our study had several limitations. First of all, more Chinese medicine target gene databases and more comprehensive disease prediction databases are needed to make the combined analysis results more reliable. The second is that generic databases and analytics alone are not enough to clarify the actual mechanism. Our results need to be further validated in cell and animal models. The comprehensive understanding of RSM and RP depends on the common development of multi-disciplines.

## Conclusion

By means of network pharmacology, our study predicted the effective components of RSM and explored the underlying mechanism of the potential anti-RP effect most likely to focus on luteolin and be used as a PTSG2 target. Through the analysis of specific signaling pathways, we believe that RSM may pass through the main PI3K-AKT, HIF-1, and TNF signaling pathways. The mechanism of resistance to radiation pneumonia is the direct or indirect synergistic effect of multiple targets and multiple pathways, rather than the result of single target and single pathway. This study revealed the potential mechanism of RSM resistance to radiation pneumonia in theory, but further experimental verification is needed to clarify its true internal mechanism.

## Data Availability Statement

The original contributions presented in the study are included in the article/**Supplementary Material**. Further inquiries can be directed to the corresponding authors.

## Author Contributions

PL completed most of the research and drafted manuscripts. XX plotted all of the charts. JZ analyzed the data. JW designed the research and reviewed the manuscript. All authors contributed to the article and approved the submitted version.

## Funding

This work was supported by the National Natural Science Foundation of China (81672975), the Six Talent Peaks Project of Jiangsu Province of China (WSN095).

## Conflict of Interest

The authors declare that the research was conducted in the absence of any commercial or financial relationships that could be construed as a potential conflict of interest.

## Publisher’s Note

All claims expressed in this article are solely those of the authors and do not necessarily represent those of their affiliated organizations, or those of the publisher, the editors and the reviewers. Any product that may be evaluated in this article, or claim that may be made by its manufacturer, is not guaranteed or endorsed by the publisher.
