# Supplementary material for: Exploring the Pharmacological Mechanism of Radix Salvia Miltiorrhizae in the Treatment of Radiation Pneumonia by Using Network Pharmacology
Source: Front Oncol. 2021 Jul 29;11:684315. doi: 10.3389/fonc.2021.684315 (PMC8358777; doi:10.3389/fonc.2021.684315)
Supplement: Supplementary file 1 [file Table_1.docx]

Table S1 The information of putative target genes of RSM

| MOL ID | Molecule Name | Target Name |
| --- | --- | --- |
| MOL000006 | luteolin | MMP2 |
| MOL000006 | luteolin | ADCY2 |
| MOL000006 | luteolin | APP |
| MOL000006 | luteolin | AR |
| MOL000006 | luteolin | BIRC4 |
| MOL000006 | luteolin | BIRC5 |
| MOL000006 | luteolin | BCL2L1 |
| MOL000006 | luteolin | CASP3 |
| MOL000006 | luteolin | CASP7 |
| MOL000006 | luteolin | CASP9 |
| MOL000006 | luteolin | CD40L |
| MOL000006 | luteolin | CDPK2 |
| MOL000006 | luteolin | TP53 |
| MOL000006 | luteolin | CDKN1A CAP20 CDKN1 CIP1 MDA6 PIC1 SDI1 WAF1 |
| MOL000006 | luteolin | DPP4 |
| MOL000006 | luteolin | TOP1 |
| MOL000006 | luteolin | TOP2A |
| MOL000006 | luteolin | MDM2 |
| MOL000006 | luteolin | EGFR |
| MOL000006 | luteolin | CCND1 |
| MOL000006 | luteolin | CCNB1 CCNB |
| MOL000006 | luteolin | GSTP1 |
| MOL000006 | luteolin | HSP90 |
| MOL000006 | luteolin | HMOX1 |
| MOL000006 | luteolin | MET |
| MOL000006 | luteolin | MCL1 |
| MOL000006 | luteolin | INSR |
| MOL000006 | luteolin | ICAM1 |
| MOL000006 | luteolin | IFNG |
| MOL000006 | luteolin | IL10 |
| MOL000006 | luteolin | IL2 |
| MOL000006 | luteolin | IL4 |
| MOL000006 | luteolin | IL6 |
| MOL000006 | luteolin | MMP1 CLG |
| MOL000006 | luteolin | NUF2 |
| MOL000006 | luteolin | MMP9 |
| MOL000006 | luteolin | MAPK1 ERK2 PRKM1 PRKM2 |
| MOL000006 | luteolin | NFKBIA |
| MOL000006 | luteolin | NCOA2 |
| MOL000006 | luteolin | ppar gamma2 |
| MOL000006 | luteolin | PIK3CA |
| MOL000006 | luteolin | PCNA |
| MOL000006 | luteolin | PTGES MGST1L1 MPGES1 PGES PIG12 |
| MOL000006 | luteolin | PTGS1 COX1 |
| MOL000006 | luteolin | PTGS2 COX2 |
| MOL000006 | luteolin | AKT1 PKB RAC |
| MOL000006 | luteolin | ERBB2 |
| MOL000006 | luteolin | RB1 |
| MOL000006 | luteolin | SLC2A4 hCG_42005 |
| MOL000006 | luteolin | JUN |
| MOL000006 | luteolin | RELA |
| MOL000006 | luteolin | PRSS1 |
| MOL000006 | luteolin | TNF |
| MOL000006 | luteolin | TYR |
| MOL000006 | luteolin | VEGFA |
| MOL000006 | luteolin | XDH XDHA |
| MOL000569 | digallate | AKR1B1 |
| MOL000569 | digallate | HSP90 |
| MOL000569 | digallate | PTGS2 COX2 |
| MOL001601 | 1,2,5,6-tetrahydrotanshinone | HTR2A |
| MOL001601 | 1,2,5,6-tetrahydrotanshinone | HTR3A |
| MOL001601 | 1,2,5,6-tetrahydrotanshinone | ADRA1A |
| MOL001601 | 1,2,5,6-tetrahydrotanshinone | ADRA1B |
| MOL001601 | 1,2,5,6-tetrahydrotanshinone | ADRA1D |
| MOL001601 | 1,2,5,6-tetrahydrotanshinone | ADRB2 |
| MOL001601 | 1,2,5,6-tetrahydrotanshinone | CA2 |
| MOL001601 | 1,2,5,6-tetrahydrotanshinone | PDE3A |
| MOL001601 | 1,2,5,6-tetrahydrotanshinone | OPRD1 OPRD |
| MOL001601 | 1,2,5,6-tetrahydrotanshinone | TOP2 |
| MOL001601 | 1,2,5,6-tetrahydrotanshinone | DRD1 |
| MOL001601 | 1,2,5,6-tetrahydrotanshinone | GABRA1 |
| MOL001601 | 1,2,5,6-tetrahydrotanshinone | HSP90 |
| MOL001601 | 1,2,5,6-tetrahydrotanshinone | CHRM1 |
| MOL001601 | 1,2,5,6-tetrahydrotanshinone | CHRM2 |
| MOL001601 | 1,2,5,6-tetrahydrotanshinone | CHRM3 |
| MOL001601 | 1,2,5,6-tetrahydrotanshinone | CHRM4 |
| MOL001601 | 1,2,5,6-tetrahydrotanshinone | CHRM5 |
| MOL001601 | 1,2,5,6-tetrahydrotanshinone | OPRM1 |
| MOL001601 | 1,2,5,6-tetrahydrotanshinone | CHRNA7 |
| MOL001601 | 1,2,5,6-tetrahydrotanshinone | NCOA1 |
| MOL001601 | 1,2,5,6-tetrahydrotanshinone | NCOA2 |
| MOL001601 | 1,2,5,6-tetrahydrotanshinone | PTGS1 COX1 |
| MOL001601 | 1,2,5,6-tetrahydrotanshinone | PTGS2 COX2 |
| MOL001601 | 1,2,5,6-tetrahydrotanshinone | RXRA |
| MOL001601 | 1,2,5,6-tetrahydrotanshinone | SCN5A |
| MOL001601 | 1,2,5,6-tetrahydrotanshinone | SLC6A3 DAT1 |
| MOL001601 | 1,2,5,6-tetrahydrotanshinone | SLC6A4 |
| MOL001659 | Poriferasterol | NR3C2 |
| MOL001659 | Poriferasterol | PGR |
| MOL001771 | poriferast-5-en-3beta-ol | NCOA2 |
| MOL001771 | poriferast-5-en-3beta-ol | PGR |
| MOL001942 | isoimperatorin | PTGS2 COX2 |
| MOL002222 | sugiol | ACHE |
| MOL002222 | sugiol | ADRA1A |
| MOL002222 | sugiol | ADRA1B |
| MOL002222 | sugiol | ADRA1D |
| MOL002222 | sugiol | ADRB2 |
| MOL002222 | sugiol | CA2 |
| MOL002222 | sugiol | DRD2 |
| MOL002222 | sugiol | OPRD1 OPRD |
| MOL002222 | sugiol | CHRM1 |
| MOL002222 | sugiol | CHRM2 |
| MOL002222 | sugiol | CHRM3 |
| MOL002222 | sugiol | CHRM4 |
| MOL002222 | sugiol | CHRM5 |
| MOL002222 | sugiol | OPRM1 |
| MOL002222 | sugiol | CHRNA7 |
| MOL002222 | sugiol | PTGS2 COX2 |
| MOL002222 | sugiol | SCN5A |
| MOL002651 | Dehydrotanshinone II A | HTR2A |
| MOL002651 | Dehydrotanshinone II A | ACHE |
| MOL002651 | Dehydrotanshinone II A | ADRA1A |
| MOL002651 | Dehydrotanshinone II A | AR |
| MOL002651 | Dehydrotanshinone II A | ADRB2 |
| MOL002651 | Dehydrotanshinone II A | OPRD1 OPRD |
| MOL002651 | Dehydrotanshinone II A | DPP4 |
| MOL002651 | Dehydrotanshinone II A | DRD1 |
| MOL002651 | Dehydrotanshinone II A | ESR1 |
| MOL002651 | Dehydrotanshinone II A | GABRA1 |
| MOL002651 | Dehydrotanshinone II A | CHRM1 |
| MOL002651 | Dehydrotanshinone II A | CHRM3 |
| MOL002651 | Dehydrotanshinone II A | CHRM4 |
| MOL002651 | Dehydrotanshinone II A | CHRM5 |
| MOL002651 | Dehydrotanshinone II A | OPRM1 |
| MOL002651 | Dehydrotanshinone II A | CHRNA7 |
| MOL002651 | Dehydrotanshinone II A | NCOA1 |
| MOL002651 | Dehydrotanshinone II A | ppar gamma2 |
| MOL002651 | Dehydrotanshinone II A | PTGS2 COX2 |
| MOL002651 | Dehydrotanshinone II A | SCN5A |
| MOL002776 | Baicalin | F10 |
| MOL002776 | Baicalin | PTP |
| MOL007036 | 5,6-dihydroxy-7-isopropyl-1,1-dimethyl-2,3-dihydrophenanthren-4-one | ACHE |
| MOL007036 | 5,6-dihydroxy-7-isopropyl-1,1-dimethyl-2,3-dihydrophenanthren-4-one | ADRA1A |
| MOL007036 | 5,6-dihydroxy-7-isopropyl-1,1-dimethyl-2,3-dihydrophenanthren-4-one | ADRA1B |
| MOL007036 | 5,6-dihydroxy-7-isopropyl-1,1-dimethyl-2,3-dihydrophenanthren-4-one | ADRB2 |
| MOL007036 | 5,6-dihydroxy-7-isopropyl-1,1-dimethyl-2,3-dihydrophenanthren-4-one | CALM |
| MOL007036 | 5,6-dihydroxy-7-isopropyl-1,1-dimethyl-2,3-dihydrophenanthren-4-one | CA2 |
| MOL007036 | 5,6-dihydroxy-7-isopropyl-1,1-dimethyl-2,3-dihydrophenanthren-4-one | TOP2 |
| MOL007036 | 5,6-dihydroxy-7-isopropyl-1,1-dimethyl-2,3-dihydrophenanthren-4-one | CHRM1 |
| MOL007036 | 5,6-dihydroxy-7-isopropyl-1,1-dimethyl-2,3-dihydrophenanthren-4-one | CHRM3 |
| MOL007036 | 5,6-dihydroxy-7-isopropyl-1,1-dimethyl-2,3-dihydrophenanthren-4-one | OPRM1 |
| MOL007036 | 5,6-dihydroxy-7-isopropyl-1,1-dimethyl-2,3-dihydrophenanthren-4-one | NCOA1 |
| MOL007036 | 5,6-dihydroxy-7-isopropyl-1,1-dimethyl-2,3-dihydrophenanthren-4-one | NCOA2 |
| MOL007036 | 5,6-dihydroxy-7-isopropyl-1,1-dimethyl-2,3-dihydrophenanthren-4-one | PTGS1 COX1 |
| MOL007036 | 5,6-dihydroxy-7-isopropyl-1,1-dimethyl-2,3-dihydrophenanthren-4-one | PTGS2 COX2 |
| MOL007036 | 5,6-dihydroxy-7-isopropyl-1,1-dimethyl-2,3-dihydrophenanthren-4-one | RXRA |
| MOL007036 | 5,6-dihydroxy-7-isopropyl-1,1-dimethyl-2,3-dihydrophenanthren-4-one | SCN5A |
| MOL007041 | 2-isopropyl-8-methylphenanthrene-3,4-dione | HTR2A |
| MOL007041 | 2-isopropyl-8-methylphenanthrene-3,4-dione | HTR3A |
| MOL007041 | 2-isopropyl-8-methylphenanthrene-3,4-dione | ADRA1A |
| MOL007041 | 2-isopropyl-8-methylphenanthrene-3,4-dione | ADRA1B |
| MOL007041 | 2-isopropyl-8-methylphenanthrene-3,4-dione | ADRA1D |
| MOL007041 | 2-isopropyl-8-methylphenanthrene-3,4-dione | AR |
| MOL007041 | 2-isopropyl-8-methylphenanthrene-3,4-dione | ADRB2 |
| MOL007041 | 2-isopropyl-8-methylphenanthrene-3,4-dione | CALM |
| MOL007041 | 2-isopropyl-8-methylphenanthrene-3,4-dione | CDPK2 |
| MOL007041 | 2-isopropyl-8-methylphenanthrene-3,4-dione | CCNA2 CCN1 CCNA |
| MOL007041 | 2-isopropyl-8-methylphenanthrene-3,4-dione | DRD1 |
| MOL007041 | 2-isopropyl-8-methylphenanthrene-3,4-dione | ESR1 |
| MOL007041 | 2-isopropyl-8-methylphenanthrene-3,4-dione | GABRA1 |
| MOL007041 | 2-isopropyl-8-methylphenanthrene-3,4-dione | CHRM1 |
| MOL007041 | 2-isopropyl-8-methylphenanthrene-3,4-dione | CHRM2 |
| MOL007041 | 2-isopropyl-8-methylphenanthrene-3,4-dione | CHRM3 |
| MOL007041 | 2-isopropyl-8-methylphenanthrene-3,4-dione | CHRM4 |
| MOL007041 | 2-isopropyl-8-methylphenanthrene-3,4-dione | CHRM5 |
| MOL007041 | 2-isopropyl-8-methylphenanthrene-3,4-dione | OPRM1 |
| MOL007041 | 2-isopropyl-8-methylphenanthrene-3,4-dione | CHRNA7 |
| MOL007041 | 2-isopropyl-8-methylphenanthrene-3,4-dione | NOS3 |
| MOL007041 | 2-isopropyl-8-methylphenanthrene-3,4-dione | NCOA2 |
| MOL007041 | 2-isopropyl-8-methylphenanthrene-3,4-dione | ppar gamma2 |
| MOL007041 | 2-isopropyl-8-methylphenanthrene-3,4-dione | PIK3CA |
| MOL007041 | 2-isopropyl-8-methylphenanthrene-3,4-dione | PTGS1 COX1 |
| MOL007041 | 2-isopropyl-8-methylphenanthrene-3,4-dione | PTGS2 COX2 |
| MOL007041 | 2-isopropyl-8-methylphenanthrene-3,4-dione | Pim-1 |
| MOL007041 | 2-isopropyl-8-methylphenanthrene-3,4-dione | RXRA |
| MOL007041 | 2-isopropyl-8-methylphenanthrene-3,4-dione | SCN5A |
| MOL007041 | 2-isopropyl-8-methylphenanthrene-3,4-dione | SLC6A3 DAT1 |
| MOL007041 | 2-isopropyl-8-methylphenanthrene-3,4-dione | SLC6A4 |
| MOL007045 | 3α-hydroxytanshinoneⅡa | ACHE |
| MOL007045 | 3α-hydroxytanshinoneⅡa | ADRB2 |
| MOL007045 | 3α-hydroxytanshinoneⅡa | OPRD1 OPRD |
| MOL007045 | 3α-hydroxytanshinoneⅡa | DPP4 |
| MOL007045 | 3α-hydroxytanshinoneⅡa | CHRM1 |
| MOL007045 | 3α-hydroxytanshinoneⅡa | CHRM5 |
| MOL007045 | 3α-hydroxytanshinoneⅡa | OPRM1 |
| MOL007045 | 3α-hydroxytanshinoneⅡa | CHRNA7 |
| MOL007045 | 3α-hydroxytanshinoneⅡa | NCOA1 |
| MOL007045 | 3α-hydroxytanshinoneⅡa | PTGS2 COX2 |
| MOL007045 | 3α-hydroxytanshinoneⅡa | SCN5A |
| MOL007045 | 3α-hydroxytanshinoneⅡa | PRSS1 |
| MOL007048 | (E)-3-[2-(3,4-dihydroxyphenyl)-7-hydroxy-benzofuran-4-yl]acrylic acid | HSP90 |
| MOL007048 | (E)-3-[2-(3,4-dihydroxyphenyl)-7-hydroxy-benzofuran-4-yl]acrylic acid |  |
| MOL007048 | (E)-3-[2-(3,4-dihydroxyphenyl)-7-hydroxy-benzofuran-4-yl]acrylic acid | PTGS2 COX2 |
| MOL007049 | 4-methylenemiltirone | HTR2A |
| MOL007049 | 4-methylenemiltirone | ADRA1A |
| MOL007049 | 4-methylenemiltirone | ADRA1B |
| MOL007049 | 4-methylenemiltirone | ADRA1D |
| MOL007049 | 4-methylenemiltirone | ADRA2A |
| MOL007049 | 4-methylenemiltirone | ADRA2C |
| MOL007049 | 4-methylenemiltirone | AR |
| MOL007049 | 4-methylenemiltirone | ADRB2 |
| MOL007049 | 4-methylenemiltirone | CA2 |
| MOL007049 | 4-methylenemiltirone | DRD2 |
| MOL007049 | 4-methylenemiltirone | OPRD1 OPRD |
| MOL007049 | 4-methylenemiltirone | TOP2 |
| MOL007049 | 4-methylenemiltirone | DRD1 |
| MOL007049 | 4-methylenemiltirone | ESR1 |
| MOL007049 | 4-methylenemiltirone | GABRA1 |
| MOL007049 | 4-methylenemiltirone | CHRM1 |
| MOL007049 | 4-methylenemiltirone | CHRM2 |
| MOL007049 | 4-methylenemiltirone | CHRM3 |
| MOL007049 | 4-methylenemiltirone | CHRM4 |
| MOL007049 | 4-methylenemiltirone | CHRM5 |
| MOL007049 | 4-methylenemiltirone | OPRM1 |
| MOL007049 | 4-methylenemiltirone | CHRNA7 |
| MOL007049 | 4-methylenemiltirone | NOS3 |
| MOL007049 | 4-methylenemiltirone | NCOA1 |
| MOL007049 | 4-methylenemiltirone | NCOA2 |
| MOL007049 | 4-methylenemiltirone | ppar gamma2 |
| MOL007049 | 4-methylenemiltirone | PTGS1 COX1 |
| MOL007049 | 4-methylenemiltirone | PTGS2 COX2 |
| MOL007049 | 4-methylenemiltirone | RXRA |
| MOL007049 | 4-methylenemiltirone | SCN5A |
| MOL007049 | 4-methylenemiltirone | SLC6A3 DAT1 |
| MOL007049 | 4-methylenemiltirone | SLC6A4 |
| MOL007050 | 2-(4-hydroxy-3-methoxyphenyl)-5-(3-hydroxypropyl)-7-methoxy-3-benzofurancarboxaldehyde | AR |
| MOL007050 | 2-(4-hydroxy-3-methoxyphenyl)-5-(3-hydroxypropyl)-7-methoxy-3-benzofurancarboxaldehyde | CDPK2 |
| MOL007050 | 2-(4-hydroxy-3-methoxyphenyl)-5-(3-hydroxypropyl)-7-methoxy-3-benzofurancarboxaldehyde | CCNA2 CCN1 CCNA |
| MOL007050 | 2-(4-hydroxy-3-methoxyphenyl)-5-(3-hydroxypropyl)-7-methoxy-3-benzofurancarboxaldehyde | ESR1 |
| MOL007050 | 2-(4-hydroxy-3-methoxyphenyl)-5-(3-hydroxypropyl)-7-methoxy-3-benzofurancarboxaldehyde | ESR2 |
| MOL007050 | 2-(4-hydroxy-3-methoxyphenyl)-5-(3-hydroxypropyl)-7-methoxy-3-benzofurancarboxaldehyde | GSK3B |
| MOL007050 | 2-(4-hydroxy-3-methoxyphenyl)-5-(3-hydroxypropyl)-7-methoxy-3-benzofurancarboxaldehyde | HSP90 |
| MOL007050 | 2-(4-hydroxy-3-methoxyphenyl)-5-(3-hydroxypropyl)-7-methoxy-3-benzofurancarboxaldehyde | MAPK14 |
| MOL007050 | 2-(4-hydroxy-3-methoxyphenyl)-5-(3-hydroxypropyl)-7-methoxy-3-benzofurancarboxaldehyde | NOSIP |
| MOL007050 | 2-(4-hydroxy-3-methoxyphenyl)-5-(3-hydroxypropyl)-7-methoxy-3-benzofurancarboxaldehyde | ppar gamma2 |
| MOL007050 | 2-(4-hydroxy-3-methoxyphenyl)-5-(3-hydroxypropyl)-7-methoxy-3-benzofurancarboxaldehyde | Pim-1 |
| MOL007058 | formyltanshinone | AR |
| MOL007058 | formyltanshinone | DPP4 |
| MOL007058 | formyltanshinone | NCOA1 |
| MOL007058 | formyltanshinone | PIK3CA |
| MOL007058 | formyltanshinone | PTGS2 COX2 |
| MOL007058 | formyltanshinone | RXRA |
| MOL007059 | 3-beta-Hydroxymethyllenetanshiquinone | ACHE |
| MOL007059 | 3-beta-Hydroxymethyllenetanshiquinone | ADRA1A |
| MOL007059 | 3-beta-Hydroxymethyllenetanshiquinone | ADRB2 |
| MOL007059 | 3-beta-Hydroxymethyllenetanshiquinone | CA2 |
| MOL007059 | 3-beta-Hydroxymethyllenetanshiquinone | OPRD1 OPRD |
| MOL007059 | 3-beta-Hydroxymethyllenetanshiquinone | DPP4 |
| MOL007059 | 3-beta-Hydroxymethyllenetanshiquinone | DRD1 |
| MOL007059 | 3-beta-Hydroxymethyllenetanshiquinone | HSP90 |
| MOL007059 | 3-beta-Hydroxymethyllenetanshiquinone | CHRM1 |
| MOL007059 | 3-beta-Hydroxymethyllenetanshiquinone | OPRM1 |
| MOL007059 | 3-beta-Hydroxymethyllenetanshiquinone | CHRNA7 |
| MOL007059 | 3-beta-Hydroxymethyllenetanshiquinone | NCOA1 |
| MOL007059 | 3-beta-Hydroxymethyllenetanshiquinone | PTGS2 COX2 |
| MOL007059 | 3-beta-Hydroxymethyllenetanshiquinone | RXRA |
| MOL007059 | 3-beta-Hydroxymethyllenetanshiquinone | PRSS1 |
| MOL007061 | Methylenetanshinquinone | HTR2A |
| MOL007061 | Methylenetanshinquinone | ACHE |
| MOL007061 | Methylenetanshinquinone | ADRA1A |
| MOL007061 | Methylenetanshinquinone | ADRB2 |
| MOL007061 | Methylenetanshinquinone | CA2 |
| MOL007061 | Methylenetanshinquinone | OPRD1 OPRD |
| MOL007061 | Methylenetanshinquinone | DPP4 |
| MOL007061 | Methylenetanshinquinone | DRD1 |
| MOL007061 | Methylenetanshinquinone | GABRA1 |
| MOL007061 | Methylenetanshinquinone | HSP90 |
| MOL007061 | Methylenetanshinquinone | CHRM1 |
| MOL007061 | Methylenetanshinquinone | CHRM2 |
| MOL007061 | Methylenetanshinquinone | CHRM3 |
| MOL007061 | Methylenetanshinquinone | CHRM5 |
| MOL007061 | Methylenetanshinquinone | OPRM1 |
| MOL007061 | Methylenetanshinquinone | CHRNA7 |
| MOL007061 | Methylenetanshinquinone | NCOA1 |
| MOL007061 | Methylenetanshinquinone | PTGS2 COX2 |
| MOL007061 | Methylenetanshinquinone | RXRA |
| MOL007061 | Methylenetanshinquinone | SCN5A |
| MOL007061 | Methylenetanshinquinone | SLC6A4 |
| MOL007061 | Methylenetanshinquinone | PRSS1 |
| MOL007063 | przewalskin a | NR3C1 |
| MOL007063 | przewalskin a | NR3C2 |
| MOL007064 | przewalskin b | NR3C1 |
| MOL007064 | przewalskin b | NR3C2 |
| MOL007064 | przewalskin b | NCOA1 |
| MOL007064 | przewalskin b | NCOA2 |
| MOL007064 | przewalskin b | PGR |
| MOL007064 | przewalskin b | PTGS2 COX2 |
| MOL007068 | Przewaquinone B | DPP4 |
| MOL007068 | Przewaquinone B | HSP90 |
| MOL007068 | Przewaquinone B | NCOA1 |
| MOL007068 | Przewaquinone B | PIK3CA |
| MOL007068 | Przewaquinone B | PTGS2 COX2 |
| MOL007068 | Przewaquinone B | RXRA |
| MOL007068 | Przewaquinone B | PRSS1 |
| MOL007069 | przewaquinone c | ACHE |
| MOL007069 | przewaquinone c | ADRA1A |
| MOL007069 | przewaquinone c | ADRB2 |
| MOL007069 | przewaquinone c | CA2 |
| MOL007069 | przewaquinone c | OPRD1 OPRD |
| MOL007069 | przewaquinone c | DPP4 |
| MOL007069 | przewaquinone c | DRD1 |
| MOL007069 | przewaquinone c | GABRA1 |
| MOL007069 | przewaquinone c | HSP90 |
| MOL007069 | przewaquinone c | CHRM1 |
| MOL007069 | przewaquinone c | CHRM2 |
| MOL007069 | przewaquinone c | CHRM3 |
| MOL007069 | przewaquinone c | CHRM4 |
| MOL007069 | przewaquinone c | CHRM5 |
| MOL007069 | przewaquinone c | OPRM1 |
| MOL007069 | przewaquinone c | CHRNA7 |
| MOL007069 | przewaquinone c | NCOA1 |
| MOL007069 | przewaquinone c | PIK3CA |
| MOL007069 | przewaquinone c | PTGS1 COX1 |
| MOL007069 | przewaquinone c | PTGS2 COX2 |
| MOL007069 | przewaquinone c | SCN5A |
| MOL007070 | (6S,7R)-6,7-dihydroxy-1,6-dimethyl-8,9-dihydro-7H-naphtho[8,7-g]benzofuran-10,11-dione | ACHE |
| MOL007070 | (6S,7R)-6,7-dihydroxy-1,6-dimethyl-8,9-dihydro-7H-naphtho[8,7-g]benzofuran-10,11-dione | CA2 |
| MOL007070 | (6S,7R)-6,7-dihydroxy-1,6-dimethyl-8,9-dihydro-7H-naphtho[8,7-g]benzofuran-10,11-dione | DPP4 |
| MOL007070 | (6S,7R)-6,7-dihydroxy-1,6-dimethyl-8,9-dihydro-7H-naphtho[8,7-g]benzofuran-10,11-dione | HSP90 |
| MOL007070 | (6S,7R)-6,7-dihydroxy-1,6-dimethyl-8,9-dihydro-7H-naphtho[8,7-g]benzofuran-10,11-dione | NCOA1 |
| MOL007070 | (6S,7R)-6,7-dihydroxy-1,6-dimethyl-8,9-dihydro-7H-naphtho[8,7-g]benzofuran-10,11-dione | PTGS2 COX2 |
| MOL007070 | (6S,7R)-6,7-dihydroxy-1,6-dimethyl-8,9-dihydro-7H-naphtho[8,7-g]benzofuran-10,11-dione | PRSS1 |
| MOL007071 | przewaquinone f | DPP4 |
| MOL007071 | przewaquinone f | NCOA1 |
| MOL007071 | przewaquinone f | PTGS2 COX2 |
| MOL007071 | przewaquinone f | PRSS1 |
| MOL007077 | sclareol | PTGS2 COX2 |
| MOL007079 | tanshinaldehyde | HTR2A |
| MOL007079 | tanshinaldehyde | ACHE |
| MOL007079 | tanshinaldehyde | ADRB2 |
| MOL007079 | tanshinaldehyde | OPRD1 OPRD |
| MOL007079 | tanshinaldehyde | DPP4 |
| MOL007079 | tanshinaldehyde | DRD1 |
| MOL007079 | tanshinaldehyde | CHRM1 |
| MOL007079 | tanshinaldehyde | OPRM1 |
| MOL007079 | tanshinaldehyde | CHRNA7 |
| MOL007079 | tanshinaldehyde | NCOA1 |
| MOL007079 | tanshinaldehyde | PTGS2 COX2 |
| MOL007079 | tanshinaldehyde | PRSS1 |
| MOL007081 | Danshenol B | CA2 |
| MOL007081 | Danshenol B | TOP2 |
| MOL007081 | Danshenol B | NR3C1 |
| MOL007081 | Danshenol B | HSP90 |
| MOL007081 | Danshenol B | OPRM1 |
| MOL007081 | Danshenol B | NCOA1 |
| MOL007081 | Danshenol B | PGR |
| MOL007081 | Danshenol B | PTGS2 COX2 |
| MOL007082 | Danshenol A | KCNMA1 |
| MOL007082 | Danshenol A | F10 |
| MOL007082 | Danshenol A | NCOA1 |
| MOL007082 | Danshenol A | PIK3CA |
| MOL007082 | Danshenol A | KCNH2 ERG ERG1 HERG |
| MOL007082 | Danshenol A | PTGS1 COX1 |
| MOL007082 | Danshenol A | PTGS2 COX2 |
| MOL007082 | Danshenol A | RXRA |
| MOL007082 | Danshenol A | SCN5A |
| MOL007085 | Salvilenone | HTR3A |
| MOL007085 | Salvilenone | AR |
| MOL007085 | Salvilenone | ESR1 |
| MOL007085 | Salvilenone | ESR2 |
| MOL007085 | Salvilenone | CHRM5 |
| MOL007085 | Salvilenone | PTGS1 COX1 |
| MOL007085 | Salvilenone | PTGS2 COX2 |
| MOL007085 | Salvilenone | Pim-1 |
| MOL007088 | cryptotanshinone | ADRA1A |
| MOL007088 | cryptotanshinone | ADRA1B |
| MOL007088 | cryptotanshinone | ADRA1D |
| MOL007088 | cryptotanshinone | APP |
| MOL007088 | cryptotanshinone | BIRC5 |
| MOL007088 | cryptotanshinone | BCL2L1 |
| MOL007088 | cryptotanshinone | ADRB2 |
| MOL007088 | cryptotanshinone | CA2 |
| MOL007088 | cryptotanshinone | OPRD1 OPRD |
| MOL007088 | cryptotanshinone | TOP2 |
| MOL007088 | cryptotanshinone | DRD1 |
| MOL007088 | cryptotanshinone | EDN1 |
| MOL007088 | cryptotanshinone | CCND1 |
| MOL007088 | cryptotanshinone | GABRA1 |
| MOL007088 | cryptotanshinone | CHRM1 |
| MOL007088 | cryptotanshinone | CHRM2 |
| MOL007088 | cryptotanshinone | CHRM3 |
| MOL007088 | cryptotanshinone | CHRM4 |
| MOL007088 | cryptotanshinone | CHRM5 |
| MOL007088 | cryptotanshinone | OPRM1 |
| MOL007088 | cryptotanshinone | CHRNA7 |
| MOL007088 | cryptotanshinone | NCOA1 |
| MOL007088 | cryptotanshinone | NCOA2 |
| MOL007088 | cryptotanshinone | PGR |
| MOL007088 | cryptotanshinone | PTGS1 COX1 |
| MOL007088 | cryptotanshinone | PTGS2 COX2 |
| MOL007088 | cryptotanshinone | STAT3 APRF |
| MOL007088 | cryptotanshinone | SCN5A |
| MOL007088 | cryptotanshinone | RELA |
| MOL007088 | cryptotanshinone | TNF |
| MOL007093 | dan-shexinkum d | ACHE |
| MOL007093 | dan-shexinkum d | ADRA1B |
| MOL007093 | dan-shexinkum d | AR |
| MOL007093 | dan-shexinkum d | ADRB2 |
| MOL007093 | dan-shexinkum d | CALM |
| MOL007093 | dan-shexinkum d | CA2 |
| MOL007093 | dan-shexinkum d | CDPK2 |
| MOL007093 | dan-shexinkum d | F10 |
| MOL007093 | dan-shexinkum d | CCNA2 CCN1 CCNA |
| MOL007093 | dan-shexinkum d | DPP4 |
| MOL007093 | dan-shexinkum d | TOP2 |
| MOL007093 | dan-shexinkum d | ESR1 |
| MOL007093 | dan-shexinkum d | ESR2 |
| MOL007093 | dan-shexinkum d | GSK3B |
| MOL007093 | dan-shexinkum d | CHRM1 |
| MOL007093 | dan-shexinkum d | NOSIP |
| MOL007093 | dan-shexinkum d | NCOA1 |
| MOL007093 | dan-shexinkum d | NCOA2 |
| MOL007093 | dan-shexinkum d | ppar gamma2 |
| MOL007093 | dan-shexinkum d | KCNH2 ERG ERG1 HERG |
| MOL007093 | dan-shexinkum d | PTGS1 COX1 |
| MOL007093 | dan-shexinkum d | PTGS2 COX2 |
| MOL007093 | dan-shexinkum d | Pim-1 |
| MOL007093 | dan-shexinkum d | RXRA |
| MOL007093 | dan-shexinkum d | CHEK1 |
| MOL007093 | dan-shexinkum d | SCN5A |
| MOL007093 | dan-shexinkum d | PRSS1 |
| MOL007094 | danshenspiroketallactone | ACHE |
| MOL007094 | danshenspiroketallactone | ADRA1A |
| MOL007094 | danshenspiroketallactone | ADRA1B |
| MOL007094 | danshenspiroketallactone | ADRA1D |
| MOL007094 | danshenspiroketallactone | ADRB2 |
| MOL007094 | danshenspiroketallactone | CA2 |
| MOL007094 | danshenspiroketallactone | DPP4 |
| MOL007094 | danshenspiroketallactone | DRD1 |
| MOL007094 | danshenspiroketallactone | ESR1 |
| MOL007094 | danshenspiroketallactone | GABRA1 |
| MOL007094 | danshenspiroketallactone | HSP90 |
| MOL007094 | danshenspiroketallactone | CHRM1 |
| MOL007094 | danshenspiroketallactone | CHRM2 |
| MOL007094 | danshenspiroketallactone | CHRM3 |
| MOL007094 | danshenspiroketallactone | CHRM4 |
| MOL007094 | danshenspiroketallactone | CHRM5 |
| MOL007094 | danshenspiroketallactone | OPRM1 |
| MOL007094 | danshenspiroketallactone | CHRNA2 |
| MOL007094 | danshenspiroketallactone | PTGS1 COX1 |
| MOL007094 | danshenspiroketallactone | PTGS2 COX2 |
| MOL007094 | danshenspiroketallactone | RXRA |
| MOL007094 | danshenspiroketallactone | SCN5A |
| MOL007094 | danshenspiroketallactone | SLC6A4 |
| MOL007098 | deoxyneocryptotanshinone | ADRA1A |
| MOL007098 | deoxyneocryptotanshinone | ADRA1B |
| MOL007098 | deoxyneocryptotanshinone | ADRA1D |
| MOL007098 | deoxyneocryptotanshinone | AR |
| MOL007098 | deoxyneocryptotanshinone | ADRB2 |
| MOL007098 | deoxyneocryptotanshinone | CA2 |
| MOL007098 | deoxyneocryptotanshinone | CDPK2 |
| MOL007098 | deoxyneocryptotanshinone | OPRD1 OPRD |
| MOL007098 | deoxyneocryptotanshinone | TOP2 |
| MOL007098 | deoxyneocryptotanshinone | DRD1 |
| MOL007098 | deoxyneocryptotanshinone | ESR1 |
| MOL007098 | deoxyneocryptotanshinone | GSK3B |
| MOL007098 | deoxyneocryptotanshinone | CHRM1 |
| MOL007098 | deoxyneocryptotanshinone | CHRM2 |
| MOL007098 | deoxyneocryptotanshinone | CHRM3 |
| MOL007098 | deoxyneocryptotanshinone | CHRM4 |
| MOL007098 | deoxyneocryptotanshinone | CHRM5 |
| MOL007098 | deoxyneocryptotanshinone | OPRM1 |
| MOL007098 | deoxyneocryptotanshinone | CHRNA7 |
| MOL007098 | deoxyneocryptotanshinone | NOS3 |
| MOL007098 | deoxyneocryptotanshinone | NCOA1 |
| MOL007098 | deoxyneocryptotanshinone | NCOA2 |
| MOL007098 | deoxyneocryptotanshinone | PTGS1 COX1 |
| MOL007098 | deoxyneocryptotanshinone | PTGS2 COX2 |
| MOL007098 | deoxyneocryptotanshinone | Pim-1 |
| MOL007098 | deoxyneocryptotanshinone | RXRA |
| MOL007098 | deoxyneocryptotanshinone | SCN5A |
| MOL007100 | dihydrotanshinlactone | HTR2A |
| MOL007100 | dihydrotanshinlactone | HTR3A |
| MOL007100 | dihydrotanshinlactone | ACHE |
| MOL007100 | dihydrotanshinlactone | ADRA1A |
| MOL007100 | dihydrotanshinlactone | ADRA1B |
| MOL007100 | dihydrotanshinlactone | ADRA1D |
| MOL007100 | dihydrotanshinlactone | AR |
| MOL007100 | dihydrotanshinlactone | ADRB2 |
| MOL007100 | dihydrotanshinlactone | CA2 |
| MOL007100 | dihydrotanshinlactone | PDE3A |
| MOL007100 | dihydrotanshinlactone | CCNA2 CCN1 CCNA |
| MOL007100 | dihydrotanshinlactone | DPP4 |
| MOL007100 | dihydrotanshinlactone | DRD1 |
| MOL007100 | dihydrotanshinlactone | ESR1 |
| MOL007100 | dihydrotanshinlactone | GABRA1 |
| MOL007100 | dihydrotanshinlactone | GSK3B |
| MOL007100 | dihydrotanshinlactone |  |
| MOL007100 | dihydrotanshinlactone | CHRM1 |
| MOL007100 | dihydrotanshinlactone | CHRM3 |
| MOL007100 | dihydrotanshinlactone | CHRM5 |
| MOL007100 | dihydrotanshinlactone | OPRM1 |
| MOL007100 | dihydrotanshinlactone | CHRNA7 |
| MOL007100 | dihydrotanshinlactone | NOSIP |
| MOL007100 | dihydrotanshinlactone | ppar gamma2 |
| MOL007100 | dihydrotanshinlactone | PIK3CA |
| MOL007100 | dihydrotanshinlactone | PTGS1 COX1 |
| MOL007100 | dihydrotanshinlactone | PTGS2 COX2 |
| MOL007100 | dihydrotanshinlactone | Pim-1 |
| MOL007100 | dihydrotanshinlactone | RXRA |
| MOL007100 | dihydrotanshinlactone | SCN5A |
| MOL007100 | dihydrotanshinlactone | SLC6A3 DAT1 |
| MOL007100 | dihydrotanshinlactone | SLC6A4 |
| MOL007100 | dihydrotanshinlactone | PRSS1 |
| MOL007101 | dihydrotanshinoneⅠ | HTR3A |
| MOL007101 | dihydrotanshinoneⅠ | ADRA1A |
| MOL007101 | dihydrotanshinoneⅠ | ADRA1B |
| MOL007101 | dihydrotanshinoneⅠ | ADRB2 |
| MOL007101 | dihydrotanshinoneⅠ | CALM |
| MOL007101 | dihydrotanshinoneⅠ | GABRA1 |
| MOL007101 | dihydrotanshinoneⅠ | HSP90 |
| MOL007101 | dihydrotanshinoneⅠ | CHRNA7 |
| MOL007101 | dihydrotanshinoneⅠ | NCOA1 |
| MOL007101 | dihydrotanshinoneⅠ | NCOA2 |
| MOL007101 | dihydrotanshinoneⅠ | PIK3CA |
| MOL007101 | dihydrotanshinoneⅠ | PTGS1 COX1 |
| MOL007101 | dihydrotanshinoneⅠ | PTGS2 COX2 |
| MOL007101 | dihydrotanshinoneⅠ | RXRA |
| MOL007101 | dihydrotanshinoneⅠ | SCN5A |
| MOL007105 | epidanshenspiroketallactone | HTR2A |
| MOL007105 | epidanshenspiroketallactone | ADRA1A |
| MOL007105 | epidanshenspiroketallactone | ADRA1B |
| MOL007105 | epidanshenspiroketallactone | ADRA1D |
| MOL007105 | epidanshenspiroketallactone | ADRB2 |
| MOL007105 | epidanshenspiroketallactone | CDPK2 |
| MOL007105 | epidanshenspiroketallactone | PDE3A |
| MOL007105 | epidanshenspiroketallactone | OPRD1 OPRD |
| MOL007105 | epidanshenspiroketallactone | DRD1 |
| MOL007105 | epidanshenspiroketallactone | ESR1 |
| MOL007105 | epidanshenspiroketallactone | GABRA1 |
| MOL007105 | epidanshenspiroketallactone | HSP90 |
| MOL007105 | epidanshenspiroketallactone | CHRM1 |
| MOL007105 | epidanshenspiroketallactone | CHRM2 |
| MOL007105 | epidanshenspiroketallactone | CHRM3 |
| MOL007105 | epidanshenspiroketallactone | CHRM4 |
| MOL007105 | epidanshenspiroketallactone | CHRM5 |
| MOL007105 | epidanshenspiroketallactone | OPRM1 |
| MOL007105 | epidanshenspiroketallactone | CHRNA7 |
| MOL007105 | epidanshenspiroketallactone | NOS3 |
| MOL007105 | epidanshenspiroketallactone | PTGS1 COX1 |
| MOL007105 | epidanshenspiroketallactone | PTGS2 COX2 |
| MOL007105 | epidanshenspiroketallactone | Pim-1 |
| MOL007105 | epidanshenspiroketallactone | RXRA |
| MOL007105 | epidanshenspiroketallactone | SCN5A |
| MOL007105 | epidanshenspiroketallactone | SLC6A4 |
| MOL007107 | C09092 | ACHE |
| MOL007107 | C09092 | ADRA1A |
| MOL007107 | C09092 | ADRA1B |
| MOL007107 | C09092 | ADRA1D |
| MOL007107 | C09092 | ADRB2 |
| MOL007107 | C09092 | CA2 |
| MOL007107 | C09092 | CHRM1 |
| MOL007107 | C09092 | CHRM2 |
| MOL007107 | C09092 | CHRM3 |
| MOL007107 | C09092 | OPRM1 |
| MOL007107 | C09092 | SCN5A |
| MOL007108 | isocryptotanshi-none | ACHE |
| MOL007108 | isocryptotanshi-none | ADRA1A |
| MOL007108 | isocryptotanshi-none | ADRA1B |
| MOL007108 | isocryptotanshi-none | ADRA1D |
| MOL007108 | isocryptotanshi-none | AR |
| MOL007108 | isocryptotanshi-none | ADRB2 |
| MOL007108 | isocryptotanshi-none | CA2 |
| MOL007108 | isocryptotanshi-none | CDPK2 |
| MOL007108 | isocryptotanshi-none | F10 |
| MOL007108 | isocryptotanshi-none | DRD2 |
| MOL007108 | isocryptotanshi-none | OPRD1 OPRD |
| MOL007108 | isocryptotanshi-none | TOP2 |
| MOL007108 | isocryptotanshi-none | DRD1 |
| MOL007108 | isocryptotanshi-none | ESR1 |
| MOL007108 | isocryptotanshi-none | GABRA1 |
| MOL007108 | isocryptotanshi-none | CHRM1 |
| MOL007108 | isocryptotanshi-none | CHRM2 |
| MOL007108 | isocryptotanshi-none | CHRM3 |
| MOL007108 | isocryptotanshi-none | CHRM4 |
| MOL007108 | isocryptotanshi-none | CHRM5 |
| MOL007108 | isocryptotanshi-none | OPRM1 |
| MOL007108 | isocryptotanshi-none | CHRNA7 |
| MOL007108 | isocryptotanshi-none | NOSIP |
| MOL007108 | isocryptotanshi-none | NCOA1 |
| MOL007108 | isocryptotanshi-none | NCOA2 |
| MOL007108 | isocryptotanshi-none | PTGS1 COX1 |
| MOL007108 | isocryptotanshi-none | PTGS2 COX2 |
| MOL007108 | isocryptotanshi-none | Pim-1 |
| MOL007108 | isocryptotanshi-none | RXRA |
| MOL007108 | isocryptotanshi-none | SCN5A |
| MOL007108 | isocryptotanshi-none | PRSS1 |
| MOL007111 | Isotanshinone II | ACHE |
| MOL007111 | Isotanshinone II | ADRA1A |
| MOL007111 | Isotanshinone II | AR |
| MOL007111 | Isotanshinone II | ADRB2 |
| MOL007111 | Isotanshinone II | CDPK2 |
| MOL007111 | Isotanshinone II | CCNA2 CCN1 CCNA |
| MOL007111 | Isotanshinone II | OPRD1 OPRD |
| MOL007111 | Isotanshinone II | DPP4 |
| MOL007111 | Isotanshinone II | DRD1 |
| MOL007111 | Isotanshinone II | ESR1 |
| MOL007111 | Isotanshinone II | ESR2 |
| MOL007111 | Isotanshinone II | GABRA1 |
| MOL007111 | Isotanshinone II | GSK3B |
| MOL007111 | Isotanshinone II | CHRM1 |
| MOL007111 | Isotanshinone II | CHRM2 |
| MOL007111 | Isotanshinone II | CHRM3 |
| MOL007111 | Isotanshinone II | CHRM5 |
| MOL007111 | Isotanshinone II | OPRM1 |
| MOL007111 | Isotanshinone II | CHRNA7 |
| MOL007111 | Isotanshinone II | NOSIP |
| MOL007111 | Isotanshinone II | PTGS2 COX2 |
| MOL007111 | Isotanshinone II | Pim-1 |
| MOL007111 | Isotanshinone II | RXRA |
| MOL007111 | Isotanshinone II | CHEK1 |
| MOL007111 | Isotanshinone II | SCN5A |
| MOL007115 | manool | NCOA2 |
| MOL007119 | miltionone Ⅰ | HTR2A |
| MOL007119 | miltionone Ⅰ | ADRA1A |
| MOL007119 | miltionone Ⅰ | ADRA1B |
| MOL007119 | miltionone Ⅰ | AR |
| MOL007119 | miltionone Ⅰ | ADRB2 |
| MOL007119 | miltionone Ⅰ | BQ8482_111389 |
| MOL007119 | miltionone Ⅰ | CA2 |
| MOL007119 | miltionone Ⅰ | CDPK2 |
| MOL007119 | miltionone Ⅰ | F10 |
| MOL007119 | miltionone Ⅰ | CCNA2 CCN1 CCNA |
| MOL007119 | miltionone Ⅰ | OPRD1 OPRD |
| MOL007119 | miltionone Ⅰ | TOP2 |
| MOL007119 | miltionone Ⅰ | ESR1 |
| MOL007119 | miltionone Ⅰ | NR3C1 |
| MOL007119 | miltionone Ⅰ | GSK3B |
| MOL007119 | miltionone Ⅰ | CHRM1 |
| MOL007119 | miltionone Ⅰ | CHRM2 |
| MOL007119 | miltionone Ⅰ | CHRM3 |
| MOL007119 | miltionone Ⅰ | OPRM1 |
| MOL007119 | miltionone Ⅰ | CHRNA7 |
| MOL007119 | miltionone Ⅰ | NCOA1 |
| MOL007119 | miltionone Ⅰ | NCOA2 |
| MOL007119 | miltionone Ⅰ | PTGS1 COX1 |
| MOL007119 | miltionone Ⅰ | PTGS2 COX2 |
| MOL007119 | miltionone Ⅰ | Pim-1 |
| MOL007119 | miltionone Ⅰ | RXRA |
| MOL007119 | miltionone Ⅰ | SCN5A |
| MOL007120 | miltionone Ⅱ | ACHE |
| MOL007120 | miltionone Ⅱ | CA2 |
| MOL007120 | miltionone Ⅱ | NR3C1 |
| MOL007120 | miltionone Ⅱ | NCOA1 |
| MOL007120 | miltionone Ⅱ | NCOA2 |
| MOL007120 | miltionone Ⅱ | PGR |
| MOL007120 | miltionone Ⅱ | PTGS2 COX2 |
| MOL007121 | miltipolone | ACHE |
| MOL007121 | miltipolone | ESR1 |
| MOL007122 | Miltirone | ADRA1A |
| MOL007122 | Miltirone | ADRA1B |
| MOL007122 | Miltirone | ADRA1D |
| MOL007122 | Miltirone | ADRA2C |
| MOL007122 | Miltirone | AR |
| MOL007122 | Miltirone | ADRB2 |
| MOL007122 | Miltirone | CA2 |
| MOL007122 | Miltirone | DRD5 DRD1B DRD1L2 |
| MOL007122 | Miltirone | OPRD1 OPRD |
| MOL007122 | Miltirone | TOP2 |
| MOL007122 | Miltirone | DRD1 |
| MOL007122 | Miltirone | ESR1 |
| MOL007122 | Miltirone | CHRM1 |
| MOL007122 | Miltirone | CHRM2 |
| MOL007122 | Miltirone | CHRM3 |
| MOL007122 | Miltirone | CHRM4 |
| MOL007122 | Miltirone | CHRM5 |
| MOL007122 | Miltirone | OPRM1 |
| MOL007122 | Miltirone | CHRNA7 |
| MOL007122 | Miltirone | NOS3 |
| MOL007122 | Miltirone | NCOA2 |
| MOL007122 | Miltirone | PTGS1 COX1 |
| MOL007122 | Miltirone | PTGS2 COX2 |
| MOL007122 | Miltirone | RXRA |
| MOL007122 | Miltirone | SCN5A |
| MOL007122 | Miltirone | SLC6A3 DAT1 |
| MOL007124 | neocryptotanshinone ii | ADRA1A |
| MOL007124 | neocryptotanshinone ii | ADRA1B |
| MOL007124 | neocryptotanshinone ii | ADRA1D |
| MOL007124 | neocryptotanshinone ii | AR |
| MOL007124 | neocryptotanshinone ii | ADRB2 |
| MOL007124 | neocryptotanshinone ii | CA2 |
| MOL007124 | neocryptotanshinone ii | CDPK2 |
| MOL007124 | neocryptotanshinone ii | PDE3A |
| MOL007124 | neocryptotanshinone ii | CCNA2 CCN1 CCNA |
| MOL007124 | neocryptotanshinone ii | OPRD1 OPRD |
| MOL007124 | neocryptotanshinone ii | DRD1 |
| MOL007124 | neocryptotanshinone ii | ESR1 |
| MOL007124 | neocryptotanshinone ii | GABRA1 |
| MOL007124 | neocryptotanshinone ii | GSK3B |
| MOL007124 | neocryptotanshinone ii | HSP90 |
| MOL007124 | neocryptotanshinone ii | CHRM1 |
| MOL007124 | neocryptotanshinone ii | CHRM2 |
| MOL007124 | neocryptotanshinone ii | CHRM3 |
| MOL007124 | neocryptotanshinone ii | CHRM4 |
| MOL007124 | neocryptotanshinone ii | OPRM1 |
| MOL007124 | neocryptotanshinone ii | CHRNA7 |
| MOL007124 | neocryptotanshinone ii | NOS3 |
| MOL007124 | neocryptotanshinone ii | PTGS1 COX1 |
| MOL007124 | neocryptotanshinone ii | PTGS2 COX2 |
| MOL007124 | neocryptotanshinone ii | Pim-1 |
| MOL007124 | neocryptotanshinone ii | RXRA |
| MOL007124 | neocryptotanshinone ii | SCN5A |
| MOL007124 | neocryptotanshinone ii | SLC6A3 DAT1 |
| MOL007124 | neocryptotanshinone ii | SLC6A4 |
| MOL007125 | neocryptotanshinone | ADRA1B |
| MOL007125 | neocryptotanshinone | ADRA1D |
| MOL007125 | neocryptotanshinone | ADRB2 |
| MOL007125 | neocryptotanshinone | CA2 |
| MOL007125 | neocryptotanshinone | TOP2 |
| MOL007125 | neocryptotanshinone | CHRM1 |
| MOL007125 | neocryptotanshinone | CHRM3 |
| MOL007125 | neocryptotanshinone | OPRM1 |
| MOL007125 | neocryptotanshinone | CHRNA7 |
| MOL007125 | neocryptotanshinone | NCOA1 |
| MOL007125 | neocryptotanshinone | NCOA2 |
| MOL007125 | neocryptotanshinone | ppar gamma2 |
| MOL007125 | neocryptotanshinone | PTGS1 COX1 |
| MOL007125 | neocryptotanshinone | PTGS2 COX2 |
| MOL007125 | neocryptotanshinone | SCN5A |
| MOL007127 | 1-methyl-8,9-dihydro-7H-naphtho[5,6-g]benzofuran-6,10,11-trione | ACHE |
| MOL007127 | 1-methyl-8,9-dihydro-7H-naphtho[5,6-g]benzofuran-6,10,11-trione | ADRA1A |
| MOL007127 | 1-methyl-8,9-dihydro-7H-naphtho[5,6-g]benzofuran-6,10,11-trione | ADRB2 |
| MOL007127 | 1-methyl-8,9-dihydro-7H-naphtho[5,6-g]benzofuran-6,10,11-trione | CA2 |
| MOL007127 | 1-methyl-8,9-dihydro-7H-naphtho[5,6-g]benzofuran-6,10,11-trione | DPP4 |
| MOL007127 | 1-methyl-8,9-dihydro-7H-naphtho[5,6-g]benzofuran-6,10,11-trione | DRD1 |
| MOL007127 | 1-methyl-8,9-dihydro-7H-naphtho[5,6-g]benzofuran-6,10,11-trione | GABRA1 |
| MOL007127 | 1-methyl-8,9-dihydro-7H-naphtho[5,6-g]benzofuran-6,10,11-trione | HSP90 |
| MOL007127 | 1-methyl-8,9-dihydro-7H-naphtho[5,6-g]benzofuran-6,10,11-trione | CHRM3 |
| MOL007127 | 1-methyl-8,9-dihydro-7H-naphtho[5,6-g]benzofuran-6,10,11-trione | CHRM5 |
| MOL007127 | 1-methyl-8,9-dihydro-7H-naphtho[5,6-g]benzofuran-6,10,11-trione | OPRM1 |
| MOL007127 | 1-methyl-8,9-dihydro-7H-naphtho[5,6-g]benzofuran-6,10,11-trione | CHRNA7 |
| MOL007127 | 1-methyl-8,9-dihydro-7H-naphtho[5,6-g]benzofuran-6,10,11-trione | NCOA1 |
| MOL007127 | 1-methyl-8,9-dihydro-7H-naphtho[5,6-g]benzofuran-6,10,11-trione | PIK3CA |
| MOL007127 | 1-methyl-8,9-dihydro-7H-naphtho[5,6-g]benzofuran-6,10,11-trione | PTGS1 COX1 |
| MOL007127 | 1-methyl-8,9-dihydro-7H-naphtho[5,6-g]benzofuran-6,10,11-trione | PTGS2 COX2 |
| MOL007127 | 1-methyl-8,9-dihydro-7H-naphtho[5,6-g]benzofuran-6,10,11-trione | RXRA |
| MOL007127 | 1-methyl-8,9-dihydro-7H-naphtho[5,6-g]benzofuran-6,10,11-trione | SCN5A |
| MOL007130 | prolithospermic acid | AR |
| MOL007130 | prolithospermic acid | CALM |
| MOL007130 | prolithospermic acid | ESR1 |
| MOL007130 | prolithospermic acid | HSP90 |
| MOL007130 | prolithospermic acid |  |
| MOL007130 | prolithospermic acid | NOSIP |
| MOL007130 | prolithospermic acid | PTGS1 COX1 |
| MOL007130 | prolithospermic acid | PTGS2 COX2 |
| MOL007130 | prolithospermic acid | PRSS1 |
| MOL007132 | (2R)-3-(3,4-dihydroxyphenyl)-2-[(Z)-3-(3,4-dihydroxyphenyl)acryloyl]oxy-propionic acid | AR |
| MOL007132 | (2R)-3-(3,4-dihydroxyphenyl)-2-[(Z)-3-(3,4-dihydroxyphenyl)acryloyl]oxy-propionic acid | CCNA2 CCN1 CCNA |
| MOL007132 | (2R)-3-(3,4-dihydroxyphenyl)-2-[(Z)-3-(3,4-dihydroxyphenyl)acryloyl]oxy-propionic acid | DPP4 |
| MOL007132 | (2R)-3-(3,4-dihydroxyphenyl)-2-[(Z)-3-(3,4-dihydroxyphenyl)acryloyl]oxy-propionic acid | ESR1 |
| MOL007132 | (2R)-3-(3,4-dihydroxyphenyl)-2-[(Z)-3-(3,4-dihydroxyphenyl)acryloyl]oxy-propionic acid | ppar gamma2 |
| MOL007132 | (2R)-3-(3,4-dihydroxyphenyl)-2-[(Z)-3-(3,4-dihydroxyphenyl)acryloyl]oxy-propionic acid | PTGS2 COX2 |
| MOL007132 | (2R)-3-(3,4-dihydroxyphenyl)-2-[(Z)-3-(3,4-dihydroxyphenyl)acryloyl]oxy-propionic acid | PRSS1 |
| MOL007141 | salvianolic acid g | PTGS2 COX2 |
| MOL007142 | salvianolic acid j | F7 |
| MOL007142 | salvianolic acid j |  |
| MOL007142 | salvianolic acid j | PRSS1 |
| MOL007143 | salvilenone Ⅰ | ACHE |
| MOL007143 | salvilenone Ⅰ | NR3C1 |
| MOL007143 | salvilenone Ⅰ |  |
| MOL007143 | salvilenone Ⅰ | NCOA1 |
| MOL007143 | salvilenone Ⅰ | NCOA2 |
| MOL007143 | salvilenone Ⅰ | PGR |
| MOL007143 | salvilenone Ⅰ | PTGS2 COX2 |
| MOL007143 | salvilenone Ⅰ | RXRA |
| MOL007145 | salviolone | HTR1A |
| MOL007145 | salviolone | HTR1B |
| MOL007145 | salviolone | HTR2A |
| MOL007145 | salviolone | HTR2C |
| MOL007145 | salviolone | HTR3A |
| MOL007145 | salviolone | ACHE |
| MOL007145 | salviolone | ADRA1A |
| MOL007145 | salviolone | ADRA1B |
| MOL007145 | salviolone | ADRA2A |
| MOL007145 | salviolone | ADRA2B |
| MOL007145 | salviolone | ADRB2 |
| MOL007145 | salviolone | PDE3A |
| MOL007145 | salviolone | DRD5 DRD1B DRD1L2 |
| MOL007145 | salviolone | DRD2 |
| MOL007145 | salviolone | OPRD1 OPRD |
| MOL007145 | salviolone | DRD1 |
| MOL007145 | salviolone | GABRA1 |
| MOL007145 | salviolone | GABRE |
| MOL007145 | salviolone | GABRG3 |
| MOL007145 | salviolone | GABRA2 |
| MOL007145 | salviolone | GABRA3 |
| MOL007145 | salviolone | GABRA5 |
| MOL007145 | salviolone | GABRA6 |
| MOL007145 | salviolone | CHRM1 |
| MOL007145 | salviolone | CHRM2 |
| MOL007145 | salviolone | CHRM3 |
| MOL007145 | salviolone | CHRM4 |
| MOL007145 | salviolone | CHRM5 |
| MOL007145 | salviolone | OPRM1 |
| MOL007145 | salviolone | CHRNA7 |
| MOL007145 | salviolone | CHRNA2 |
| MOL007145 | salviolone | PTGS1 COX1 |
| MOL007145 | salviolone | PTGS2 COX2 |
| MOL007145 | salviolone | SCN5A |
| MOL007145 | salviolone | SLC6A3 DAT1 |
| MOL007145 | salviolone | SLC6A2 |
| MOL007145 | salviolone | SLC6A4 |
| MOL007150 | (6S)-6-hydroxy-1-methyl-6-methylol-8,9-dihydro-7H-naphtho[8,7-g]benzofuran-10,11-quinone | ACHE |
| MOL007150 | (6S)-6-hydroxy-1-methyl-6-methylol-8,9-dihydro-7H-naphtho[8,7-g]benzofuran-10,11-quinone | CA2 |
| MOL007150 | (6S)-6-hydroxy-1-methyl-6-methylol-8,9-dihydro-7H-naphtho[8,7-g]benzofuran-10,11-quinone | DPP4 |
| MOL007150 | (6S)-6-hydroxy-1-methyl-6-methylol-8,9-dihydro-7H-naphtho[8,7-g]benzofuran-10,11-quinone | HSP90 |
| MOL007150 | (6S)-6-hydroxy-1-methyl-6-methylol-8,9-dihydro-7H-naphtho[8,7-g]benzofuran-10,11-quinone | NCOA1 |
| MOL007150 | (6S)-6-hydroxy-1-methyl-6-methylol-8,9-dihydro-7H-naphtho[8,7-g]benzofuran-10,11-quinone | PTGS2 COX2 |
| MOL007150 | (6S)-6-hydroxy-1-methyl-6-methylol-8,9-dihydro-7H-naphtho[8,7-g]benzofuran-10,11-quinone | PRSS1 |
| MOL007151 | Tanshindiol B | ACHE |
| MOL007151 | Tanshindiol B | CA2 |
| MOL007151 | Tanshindiol B | DPP4 |
| MOL007151 | Tanshindiol B | HSP90 |
| MOL007151 | Tanshindiol B | NCOA1 |
| MOL007151 | Tanshindiol B | PTGS2 COX2 |
| MOL007152 | Przewaquinone E | ACHE |
| MOL007152 | Przewaquinone E | CA2 |
| MOL007152 | Przewaquinone E | DPP4 |
| MOL007152 | Przewaquinone E | HSP90 |
| MOL007152 | Przewaquinone E | NCOA1 |
| MOL007152 | Przewaquinone E | PTGS2 COX2 |
| MOL007154 | tanshinone iia | ACHE |
| MOL007154 | tanshinone iia | AHSA1 |
| MOL007154 | tanshinone iia | ADRA1A |
| MOL007154 | tanshinone iia | BCL2 |
| MOL007154 | tanshinone iia | ADRB2 |
| MOL007154 | tanshinone iia | CALCR |
| MOL007154 | tanshinone iia | CASP3 |
| MOL007154 | tanshinone iia | TP53 |
| MOL007154 | tanshinone iia | CDKN1A CAP20 CDKN1 CIP1 MDA6 PIC1 SDI1 WAF1 |
| MOL007154 | tanshinone iia | CYP1A1 |
| MOL007154 | tanshinone iia | CYP1A2 |
| MOL007154 | tanshinone iia | CYP3A4 |
| MOL007154 | tanshinone iia | OPRD1 OPRD |
| MOL007154 | tanshinone iia | DPP4 |
| MOL007154 | tanshinone iia | DRD1 |
| MOL007154 | tanshinone iia | EDN1 |
| MOL007154 | tanshinone iia | EDN1R |
| MOL007154 | tanshinone iia | ECE1 |
| MOL007154 | tanshinone iia | FASN |
| MOL007154 | tanshinone iia | ITGB3 |
| MOL007154 | tanshinone iia | MMP9 |
| MOL007154 | tanshinone iia | CHRM1 |
| MOL007154 | tanshinone iia | CHRM2 |
| MOL007154 | tanshinone iia | CHRM3 |
| MOL007154 | tanshinone iia | CHRM4 |
| MOL007154 | tanshinone iia | CHRM5 |
| MOL007154 | tanshinone iia | OPRM1 |
| MOL007154 | tanshinone iia | MYC |
| MOL007154 | tanshinone iia | CHRNA7 |
| MOL007154 | tanshinone iia | NFKBIA |
| MOL007154 | tanshinone iia | NCOA1 |
| MOL007154 | tanshinone iia | NR1I2 hCG_21777 |
| MOL007154 | tanshinone iia | NPM1 |
| MOL007154 | tanshinone iia | PARP4 ADPRTL1 KIAA0177 PARPL |
| MOL007154 | tanshinone iia | PTGS2 COX2 |
| MOL007154 | tanshinone iia | FOS |
| MOL007154 | tanshinone iia | RXRA |
| MOL007154 | tanshinone iia | SCN5A |
| MOL007154 | tanshinone iia | JUN |
| MOL007154 | tanshinone iia | RELA |
| MOL007155 | (6S)-6-(hydroxymethyl)-1,6-dimethyl-8,9-dihydro-7H-naphtho[8,7-g]benzofuran-10,11-dione | ACHE |
| MOL007155 | (6S)-6-(hydroxymethyl)-1,6-dimethyl-8,9-dihydro-7H-naphtho[8,7-g]benzofuran-10,11-dione | ADRA1A |
| MOL007155 | (6S)-6-(hydroxymethyl)-1,6-dimethyl-8,9-dihydro-7H-naphtho[8,7-g]benzofuran-10,11-dione | ADRB2 |
| MOL007155 | (6S)-6-(hydroxymethyl)-1,6-dimethyl-8,9-dihydro-7H-naphtho[8,7-g]benzofuran-10,11-dione | OPRD1 OPRD |
| MOL007155 | (6S)-6-(hydroxymethyl)-1,6-dimethyl-8,9-dihydro-7H-naphtho[8,7-g]benzofuran-10,11-dione | DPP4 |
| MOL007155 | (6S)-6-(hydroxymethyl)-1,6-dimethyl-8,9-dihydro-7H-naphtho[8,7-g]benzofuran-10,11-dione | CHRM1 |
| MOL007155 | (6S)-6-(hydroxymethyl)-1,6-dimethyl-8,9-dihydro-7H-naphtho[8,7-g]benzofuran-10,11-dione | OPRM1 |
| MOL007155 | (6S)-6-(hydroxymethyl)-1,6-dimethyl-8,9-dihydro-7H-naphtho[8,7-g]benzofuran-10,11-dione | CHRNA7 |
| MOL007155 | (6S)-6-(hydroxymethyl)-1,6-dimethyl-8,9-dihydro-7H-naphtho[8,7-g]benzofuran-10,11-dione | NCOA1 |
| MOL007155 | (6S)-6-(hydroxymethyl)-1,6-dimethyl-8,9-dihydro-7H-naphtho[8,7-g]benzofuran-10,11-dione | PTGS2 COX2 |
| MOL007155 | (6S)-6-(hydroxymethyl)-1,6-dimethyl-8,9-dihydro-7H-naphtho[8,7-g]benzofuran-10,11-dione | SCN5A |
| MOL007155 | (6S)-6-(hydroxymethyl)-1,6-dimethyl-8,9-dihydro-7H-naphtho[8,7-g]benzofuran-10,11-dione | PRSS1 |
| MOL007156 | tanshinone Ⅵ | AR |
| MOL007156 | tanshinone Ⅵ | BQ8482_111389 |
| MOL007156 | tanshinone Ⅵ | CALM |
| MOL007156 | tanshinone Ⅵ | F10 |
| MOL007156 | tanshinone Ⅵ | ESR1 |
| MOL007156 | tanshinone Ⅵ | HSP90 |
| MOL007156 | tanshinone Ⅵ | NCOA1 |
| MOL007156 | tanshinone Ⅵ | NCOA2 |
| MOL007156 | tanshinone Ⅵ | ppar gamma2 |
| MOL007156 | tanshinone Ⅵ | PTGS1 COX1 |
| MOL007156 | tanshinone Ⅵ | PTGS2 COX2 |
| MOL007156 | tanshinone Ⅵ | SCN5A |

Table s2 The information of the genes related to Radiation Pneumonia

| Gene Symbol | Description | Category |  | Giftsc | | GC Id | Relevance score | GeneCards Link |
| --- | --- | --- | --- | --- | --- | --- | --- | --- |
| IL6 | Interleukin 6 | Protein Coding |  | 55 | GC07P022765 | | 49.28 | https://www.genecards.org/cgi-bin/carddisp.pl?gene=IL6 |
| TP53 | Tumor Protein P53 | Protein Coding |  | 60 | GC17M007661 | | 43.53 | https://www.genecards.org/cgi-bin/carddisp.pl?gene=TP53 |
| IFNG | Interferon Gamma | Protein Coding |  | 54 | GC12M068064 | | 37.35 | https://www.genecards.org/cgi-bin/carddisp.pl?gene=IFNG |
| TGFB1 | Transforming Growth Factor Beta 1 | Protein Coding |  | 59 | GC19M041301 | | 35.54 | https://www.genecards.org/cgi-bin/carddisp.pl?gene=TGFB1 |
| EGFR | Epidermal Growth Factor Receptor | Protein Coding |  | 62 | GC07P055019 | | 35.07 | https://www.genecards.org/cgi-bin/carddisp.pl?gene=EGFR |
| ATM | ATM Serine/Threonine Kinase | Protein Coding |  | 60 | GC11P108127 | | 33.08 | https://www.genecards.org/cgi-bin/carddisp.pl?gene=ATM |
| LIG4 | DNA Ligase 4 | Protein Coding |  | 54 | GC13M108207 | | 32.48 | https://www.genecards.org/cgi-bin/carddisp.pl?gene=LIG4 |
| TNF | Tumor Necrosis Factor | Protein Coding |  | 59 | GC06P032499 | | 32.11 | https://www.genecards.org/cgi-bin/carddisp.pl?gene=TNF |
| TLR2 | Toll Like Receptor 2 | Protein Coding |  | 56 | GC04P153684 | | 30.82 | https://www.genecards.org/cgi-bin/carddisp.pl?gene=TLR2 |
| ELANE | Elastase, Neutrophil Expressed | Protein Coding |  | 54 | GC19P000854 | | 30.11 | https://www.genecards.org/cgi-bin/carddisp.pl?gene=ELANE |
| SFTPC | Surfactant Protein C | Protein Coding |  | 48 | GC08P022156 | | 29.58 | https://www.genecards.org/cgi-bin/carddisp.pl?gene=SFTPC |
| DCLRE1C | DNA Cross-Link Repair 1C | Protein Coding |  | 50 | GC10M014897 | | 29.36 | https://www.genecards.org/cgi-bin/carddisp.pl?gene=DCLRE1C |
| TERT | Telomerase Reverse Transcriptase | Protein Coding |  | 57 | GC05M001253 | | 29.22 | https://www.genecards.org/cgi-bin/carddisp.pl?gene=TERT |
| AKT1 | AKT Serine/Threonine Kinase 1 | Protein Coding |  | 62 | GC14M104769 | | 29.03 | https://www.genecards.org/cgi-bin/carddisp.pl?gene=AKT1 |
| CD40LG | CD40 Ligand | Protein Coding |  | 54 | GC0XP136649 | | 27.85 | https://www.genecards.org/cgi-bin/carddisp.pl?gene=CD40LG |
| CDH1 | Cadherin 1 | Protein Coding |  | 57 | GC16P068737 | | 27.33 | https://www.genecards.org/cgi-bin/carddisp.pl?gene=CDH1 |
| STAT3 | Signal Transducer And Activator Of Transcription 3 | Protein Coding |  | 59 | GC17M042313 | | 27.29 | https://www.genecards.org/cgi-bin/carddisp.pl?gene=STAT3 |
| BRCA1 | BRCA1 DNA Repair Associated | Protein Coding |  | 57 | GC17M043044 | | 27.16 | https://www.genecards.org/cgi-bin/carddisp.pl?gene=BRCA1 |
| CXCL8 | C-X-C Motif Chemokine Ligand 8 | Protein Coding |  | 46 | GC04P073740 | | 26.92 | https://www.genecards.org/cgi-bin/carddisp.pl?gene=CXCL8 |
| PTEN | Phosphatase And Tensin Homolog | Protein Coding |  | 59 | GC10P087863 | | 24.24 | https://www.genecards.org/cgi-bin/carddisp.pl?gene=PTEN |
| CDKN2A | Cyclin Dependent Kinase Inhibitor 2A | Protein Coding |  | 57 | GC09M021957 | | 24.22 | https://www.genecards.org/cgi-bin/carddisp.pl?gene=CDKN2A |
| NBN | Nibrin | Protein Coding |  | 54 | GC08M089933 | | 24.21 | https://www.genecards.org/cgi-bin/carddisp.pl?gene=NBN |
| CRP | C-Reactive Protein | Protein Coding |  | 53 | GC01M159682 | | 24.11 | https://www.genecards.org/cgi-bin/carddisp.pl?gene=CRP |
| NSMCE3 | NSE3 Homolog, SMC5-SMC6 Complex Component | Protein Coding |  | 35 | GC15M029269 | | 23.32 | https://www.genecards.org/cgi-bin/carddisp.pl?gene=NSMCE3 |
| MUC5B | Mucin 5B, Oligomeric Mucus/Gel-Forming | Protein Coding |  | 47 | GC11P001244 | | 23.11 | https://www.genecards.org/cgi-bin/carddisp.pl?gene=MUC5B |
| TTR | Transthyretin | Protein Coding |  | 56 | GC18P031591 | | 23.03 | https://www.genecards.org/cgi-bin/carddisp.pl?gene=TTR |
| KRAS | KRAS Proto-Oncogene, GTPase | Protein Coding |  | 57 | GC12M025204 | | 22.91 | https://www.genecards.org/cgi-bin/carddisp.pl?gene=KRAS |
| FASLG | Fas Ligand | Protein Coding |  | 54 | GC01P172628 | | 22.57 | https://www.genecards.org/cgi-bin/carddisp.pl?gene=FASLG |
| CSF3 | Colony Stimulating Factor 3 | Protein Coding |  | 46 | GC17P040015 | | 22.17 | https://www.genecards.org/cgi-bin/carddisp.pl?gene=CSF3 |
| IL10 | Interleukin 10 | Protein Coding |  | 53 | GC01M206767 | | 22.15 | https://www.genecards.org/cgi-bin/carddisp.pl?gene=IL10 |
| PDGFB | Platelet Derived Growth Factor Subunit B | Protein Coding |  | 56 | GC22M043950 | | 21.97 | https://www.genecards.org/cgi-bin/carddisp.pl?gene=PDGFB |
| MMP9 | Matrix Metallopeptidase 9 | Protein Coding |  | 60 | GC20P046008 | | 21.8 | https://www.genecards.org/cgi-bin/carddisp.pl?gene=MMP9 |
| CCND1 | Cyclin D1 | Protein Coding |  | 59 | GC11P069641 | | 21.38 | https://www.genecards.org/cgi-bin/carddisp.pl?gene=CCND1 |
| ALB | Albumin | Protein Coding |  | 57 | GC04P073397 | | 21.17 | https://www.genecards.org/cgi-bin/carddisp.pl?gene=ALB |
| BAX | BCL2 Associated X, Apoptosis Regulator | Protein Coding |  | 54 | GC19P048954 | | 20.99 | https://www.genecards.org/cgi-bin/carddisp.pl?gene=BAX |
| MUC1 | Mucin 1, Cell Surface Associated | Protein Coding |  | 54 | GC01M155158 | | 20.89 | https://www.genecards.org/cgi-bin/carddisp.pl?gene=MUC1 |
| VEGFA | Vascular Endothelial Growth Factor A | Protein Coding |  | 56 | GC06P043770 | | 20.82 | https://www.genecards.org/cgi-bin/carddisp.pl?gene=VEGFA |
| HRAS | HRas Proto-Oncogene, GTPase | Protein Coding |  | 59 | GC11M000522 | | 20.67 | https://www.genecards.org/cgi-bin/carddisp.pl?gene=HRAS |
| SFTPD | Surfactant Protein D | Protein Coding |  | 50 | GC10M079937 | | 20.66 | https://www.genecards.org/cgi-bin/carddisp.pl?gene=SFTPD |
| SRC | SRC Proto-Oncogene, Non-Receptor Tyrosine Kinase | Protein Coding |  | 59 | GC20P037344 | | 20.55 | https://www.genecards.org/cgi-bin/carddisp.pl?gene=SRC |
| FOXP3 | Forkhead Box P3 | Protein Coding |  | 53 | GC0XM049250 | | 20.52 | https://www.genecards.org/cgi-bin/carddisp.pl?gene=FOXP3 |
| NHEJ1 | Non-Homologous End Joining Factor 1 | Protein Coding |  | 48 | GC02M219075 | | 20.48 | https://www.genecards.org/cgi-bin/carddisp.pl?gene=NHEJ1 |
| ERBB2 | Erb-B2 Receptor Tyrosine Kinase 2 | Protein Coding |  | 62 | GC17P039687 | | 20.42 | https://www.genecards.org/cgi-bin/carddisp.pl?gene=ERBB2 |
| PIK3CA | Phosphatidylinositol-4,5-Bisphosphate 3-Kinase Catalytic Subunit Alpha | Protein Coding |  | 59 | GC03P179148 | | 20.26 | https://www.genecards.org/cgi-bin/carddisp.pl?gene=PIK3CA |
| PTPRC | Protein Tyrosine Phosphatase Receptor Type C | Protein Coding |  | 57 | GC01P198607 | | 20.23 | https://www.genecards.org/cgi-bin/carddisp.pl?gene=PTPRC |
| IL1B | Interleukin 1 Beta | Protein Coding |  | 54 | GC02M112829 | | 19.92 | https://www.genecards.org/cgi-bin/carddisp.pl?gene=IL1B |
| CASP8 | Caspase 8 | Protein Coding |  | 59 | GC02P201233 | | 19.86 | https://www.genecards.org/cgi-bin/carddisp.pl?gene=CASP8 |
| MIR21 | MicroRNA 21 | RNA Gene |  | 24 | GC17P059841 | | 19.23 | https://www.genecards.org/cgi-bin/carddisp.pl?gene=MIR21 |
| TLR4 | Toll Like Receptor 4 | Protein Coding |  | 57 | GC09P117704 | | 19.19 | https://www.genecards.org/cgi-bin/carddisp.pl?gene=TLR4 |
| KIT | KIT Proto-Oncogene, Receptor Tyrosine Kinase | Protein Coding |  | 59 | GC04P054657 | | 18.98 | https://www.genecards.org/cgi-bin/carddisp.pl?gene=KIT |
| BRAF | B-Raf Proto-Oncogene, Serine/Threonine Kinase | Protein Coding |  | 61 | GC07M140719 | | 18.96 | https://www.genecards.org/cgi-bin/carddisp.pl?gene=BRAF |
| CDK4 | Cyclin Dependent Kinase 4 | Protein Coding |  | 62 | GC12M057743 | | 18.88 | https://www.genecards.org/cgi-bin/carddisp.pl?gene=CDK4 |
| CTNNB1 | Catenin Beta 1 | Protein Coding |  | 60 | GC03P041236 | | 18.68 | https://www.genecards.org/cgi-bin/carddisp.pl?gene=CTNNB1 |
| CHEK2 | Checkpoint Kinase 2 | Protein Coding |  | 60 | GC22M028687 | | 18.67 | https://www.genecards.org/cgi-bin/carddisp.pl?gene=CHEK2 |
| MTOR | Mechanistic Target Of Rapamycin Kinase | Protein Coding |  | 61 | GC01M011166 | | 18.57 | https://www.genecards.org/cgi-bin/carddisp.pl?gene=MTOR |
| SFTPA1 | Surfactant Protein A1 | Protein Coding |  | 48 | GC10P082997 | | 18.56 | https://www.genecards.org/cgi-bin/carddisp.pl?gene=SFTPA1 |
| NRAS | NRAS Proto-Oncogene, GTPase | Protein Coding |  | 57 | GC01M114704 | | 18.33 | https://www.genecards.org/cgi-bin/carddisp.pl?gene=NRAS |
| CCL2 | C-C Motif Chemokine Ligand 2 | Protein Coding |  | 55 | GC17P034255 | | 18.11 | https://www.genecards.org/cgi-bin/carddisp.pl?gene=CCL2 |
| RNF168 | Ring Finger Protein 168 | Protein Coding |  | 50 | GC03M196468 | | 18.02 | https://www.genecards.org/cgi-bin/carddisp.pl?gene=RNF168 |
| FGF2 | Fibroblast Growth Factor 2 | Protein Coding |  | 54 | GC04P122826 | | 17.67 | https://www.genecards.org/cgi-bin/carddisp.pl?gene=FGF2 |
| CASP3 | Caspase 3 | Protein Coding |  | 57 | GC04M184627 | | 17.61 | https://www.genecards.org/cgi-bin/carddisp.pl?gene=CASP3 |
| MRE11 | MRE11 Homolog, Double Strand Break Repair Nuclease | Protein Coding |  | 46 | GC11M094529 | | 17.55 | https://www.genecards.org/cgi-bin/carddisp.pl?gene=MRE11 |
| TERC | Telomerase RNA Component | RNA Gene |  | 31 | GC03M169765 | | 17.49 | https://www.genecards.org/cgi-bin/carddisp.pl?gene=TERC |
| IL13 | Interleukin 13 | Protein Coding |  | 50 | GC05P132656 | | 17.42 | https://www.genecards.org/cgi-bin/carddisp.pl?gene=IL13 |
| SFTPA2 | Surfactant Protein A2 | Protein Coding |  | 46 | GC10M079563 | | 17.04 | https://www.genecards.org/cgi-bin/carddisp.pl?gene=SFTPA2 |
| IL4 | Interleukin 4 | Protein Coding |  | 53 | GC05P132673 | | 17.03 | https://www.genecards.org/cgi-bin/carddisp.pl?gene=IL4 |
| IL17A | Interleukin 17A | Protein Coding |  | 49 | GC06P052186 | | 16.99 | https://www.genecards.org/cgi-bin/carddisp.pl?gene=IL17A |
| ICAM1 | Intercellular Adhesion Molecule 1 | Protein Coding |  | 57 | GC19P010270 | | 16.62 | https://www.genecards.org/cgi-bin/carddisp.pl?gene=ICAM1 |
| MPO | Myeloperoxidase | Protein Coding |  | 57 | GC17M058269 | | 16.59 | https://www.genecards.org/cgi-bin/carddisp.pl?gene=MPO |
| LACTB | Lactamase Beta | Protein Coding |  | 42 | GC15P069629 | | 16.52 | https://www.genecards.org/cgi-bin/carddisp.pl?gene=LACTB |
| MET | MET Proto-Oncogene, Receptor Tyrosine Kinase | Protein Coding |  | 62 | GC07P116672 | | 16.49 | https://www.genecards.org/cgi-bin/carddisp.pl?gene=MET |
| CCL3 | C-C Motif Chemokine Ligand 3 | Protein Coding |  | 45 | GC17M036088 | | 16.29 | https://www.genecards.org/cgi-bin/carddisp.pl?gene=CCL3 |
| CD19 | CD19 Molecule | Protein Coding |  | 56 | GC16P028943 | | 16.27 | https://www.genecards.org/cgi-bin/carddisp.pl?gene=CD19 |
| RAD50 | RAD50 Double Strand Break Repair Protein | Protein Coding |  | 56 | GC05P132556 | | 16.22 | https://www.genecards.org/cgi-bin/carddisp.pl?gene=RAD50 |
| PPARG | Peroxisome Proliferator Activated Receptor Gamma | Protein Coding |  | 59 | GC03P012328 | | 16.11 | https://www.genecards.org/cgi-bin/carddisp.pl?gene=PPARG |
| STAT1 | Signal Transducer And Activator Of Transcription 1 | Protein Coding |  | 59 | GC02M190964 | | 16.09 | https://www.genecards.org/cgi-bin/carddisp.pl?gene=STAT1 |
| CDKN1A | Cyclin Dependent Kinase Inhibitor 1A | Protein Coding |  | 57 | GC06P043169 | | 16.02 | https://www.genecards.org/cgi-bin/carddisp.pl?gene=CDKN1A |
| NKX2-1 | NK2 Homeobox 1 | Protein Coding |  | 51 | GC14M036516 | | 15.95 | https://www.genecards.org/cgi-bin/carddisp.pl?gene=NKX2-1 |
| CCL5 | C-C Motif Chemokine Ligand 5 | Protein Coding |  | 50 | GC17M035871 | | 15.94 | https://www.genecards.org/cgi-bin/carddisp.pl?gene=CCL5 |
| CSF2 | Colony Stimulating Factor 2 | Protein Coding |  | 50 | GC05P132073 | | 15.81 | https://www.genecards.org/cgi-bin/carddisp.pl?gene=CSF2 |
| MIR221 | MicroRNA 221 | RNA Gene |  | 21 | GC0XM045746 | | 15.67 | https://www.genecards.org/cgi-bin/carddisp.pl?gene=MIR221 |
| IL2 | Interleukin 2 | Protein Coding |  | 52 | GC04M122451 | | 15.49 | https://www.genecards.org/cgi-bin/carddisp.pl?gene=IL2 |
| TIMP1 | TIMP Metallopeptidase Inhibitor 1 | Protein Coding |  | 51 | GC0XP047583 | | 15.45 | https://www.genecards.org/cgi-bin/carddisp.pl?gene=TIMP1 |
| BCL2 | BCL2 Apoptosis Regulator | Protein Coding |  | 59 | GC18M063123 | | 15.36 | https://www.genecards.org/cgi-bin/carddisp.pl?gene=BCL2 |
| MAPK8 | Mitogen-Activated Protein Kinase 8 | Protein Coding |  | 56 | GC10P048306 | | 15.35 | https://www.genecards.org/cgi-bin/carddisp.pl?gene=MAPK8 |
| TP63 | Tumor Protein P63 | Protein Coding |  | 53 | GC03P189566 | | 15.3 | https://www.genecards.org/cgi-bin/carddisp.pl?gene=TP63 |
| RAF1 | Raf-1 Proto-Oncogene, Serine/Threonine Kinase | Protein Coding |  | 62 | GC03M012583 | | 15.28 | https://www.genecards.org/cgi-bin/carddisp.pl?gene=RAF1 |
| IDH1 | Isocitrate Dehydrogenase (NADP(+)) 1, Cytosolic | Protein Coding |  | 59 | GC02M208236 | | 15.28 | https://www.genecards.org/cgi-bin/carddisp.pl?gene=IDH1 |
| RTEL1 | Regulator Of Telomere Elongation Helicase 1 | Protein Coding |  | 46 | GC20P063658 | | 15.27 | https://www.genecards.org/cgi-bin/carddisp.pl?gene=RTEL1 |
| MYD88 | MYD88 Innate Immune Signal Transduction Adaptor | Protein Coding |  | 56 | GC03P038179 | | 15.25 | https://www.genecards.org/cgi-bin/carddisp.pl?gene=MYD88 |
| ABCA3 | ATP Binding Cassette Subfamily A Member 3 | Protein Coding |  | 54 | GC16M002275 | | 15.19 | https://www.genecards.org/cgi-bin/carddisp.pl?gene=ABCA3 |
| JUN | Jun Proto-Oncogene, AP-1 Transcription Factor Subunit | Protein Coding |  | 56 | GC01M058780 | | 15.14 | https://www.genecards.org/cgi-bin/carddisp.pl?gene=JUN |
| MIR155 | MicroRNA 155 | RNA Gene |  | 18 | GC21P025573 | | 15.07 | https://www.genecards.org/cgi-bin/carddisp.pl?gene=MIR155 |
| MIR34A | MicroRNA 34a | RNA Gene |  | 23 | GC01M009151 | | 15.06 | https://www.genecards.org/cgi-bin/carddisp.pl?gene=MIR34A |
| RAG1 | Recombination Activating 1 | Protein Coding |  | 52 | GC11P036546 | | 15.05 | https://www.genecards.org/cgi-bin/carddisp.pl?gene=RAG1 |
| MAPK1 | Mitogen-Activated Protein Kinase 1 | Protein Coding |  | 58 | GC22M021754 | | 14.92 | https://www.genecards.org/cgi-bin/carddisp.pl?gene=MAPK1 |
| SFTPB | Surfactant Protein B | Protein Coding |  | 50 | GC02M085657 | | 14.86 | https://www.genecards.org/cgi-bin/carddisp.pl?gene=SFTPB |
| PTGS2 | Prostaglandin-Endoperoxide Synthase 2 | Protein Coding |  | 56 | GC01M186640 | | 14.76 | https://www.genecards.org/cgi-bin/carddisp.pl?gene=PTGS2 |
| CXCL1 | C-X-C Motif Chemokine Ligand 1 | Protein Coding |  | 49 | GC04P073869 | | 14.69 | https://www.genecards.org/cgi-bin/carddisp.pl?gene=CXCL1 |
| MAP2K1 | Mitogen-Activated Protein Kinase Kinase 1 | Protein Coding |  | 61 | GC15P066386 | | 14.62 | https://www.genecards.org/cgi-bin/carddisp.pl?gene=MAP2K1 |
| IDH2 | Isocitrate Dehydrogenase (NADP(+)) 2, Mitochondrial | Protein Coding |  | 58 | GC15M090083 | | 14.46 | https://www.genecards.org/cgi-bin/carddisp.pl?gene=IDH2 |
| CR2 | Complement C3d Receptor 2 | Protein Coding |  | 51 | GC01P207454 | | 14.16 | https://www.genecards.org/cgi-bin/carddisp.pl?gene=CR2 |
| CXCR3 | C-X-C Motif Chemokine Receptor 3 | Protein Coding |  | 50 | GC0XM071615 | | 14.16 | https://www.genecards.org/cgi-bin/carddisp.pl?gene=CXCR3 |
| RB1 | RB Transcriptional Corepressor 1 | Protein Coding |  | 56 | GC13P048303 | | 14.11 | https://www.genecards.org/cgi-bin/carddisp.pl?gene=RB1 |
| PLG | Plasminogen | Protein Coding |  | 54 | GC06P160702 | | 14.06 | https://www.genecards.org/cgi-bin/carddisp.pl?gene=PLG |
| MDM2 | MDM2 Proto-Oncogene | Protein Coding |  | 59 | GC12P068808 | | 14.05 | https://www.genecards.org/cgi-bin/carddisp.pl?gene=MDM2 |
| FGFR3 | Fibroblast Growth Factor Receptor 3 | Protein Coding |  | 62 | GC04P001795 | | 14.03 | https://www.genecards.org/cgi-bin/carddisp.pl?gene=FGFR3 |
| NFKB1 | Nuclear Factor Kappa B Subunit 1 | Protein Coding |  | 59 | GC04P102501 | | 13.98 | https://www.genecards.org/cgi-bin/carddisp.pl?gene=NFKB1 |
| PTPN11 | Protein Tyrosine Phosphatase Non-Receptor Type 11 | Protein Coding |  | 58 | GC12P112418 | | 13.98 | https://www.genecards.org/cgi-bin/carddisp.pl?gene=PTPN11 |
| MIR222 | MicroRNA 222 | RNA Gene |  | 21 | GC0XM045747 | | 13.81 | https://www.genecards.org/cgi-bin/carddisp.pl?gene=MIR222 |
| ERCC1 | ERCC Excision Repair 1, Endonuclease Non-Catalytic Subunit | Protein Coding |  | 52 | GC19M045409 | | 13.77 | https://www.genecards.org/cgi-bin/carddisp.pl?gene=ERCC1 |
| CHAT | Choline O-Acetyltransferase | Protein Coding |  | 54 | GC10P049609 | | 13.73 | https://www.genecards.org/cgi-bin/carddisp.pl?gene=CHAT |
| FN1 | Fibronectin 1 | Protein Coding |  | 56 | GC02M215360 | | 13.68 | https://www.genecards.org/cgi-bin/carddisp.pl?gene=FN1 |
| CXCR4 | C-X-C Motif Chemokine Receptor 4 | Protein Coding |  | 59 | GC02M136114 | | 13.65 | https://www.genecards.org/cgi-bin/carddisp.pl?gene=CXCR4 |
| STK11 | Serine/Threonine Kinase 11 | Protein Coding |  | 55 | GC19P001205 | | 13.64 | https://www.genecards.org/cgi-bin/carddisp.pl?gene=STK11 |
| MIRLET7B | MicroRNA Let-7b | RNA Gene |  | 21 | GC22P046123 | | 13.62 | https://www.genecards.org/cgi-bin/carddisp.pl?gene=MIRLET7B |
| SMAD4 | SMAD Family Member 4 | Protein Coding |  | 57 | GC18P051028 | | 13.62 | https://www.genecards.org/cgi-bin/carddisp.pl?gene=SMAD4 |
| HIF1A | Hypoxia Inducible Factor 1 Subunit Alpha | Protein Coding |  | 54 | GC14P061695 | | 13.51 | https://www.genecards.org/cgi-bin/carddisp.pl?gene=HIF1A |
| SCGB1A1 | Secretoglobin Family 1A Member 1 | Protein Coding |  | 46 | GC11P062437 | | 13.44 | https://www.genecards.org/cgi-bin/carddisp.pl?gene=SCGB1A1 |
| IL3 | Interleukin 3 | Protein Coding |  | 49 | GC05P132060 | | 13.38 | https://www.genecards.org/cgi-bin/carddisp.pl?gene=IL3 |
| PIK3R1 | Phosphoinositide-3-Kinase Regulatory Subunit 1 | Protein Coding |  | 58 | GC05P068215 | | 13.3 | https://www.genecards.org/cgi-bin/carddisp.pl?gene=PIK3R1 |
| FAS | Fas Cell Surface Death Receptor | Protein Coding |  | 56 | GC10P088969 | | 13.25 | https://www.genecards.org/cgi-bin/carddisp.pl?gene=FAS |
| TNFRSF10B | TNF Receptor Superfamily Member 10b | Protein Coding |  | 57 | GC08M023020 | | 13.23 | https://www.genecards.org/cgi-bin/carddisp.pl?gene=TNFRSF10B |
| MIR181A2 | MicroRNA 181a-2 | RNA Gene |  | 20 | GC09P124692 | | 13.2 | https://www.genecards.org/cgi-bin/carddisp.pl?gene=MIR181A2 |
| MIR15A | MicroRNA 15a | RNA Gene |  | 16 | GC13M050049 | | 13.14 | https://www.genecards.org/cgi-bin/carddisp.pl?gene=MIR15A |
| HGF | Hepatocyte Growth Factor | Protein Coding |  | 58 | GC07M081699 | | 13.12 | https://www.genecards.org/cgi-bin/carddisp.pl?gene=HGF |
| MIR20A | MicroRNA 20a | RNA Gene |  | 19 | GC13P091412 | | 13.11 | https://www.genecards.org/cgi-bin/carddisp.pl?gene=MIR20A |
| CXCL10 | C-X-C Motif Chemokine Ligand 10 | Protein Coding |  | 50 | GC04M076021 | | 13.07 | https://www.genecards.org/cgi-bin/carddisp.pl?gene=CXCL10 |
| MIR146A | MicroRNA 146a | RNA Gene |  | 23 | GC05P160485 | | 12.94 | https://www.genecards.org/cgi-bin/carddisp.pl?gene=MIR146A |
| PRKCD | Protein Kinase C Delta | Protein Coding |  | 60 | GC03P053190 | | 12.88 | https://www.genecards.org/cgi-bin/carddisp.pl?gene=PRKCD |
| DNMT3B | DNA Methyltransferase 3 Beta | Protein Coding |  | 56 | GC20P032762 | | 12.87 | https://www.genecards.org/cgi-bin/carddisp.pl?gene=DNMT3B |
| IL1A | Interleukin 1 Alpha | Protein Coding |  | 50 | GC02M112773 | | 12.82 | https://www.genecards.org/cgi-bin/carddisp.pl?gene=IL1A |
| MIR19A | MicroRNA 19a | RNA Gene |  | 19 | GC13P091411 | | 12.82 | https://www.genecards.org/cgi-bin/carddisp.pl?gene=MIR19A |
| MIR29C | MicroRNA 29c | RNA Gene |  | 18 | GC01M207802 | | 12.79 | https://www.genecards.org/cgi-bin/carddisp.pl?gene=MIR29C |
| MMP1 | Matrix Metallopeptidase 1 | Protein Coding |  | 58 | GC11M102810 | | 12.78 | https://www.genecards.org/cgi-bin/carddisp.pl?gene=MMP1 |
| MIR203A | MicroRNA 203a | RNA Gene |  | 21 | GC14P104119 | | 12.7 | https://www.genecards.org/cgi-bin/carddisp.pl?gene=MIR203A |
| EGF | Epidermal Growth Factor | Protein Coding |  | 57 | GC04P109912 | | 12.68 | https://www.genecards.org/cgi-bin/carddisp.pl?gene=EGF |
| TGFBR2 | Transforming Growth Factor Beta Receptor 2 | Protein Coding |  | 58 | GC03P030623 | | 12.64 | https://www.genecards.org/cgi-bin/carddisp.pl?gene=TGFBR2 |
| CXCL12 | C-X-C Motif Chemokine Ligand 12 | Protein Coding |  | 51 | GC10M044334 | | 12.62 | https://www.genecards.org/cgi-bin/carddisp.pl?gene=CXCL12 |
| MIR125A | MicroRNA 125a | RNA Gene |  | 21 | GC19P051733 | | 12.6 | https://www.genecards.org/cgi-bin/carddisp.pl?gene=MIR125A |
| MIR210 | MicroRNA 210 | RNA Gene |  | 21 | GC11M000610 | | 12.58 | https://www.genecards.org/cgi-bin/carddisp.pl?gene=MIR210 |
| IRF1 | Interferon Regulatory Factor 1 | Protein Coding |  | 54 | GC05M132481 | | 12.55 | https://www.genecards.org/cgi-bin/carddisp.pl?gene=IRF1 |
| DSP | Desmoplakin | Protein Coding |  | 56 | GC06P007541 | | 12.53 | https://www.genecards.org/cgi-bin/carddisp.pl?gene=DSP |
| MIRLET7E | MicroRNA Let-7e | RNA Gene |  | 21 | GC19P051732 | | 12.52 | https://www.genecards.org/cgi-bin/carddisp.pl?gene=MIRLET7E |
| MIR205 | MicroRNA 205 | RNA Gene |  | 20 | GC01P209432 | | 12.44 | https://www.genecards.org/cgi-bin/carddisp.pl?gene=MIR205 |
| XRCC1 | X-Ray Repair Cross Complementing 1 | Protein Coding |  | 49 | GC19M043543 | | 12.29 | https://www.genecards.org/cgi-bin/carddisp.pl?gene=XRCC1 |
| PARN | Poly(A)-Specific Ribonuclease | Protein Coding |  | 51 | GC16M014435 | | 12.28 | https://www.genecards.org/cgi-bin/carddisp.pl?gene=PARN |
| GSTM1 | Glutathione S-Transferase Mu 1 | Protein Coding |  | 48 | GC01P109687 | | 12.27 | https://www.genecards.org/cgi-bin/carddisp.pl?gene=GSTM1 |
| PARP1 | Poly(ADP-Ribose) Polymerase 1 | Protein Coding |  | 56 | GC01M226360 | | 12.26 | https://www.genecards.org/cgi-bin/carddisp.pl?gene=PARP1 |
| ITGAM | Integrin Subunit Alpha M | Protein Coding |  | 53 | GC16P031326 | | 12.2 | https://www.genecards.org/cgi-bin/carddisp.pl?gene=ITGAM |
| HMGB1 | High Mobility Group Box 1 | Protein Coding |  | 50 | GC13M030456 | | 12.16 | https://www.genecards.org/cgi-bin/carddisp.pl?gene=HMGB1 |
| XRCC3 | X-Ray Repair Cross Complementing 3 | Protein Coding |  | 46 | GC14M103697 | | 12.16 | https://www.genecards.org/cgi-bin/carddisp.pl?gene=XRCC3 |
| MIR145 | MicroRNA 145 | RNA Gene |  | 23 | GC05P149430 | | 12.13 | https://www.genecards.org/cgi-bin/carddisp.pl?gene=MIR145 |
| MIR141 | MicroRNA 141 | RNA Gene |  | 22 | GC12P007639 | | 12.1 | https://www.genecards.org/cgi-bin/carddisp.pl?gene=MIR141 |
| MIR195 | MicroRNA 195 | RNA Gene |  | 20 | GC17M007018 | | 12.08 | https://www.genecards.org/cgi-bin/carddisp.pl?gene=MIR195 |
| BIRC5 | Baculoviral IAP Repeat Containing 5 | Protein Coding |  | 53 | GC17P078214 | | 12.06 | https://www.genecards.org/cgi-bin/carddisp.pl?gene=BIRC5 |
| MIR143 | MicroRNA 143 | RNA Gene |  | 23 | GC05P149410 | | 12.03 | https://www.genecards.org/cgi-bin/carddisp.pl?gene=MIR143 |
| NME1 | NME/NM23 Nucleoside Diphosphate Kinase 1 | Protein Coding |  | 54 | GC17P051154 | | 11.98 | https://www.genecards.org/cgi-bin/carddisp.pl?gene=NME1 |
| MIR93 | MicroRNA 93 | RNA Gene |  | 21 | GC07M100198 | | 11.96 | https://www.genecards.org/cgi-bin/carddisp.pl?gene=MIR93 |
| MIR191 | MicroRNA 191 | RNA Gene |  | 21 | GC03M049100 | | 11.94 | https://www.genecards.org/cgi-bin/carddisp.pl?gene=MIR191 |
| MMP2 | Matrix Metallopeptidase 2 | Protein Coding |  | 60 | GC16P055424 | | 11.92 | https://www.genecards.org/cgi-bin/carddisp.pl?gene=MMP2 |
| ERCC2 | ERCC Excision Repair 2, TFIIH Core Complex Helicase Subunit | Protein Coding |  | 53 | GC19M045349 | | 11.89 | https://www.genecards.org/cgi-bin/carddisp.pl?gene=ERCC2 |
| MIR100 | MicroRNA 100 | RNA Gene |  | 21 | GC11M122152 | | 11.88 | https://www.genecards.org/cgi-bin/carddisp.pl?gene=MIR100 |
| CYCS | Cytochrome C, Somatic | Protein Coding |  | 55 | GC07M025158 | | 11.87 | https://www.genecards.org/cgi-bin/carddisp.pl?gene=CYCS |
| MIR31 | MicroRNA 31 | RNA Gene |  | 20 | GC09M021507 | | 11.85 | https://www.genecards.org/cgi-bin/carddisp.pl?gene=MIR31 |
| NFKB2 | Nuclear Factor Kappa B Subunit 2 | Protein Coding |  | 59 | GC10P102394 | | 11.84 | https://www.genecards.org/cgi-bin/carddisp.pl?gene=NFKB2 |
| OGG1 | 8-Oxoguanine DNA Glycosylase | Protein Coding |  | 53 | GC03P009751 | | 11.83 | https://www.genecards.org/cgi-bin/carddisp.pl?gene=OGG1 |
| ANXA5 | Annexin A5 | Protein Coding |  | 52 | GC04M121667 | | 11.83 | https://www.genecards.org/cgi-bin/carddisp.pl?gene=ANXA5 |
| ALK | ALK Receptor Tyrosine Kinase | Protein Coding |  | 58 | GC02M029156 | | 11.81 | https://www.genecards.org/cgi-bin/carddisp.pl?gene=ALK |
| MAPK3 | Mitogen-Activated Protein Kinase 3 | Protein Coding |  | 56 | GC16M030125 | | 11.8 | https://www.genecards.org/cgi-bin/carddisp.pl?gene=MAPK3 |
| MYC | MYC Proto-Oncogene, BHLH Transcription Factor | Protein Coding |  | 59 | GC08P127735 | | 11.78 | https://www.genecards.org/cgi-bin/carddisp.pl?gene=MYC |
| STN1 | STN1 Subunit Of CST Complex | Protein Coding |  | 37 | GC10M103872 | | 11.68 | https://www.genecards.org/cgi-bin/carddisp.pl?gene=STN1 |
| ABCB1 | ATP Binding Cassette Subfamily B Member 1 | Protein Coding |  | 57 | GC07M087504 | | 11.66 | https://www.genecards.org/cgi-bin/carddisp.pl?gene=ABCB1 |
| MLH1 | MutL Homolog 1 | Protein Coding |  | 53 | GC03P036993 | | 11.66 | https://www.genecards.org/cgi-bin/carddisp.pl?gene=MLH1 |
| MIR106A | MicroRNA 106a | RNA Gene |  | 18 | GC0XM134233 | | 11.65 | https://www.genecards.org/cgi-bin/carddisp.pl?gene=MIR106A |
| MIR335 | MicroRNA 335 | RNA Gene |  | 18 | GC07P130496 | | 11.64 | https://www.genecards.org/cgi-bin/carddisp.pl?gene=MIR335 |
| KMT2A | Lysine Methyltransferase 2A | Protein Coding |  | 50 | GC11P118436 | | 11.6 | https://www.genecards.org/cgi-bin/carddisp.pl?gene=KMT2A |
| MIR214 | MicroRNA 214 | RNA Gene |  | 20 | GC01M172218 | | 11.57 | https://www.genecards.org/cgi-bin/carddisp.pl?gene=MIR214 |
| SPP1 | Secreted Phosphoprotein 1 | Protein Coding |  | 52 | GC04P087975 | | 11.53 | https://www.genecards.org/cgi-bin/carddisp.pl?gene=SPP1 |
| MIR15B | MicroRNA 15b | RNA Gene |  | 19 | GC03P160404 | | 11.52 | https://www.genecards.org/cgi-bin/carddisp.pl?gene=MIR15B |
| MIR24-2 | MicroRNA 24-2 | RNA Gene |  | 20 | GC19M013944 | | 11.51 | https://www.genecards.org/cgi-bin/carddisp.pl?gene=MIR24-2 |
| MIR25 | MicroRNA 25 | RNA Gene |  | 20 | GC07M100093 | | 11.49 | https://www.genecards.org/cgi-bin/carddisp.pl?gene=MIR25 |
| MIR126 | MicroRNA 126 | RNA Gene |  | 23 | GC09P136670 | | 11.49 | https://www.genecards.org/cgi-bin/carddisp.pl?gene=MIR126 |
| MIR182 | MicroRNA 182 | RNA Gene |  | 20 | GC07M129770 | | 11.45 | https://www.genecards.org/cgi-bin/carddisp.pl?gene=MIR182 |
| MMP7 | Matrix Metallopeptidase 7 | Protein Coding |  | 54 | GC11M102425 | | 11.39 | https://www.genecards.org/cgi-bin/carddisp.pl?gene=MMP7 |
| ABL1 | ABL Proto-Oncogene 1, Non-Receptor Tyrosine Kinase | Protein Coding |  | 59 | GC09P130713 | | 11.38 | https://www.genecards.org/cgi-bin/carddisp.pl?gene=ABL1 |
| FGFR1 | Fibroblast Growth Factor Receptor 1 | Protein Coding |  | 62 | GC08M038400 | | 11.38 | https://www.genecards.org/cgi-bin/carddisp.pl?gene=FGFR1 |
| TOP2A | DNA Topoisomerase II Alpha | Protein Coding |  | 57 | GC17M040388 | | 11.37 | https://www.genecards.org/cgi-bin/carddisp.pl?gene=TOP2A |
| FLT3 | Fms Related Tyrosine Kinase 3 | Protein Coding |  | 60 | GC13M028003 | | 11.29 | https://www.genecards.org/cgi-bin/carddisp.pl?gene=FLT3 |
| CASP9 | Caspase 9 | Protein Coding |  | 54 | GC01M015565 | | 11.28 | https://www.genecards.org/cgi-bin/carddisp.pl?gene=CASP9 |
| CD44 | CD44 Molecule (Indian Blood Group) | Protein Coding |  | 54 | GC11P035168 | | 11.28 | https://www.genecards.org/cgi-bin/carddisp.pl?gene=CD44 |
| ERCC6 | ERCC Excision Repair 6, Chromatin Remodeling Factor | Protein Coding |  | 51 | GC10M049454 | | 11.26 | https://www.genecards.org/cgi-bin/carddisp.pl?gene=ERCC6 |
| TLR5 | Toll Like Receptor 5 | Protein Coding |  | 54 | GC01M223109 | | 11.25 | https://www.genecards.org/cgi-bin/carddisp.pl?gene=TLR5 |
| MIR200A | MicroRNA 200a | RNA Gene |  | 22 | GC01P001206 | | 11.23 | https://www.genecards.org/cgi-bin/carddisp.pl?gene=MIR200A |
| FHIT | Fragile Histidine Triad Diadenosine Triphosphatase | Protein Coding |  | 51 | GC03M059712 | | 11.22 | https://www.genecards.org/cgi-bin/carddisp.pl?gene=FHIT |
| MIR34C | MicroRNA 34c | RNA Gene |  | 21 | GC11P111565 | | 11.2 | https://www.genecards.org/cgi-bin/carddisp.pl?gene=MIR34C |
| MIR22 | MicroRNA 22 | RNA Gene |  | 20 | GC17M001713 | | 11.2 | https://www.genecards.org/cgi-bin/carddisp.pl?gene=MIR22 |
| MIR32 | MicroRNA 32 | RNA Gene |  | 20 | GC09M109046 | | 11.2 | https://www.genecards.org/cgi-bin/carddisp.pl?gene=MIR32 |
| MIR451A | MicroRNA 451a | RNA Gene |  | 18 | GC17M028861 | | 11.18 | https://www.genecards.org/cgi-bin/carddisp.pl?gene=MIR451A |
| IGHM | Immunoglobulin Heavy Constant Mu | Protein Coding |  | 35 | GC14M105852 | | 11.18 | https://www.genecards.org/cgi-bin/carddisp.pl?gene=IGHM |
| TNFRSF13C | TNF Receptor Superfamily Member 13C | Protein Coding |  | 51 | GC22M042909 | | 11.14 | https://www.genecards.org/cgi-bin/carddisp.pl?gene=TNFRSF13C |
| MIR128-2 | MicroRNA 128-2 | RNA Gene |  | 21 | GC03P035753 | | 11.14 | https://www.genecards.org/cgi-bin/carddisp.pl?gene=MIR128-2 |
| IL1RN | Interleukin 1 Receptor Antagonist | Protein Coding |  | 54 | GC02P114223 | | 11.13 | https://www.genecards.org/cgi-bin/carddisp.pl?gene=IL1RN |
| GSTP1 | Glutathione S-Transferase Pi 1 | Protein Coding |  | 57 | GC11P067583 | | 11.11 | https://www.genecards.org/cgi-bin/carddisp.pl?gene=GSTP1 |
| SERPINE1 | Serpin Family E Member 1 | Protein Coding |  | 57 | GC07P101127 | | 11.09 | https://www.genecards.org/cgi-bin/carddisp.pl?gene=SERPINE1 |
| MIR107 | MicroRNA 107 | RNA Gene |  | 20 | GC10M089604 | | 11.08 | https://www.genecards.org/cgi-bin/carddisp.pl?gene=MIR107 |
| BCL2L1 | BCL2 Like 1 | Protein Coding |  | 54 | GC20M031664 | | 10.97 | https://www.genecards.org/cgi-bin/carddisp.pl?gene=BCL2L1 |
| CD79A | CD79a Molecule | Protein Coding |  | 53 | GC19P041877 | | 10.94 | https://www.genecards.org/cgi-bin/carddisp.pl?gene=CD79A |
| MIR18A | MicroRNA 18a | RNA Gene |  | 18 | GC13P091405 | | 10.93 | https://www.genecards.org/cgi-bin/carddisp.pl?gene=MIR18A |
| MIR106B | MicroRNA 106b | RNA Gene |  | 22 | GC07M100199 | | 10.92 | https://www.genecards.org/cgi-bin/carddisp.pl?gene=MIR106B |
| ACE | Angiotensin I Converting Enzyme | Protein Coding |  | 56 | GC17P063477 | | 10.91 | https://www.genecards.org/cgi-bin/carddisp.pl?gene=ACE |
| SOD2 | Superoxide Dismutase 2 | Protein Coding |  | 57 | GC06M159669 | | 10.87 | https://www.genecards.org/cgi-bin/carddisp.pl?gene=SOD2 |
| IGF1 | Insulin Like Growth Factor 1 | Protein Coding |  | 55 | GC12M102395 | | 10.87 | https://www.genecards.org/cgi-bin/carddisp.pl?gene=IGF1 |
| MIR29A | MicroRNA 29a | RNA Gene |  | 22 | GC07M130876 | | 10.83 | https://www.genecards.org/cgi-bin/carddisp.pl?gene=MIR29A |
| MIRLET7G | MicroRNA Let-7g | RNA Gene |  | 20 | GC03M052268 | | 10.8 | https://www.genecards.org/cgi-bin/carddisp.pl?gene=MIRLET7G |
| DDR2 | Discoidin Domain Receptor Tyrosine Kinase 2 | Protein Coding |  | 59 | GC01P162631 | | 10.79 | https://www.genecards.org/cgi-bin/carddisp.pl?gene=DDR2 |
| EDN1 | Endothelin 1 | Protein Coding |  | 53 | GC06P012290 | | 10.79 | https://www.genecards.org/cgi-bin/carddisp.pl?gene=EDN1 |
| MIR140 | MicroRNA 140 | RNA Gene |  | 21 | GC16P069934 | | 10.79 | https://www.genecards.org/cgi-bin/carddisp.pl?gene=MIR140 |
| MIRLET7D | MicroRNA Let-7d | RNA Gene |  | 22 | GC09P094178 | | 10.71 | https://www.genecards.org/cgi-bin/carddisp.pl?gene=MIRLET7D |
| RMRP | RNA Component Of Mitochondrial RNA Processing Endoribonuclease | RNA Gene |  | 28 | GC09M035655 | | 10.66 | https://www.genecards.org/cgi-bin/carddisp.pl?gene=RMRP |
| RAD51 | RAD51 Recombinase | Protein Coding |  | 57 | GC15P040694 | | 10.64 | https://www.genecards.org/cgi-bin/carddisp.pl?gene=RAD51 |
| MIR34B | MicroRNA 34b | RNA Gene |  | 22 | GC11P111561 | | 10.61 | https://www.genecards.org/cgi-bin/carddisp.pl?gene=MIR34B |
| PRKDC | Protein Kinase, DNA-Activated, Catalytic Subunit | Protein Coding |  | 56 | GC08M047773 | | 10.61 | https://www.genecards.org/cgi-bin/carddisp.pl?gene=PRKDC |
| JAK2 | Janus Kinase 2 | Protein Coding |  | 60 | GC09P004985 | | 10.59 | https://www.genecards.org/cgi-bin/carddisp.pl?gene=JAK2 |
| TNFRSF13B | TNF Receptor Superfamily Member 13B | Protein Coding |  | 53 | GC17M016929 | | 10.58 | https://www.genecards.org/cgi-bin/carddisp.pl?gene=TNFRSF13B |
| MIR199B | MicroRNA 199b | RNA Gene |  | 20 | GC09M128244 | | 10.56 | https://www.genecards.org/cgi-bin/carddisp.pl?gene=MIR199B |
| CD34 | CD34 Molecule | Protein Coding |  | 50 | GC01M207880 | | 10.55 | https://www.genecards.org/cgi-bin/carddisp.pl?gene=CD34 |
| DHFR | Dihydrofolate Reductase | Protein Coding |  | 56 | GC05M080626 | | 10.52 | https://www.genecards.org/cgi-bin/carddisp.pl?gene=DHFR |
| CCN2 | Cellular Communication Network Factor 2 | Protein Coding |  | 45 | GC06M131948 | | 10.46 | https://www.genecards.org/cgi-bin/carddisp.pl?gene=CCN2 |
| PRKN | Parkin RBR E3 Ubiquitin Protein Ligase | Protein Coding |  | 44 | GC06M161348 | | 10.44 | https://www.genecards.org/cgi-bin/carddisp.pl?gene=PRKN |
| WRN | WRN RecQ Like Helicase | Protein Coding |  | 53 | GC08P031010 | | 10.44 | https://www.genecards.org/cgi-bin/carddisp.pl?gene=WRN |
| FGFR2 | Fibroblast Growth Factor Receptor 2 | Protein Coding |  | 61 | GC10M121401 | | 10.38 | https://www.genecards.org/cgi-bin/carddisp.pl?gene=FGFR2 |
| MIR148A | MicroRNA 148a | RNA Gene |  | 19 | GC07M025993 | | 10.38 | https://www.genecards.org/cgi-bin/carddisp.pl?gene=MIR148A |
| FGF7 | Fibroblast Growth Factor 7 | Protein Coding |  | 48 | GC15P049423 | | 10.36 | https://www.genecards.org/cgi-bin/carddisp.pl?gene=FGF7 |
| H19 | H19 Imprinted Maternally Expressed Transcript | RNA Gene |  | 29 | GC11M001995 | | 10.31 | https://www.genecards.org/cgi-bin/carddisp.pl?gene=H19 |
| MIR200C | MicroRNA 200c | RNA Gene |  | 21 | GC12P007638 | | 10.29 | https://www.genecards.org/cgi-bin/carddisp.pl?gene=MIR200C |
| H2AFX | H2A Histone Family Member X | Protein Coding |  | 50 | GC11M119095 | | 10.27 | https://www.genecards.org/cgi-bin/carddisp.pl?gene=H2AFX |
| CYP1A1 | Cytochrome P450 Family 1 Subfamily A Member 1 | Protein Coding |  | 53 | GC15M074719 | | 10.19 | https://www.genecards.org/cgi-bin/carddisp.pl?gene=CYP1A1 |
| MIRLET7C | MicroRNA Let-7c | RNA Gene |  | 21 | GC21P016559 | | 10.17 | https://www.genecards.org/cgi-bin/carddisp.pl?gene=MIRLET7C |
| ZAP70 | Zeta Chain Of T Cell Receptor Associated Protein Kinase 70 | Protein Coding |  | 58 | GC02P097696 | | 10.1 | https://www.genecards.org/cgi-bin/carddisp.pl?gene=ZAP70 |
| EPO | Erythropoietin | Protein Coding |  | 48 | GC07P100720 | | 10.09 | https://www.genecards.org/cgi-bin/carddisp.pl?gene=EPO |
| PLAU | Plasminogen Activator, Urokinase | Protein Coding |  | 58 | GC10P073909 | | 10.09 | https://www.genecards.org/cgi-bin/carddisp.pl?gene=PLAU |
| MIR183 | MicroRNA 183 | RNA Gene |  | 19 | GC07M129785 | | 10.08 | https://www.genecards.org/cgi-bin/carddisp.pl?gene=MIR183 |
| CCR7 | C-C Motif Chemokine Receptor 7 | Protein Coding |  | 51 | GC17M040556 | | 10.06 | https://www.genecards.org/cgi-bin/carddisp.pl?gene=CCR7 |
| KDR | Kinase Insert Domain Receptor | Protein Coding |  | 60 | GC04M055078 | | 10.05 | https://www.genecards.org/cgi-bin/carddisp.pl?gene=KDR |
| BMP6 | Bone Morphogenetic Protein 6 | Protein Coding |  | 50 | GC06P007726 | | 10.03 | https://www.genecards.org/cgi-bin/carddisp.pl?gene=BMP6 |
| MIR200B | MicroRNA 200b | RNA Gene |  | 21 | GC01P001167 | | 10.03 | https://www.genecards.org/cgi-bin/carddisp.pl?gene=MIR200B |
| MIR96 | MicroRNA 96 | RNA Gene |  | 20 | GC07M129774 | | 10.03 | https://www.genecards.org/cgi-bin/carddisp.pl?gene=MIR96 |
| TLR9 | Toll Like Receptor 9 | Protein Coding |  | 52 | GC03M052222 | | 10.01 | https://www.genecards.org/cgi-bin/carddisp.pl?gene=TLR9 |
| JAK3 | Janus Kinase 3 | Protein Coding |  | 58 | GC19M017790 | | 9.99 | https://www.genecards.org/cgi-bin/carddisp.pl?gene=JAK3 |
| F2 | Coagulation Factor II, Thrombin | Protein Coding |  | 55 | GC11P046740 | | 9.97 | https://www.genecards.org/cgi-bin/carddisp.pl?gene=F2 |
| SERPINH1 | Serpin Family H Member 1 | Protein Coding |  | 52 | GC11P075562 | | 9.97 | https://www.genecards.org/cgi-bin/carddisp.pl?gene=SERPINH1 |
| TFRC | Transferrin Receptor | Protein Coding |  | 54 | GC03M196027 | | 9.95 | https://www.genecards.org/cgi-bin/carddisp.pl?gene=TFRC |
| MYCN | MYCN Proto-Oncogene, BHLH Transcription Factor | Protein Coding |  | 53 | GC02P015954 | | 9.94 | https://www.genecards.org/cgi-bin/carddisp.pl?gene=MYCN |
| IFNA2 | Interferon Alpha 2 | Protein Coding |  | 48 | GC09M021374 | | 9.92 | https://www.genecards.org/cgi-bin/carddisp.pl?gene=IFNA2 |
| ENO2 | Enolase 2 | Protein Coding |  | 54 | GC12P006913 | | 9.9 | https://www.genecards.org/cgi-bin/carddisp.pl?gene=ENO2 |
| MIR150 | MicroRNA 150 | RNA Gene |  | 22 | GC19M049500 | | 9.82 | https://www.genecards.org/cgi-bin/carddisp.pl?gene=MIR150 |
| MIR26A1 | MicroRNA 26a-1 | RNA Gene |  | 21 | GC03P037969 | | 9.81 | https://www.genecards.org/cgi-bin/carddisp.pl?gene=MIR26A1 |
| MIR204 | MicroRNA 204 | RNA Gene |  | 22 | GC09M070809 | | 9.8 | https://www.genecards.org/cgi-bin/carddisp.pl?gene=MIR204 |
| HOTAIR | HOX Transcript Antisense RNA | RNA Gene |  | 26 | GC12M053962 | | 9.8 | https://www.genecards.org/cgi-bin/carddisp.pl?gene=HOTAIR |
| IFNA1 | Interferon Alpha 1 | Protein Coding |  | 45 | GC09P021461 | | 9.79 | https://www.genecards.org/cgi-bin/carddisp.pl?gene=IFNA1 |
| IL5 | Interleukin 5 | Protein Coding |  | 51 | GC05M132541 | | 9.79 | https://www.genecards.org/cgi-bin/carddisp.pl?gene=IL5 |
| SETBP1 | SET Binding Protein 1 | Protein Coding |  | 47 | GC18P044680 | | 9.78 | https://www.genecards.org/cgi-bin/carddisp.pl?gene=SETBP1 |
| MEG3 | Maternally Expressed 3 | RNA Gene |  | 32 | GC14P103811 | | 9.78 | https://www.genecards.org/cgi-bin/carddisp.pl?gene=MEG3 |
| PMS2 | PMS1 Homolog 2, Mismatch Repair System Component | Protein Coding |  | 54 | GC07M005973 | | 9.74 | https://www.genecards.org/cgi-bin/carddisp.pl?gene=PMS2 |
| DNMT3A | DNA Methyltransferase 3 Alpha | Protein Coding |  | 57 | GC02M025194 | | 9.7 | https://www.genecards.org/cgi-bin/carddisp.pl?gene=DNMT3A |
| MIR224 | MicroRNA 224 | RNA Gene |  | 18 | GC0XM151958 | | 9.68 | https://www.genecards.org/cgi-bin/carddisp.pl?gene=MIR224 |
| LRBA | LPS Responsive Beige-Like Anchor Protein | Protein Coding |  | 47 | GC04M150264 | | 9.66 | https://www.genecards.org/cgi-bin/carddisp.pl?gene=LRBA |
| MIR192 | MicroRNA 192 | RNA Gene |  | 22 | GC11M064891 | | 9.66 | https://www.genecards.org/cgi-bin/carddisp.pl?gene=MIR192 |
| MIR181C | MicroRNA 181c | RNA Gene |  | 21 | GC19P013858 | | 9.66 | https://www.genecards.org/cgi-bin/carddisp.pl?gene=MIR181C |
| TGFA | Transforming Growth Factor Alpha | Protein Coding |  | 53 | GC02M070410 | | 9.64 | https://www.genecards.org/cgi-bin/carddisp.pl?gene=TGFA |
| MALAT1 | Metastasis Associated Lung Adenocarcinoma Transcript 1 | RNA Gene |  | 24 | GC11P065497 | | 9.63 | https://www.genecards.org/cgi-bin/carddisp.pl?gene=MALAT1 |
| CCL17 | C-C Motif Chemokine Ligand 17 | Protein Coding |  | 45 | GC16P057404 | | 9.62 | https://www.genecards.org/cgi-bin/carddisp.pl?gene=CCL17 |
| GYPA | Glycophorin A (MNS Blood Group) | Protein Coding |  | 49 | GC04M144109 | | 9.55 | https://www.genecards.org/cgi-bin/carddisp.pl?gene=GYPA |
| PLAT | Plasminogen Activator, Tissue Type | Protein Coding |  | 56 | GC08M042174 | | 9.54 | https://www.genecards.org/cgi-bin/carddisp.pl?gene=PLAT |
| MCL1 | MCL1 Apoptosis Regulator, BCL2 Family Member | Protein Coding |  | 54 | GC01M150596 | | 9.5 | https://www.genecards.org/cgi-bin/carddisp.pl?gene=MCL1 |
| RNASE3 | Ribonuclease A Family Member 3 | Protein Coding |  | 46 | GC14P020891 | | 9.49 | https://www.genecards.org/cgi-bin/carddisp.pl?gene=RNASE3 |
| SYP | Synaptophysin | Protein Coding |  | 50 | GC0XM049187 | | 9.47 | https://www.genecards.org/cgi-bin/carddisp.pl?gene=SYP |
| CARD11 | Caspase Recruitment Domain Family Member 11 | Protein Coding |  | 53 | GC07M002912 | | 9.46 | https://www.genecards.org/cgi-bin/carddisp.pl?gene=CARD11 |
| NFE2L2 | Nuclear Factor, Erythroid 2 Like 2 | Protein Coding |  | 54 | GC02M177227 | | 9.45 | https://www.genecards.org/cgi-bin/carddisp.pl?gene=NFE2L2 |
| HSPD1 | Heat Shock Protein Family D (Hsp60) Member 1 | Protein Coding |  | 54 | GC02M197486 | | 9.43 | https://www.genecards.org/cgi-bin/carddisp.pl?gene=HSPD1 |
| MAPK14 | Mitogen-Activated Protein Kinase 14 | Protein Coding |  | 59 | GC06P043143 | | 9.37 | https://www.genecards.org/cgi-bin/carddisp.pl?gene=MAPK14 |
| MIR146B | MicroRNA 146b | RNA Gene |  | 20 | GC10P102436 | | 9.37 | https://www.genecards.org/cgi-bin/carddisp.pl?gene=MIR146B |
| MIR185 | MicroRNA 185 | RNA Gene |  | 22 | GC22P020034 | | 9.35 | https://www.genecards.org/cgi-bin/carddisp.pl?gene=MIR185 |
| MS4A1 | Membrane Spanning 4-Domains A1 | Protein Coding |  | 54 | GC11P060474 | | 9.32 | https://www.genecards.org/cgi-bin/carddisp.pl?gene=MS4A1 |
| TP53BP1 | Tumor Protein P53 Binding Protein 1 | Protein Coding |  | 49 | GC15M043403 | | 9.32 | https://www.genecards.org/cgi-bin/carddisp.pl?gene=TP53BP1 |
| CD36 | CD36 Molecule | Protein Coding |  | 54 | GC07P080369 | | 9.31 | https://www.genecards.org/cgi-bin/carddisp.pl?gene=CD36 |
| TNC | Tenascin C | Protein Coding |  | 53 | GC09M115019 | | 9.28 | https://www.genecards.org/cgi-bin/carddisp.pl?gene=TNC |
| MIR130A | MicroRNA 130a | RNA Gene |  | 20 | GC11P057641 | | 9.27 | https://www.genecards.org/cgi-bin/carddisp.pl?gene=MIR130A |
| MIR30D | MicroRNA 30d | RNA Gene |  | 17 | GC08M134804 | | 9.27 | https://www.genecards.org/cgi-bin/carddisp.pl?gene=MIR30D |
| PLAUR | Plasminogen Activator, Urokinase Receptor | Protein Coding |  | 51 | GC19M043646 | | 9.26 | https://www.genecards.org/cgi-bin/carddisp.pl?gene=PLAUR |
| GAS5 | Growth Arrest Specific 5 | RNA Gene |  | 23 | GC01M174052 | | 9.16 | https://www.genecards.org/cgi-bin/carddisp.pl?gene=GAS5 |
| CDK1 | Cyclin Dependent Kinase 1 | Protein Coding |  | 52 | GC10P060772 | | 9.16 | https://www.genecards.org/cgi-bin/carddisp.pl?gene=CDK1 |
| CCL18 | C-C Motif Chemokine Ligand 18 | Protein Coding |  | 42 | GC17P036064 | | 9.13 | https://www.genecards.org/cgi-bin/carddisp.pl?gene=CCL18 |
| PCNA | Proliferating Cell Nuclear Antigen | Protein Coding |  | 57 | GC20M005114 | | 9.12 | https://www.genecards.org/cgi-bin/carddisp.pl?gene=PCNA |
| LTF | Lactotransferrin | Protein Coding |  | 50 | GC03M046435 | | 9.11 | https://www.genecards.org/cgi-bin/carddisp.pl?gene=LTF |
| PRNP | Prion Protein | Protein Coding |  | 53 | GC20P004615 | | 9.1 | https://www.genecards.org/cgi-bin/carddisp.pl?gene=PRNP |
| IL18 | Interleukin 18 | Protein Coding |  | 50 | GC11M112143 | | 9.06 | https://www.genecards.org/cgi-bin/carddisp.pl?gene=IL18 |
| TYMS | Thymidylate Synthetase | Protein Coding |  | 56 | GC18P000657 | | 9.04 | https://www.genecards.org/cgi-bin/carddisp.pl?gene=TYMS |
| MBL2 | Mannose Binding Lectin 2 | Protein Coding |  | 53 | GC10M052760 | | 9.04 | https://www.genecards.org/cgi-bin/carddisp.pl?gene=MBL2 |
| BTK | Bruton Tyrosine Kinase | Protein Coding |  | 60 | GC0XM101349 | | 9.03 | https://www.genecards.org/cgi-bin/carddisp.pl?gene=BTK |
| CXCL9 | C-X-C Motif Chemokine Ligand 9 | Protein Coding |  | 44 | GC04M076001 | | 9.02 | https://www.genecards.org/cgi-bin/carddisp.pl?gene=CXCL9 |
| IL1R1 | Interleukin 1 Receptor Type 1 | Protein Coding |  | 52 | GC02P102136 | | 9 | https://www.genecards.org/cgi-bin/carddisp.pl?gene=IL1R1 |
| NQO1 | NAD(P)H Quinone Dehydrogenase 1 | Protein Coding |  | 56 | GC16M069706 | | 8.97 | https://www.genecards.org/cgi-bin/carddisp.pl?gene=NQO1 |
| PIK3C2A | Phosphatidylinositol-4-Phosphate 3-Kinase Catalytic Subunit Type 2 Alpha | Protein Coding |  | 51 | GC11M017136 | | 8.93 | https://www.genecards.org/cgi-bin/carddisp.pl?gene=PIK3C2A |
| CTLA4 | Cytotoxic T-Lymphocyte Associated Protein 4 | Protein Coding |  | 53 | GC02P203867 | | 8.92 | https://www.genecards.org/cgi-bin/carddisp.pl?gene=CTLA4 |
| ADA | Adenosine Deaminase | Protein Coding |  | 57 | GC20M044620 | | 8.91 | https://www.genecards.org/cgi-bin/carddisp.pl?gene=ADA |
| TF | Transferrin | Protein Coding |  | 56 | GC03P133663 | | 8.91 | https://www.genecards.org/cgi-bin/carddisp.pl?gene=TF |
| MIR137 | MicroRNA 137 | RNA Gene |  | 19 | GC01M098046 | | 8.89 | https://www.genecards.org/cgi-bin/carddisp.pl?gene=MIR137 |
| SMAD3 | SMAD Family Member 3 | Protein Coding |  | 55 | GC15P067063 | | 8.89 | https://www.genecards.org/cgi-bin/carddisp.pl?gene=SMAD3 |
| SERPINA3 | Serpin Family A Member 3 | Protein Coding |  | 50 | GC14P094612 | | 8.86 | https://www.genecards.org/cgi-bin/carddisp.pl?gene=SERPINA3 |
| MIR372 | MicroRNA 372 | RNA Gene |  | 19 | GC19P054025 | | 8.85 | https://www.genecards.org/cgi-bin/carddisp.pl?gene=MIR372 |
| APEX1 | Apurinic/Apyrimidinic Endodeoxyribonuclease 1 | Protein Coding |  | 51 | GC14P020455 | | 8.82 | https://www.genecards.org/cgi-bin/carddisp.pl?gene=APEX1 |
| THBD | Thrombomodulin | Protein Coding |  | 50 | GC20M023026 | | 8.82 | https://www.genecards.org/cgi-bin/carddisp.pl?gene=THBD |
| ITGB1 | Integrin Subunit Beta 1 | Protein Coding |  | 56 | GC10M032936 | | 8.82 | https://www.genecards.org/cgi-bin/carddisp.pl?gene=ITGB1 |
| TGFBR1 | Transforming Growth Factor Beta Receptor 1 | Protein Coding |  | 59 | GC09P099104 | | 8.81 | https://www.genecards.org/cgi-bin/carddisp.pl?gene=TGFBR1 |
| TMEM173 | Transmembrane Protein 173 | Protein Coding |  | 48 | GC05M139475 | | 8.79 | https://www.genecards.org/cgi-bin/carddisp.pl?gene=TMEM173 |
| GPT | Glutamic--Pyruvic Transaminase | Protein Coding |  | 48 | GC08P144502 | | 8.76 | https://www.genecards.org/cgi-bin/carddisp.pl?gene=GPT |
| KRT7 | Keratin 7 | Protein Coding |  | 48 | GC12P052232 | | 8.76 | https://www.genecards.org/cgi-bin/carddisp.pl?gene=KRT7 |
| MAPT | Microtubule Associated Protein Tau | Protein Coding |  | 57 | GC17P045894 | | 8.75 | https://www.genecards.org/cgi-bin/carddisp.pl?gene=MAPT |
| PDGFA | Platelet Derived Growth Factor Subunit A | Protein Coding |  | 50 | GC07M000497 | | 8.75 | https://www.genecards.org/cgi-bin/carddisp.pl?gene=PDGFA |
| SMAD2 | SMAD Family Member 2 | Protein Coding |  | 53 | GC18M047809 | | 8.71 | https://www.genecards.org/cgi-bin/carddisp.pl?gene=SMAD2 |
| UCA1 | Urothelial Cancer Associated 1 | RNA Gene |  | 23 | GC19P015828 | | 8.71 | https://www.genecards.org/cgi-bin/carddisp.pl?gene=UCA1 |
| CR1 | Complement C3b/C4b Receptor 1 (Knops Blood Group) | Protein Coding |  | 51 | GC01P207496 | | 8.7 | https://www.genecards.org/cgi-bin/carddisp.pl?gene=CR1 |
| CAV1 | Caveolin 1 | Protein Coding |  | 54 | GC07P116524 | | 8.68 | https://www.genecards.org/cgi-bin/carddisp.pl?gene=CAV1 |
| NEU1 | Neuraminidase 1 | Protein Coding |  | 50 | GC06M031857 | | 8.67 | https://www.genecards.org/cgi-bin/carddisp.pl?gene=NEU1 |
| CD55 | CD55 Molecule (Cromer Blood Group) | Protein Coding |  | 53 | GC01P207321 | | 8.61 | https://www.genecards.org/cgi-bin/carddisp.pl?gene=CD55 |
| GFI1 | Growth Factor Independent 1 Transcriptional Repressor | Protein Coding |  | 48 | GC01M092412 | | 8.61 | https://www.genecards.org/cgi-bin/carddisp.pl?gene=GFI1 |
| B2M | Beta-2-Microglobulin | Protein Coding |  | 56 | GC15P044711 | | 8.59 | https://www.genecards.org/cgi-bin/carddisp.pl?gene=B2M |
| DHPS | Deoxyhypusine Synthase | Protein Coding |  | 47 | GC19M012676 | | 8.59 | https://www.genecards.org/cgi-bin/carddisp.pl?gene=DHPS |
| PVT1 | Pvt1 Oncogene | RNA Gene |  | 26 | GC08P127808 | | 8.58 | https://www.genecards.org/cgi-bin/carddisp.pl?gene=PVT1 |
| CD40 | CD40 Molecule | Protein Coding |  | 54 | GC20P046118 | | 8.53 | https://www.genecards.org/cgi-bin/carddisp.pl?gene=CD40 |
| TUG1 | Taurine Up-Regulated 1 | RNA Gene |  | 25 | GC22P030969 | | 8.47 | https://www.genecards.org/cgi-bin/carddisp.pl?gene=TUG1 |
| PDGFRB | Platelet Derived Growth Factor Receptor Beta | Protein Coding |  | 62 | GC05M150113 | | 8.47 | https://www.genecards.org/cgi-bin/carddisp.pl?gene=PDGFRB |
| MIR133B | MicroRNA 133b | RNA Gene |  | 22 | GC06P052148 | | 8.45 | https://www.genecards.org/cgi-bin/carddisp.pl?gene=MIR133B |
| TYMP | Thymidine Phosphorylase | Protein Coding |  | 53 | GC22M050525 | | 8.44 | https://www.genecards.org/cgi-bin/carddisp.pl?gene=TYMP |
| BCL10 | BCL10 Immune Signaling Adaptor | Protein Coding |  | 52 | GC01M085265 | | 8.35 | https://www.genecards.org/cgi-bin/carddisp.pl?gene=BCL10 |
| CSF1 | Colony Stimulating Factor 1 | Protein Coding |  | 50 | GC01P109911 | | 8.35 | https://www.genecards.org/cgi-bin/carddisp.pl?gene=CSF1 |
| CCL11 | C-C Motif Chemokine Ligand 11 | Protein Coding |  | 49 | GC17P034285 | | 8.34 | https://www.genecards.org/cgi-bin/carddisp.pl?gene=CCL11 |
| HMOX1 | Heme Oxygenase 1 | Protein Coding |  | 59 | GC22P035380 | | 8.29 | https://www.genecards.org/cgi-bin/carddisp.pl?gene=HMOX1 |
| SOD1 | Superoxide Dismutase 1 | Protein Coding |  | 59 | GC21P031659 | | 8.29 | https://www.genecards.org/cgi-bin/carddisp.pl?gene=SOD1 |
| XIAP | X-Linked Inhibitor Of Apoptosis | Protein Coding |  | 54 | GC0XP123859 | | 8.28 | https://www.genecards.org/cgi-bin/carddisp.pl?gene=XIAP |
| IGF1R | Insulin Like Growth Factor 1 Receptor | Protein Coding |  | 62 | GC15P098648 | | 8.27 | https://www.genecards.org/cgi-bin/carddisp.pl?gene=IGF1R |
| S100B | S100 Calcium Binding Protein B | Protein Coding |  | 52 | GC21M046922 | | 8.26 | https://www.genecards.org/cgi-bin/carddisp.pl?gene=S100B |
| CDKN2B-AS1 | CDKN2B Antisense RNA 1 | RNA Gene |  | 21 | GC09P021994 | | 8.25 | https://www.genecards.org/cgi-bin/carddisp.pl?gene=CDKN2B-AS1 |
| MSH2 | MutS Homolog 2 | Protein Coding |  | 54 | GC02P047402 | | 8.23 | https://www.genecards.org/cgi-bin/carddisp.pl?gene=MSH2 |
| CD79B | CD79b Molecule | Protein Coding |  | 52 | GC17M063928 | | 8.23 | https://www.genecards.org/cgi-bin/carddisp.pl?gene=CD79B |
| TNFRSF11A | TNF Receptor Superfamily Member 11a | Protein Coding |  | 53 | GC18P062325 | | 8.22 | https://www.genecards.org/cgi-bin/carddisp.pl?gene=TNFRSF11A |
| CHGA | Chromogranin A | Protein Coding |  | 48 | GC14P092923 | | 8.22 | https://www.genecards.org/cgi-bin/carddisp.pl?gene=CHGA |
| NOTCH1 | Notch Receptor 1 | Protein Coding |  | 59 | GC09M136558 | | 8.22 | https://www.genecards.org/cgi-bin/carddisp.pl?gene=NOTCH1 |
| PPP2R1B | Protein Phosphatase 2 Scaffold Subunit Abeta | Protein Coding |  | 54 | GC11M111695 | | 8.2 | https://www.genecards.org/cgi-bin/carddisp.pl?gene=PPP2R1B |
| AR | Androgen Receptor | Protein Coding |  | 59 | GC0XP067544 | | 8.2 | https://www.genecards.org/cgi-bin/carddisp.pl?gene=AR |
| DLEC1 | DLEC1 Cilia And Flagella Associated Protein | Protein Coding |  | 43 | GC03P038080 | | 8.15 | https://www.genecards.org/cgi-bin/carddisp.pl?gene=DLEC1 |
| IL2RA | Interleukin 2 Receptor Subunit Alpha | Protein Coding |  | 57 | GC10M006010 | | 8.14 | https://www.genecards.org/cgi-bin/carddisp.pl?gene=IL2RA |
| MIR98 | MicroRNA 98 | RNA Gene |  | 18 | GC0XM053573 | | 8.08 | https://www.genecards.org/cgi-bin/carddisp.pl?gene=MIR98 |
| DICER1 | Dicer 1, Ribonuclease III | Protein Coding |  | 54 | GC14M095086 | | 8.07 | https://www.genecards.org/cgi-bin/carddisp.pl?gene=DICER1 |
| CCAT1 | Colon Cancer Associated Transcript 1 | RNA Gene |  | 17 | GC08M127207 | | 8.06 | https://www.genecards.org/cgi-bin/carddisp.pl?gene=CCAT1 |
| SLC22A18 | Solute Carrier Family 22 Member 18 | Protein Coding |  | 48 | GC11P002920 | | 8.01 | https://www.genecards.org/cgi-bin/carddisp.pl?gene=SLC22A18 |
| TNFSF10 | TNF Superfamily Member 10 | Protein Coding |  | 53 | GC03M172505 | | 8.01 | https://www.genecards.org/cgi-bin/carddisp.pl?gene=TNFSF10 |
| CAT | Catalase | Protein Coding |  | 56 | GC11P034460 | | 8 | https://www.genecards.org/cgi-bin/carddisp.pl?gene=CAT |
| ITGA4 | Integrin Subunit Alpha 4 | Protein Coding |  | 54 | GC02P181456 | | 8 | https://www.genecards.org/cgi-bin/carddisp.pl?gene=ITGA4 |
| KITLG | KIT Ligand | Protein Coding |  | 51 | GC12M088492 | | 7.99 | https://www.genecards.org/cgi-bin/carddisp.pl?gene=KITLG |
| KRT19 | Keratin 19 | Protein Coding |  | 52 | GC17M041523 | | 7.97 | https://www.genecards.org/cgi-bin/carddisp.pl?gene=KRT19 |
| MIR486-1 | MicroRNA 486-1 | RNA Gene |  | 16 | GC08M041660 | | 7.96 | https://www.genecards.org/cgi-bin/carddisp.pl?gene=MIR486-1 |
| CYP2A6 | Cytochrome P450 Family 2 Subfamily A Member 6 | Protein Coding |  | 54 | GC19M040843 | | 7.95 | https://www.genecards.org/cgi-bin/carddisp.pl?gene=CYP2A6 |
| NFKBIA | NFKB Inhibitor Alpha | Protein Coding |  | 57 | GC14M035401 | | 7.95 | https://www.genecards.org/cgi-bin/carddisp.pl?gene=NFKBIA |
| XIST | X Inactive Specific Transcript | RNA Gene |  | 25 | GC0XM073820 | | 7.95 | https://www.genecards.org/cgi-bin/carddisp.pl?gene=XIST |
| NSD1 | Nuclear Receptor Binding SET Domain Protein 1 | Protein Coding |  | 49 | GC05P177134 | | 7.93 | https://www.genecards.org/cgi-bin/carddisp.pl?gene=NSD1 |
| ROS1 | ROS Proto-Oncogene 1, Receptor Tyrosine Kinase | Protein Coding |  | 51 | GC06M117287 | | 7.92 | https://www.genecards.org/cgi-bin/carddisp.pl?gene=ROS1 |
| MIRLET7A3 | MicroRNA Let-7a-3 | RNA Gene |  | 20 | GC22P046112 | | 7.92 | https://www.genecards.org/cgi-bin/carddisp.pl?gene=MIRLET7A3 |
| MIR342 | MicroRNA 342 | RNA Gene |  | 20 | GC14P100109 | | 7.92 | https://www.genecards.org/cgi-bin/carddisp.pl?gene=MIR342 |
| MIR197 | MicroRNA 197 | RNA Gene |  | 19 | GC01P109549 | | 7.92 | https://www.genecards.org/cgi-bin/carddisp.pl?gene=MIR197 |
| DCK | Deoxycytidine Kinase | Protein Coding |  | 52 | GC04P070992 | | 7.9 | https://www.genecards.org/cgi-bin/carddisp.pl?gene=DCK |
| CCNA2 | Cyclin A2 | Protein Coding |  | 51 | GC04M121816 | | 7.87 | https://www.genecards.org/cgi-bin/carddisp.pl?gene=CCNA2 |
| CCNB1 | Cyclin B1 | Protein Coding |  | 53 | GC05P069167 | | 7.87 | https://www.genecards.org/cgi-bin/carddisp.pl?gene=CCNB1 |
| CEACAM5 | Carcinoembryonic Antigen Related Cell Adhesion Molecule 5 | Protein Coding |  | 48 | GC19P041709 | | 7.86 | https://www.genecards.org/cgi-bin/carddisp.pl?gene=CEACAM5 |
| BRCA2 | BRCA2 DNA Repair Associated | Protein Coding |  | 55 | GC13P032315 | | 7.84 | https://www.genecards.org/cgi-bin/carddisp.pl?gene=BRCA2 |
| CDKN3 | Cyclin Dependent Kinase Inhibitor 3 | Protein Coding |  | 47 | GC14P054398 | | 7.83 | https://www.genecards.org/cgi-bin/carddisp.pl?gene=CDKN3 |
| SELE | Selectin E | Protein Coding |  | 50 | GC01M169722 | | 7.83 | https://www.genecards.org/cgi-bin/carddisp.pl?gene=SELE |
| ATR | ATR Serine/Threonine Kinase | Protein Coding |  | 58 | GC03M142449 | | 7.82 | https://www.genecards.org/cgi-bin/carddisp.pl?gene=ATR |
| SHH | Sonic Hedgehog Signaling Molecule | Protein Coding |  | 56 | GC07M155799 | | 7.79 | https://www.genecards.org/cgi-bin/carddisp.pl?gene=SHH |
| MIR429 | MicroRNA 429 | RNA Gene |  | 21 | GC01P001212 | | 7.74 | https://www.genecards.org/cgi-bin/carddisp.pl?gene=MIR429 |
| IGF2R | Insulin Like Growth Factor 2 Receptor | Protein Coding |  | 51 | GC06P159969 | | 7.69 | https://www.genecards.org/cgi-bin/carddisp.pl?gene=IGF2R |
| MGMT | O-6-Methylguanine-DNA Methyltransferase | Protein Coding |  | 55 | GC10P129467 | | 7.69 | https://www.genecards.org/cgi-bin/carddisp.pl?gene=MGMT |
| IRAK4 | Interleukin 1 Receptor Associated Kinase 4 | Protein Coding |  | 54 | GC12P043758 | | 7.69 | https://www.genecards.org/cgi-bin/carddisp.pl?gene=IRAK4 |
| RARB | Retinoic Acid Receptor Beta | Protein Coding |  | 56 | GC03P025194 | | 7.56 | https://www.genecards.org/cgi-bin/carddisp.pl?gene=RARB |
| MUC5AC | Mucin 5AC, Oligomeric Mucus/Gel-Forming | Protein Coding |  | 44 | GC11P001151 | | 7.56 | https://www.genecards.org/cgi-bin/carddisp.pl?gene=MUC5AC |
| RPS6KB1 | Ribosomal Protein S6 Kinase B1 | Protein Coding |  | 56 | GC17P059893 | | 7.54 | https://www.genecards.org/cgi-bin/carddisp.pl?gene=RPS6KB1 |
| INS | Insulin | Protein Coding |  | 54 | GC11M002159 | | 7.52 | https://www.genecards.org/cgi-bin/carddisp.pl?gene=INS |
| TNFRSF1B | TNF Receptor Superfamily Member 1B | Protein Coding |  | 54 | GC01P012167 | | 7.51 | https://www.genecards.org/cgi-bin/carddisp.pl?gene=TNFRSF1B |
| SERPINA1 | Serpin Family A Member 1 | Protein Coding |  | 56 | GC14M094376 | | 7.47 | https://www.genecards.org/cgi-bin/carddisp.pl?gene=SERPINA1 |
| TEAD1 | TEA Domain Transcription Factor 1 | Protein Coding |  | 53 | GC11P012674 | | 7.44 | https://www.genecards.org/cgi-bin/carddisp.pl?gene=TEAD1 |
| PECAM1 | Platelet And Endothelial Cell Adhesion Molecule 1 | Protein Coding |  | 46 | GC17M064319 | | 7.43 | https://www.genecards.org/cgi-bin/carddisp.pl?gene=PECAM1 |
| BCL11B | BAF Chromatin Remodeling Complex Subunit BCL11B | Protein Coding |  | 48 | GC14M099169 | | 7.42 | https://www.genecards.org/cgi-bin/carddisp.pl?gene=BCL11B |
| GADD45A | Growth Arrest And DNA Damage Inducible Alpha | Protein Coding |  | 51 | GC01P067685 | | 7.41 | https://www.genecards.org/cgi-bin/carddisp.pl?gene=GADD45A |
| CACNA1C | Calcium Voltage-Gated Channel Subunit Alpha1 C | Protein Coding |  | 56 | GC12P001970 | | 7.39 | https://www.genecards.org/cgi-bin/carddisp.pl?gene=CACNA1C |
| HLA-B | Major Histocompatibility Complex, Class I, B | Protein Coding |  | 51 | GC06M031277 | | 7.39 | https://www.genecards.org/cgi-bin/carddisp.pl?gene=HLA-B |
| HSP90AA1 | Heat Shock Protein 90 Alpha Family Class A Member 1 | Protein Coding |  | 54 | GC14M102080 | | 7.39 | https://www.genecards.org/cgi-bin/carddisp.pl?gene=HSP90AA1 |
| CYBB | Cytochrome B-245 Beta Chain | Protein Coding |  | 53 | GC0XP037780 | | 7.39 | https://www.genecards.org/cgi-bin/carddisp.pl?gene=CYBB |
| IKZF3 | IKAROS Family Zinc Finger 3 | Protein Coding |  | 48 | GC17M039759 | | 7.38 | https://www.genecards.org/cgi-bin/carddisp.pl?gene=IKZF3 |
| NOTCH3 | Notch Receptor 3 | Protein Coding |  | 56 | GC19M015131 | | 7.37 | https://www.genecards.org/cgi-bin/carddisp.pl?gene=NOTCH3 |
| ACP5 | Acid Phosphatase 5, Tartrate Resistant | Protein Coding |  | 53 | GC19M011574 | | 7.35 | https://www.genecards.org/cgi-bin/carddisp.pl?gene=ACP5 |
| CASP10 | Caspase 10 | Protein Coding |  | 54 | GC02P201182 | | 7.32 | https://www.genecards.org/cgi-bin/carddisp.pl?gene=CASP10 |
| IFNB1 | Interferon Beta 1 | Protein Coding |  | 48 | GC09M021077 | | 7.31 | https://www.genecards.org/cgi-bin/carddisp.pl?gene=IFNB1 |
| RAC1 | Rac Family Small GTPase 1 | Protein Coding |  | 56 | GC07P006380 | | 7.24 | https://www.genecards.org/cgi-bin/carddisp.pl?gene=RAC1 |
| CALCA | Calcitonin Related Polypeptide Alpha | Protein Coding |  | 49 | GC11M014945 | | 7.23 | https://www.genecards.org/cgi-bin/carddisp.pl?gene=CALCA |
| CD38 | CD38 Molecule | Protein Coding |  | 51 | GC04P015779 | | 7.21 | https://www.genecards.org/cgi-bin/carddisp.pl?gene=CD38 |
| LINC-ROR | Long Intergenic Non-Protein Coding RNA, Regulator Of Reprogramming | RNA Gene |  | 17 | GC18M057054 | | 7.21 | https://www.genecards.org/cgi-bin/carddisp.pl?gene=LINC-ROR |
| TCF3 | Transcription Factor 3 | Protein Coding |  | 52 | GC19M001609 | | 7.2 | https://www.genecards.org/cgi-bin/carddisp.pl?gene=TCF3 |
| UNC119 | Unc-119 Lipid Binding Chaperone | Protein Coding |  | 48 | GC17M028546 | | 7.18 | https://www.genecards.org/cgi-bin/carddisp.pl?gene=UNC119 |
| FLT4 | Fms Related Tyrosine Kinase 4 | Protein Coding |  | 59 | GC05M180607 | | 7.18 | https://www.genecards.org/cgi-bin/carddisp.pl?gene=FLT4 |
| GJA1 | Gap Junction Protein Alpha 1 | Protein Coding |  | 57 | GC06P121436 | | 7.17 | https://www.genecards.org/cgi-bin/carddisp.pl?gene=GJA1 |
| SH2D1A | SH2 Domain Containing 1A | Protein Coding |  | 53 | GC0XP124227 | | 7.16 | https://www.genecards.org/cgi-bin/carddisp.pl?gene=SH2D1A |
| VEGFC | Vascular Endothelial Growth Factor C | Protein Coding |  | 53 | GC04M176683 | | 7.15 | https://www.genecards.org/cgi-bin/carddisp.pl?gene=VEGFC |
| TFE3 | Transcription Factor Binding To IGHM Enhancer 3 | Protein Coding |  | 49 | GC0XM049028 | | 7.15 | https://www.genecards.org/cgi-bin/carddisp.pl?gene=TFE3 |
| TAC1 | Tachykinin Precursor 1 | Protein Coding |  | 50 | GC07P097731 | | 7.12 | https://www.genecards.org/cgi-bin/carddisp.pl?gene=TAC1 |
| BIRC3 | Baculoviral IAP Repeat Containing 3 | Protein Coding |  | 53 | GC11P102317 | | 7.1 | https://www.genecards.org/cgi-bin/carddisp.pl?gene=BIRC3 |
| CXCL2 | C-X-C Motif Chemokine Ligand 2 | Protein Coding |  | 46 | GC04M074097 | | 7.08 | https://www.genecards.org/cgi-bin/carddisp.pl?gene=CXCL2 |
| CDKN1B | Cyclin Dependent Kinase Inhibitor 1B | Protein Coding |  | 55 | GC12P012716 | | 7.08 | https://www.genecards.org/cgi-bin/carddisp.pl?gene=CDKN1B |
| TRAF3 | TNF Receptor Associated Factor 3 | Protein Coding |  | 53 | GC14P103758 | | 7.08 | https://www.genecards.org/cgi-bin/carddisp.pl?gene=TRAF3 |
| TNFSF11 | TNF Superfamily Member 11 | Protein Coding |  | 54 | GC13P042562 | | 7.05 | https://www.genecards.org/cgi-bin/carddisp.pl?gene=TNFSF11 |
| VWF | Von Willebrand Factor | Protein Coding |  | 55 | GC12M005917 | | 7.04 | https://www.genecards.org/cgi-bin/carddisp.pl?gene=VWF |
| NEAT1 | Nuclear Paraspeckle Assembly Transcript 1 | RNA Gene |  | 24 | GC11P065550 | | 7.02 | https://www.genecards.org/cgi-bin/carddisp.pl?gene=NEAT1 |
| NCAM1 | Neural Cell Adhesion Molecule 1 | Protein Coding |  | 52 | GC11P112961 | | 7.01 | https://www.genecards.org/cgi-bin/carddisp.pl?gene=NCAM1 |
| IL7 | Interleukin 7 | Protein Coding |  | 46 | GC08M078689 | | 6.99 | https://www.genecards.org/cgi-bin/carddisp.pl?gene=IL7 |
| CFH | Complement Factor H | Protein Coding |  | 52 | GC01P196621 | | 6.96 | https://www.genecards.org/cgi-bin/carddisp.pl?gene=CFH |
| PCAT1 | Prostate Cancer Associated Transcript 1 | RNA Gene |  | 20 | GC08P126559 | | 6.96 | https://www.genecards.org/cgi-bin/carddisp.pl?gene=PCAT1 |
| ELN | Elastin | Protein Coding |  | 50 | GC07P074027 | | 6.96 | https://www.genecards.org/cgi-bin/carddisp.pl?gene=ELN |
| COL1A1 | Collagen Type I Alpha 1 Chain | Protein Coding |  | 56 | GC17M050183 | | 6.93 | https://www.genecards.org/cgi-bin/carddisp.pl?gene=COL1A1 |
| SLC6A3 | Solute Carrier Family 6 Member 3 | Protein Coding |  | 56 | GC05M001392 | | 6.9 | https://www.genecards.org/cgi-bin/carddisp.pl?gene=SLC6A3 |
| DNMT1 | DNA Methyltransferase 1 | Protein Coding |  | 57 | GC19M010133 | | 6.86 | https://www.genecards.org/cgi-bin/carddisp.pl?gene=DNMT1 |
| E2F1 | E2F Transcription Factor 1 | Protein Coding |  | 50 | GC20M033675 | | 6.86 | https://www.genecards.org/cgi-bin/carddisp.pl?gene=E2F1 |
| RHOA | Ras Homolog Family Member A | Protein Coding |  | 52 | GC03M049397 | | 6.85 | https://www.genecards.org/cgi-bin/carddisp.pl?gene=RHOA |
| SP110 | SP110 Nuclear Body Protein | Protein Coding |  | 46 | GC02M230167 | | 6.84 | https://www.genecards.org/cgi-bin/carddisp.pl?gene=SP110 |
| TOP1 | DNA Topoisomerase I | Protein Coding |  | 53 | GC20P041028 | | 6.83 | https://www.genecards.org/cgi-bin/carddisp.pl?gene=TOP1 |
| CANX | Calnexin | Protein Coding |  | 51 | GC05P179678 | | 6.83 | https://www.genecards.org/cgi-bin/carddisp.pl?gene=CANX |
| CCL22 | C-C Motif Chemokine Ligand 22 | Protein Coding |  | 43 | GC16P057359 | | 6.81 | https://www.genecards.org/cgi-bin/carddisp.pl?gene=CCL22 |
| RNF8 | Ring Finger Protein 8 | Protein Coding |  | 48 | GC06P043189 | | 6.81 | https://www.genecards.org/cgi-bin/carddisp.pl?gene=RNF8 |
| MDC1 | Mediator Of DNA Damage Checkpoint 1 | Protein Coding |  | 47 | GC06M030714 | | 6.81 | https://www.genecards.org/cgi-bin/carddisp.pl?gene=MDC1 |
| MME | Membrane Metalloendopeptidase | Protein Coding |  | 57 | GC03P155024 | | 6.79 | https://www.genecards.org/cgi-bin/carddisp.pl?gene=MME |
| CDH2 | Cadherin 2 | Protein Coding |  | 56 | GC18M027950 | | 6.79 | https://www.genecards.org/cgi-bin/carddisp.pl?gene=CDH2 |
| FLT1 | Fms Related Tyrosine Kinase 1 | Protein Coding |  | 57 | GC13M028300 | | 6.78 | https://www.genecards.org/cgi-bin/carddisp.pl?gene=FLT1 |
| EGR1 | Early Growth Response 1 | Protein Coding |  | 50 | GC05P138465 | | 6.77 | https://www.genecards.org/cgi-bin/carddisp.pl?gene=EGR1 |
| ILK | Integrin Linked Kinase | Protein Coding |  | 53 | GC11P006581 | | 6.77 | https://www.genecards.org/cgi-bin/carddisp.pl?gene=ILK |
| NR5A1 | Nuclear Receptor Subfamily 5 Group A Member 1 | Protein Coding |  | 56 | GC09M124481 | | 6.77 | https://www.genecards.org/cgi-bin/carddisp.pl?gene=NR5A1 |
| FMR1 | Fragile X Mental Retardation 1 | Protein Coding |  | 51 | GC0XP147912 | | 6.77 | https://www.genecards.org/cgi-bin/carddisp.pl?gene=FMR1 |
| MIR499A | MicroRNA 499a | RNA Gene |  | 21 | GC20P034990 | | 6.77 | https://www.genecards.org/cgi-bin/carddisp.pl?gene=MIR499A |
| MIR377 | MicroRNA 377 | RNA Gene |  | 18 | GC14P103837 | | 6.77 | https://www.genecards.org/cgi-bin/carddisp.pl?gene=MIR377 |
| SLC2A1 | Solute Carrier Family 2 Member 1 | Protein Coding |  | 58 | GC01M042925 | | 6.76 | https://www.genecards.org/cgi-bin/carddisp.pl?gene=SLC2A1 |
| CDK2 | Cyclin Dependent Kinase 2 | Protein Coding |  | 60 | GC12P055966 | | 6.76 | https://www.genecards.org/cgi-bin/carddisp.pl?gene=CDK2 |
| TTN | Titin | Protein Coding |  | 54 | GC02M178525 | | 6.73 | https://www.genecards.org/cgi-bin/carddisp.pl?gene=TTN |
| SERPINC1 | Serpin Family C Member 1 | Protein Coding |  | 54 | GC01M174079 | | 6.72 | https://www.genecards.org/cgi-bin/carddisp.pl?gene=SERPINC1 |
| DDR1 | Discoidin Domain Receptor Tyrosine Kinase 1 | Protein Coding |  | 53 | GC06P032478 | | 6.68 | https://www.genecards.org/cgi-bin/carddisp.pl?gene=DDR1 |
| ERBB3 | Erb-B2 Receptor Tyrosine Kinase 3 | Protein Coding |  | 60 | GC12P056079 | | 6.68 | https://www.genecards.org/cgi-bin/carddisp.pl?gene=ERBB3 |
| CD69 | CD69 Molecule | Protein Coding |  | 46 | GC12M012620 | | 6.66 | https://www.genecards.org/cgi-bin/carddisp.pl?gene=CD69 |
| VTN | Vitronectin | Protein Coding |  | 50 | GC17M029379 | | 6.65 | https://www.genecards.org/cgi-bin/carddisp.pl?gene=VTN |
| RNU4ATAC | RNA, U4atac Small Nuclear (U12-Dependent Splicing) | RNA Gene |  | 23 | GC02P121531 | | 6.65 | https://www.genecards.org/cgi-bin/carddisp.pl?gene=RNU4ATAC |
| NCF1 | Neutrophil Cytosolic Factor 1 | Protein Coding |  | 54 | GC07P074773 | | 6.64 | https://www.genecards.org/cgi-bin/carddisp.pl?gene=NCF1 |
| KRT18 | Keratin 18 | Protein Coding |  | 55 | GC12P052948 | | 6.64 | https://www.genecards.org/cgi-bin/carddisp.pl?gene=KRT18 |
| SPRY4-IT1 | SPRY4 Intronic Transcript 1 | RNA Gene |  | 14 | GC05U901574 | | 6.63 | https://www.genecards.org/cgi-bin/carddisp.pl?gene=SPRY4-IT1 |
| PRKCA | Protein Kinase C Alpha | Protein Coding |  | 57 | GC17P066302 | | 6.61 | https://www.genecards.org/cgi-bin/carddisp.pl?gene=PRKCA |
| TIMP2 | TIMP Metallopeptidase Inhibitor 2 | Protein Coding |  | 50 | GC17M078852 | | 6.53 | https://www.genecards.org/cgi-bin/carddisp.pl?gene=TIMP2 |
| CA9 | Carbonic Anhydrase 9 | Protein Coding |  | 51 | GC09P035673 | | 6.52 | https://www.genecards.org/cgi-bin/carddisp.pl?gene=CA9 |
| DEFB4A | Defensin Beta 4A | Protein Coding |  | 42 | GC08P007895 | | 6.52 | https://www.genecards.org/cgi-bin/carddisp.pl?gene=DEFB4A |
| XRCC5 | X-Ray Repair Cross Complementing 5 | Protein Coding |  | 50 | GC02P216107 | | 6.48 | https://www.genecards.org/cgi-bin/carddisp.pl?gene=XRCC5 |
| CFTR | CF Transmembrane Conductance Regulator | Protein Coding |  | 58 | GC07P117465 | | 6.48 | https://www.genecards.org/cgi-bin/carddisp.pl?gene=CFTR |
| CDKN2B | Cyclin Dependent Kinase Inhibitor 2B | Protein Coding |  | 53 | GC09M021992 | | 6.48 | https://www.genecards.org/cgi-bin/carddisp.pl?gene=CDKN2B |
| GRP | Gastrin Releasing Peptide | Protein Coding |  | 46 | GC18P059220 | | 6.47 | https://www.genecards.org/cgi-bin/carddisp.pl?gene=GRP |
| YWHAE | Tyrosine 3-Monooxygenase/Tryptophan 5-Monooxygenase Activation Protein Epsilon | Protein Coding |  | 56 | GC17M001346 | | 6.45 | https://www.genecards.org/cgi-bin/carddisp.pl?gene=YWHAE |
| DES | Desmin | Protein Coding |  | 54 | GC02P219418 | | 6.42 | https://www.genecards.org/cgi-bin/carddisp.pl?gene=DES |
| IL12RB1 | Interleukin 12 Receptor Subunit Beta 1 | Protein Coding |  | 51 | GC19M018030 | | 6.41 | https://www.genecards.org/cgi-bin/carddisp.pl?gene=IL12RB1 |
| TNFRSF1A | TNF Receptor Superfamily Member 1A | Protein Coding |  | 56 | GC12M006308 | | 6.39 | https://www.genecards.org/cgi-bin/carddisp.pl?gene=TNFRSF1A |
| CASC2 | Cancer Susceptibility 2 | RNA Gene |  | 28 | GC10P118046 | | 6.38 | https://www.genecards.org/cgi-bin/carddisp.pl?gene=CASC2 |
| TSC1 | TSC Complex Subunit 1 | Protein Coding |  | 54 | GC09M132796 | | 6.38 | https://www.genecards.org/cgi-bin/carddisp.pl?gene=TSC1 |
| CDK6 | Cyclin Dependent Kinase 6 | Protein Coding |  | 60 | GC07M092604 | | 6.36 | https://www.genecards.org/cgi-bin/carddisp.pl?gene=CDK6 |
| CYLD | CYLD Lysine 63 Deubiquitinase | Protein Coding |  | 54 | GC16P050742 | | 6.32 | https://www.genecards.org/cgi-bin/carddisp.pl?gene=CYLD |
| SELL | Selectin L | Protein Coding |  | 48 | GC01M169659 | | 6.32 | https://www.genecards.org/cgi-bin/carddisp.pl?gene=SELL |
| NOS3 | Nitric Oxide Synthase 3 | Protein Coding |  | 57 | GC07P150990 | | 6.3 | https://www.genecards.org/cgi-bin/carddisp.pl?gene=NOS3 |
| ABCC1 | ATP Binding Cassette Subfamily C Member 1 | Protein Coding |  | 54 | GC16P015949 | | 6.29 | https://www.genecards.org/cgi-bin/carddisp.pl?gene=ABCC1 |
| AXL | AXL Receptor Tyrosine Kinase | Protein Coding |  | 57 | GC19P041219 | | 6.28 | https://www.genecards.org/cgi-bin/carddisp.pl?gene=AXL |
| ABCG2 | ATP Binding Cassette Subfamily G Member 2 (Junior Blood Group) | Protein Coding |  | 57 | GC04M088090 | | 6.28 | https://www.genecards.org/cgi-bin/carddisp.pl?gene=ABCG2 |
| HIC1 | HIC ZBTB Transcriptional Repressor 1 | Protein Coding |  | 47 | GC17P002054 | | 6.26 | https://www.genecards.org/cgi-bin/carddisp.pl?gene=HIC1 |
| RASSF1 | Ras Association Domain Family Member 1 | Protein Coding |  | 51 | GC03M050369 | | 6.26 | https://www.genecards.org/cgi-bin/carddisp.pl?gene=RASSF1 |
| HOTTIP | HOXA Distal Transcript Antisense RNA | RNA Gene |  | 22 | GC07P027198 | | 6.25 | https://www.genecards.org/cgi-bin/carddisp.pl?gene=HOTTIP |
| CCAT2 | Colon Cancer Associated Transcript 2 | RNA Gene |  | 15 | GC08P127400 | | 6.25 | https://www.genecards.org/cgi-bin/carddisp.pl?gene=CCAT2 |
| VCAM1 | Vascular Cell Adhesion Molecule 1 | Protein Coding |  | 52 | GC01P100719 | | 6.23 | https://www.genecards.org/cgi-bin/carddisp.pl?gene=VCAM1 |
| CYBA | Cytochrome B-245 Alpha Chain | Protein Coding |  | 52 | GC16M088643 | | 6.23 | https://www.genecards.org/cgi-bin/carddisp.pl?gene=CYBA |
| LBP | Lipopolysaccharide Binding Protein | Protein Coding |  | 50 | GC20P038346 | | 6.22 | https://www.genecards.org/cgi-bin/carddisp.pl?gene=LBP |
| HPRT1 | Hypoxanthine Phosphoribosyltransferase 1 | Protein Coding |  | 53 | GC0XP134460 | | 6.22 | https://www.genecards.org/cgi-bin/carddisp.pl?gene=HPRT1 |
| IGFBP5 | Insulin Like Growth Factor Binding Protein 5 | Protein Coding |  | 48 | GC02M216672 | | 6.22 | https://www.genecards.org/cgi-bin/carddisp.pl?gene=IGFBP5 |
| GZMB | Granzyme B | Protein Coding |  | 51 | GC14M024630 | | 6.21 | https://www.genecards.org/cgi-bin/carddisp.pl?gene=GZMB |
| TNFSF13B | TNF Superfamily Member 13b | Protein Coding |  | 51 | GC13P108251 | | 6.2 | https://www.genecards.org/cgi-bin/carddisp.pl?gene=TNFSF13B |
| AFP | Alpha Fetoprotein | Protein Coding |  | 52 | GC04P073431 | | 6.2 | https://www.genecards.org/cgi-bin/carddisp.pl?gene=AFP |
| ENO1 | Enolase 1 | Protein Coding |  | 54 | GC01M008861 | | 6.2 | https://www.genecards.org/cgi-bin/carddisp.pl?gene=ENO1 |
| CD274 | CD274 Molecule | Protein Coding |  | 51 | GC09P005450 | | 6.19 | https://www.genecards.org/cgi-bin/carddisp.pl?gene=CD274 |
| GNRH1 | Gonadotropin Releasing Hormone 1 | Protein Coding |  | 48 | GC08M025419 | | 6.13 | https://www.genecards.org/cgi-bin/carddisp.pl?gene=GNRH1 |
| NPAT | Nuclear Protein, Coactivator Of Histone Transcription | Protein Coding |  | 42 | GC11M108157 | | 6.12 | https://www.genecards.org/cgi-bin/carddisp.pl?gene=NPAT |
| PIK3CG | Phosphatidylinositol-4,5-Bisphosphate 3-Kinase Catalytic Subunit Gamma | Protein Coding |  | 54 | GC07P106865 | | 6.11 | https://www.genecards.org/cgi-bin/carddisp.pl?gene=PIK3CG |
| POMC | Proopiomelanocortin | Protein Coding |  | 54 | GC02M025160 | | 6.1 | https://www.genecards.org/cgi-bin/carddisp.pl?gene=POMC |
| TRPV1 | Transient Receptor Potential Cation Channel Subfamily V Member 1 | Protein Coding |  | 53 | GC17M003565 | | 6.08 | https://www.genecards.org/cgi-bin/carddisp.pl?gene=TRPV1 |
| CD14 | CD14 Molecule | Protein Coding |  | 51 | GC05M140594 | | 6.07 | https://www.genecards.org/cgi-bin/carddisp.pl?gene=CD14 |
| IL6R | Interleukin 6 Receptor | Protein Coding |  | 54 | GC01P154377 | | 6.06 | https://www.genecards.org/cgi-bin/carddisp.pl?gene=IL6R |
| TP73 | Tumor Protein P73 | Protein Coding |  | 52 | GC01P003652 | | 6.05 | https://www.genecards.org/cgi-bin/carddisp.pl?gene=TP73 |
| MAP3K8 | Mitogen-Activated Protein Kinase Kinase Kinase 8 | Protein Coding |  | 54 | GC10P030458 | | 6.04 | https://www.genecards.org/cgi-bin/carddisp.pl?gene=MAP3K8 |
| MXRA5 | Matrix Remodeling Associated 5 | Protein Coding |  | 37 | GC0XM003308 | | 6.04 | https://www.genecards.org/cgi-bin/carddisp.pl?gene=MXRA5 |
| HLA-DQB1 | Major Histocompatibility Complex, Class II, DQ Beta 1 | Protein Coding |  | 50 | GC06M032629 | | 6.03 | https://www.genecards.org/cgi-bin/carddisp.pl?gene=HLA-DQB1 |
| BANCR | BRAF-Activated Non-Protein Coding RNA | RNA Gene |  | 14 | GC09M069296 | | 6.02 | https://www.genecards.org/cgi-bin/carddisp.pl?gene=BANCR |
| ALPL | Alkaline Phosphatase, Biomineralization Associated | Protein Coding |  | 57 | GC01P021508 | | 6.01 | https://www.genecards.org/cgi-bin/carddisp.pl?gene=ALPL |
| HP | Haptoglobin | Protein Coding |  | 50 | GC16P072089 | | 5.95 | https://www.genecards.org/cgi-bin/carddisp.pl?gene=HP |
| CYP2E1 | Cytochrome P450 Family 2 Subfamily E Member 1 | Protein Coding |  | 52 | GC10P133520 | | 5.95 | https://www.genecards.org/cgi-bin/carddisp.pl?gene=CYP2E1 |
| CCL4 | C-C Motif Chemokine Ligand 4 | Protein Coding |  | 47 | GC17P036103 | | 5.91 | https://www.genecards.org/cgi-bin/carddisp.pl?gene=CCL4 |
| PTHLH | Parathyroid Hormone Like Hormone | Protein Coding |  | 52 | GC12M027959 | | 5.9 | https://www.genecards.org/cgi-bin/carddisp.pl?gene=PTHLH |
| CADM1 | Cell Adhesion Molecule 1 | Protein Coding |  | 48 | GC11M115169 | | 5.9 | https://www.genecards.org/cgi-bin/carddisp.pl?gene=CADM1 |
| AKT2 | AKT Serine/Threonine Kinase 2 | Protein Coding |  | 60 | GC19M040230 | | 5.9 | https://www.genecards.org/cgi-bin/carddisp.pl?gene=AKT2 |
| IGFBP3 | Insulin Like Growth Factor Binding Protein 3 | Protein Coding |  | 51 | GC07M045912 | | 5.89 | https://www.genecards.org/cgi-bin/carddisp.pl?gene=IGFBP3 |
| BAK1 | BCL2 Antagonist/Killer 1 | Protein Coding |  | 51 | GC06M033572 | | 5.86 | https://www.genecards.org/cgi-bin/carddisp.pl?gene=BAK1 |
| LPAR1 | Lysophosphatidic Acid Receptor 1 | Protein Coding |  | 52 | GC09M110873 | | 5.85 | https://www.genecards.org/cgi-bin/carddisp.pl?gene=LPAR1 |
| PTK2 | Protein Tyrosine Kinase 2 | Protein Coding |  | 54 | GC08M140657 | | 5.83 | https://www.genecards.org/cgi-bin/carddisp.pl?gene=PTK2 |
| HNF1A-AS1 | HNF1A Antisense RNA 1 | RNA Gene |  | 18 | GC12M120987 | | 5.83 | https://www.genecards.org/cgi-bin/carddisp.pl?gene=HNF1A-AS1 |
| SNHG16 | Small Nucleolar RNA Host Gene 16 | RNA Gene |  | 17 | GC17P076559 | | 5.83 | https://www.genecards.org/cgi-bin/carddisp.pl?gene=SNHG16 |
| AFAP1-AS1 | AFAP1 Antisense RNA 1 | RNA Gene |  | 16 | GC04P007756 | | 5.83 | https://www.genecards.org/cgi-bin/carddisp.pl?gene=AFAP1-AS1 |
| HAGLR | HOXD Antisense Growth-Associated Long Non-Coding RNA | RNA Gene |  | 15 | GC02M176173 | | 5.83 | https://www.genecards.org/cgi-bin/carddisp.pl?gene=HAGLR |
| PANDAR | Promoter Of CDKN1A Antisense DNA Damage Activated RNA | RNA Gene |  | 14 | GC06M036673 | | 5.83 | https://www.genecards.org/cgi-bin/carddisp.pl?gene=PANDAR |
| PTCH1 | Patched 1 | Protein Coding |  | 57 | GC09M095442 | | 5.82 | https://www.genecards.org/cgi-bin/carddisp.pl?gene=PTCH1 |
| IGHE | Immunoglobulin Heavy Constant Epsilon | Protein Coding |  | 31 | GC14M105764 | | 5.82 | https://www.genecards.org/cgi-bin/carddisp.pl?gene=IGHE |
| CCR4 | C-C Motif Chemokine Receptor 4 | Protein Coding |  | 51 | GC03P032968 | | 5.81 | https://www.genecards.org/cgi-bin/carddisp.pl?gene=CCR4 |
| CCND2 | Cyclin D2 | Protein Coding |  | 56 | GC12P006034 | | 5.77 | https://www.genecards.org/cgi-bin/carddisp.pl?gene=CCND2 |
| CFLAR | CASP8 And FADD Like Apoptosis Regulator | Protein Coding |  | 53 | GC02P201117 | | 5.75 | https://www.genecards.org/cgi-bin/carddisp.pl?gene=CFLAR |
| NRG1 | Neuregulin 1 | Protein Coding |  | 52 | GC08P031617 | | 5.75 | https://www.genecards.org/cgi-bin/carddisp.pl?gene=NRG1 |
| ESR2 | Estrogen Receptor 2 | Protein Coding |  | 56 | GC14M064084 | | 5.75 | https://www.genecards.org/cgi-bin/carddisp.pl?gene=ESR2 |
| HTRA1 | HtrA Serine Peptidase 1 | Protein Coding |  | 50 | GC10P122461 | | 5.74 | https://www.genecards.org/cgi-bin/carddisp.pl?gene=HTRA1 |
| THPO | Thrombopoietin | Protein Coding |  | 49 | GC03M184371 | | 5.72 | https://www.genecards.org/cgi-bin/carddisp.pl?gene=THPO |
| CXCL5 | C-X-C Motif Chemokine Ligand 5 | Protein Coding |  | 46 | GC04M073995 | | 5.71 | https://www.genecards.org/cgi-bin/carddisp.pl?gene=CXCL5 |
| DPYD | Dihydropyrimidine Dehydrogenase | Protein Coding |  | 59 | GC01M097015 | | 5.71 | https://www.genecards.org/cgi-bin/carddisp.pl?gene=DPYD |
| MSMB | Microseminoprotein Beta | Protein Coding |  | 46 | GC10M046033 | | 5.7 | https://www.genecards.org/cgi-bin/carddisp.pl?gene=MSMB |
| EPCAM | Epithelial Cell Adhesion Molecule | Protein Coding |  | 53 | GC02P047345 | | 5.68 | https://www.genecards.org/cgi-bin/carddisp.pl?gene=EPCAM |
| KCNQ1OT1 | KCNQ1 Opposite Strand/Antisense Transcript 1 | RNA Gene |  | 28 | GC11M002661 | | 5.68 | https://www.genecards.org/cgi-bin/carddisp.pl?gene=KCNQ1OT1 |
| HOXA11-AS | HOXA11 Antisense RNA | RNA Gene |  | 20 | GC07P027184 | | 5.68 | https://www.genecards.org/cgi-bin/carddisp.pl?gene=HOXA11-AS |
| LNCRNA-ATB | Long Noncoding RNA Activated By TGF-Beta | RNA Gene |  | 4 | GC14U902188 | | 5.68 | https://www.genecards.org/cgi-bin/carddisp.pl?gene=LNCRNA-ATB |
| TWIST1 | Twist Family BHLH Transcription Factor 1 | Protein Coding |  | 52 | GC07M019020 | | 5.67 | https://www.genecards.org/cgi-bin/carddisp.pl?gene=TWIST1 |
| NAPSA | Napsin A Aspartic Peptidase | Protein Coding |  | 47 | GC19M050378 | | 5.67 | https://www.genecards.org/cgi-bin/carddisp.pl?gene=NAPSA |
| MIR345 | MicroRNA 345 | RNA Gene |  | 20 | GC14P100307 | | 5.66 | https://www.genecards.org/cgi-bin/carddisp.pl?gene=MIR345 |
| CD27 | CD27 Molecule | Protein Coding |  | 51 | GC12P006425 | | 5.65 | https://www.genecards.org/cgi-bin/carddisp.pl?gene=CD27 |
| NOS2 | Nitric Oxide Synthase 2 | Protein Coding |  | 56 | GC17M027756 | | 5.65 | https://www.genecards.org/cgi-bin/carddisp.pl?gene=NOS2 |
| IL4R | Interleukin 4 Receptor | Protein Coding |  | 54 | GC16P027325 | | 5.64 | https://www.genecards.org/cgi-bin/carddisp.pl?gene=IL4R |
| MB | Myoglobin | Protein Coding |  | 50 | GC22M035606 | | 5.64 | https://www.genecards.org/cgi-bin/carddisp.pl?gene=MB |
| MIR99A | MicroRNA 99a | RNA Gene |  | 21 | GC21P016539 | | 5.63 | https://www.genecards.org/cgi-bin/carddisp.pl?gene=MIR99A |
| BDNF | Brain Derived Neurotrophic Factor | Protein Coding |  | 53 | GC11M027654 | | 5.62 | https://www.genecards.org/cgi-bin/carddisp.pl?gene=BDNF |
| TUSC7 | Tumor Suppressor Candidate 7 | RNA Gene |  | 16 | GC03P116709 | | 5.62 | https://www.genecards.org/cgi-bin/carddisp.pl?gene=TUSC7 |
| CTSB | Cathepsin B | Protein Coding |  | 57 | GC08M011842 | | 5.6 | https://www.genecards.org/cgi-bin/carddisp.pl?gene=CTSB |
| ANGPT2 | Angiopoietin 2 | Protein Coding |  | 50 | GC08M006499 | | 5.6 | https://www.genecards.org/cgi-bin/carddisp.pl?gene=ANGPT2 |
| PTK2B | Protein Tyrosine Kinase 2 Beta | Protein Coding |  | 55 | GC08P027311 | | 5.57 | https://www.genecards.org/cgi-bin/carddisp.pl?gene=PTK2B |
| MECP2 | Methyl-CpG Binding Protein 2 | Protein Coding |  | 52 | GC0XM154021 | | 5.57 | https://www.genecards.org/cgi-bin/carddisp.pl?gene=MECP2 |
| SNHG1 | Small Nucleolar RNA Host Gene 1 | RNA Gene |  | 22 | GC11M062992 | | 5.56 | https://www.genecards.org/cgi-bin/carddisp.pl?gene=SNHG1 |
| TLR6 | Toll Like Receptor 6 | Protein Coding |  | 50 | GC04M038828 | | 5.56 | https://www.genecards.org/cgi-bin/carddisp.pl?gene=TLR6 |
| MIR23A | MicroRNA 23a | RNA Gene |  | 21 | GC19M013954 | | 5.56 | https://www.genecards.org/cgi-bin/carddisp.pl?gene=MIR23A |
| TDP1 | Tyrosyl-DNA Phosphodiesterase 1 | Protein Coding |  | 51 | GC14P089954 | | 5.55 | https://www.genecards.org/cgi-bin/carddisp.pl?gene=TDP1 |
| VDR | Vitamin D Receptor | Protein Coding |  | 57 | GC12M047841 | | 5.54 | https://www.genecards.org/cgi-bin/carddisp.pl?gene=VDR |
| WT1 | WT1 Transcription Factor | Protein Coding |  | 54 | GC11M032365 | | 5.54 | https://www.genecards.org/cgi-bin/carddisp.pl?gene=WT1 |
| BCYRN1 | Brain Cytoplasmic RNA 1 | RNA Gene |  | 18 | GC02P047331 | | 5.53 | https://www.genecards.org/cgi-bin/carddisp.pl?gene=BCYRN1 |
| TXN | Thioredoxin | Protein Coding |  | 52 | GC09M110243 | | 5.52 | https://www.genecards.org/cgi-bin/carddisp.pl?gene=TXN |
| KLK3 | Kallikrein Related Peptidase 3 | Protein Coding |  | 53 | GC19P050854 | | 5.52 | https://www.genecards.org/cgi-bin/carddisp.pl?gene=KLK3 |
| TIRAP | TIR Domain Containing Adaptor Protein | Protein Coding |  | 47 | GC11P126282 | | 5.52 | https://www.genecards.org/cgi-bin/carddisp.pl?gene=TIRAP |
| VEGFD | Vascular Endothelial Growth Factor D | Protein Coding |  | 39 | GC0XM015345 | | 5.52 | https://www.genecards.org/cgi-bin/carddisp.pl?gene=VEGFD |
| ZFAS1 | ZNFX1 Antisense RNA 1 | RNA Gene |  | 20 | GC20P049333 | | 5.52 | https://www.genecards.org/cgi-bin/carddisp.pl?gene=ZFAS1 |
| VIM | Vimentin | Protein Coding |  | 56 | GC10P017227 | | 5.51 | https://www.genecards.org/cgi-bin/carddisp.pl?gene=VIM |
| ERBB4 | Erb-B2 Receptor Tyrosine Kinase 4 | Protein Coding |  | 62 | GC02M211375 | | 5.51 | https://www.genecards.org/cgi-bin/carddisp.pl?gene=ERBB4 |
| BCL2L11 | BCL2 Like 11 | Protein Coding |  | 51 | GC02P111119 | | 5.5 | https://www.genecards.org/cgi-bin/carddisp.pl?gene=BCL2L11 |
| PRDM1 | PR/SET Domain 1 | Protein Coding |  | 51 | GC06P106086 | | 5.49 | https://www.genecards.org/cgi-bin/carddisp.pl?gene=PRDM1 |
| HSPB1 | Heat Shock Protein Family B (Small) Member 1 | Protein Coding |  | 57 | GC07P076302 | | 5.48 | https://www.genecards.org/cgi-bin/carddisp.pl?gene=HSPB1 |
| MEIG1 | Meiosis/Spermiogenesis Associated 1 | Protein Coding |  | 34 | GC10P014954 | | 5.46 | https://www.genecards.org/cgi-bin/carddisp.pl?gene=MEIG1 |
| TP73-AS1 | TP73 Antisense RNA 1 | RNA Gene |  | 25 | GC01M003735 | | 5.46 | https://www.genecards.org/cgi-bin/carddisp.pl?gene=TP73-AS1 |
| GSTT1 | Glutathione S-Transferase Theta 1 | Protein Coding |  | 39 | GC22Mi00270 | | 5.45 | https://www.genecards.org/cgi-bin/carddisp.pl?gene=GSTT1 |
| SNCA | Synuclein Alpha | Protein Coding |  | 56 | GC04M089724 | | 5.44 | https://www.genecards.org/cgi-bin/carddisp.pl?gene=SNCA |
| SLC17A5 | Solute Carrier Family 17 Member 5 | Protein Coding |  | 50 | GC06M073593 | | 5.44 | https://www.genecards.org/cgi-bin/carddisp.pl?gene=SLC17A5 |
| C3 | Complement C3 | Protein Coding |  | 54 | GC19M006677 | | 5.42 | https://www.genecards.org/cgi-bin/carddisp.pl?gene=C3 |
| CCR6 | C-C Motif Chemokine Receptor 6 | Protein Coding |  | 51 | GC06P167111 | | 5.41 | https://www.genecards.org/cgi-bin/carddisp.pl?gene=CCR6 |
| FOLH1 | Folate Hydrolase 1 | Protein Coding |  | 53 | GC11M056090 | | 5.38 | https://www.genecards.org/cgi-bin/carddisp.pl?gene=FOLH1 |
| BIRC2 | Baculoviral IAP Repeat Containing 2 | Protein Coding |  | 52 | GC11P102347 | | 5.37 | https://www.genecards.org/cgi-bin/carddisp.pl?gene=BIRC2 |
| KRT13 | Keratin 13 | Protein Coding |  | 49 | GC17M041500 | | 5.37 | https://www.genecards.org/cgi-bin/carddisp.pl?gene=KRT13 |
| MIAT | Myocardial Infarction Associated Transcript | RNA Gene |  | 25 | GC22P026646 | | 5.36 | https://www.genecards.org/cgi-bin/carddisp.pl?gene=MIAT |
| SNHG12 | Small Nucleolar RNA Host Gene 12 | RNA Gene |  | 24 | GC01M028578 | | 5.36 | https://www.genecards.org/cgi-bin/carddisp.pl?gene=SNHG12 |
| SOX2-OT | SOX2 Overlapping Transcript | RNA Gene |  | 22 | GC03P180989 | | 5.36 | https://www.genecards.org/cgi-bin/carddisp.pl?gene=SOX2-OT |
| TP53COR1 | Tumor Protein P53 Pathway Corepressor 1 | RNA Gene |  | 10 | GC06U903133 | | 5.36 | https://www.genecards.org/cgi-bin/carddisp.pl?gene=TP53COR1 |
| RUNX1 | RUNX Family Transcription Factor 1 | Protein Coding |  | 55 | GC21M034787 | | 5.35 | https://www.genecards.org/cgi-bin/carddisp.pl?gene=RUNX1 |
| FAM189A1 | Family With Sequence Similarity 189 Member A1 | Protein Coding |  | 36 | GC15M029120 | | 5.34 | https://www.genecards.org/cgi-bin/carddisp.pl?gene=FAM189A1 |
| F3 | Coagulation Factor III, Tissue Factor | Protein Coding |  | 51 | GC01M094466 | | 5.33 | https://www.genecards.org/cgi-bin/carddisp.pl?gene=F3 |
| CREB1 | CAMP Responsive Element Binding Protein 1 | Protein Coding |  | 54 | GC02P207529 | | 5.33 | https://www.genecards.org/cgi-bin/carddisp.pl?gene=CREB1 |
| FOS | Fos Proto-Oncogene, AP-1 Transcription Factor Subunit | Protein Coding |  | 57 | GC14P075278 | | 5.32 | https://www.genecards.org/cgi-bin/carddisp.pl?gene=FOS |
| ITPA | Inosine Triphosphatase | Protein Coding |  | 53 | GC20P003189 | | 5.3 | https://www.genecards.org/cgi-bin/carddisp.pl?gene=ITPA |
| ERCC4 | ERCC Excision Repair 4, Endonuclease Catalytic Subunit | Protein Coding |  | 51 | GC16P014014 | | 5.29 | https://www.genecards.org/cgi-bin/carddisp.pl?gene=ERCC4 |
| NCF2 | Neutrophil Cytosolic Factor 2 | Protein Coding |  | 53 | GC01M183524 | | 5.28 | https://www.genecards.org/cgi-bin/carddisp.pl?gene=NCF2 |
| STAT5A | Signal Transducer And Activator Of Transcription 5A | Protein Coding |  | 52 | GC17P042287 | | 5.26 | https://www.genecards.org/cgi-bin/carddisp.pl?gene=STAT5A |
| KLRK1 | Killer Cell Lectin Like Receptor K1 | Protein Coding |  | 46 | GC12M012632 | | 5.25 | https://www.genecards.org/cgi-bin/carddisp.pl?gene=KLRK1 |
| PPBP | Pro-Platelet Basic Protein | Protein Coding |  | 49 | GC04M073986 | | 5.25 | https://www.genecards.org/cgi-bin/carddisp.pl?gene=PPBP |
| TRIM13 | Tripartite Motif Containing 13 | Protein Coding |  | 42 | GC13P049995 | | 5.24 | https://www.genecards.org/cgi-bin/carddisp.pl?gene=TRIM13 |
| APOE | Apolipoprotein E | Protein Coding |  | 57 | GC19P044906 | | 5.24 | https://www.genecards.org/cgi-bin/carddisp.pl?gene=APOE |
| APP | Amyloid Beta Precursor Protein | Protein Coding |  | 57 | GC21M025880 | | 5.24 | https://www.genecards.org/cgi-bin/carddisp.pl?gene=APP |
| EFNA5 | Ephrin A5 | Protein Coding |  | 50 | GC05M107376 | | 5.23 | https://www.genecards.org/cgi-bin/carddisp.pl?gene=EFNA5 |
| TH | Tyrosine Hydroxylase | Protein Coding |  | 58 | GC11M002163 | | 5.23 | https://www.genecards.org/cgi-bin/carddisp.pl?gene=TH |
| TKT | Transketolase | Protein Coding |  | 54 | GC03M053258 | | 5.22 | https://www.genecards.org/cgi-bin/carddisp.pl?gene=TKT |
| IL11 | Interleukin 11 | Protein Coding |  | 47 | GC19M055364 | | 5.21 | https://www.genecards.org/cgi-bin/carddisp.pl?gene=IL11 |
| AICDA | Activation Induced Cytidine Deaminase | Protein Coding |  | 53 | GC12M008602 | | 5.21 | https://www.genecards.org/cgi-bin/carddisp.pl?gene=AICDA |
| ASCL1 | Achaete-Scute Family BHLH Transcription Factor 1 | Protein Coding |  | 49 | GC12P102957 | | 5.19 | https://www.genecards.org/cgi-bin/carddisp.pl?gene=ASCL1 |
| DANCR | Differentiation Antagonizing Non-Protein Coding RNA | RNA Gene |  | 21 | GC04P052712 | | 5.19 | https://www.genecards.org/cgi-bin/carddisp.pl?gene=DANCR |
| BLACAT1 | Bladder Cancer Associated Transcript 1 | RNA Gene |  | 15 | GC01M205434 | | 5.19 | https://www.genecards.org/cgi-bin/carddisp.pl?gene=BLACAT1 |
| GHET1 | Gastric Carcinoma Proliferation Enhancing Transcript 1 | RNA Gene |  | 13 | GC07P149020 | | 5.19 | https://www.genecards.org/cgi-bin/carddisp.pl?gene=GHET1 |
| TNFRSF11B | TNF Receptor Superfamily Member 11b | Protein Coding |  | 54 | GC08M118923 | | 5.19 | https://www.genecards.org/cgi-bin/carddisp.pl?gene=TNFRSF11B |
| MMP12 | Matrix Metallopeptidase 12 | Protein Coding |  | 50 | GC11M102862 | | 5.18 | https://www.genecards.org/cgi-bin/carddisp.pl?gene=MMP12 |
| CD28 | CD28 Molecule | Protein Coding |  | 53 | GC02P203706 | | 5.18 | https://www.genecards.org/cgi-bin/carddisp.pl?gene=CD28 |
| THBS1 | Thrombospondin 1 | Protein Coding |  | 51 | GC15P039581 | | 5.16 | https://www.genecards.org/cgi-bin/carddisp.pl?gene=THBS1 |
| PTAFR | Platelet Activating Factor Receptor | Protein Coding |  | 48 | GC01M028161 | | 5.15 | https://www.genecards.org/cgi-bin/carddisp.pl?gene=PTAFR |
| MIF | Macrophage Migration Inhibitory Factor | Protein Coding |  | 55 | GC22P023894 | | 5.14 | https://www.genecards.org/cgi-bin/carddisp.pl?gene=MIF |
| IRF7 | Interferon Regulatory Factor 7 | Protein Coding |  | 53 | GC11M000612 | | 5.13 | https://www.genecards.org/cgi-bin/carddisp.pl?gene=IRF7 |
| HIST2H2AA3 | Histone Cluster 2 H2A Family Member A3 | Protein Coding |  | 40 | GC01M149848 | | 5.12 | https://www.genecards.org/cgi-bin/carddisp.pl?gene=HIST2H2AA3 |
| CPLANE1 | Ciliogenesis And Planar Polarity Effector 1 | Protein Coding |  | 33 | GC05M037065 | | 5.1 | https://www.genecards.org/cgi-bin/carddisp.pl?gene=CPLANE1 |
| TUBB | Tubulin Beta Class I | Protein Coding |  | 56 | GC06P030720 | | 5.09 | https://www.genecards.org/cgi-bin/carddisp.pl?gene=TUBB |
| CCND3 | Cyclin D3 | Protein Coding |  | 54 | GC06M041934 | | 5.08 | https://www.genecards.org/cgi-bin/carddisp.pl?gene=CCND3 |
| EPHX1 | Epoxide Hydrolase 1 | Protein Coding |  | 53 | GC01P225810 | | 5.07 | https://www.genecards.org/cgi-bin/carddisp.pl?gene=EPHX1 |
| HLA-A | Major Histocompatibility Complex, Class I, A | Protein Coding |  | 53 | GC06P032430 | | 5.07 | https://www.genecards.org/cgi-bin/carddisp.pl?gene=HLA-A |
| UBE2N | Ubiquitin Conjugating Enzyme E2 N | Protein Coding |  | 54 | GC12M093423 | | 5.07 | https://www.genecards.org/cgi-bin/carddisp.pl?gene=UBE2N |
| CXCR1 | C-X-C Motif Chemokine Receptor 1 | Protein Coding |  | 49 | GC02M218162 | | 5.06 | https://www.genecards.org/cgi-bin/carddisp.pl?gene=CXCR1 |
| DUOX1 | Dual Oxidase 1 | Protein Coding |  | 47 | GC15P045129 | | 5.06 | https://www.genecards.org/cgi-bin/carddisp.pl?gene=DUOX1 |
| CXCR5 | C-X-C Motif Chemokine Receptor 5 | Protein Coding |  | 47 | GC11P118884 | | 5.06 | https://www.genecards.org/cgi-bin/carddisp.pl?gene=CXCR5 |
| PIM2 | Pim-2 Proto-Oncogene, Serine/Threonine Kinase | Protein Coding |  | 51 | GC0XM048913 | | 5.03 | https://www.genecards.org/cgi-bin/carddisp.pl?gene=PIM2 |
| APAF1 | Apoptotic Peptidase Activating Factor 1 | Protein Coding |  | 53 | GC12P098645 | | 5.03 | https://www.genecards.org/cgi-bin/carddisp.pl?gene=APAF1 |
| MMP8 | Matrix Metallopeptidase 8 | Protein Coding |  | 53 | GC11M102617 | | 5.02 | https://www.genecards.org/cgi-bin/carddisp.pl?gene=MMP8 |
| LUCAT1 | Lung Cancer Associated Transcript 1 | RNA Gene |  | 17 | GC05M091054 | | 5.01 | https://www.genecards.org/cgi-bin/carddisp.pl?gene=LUCAT1 |
| SETD2 | SET Domain Containing 2, Histone Lysine Methyltransferase | Protein Coding |  | 53 | GC03M047033 | | 5.01 | https://www.genecards.org/cgi-bin/carddisp.pl?gene=SETD2 |
| SERPINB3 | Serpin Family B Member 3 | Protein Coding |  | 48 | GC18M063638 | | 5 | https://www.genecards.org/cgi-bin/carddisp.pl?gene=SERPINB3 |
| DAPK1 | Death Associated Protein Kinase 1 | Protein Coding |  | 54 | GC09P087497 | | 5 | https://www.genecards.org/cgi-bin/carddisp.pl?gene=DAPK1 |
| DPP9 | Dipeptidyl Peptidase 9 | Protein Coding |  | 47 | GC19M004675 | | 4.99 | https://www.genecards.org/cgi-bin/carddisp.pl?gene=DPP9 |
| BAD | BCL2 Associated Agonist Of Cell Death | Protein Coding |  | 53 | GC11M064288 | | 4.97 | https://www.genecards.org/cgi-bin/carddisp.pl?gene=BAD |
| ATP11A | ATPase Phospholipid Transporting 11A | Protein Coding |  | 48 | GC13P112690 | | 4.97 | https://www.genecards.org/cgi-bin/carddisp.pl?gene=ATP11A |
| CAMP | Cathelicidin Antimicrobial Peptide | Protein Coding |  | 47 | GC03P048227 | | 4.96 | https://www.genecards.org/cgi-bin/carddisp.pl?gene=CAMP |
| ATF3 | Activating Transcription Factor 3 | Protein Coding |  | 50 | GC01P212565 | | 4.96 | https://www.genecards.org/cgi-bin/carddisp.pl?gene=ATF3 |
| CLU | Clusterin | Protein Coding |  | 53 | GC08M027596 | | 4.95 | https://www.genecards.org/cgi-bin/carddisp.pl?gene=CLU |
| IL24 | Interleukin 24 | Protein Coding |  | 48 | GC01P206897 | | 4.94 | https://www.genecards.org/cgi-bin/carddisp.pl?gene=IL24 |
| SERPINB1 | Serpin Family B Member 1 | Protein Coding |  | 46 | GC06M002833 | | 4.93 | https://www.genecards.org/cgi-bin/carddisp.pl?gene=SERPINB1 |
| MMP14 | Matrix Metallopeptidase 14 | Protein Coding |  | 57 | GC14P024599 | | 4.93 | https://www.genecards.org/cgi-bin/carddisp.pl?gene=MMP14 |
| VIP | Vasoactive Intestinal Peptide | Protein Coding |  | 50 | GC06P152750 | | 4.93 | https://www.genecards.org/cgi-bin/carddisp.pl?gene=VIP |
| CXCR2 | C-X-C Motif Chemokine Receptor 2 | Protein Coding |  | 54 | GC02P218125 | | 4.93 | https://www.genecards.org/cgi-bin/carddisp.pl?gene=CXCR2 |
| LGALS3 | Galectin 3 | Protein Coding |  | 51 | GC14P055124 | | 4.91 | https://www.genecards.org/cgi-bin/carddisp.pl?gene=LGALS3 |
| CFI | Complement Factor I | Protein Coding |  | 52 | GC04M109740 | | 4.91 | https://www.genecards.org/cgi-bin/carddisp.pl?gene=CFI |
| RALBP1 | RalA Binding Protein 1 | Protein Coding |  | 51 | GC18P009465 | | 4.9 | https://www.genecards.org/cgi-bin/carddisp.pl?gene=RALBP1 |
| GGT1 | Gamma-Glutamyltransferase 1 | Protein Coding |  | 54 | GC22P024583 | | 4.89 | https://www.genecards.org/cgi-bin/carddisp.pl?gene=GGT1 |
| BGLAP | Bone Gamma-Carboxyglutamate Protein | Protein Coding |  | 46 | GC01P156211 | | 4.89 | https://www.genecards.org/cgi-bin/carddisp.pl?gene=BGLAP |
| BPIFA1 | BPI Fold Containing Family A Member 1 | Protein Coding |  | 42 | GC20P033235 | | 4.88 | https://www.genecards.org/cgi-bin/carddisp.pl?gene=BPIFA1 |
| IL2RB | Interleukin 2 Receptor Subunit Beta | Protein Coding |  | 55 | GC22M037125 | | 4.87 | https://www.genecards.org/cgi-bin/carddisp.pl?gene=IL2RB |
| TINCR | TINCR Ubiquitin Domain Containing | Protein Coding |  | 26 | GC19M005558 | | 4.83 | https://www.genecards.org/cgi-bin/carddisp.pl?gene=TINCR |
| MIR27B | MicroRNA 27b | RNA Gene |  | 21 | GC09P095102 | | 4.83 | https://www.genecards.org/cgi-bin/carddisp.pl?gene=MIR27B |
| FENDRR | FOXF1 Adjacent Non-Coding Developmental Regulatory RNA | RNA Gene |  | 20 | GC16M086511 | | 4.83 | https://www.genecards.org/cgi-bin/carddisp.pl?gene=FENDRR |
| LINC00261 | Long Intergenic Non-Protein Coding RNA 261 | RNA Gene |  | 19 | GC20M022529 | | 4.83 | https://www.genecards.org/cgi-bin/carddisp.pl?gene=LINC00261 |
| BCAR4 | Breast Cancer Anti-Estrogen Resistance 4 | RNA Gene |  | 19 | GC16M011819 | | 4.83 | https://www.genecards.org/cgi-bin/carddisp.pl?gene=BCAR4 |
| MIR29B2 | MicroRNA 29b-2 | RNA Gene |  | 17 | GC01M207810 | | 4.83 | https://www.genecards.org/cgi-bin/carddisp.pl?gene=MIR29B2 |
| RNY1 | RNA, Ro60-Associated Y1 | RNA Gene |  | 17 | GC07M148987 | | 4.83 | https://www.genecards.org/cgi-bin/carddisp.pl?gene=RNY1 |
| RNY3 | RNA, Ro60-Associated Y3 | RNA Gene |  | 16 | GC07P149019 | | 4.83 | https://www.genecards.org/cgi-bin/carddisp.pl?gene=RNY3 |
| FCGR2A | Fc Fragment Of IgG Receptor IIa | Protein Coding |  | 52 | GC01P161505 | | 4.83 | https://www.genecards.org/cgi-bin/carddisp.pl?gene=FCGR2A |
| MIR139 | MicroRNA 139 | RNA Gene |  | 20 | GC11M072615 | | 4.78 | https://www.genecards.org/cgi-bin/carddisp.pl?gene=MIR139 |
| HMGA2 | High Mobility Group AT-Hook 2 | Protein Coding |  | 51 | GC12P065824 | | 4.77 | https://www.genecards.org/cgi-bin/carddisp.pl?gene=HMGA2 |
| HSF4 | Heat Shock Transcription Factor 4 | Protein Coding |  | 48 | GC16P067197 | | 4.76 | https://www.genecards.org/cgi-bin/carddisp.pl?gene=HSF4 |
| MKI67 | Marker Of Proliferation Ki-67 | Protein Coding |  | 48 | GC10M128096 | | 4.76 | https://www.genecards.org/cgi-bin/carddisp.pl?gene=MKI67 |
| CDC25C | Cell Division Cycle 25C | Protein Coding |  | 54 | GC05M138296 | | 4.74 | https://www.genecards.org/cgi-bin/carddisp.pl?gene=CDC25C |
| HERC2 | HECT And RLD Domain Containing E3 Ubiquitin Protein Ligase 2 | Protein Coding |  | 51 | GC15M028111 | | 4.72 | https://www.genecards.org/cgi-bin/carddisp.pl?gene=HERC2 |
| TNFRSF17 | TNF Receptor Superfamily Member 17 | Protein Coding |  | 50 | GC16P012058 | | 4.72 | https://www.genecards.org/cgi-bin/carddisp.pl?gene=TNFRSF17 |
| IGF2 | Insulin Like Growth Factor 2 | Protein Coding |  | 54 | GC11M002130 | | 4.71 | https://www.genecards.org/cgi-bin/carddisp.pl?gene=IGF2 |
| TNFRSF10A | TNF Receptor Superfamily Member 10a | Protein Coding |  | 51 | GC08M023190 | | 4.68 | https://www.genecards.org/cgi-bin/carddisp.pl?gene=TNFRSF10A |
| SKP2 | S-Phase Kinase Associated Protein 2 | Protein Coding |  | 51 | GC05P036103 | | 4.67 | https://www.genecards.org/cgi-bin/carddisp.pl?gene=SKP2 |
| CTTN | Cortactin | Protein Coding |  | 48 | GC11P070398 | | 4.67 | https://www.genecards.org/cgi-bin/carddisp.pl?gene=CTTN |
| SLPI | Secretory Leukocyte Peptidase Inhibitor | Protein Coding |  | 45 | GC20M045252 | | 4.66 | https://www.genecards.org/cgi-bin/carddisp.pl?gene=SLPI |
| RELA | RELA Proto-Oncogene, NF-KB Subunit | Protein Coding |  | 56 | GC11M065671 | | 4.65 | https://www.genecards.org/cgi-bin/carddisp.pl?gene=RELA |
| SELP | Selectin P | Protein Coding |  | 52 | GC01M169558 | | 4.64 | https://www.genecards.org/cgi-bin/carddisp.pl?gene=SELP |
| GSK3B | Glycogen Synthase Kinase 3 Beta | Protein Coding |  | 57 | GC03M119821 | | 4.64 | https://www.genecards.org/cgi-bin/carddisp.pl?gene=GSK3B |
| CD8A | CD8a Molecule | Protein Coding |  | 53 | GC02M086784 | | 4.64 | https://www.genecards.org/cgi-bin/carddisp.pl?gene=CD8A |
| EPB41L3 | Erythrocyte Membrane Protein Band 4.1 Like 3 | Protein Coding |  | 48 | GC18M005382 | | 4.63 | https://www.genecards.org/cgi-bin/carddisp.pl?gene=EPB41L3 |
| MIR31HG | MIR31 Host Gene | RNA Gene |  | 18 | GC09M021455 | | 4.63 | https://www.genecards.org/cgi-bin/carddisp.pl?gene=MIR31HG |
| SNHG15 | Small Nucleolar RNA Host Gene 15 | RNA Gene |  | 18 | GC07M044983 | | 4.63 | https://www.genecards.org/cgi-bin/carddisp.pl?gene=SNHG15 |
| SNHG20 | Small Nucleolar RNA Host Gene 20 | RNA Gene |  | 17 | GC17P077087 | | 4.63 | https://www.genecards.org/cgi-bin/carddisp.pl?gene=SNHG20 |
| IRAIN | IGF1R Antisense Imprinted Non-Protein Coding RNA | RNA Gene |  | 14 | GC15M098645 | | 4.63 | https://www.genecards.org/cgi-bin/carddisp.pl?gene=IRAIN |
| LTA | Lymphotoxin Alpha | Protein Coding |  | 48 | GC06P032497 | | 4.63 | https://www.genecards.org/cgi-bin/carddisp.pl?gene=LTA |
| MSH6 | MutS Homolog 6 | Protein Coding |  | 55 | GC02P047695 | | 4.62 | https://www.genecards.org/cgi-bin/carddisp.pl?gene=MSH6 |
| POLB | DNA Polymerase Beta | Protein Coding |  | 53 | GC08P042315 | | 4.62 | https://www.genecards.org/cgi-bin/carddisp.pl?gene=POLB |
| MVP | Major Vault Protein | Protein Coding |  | 45 | GC16P029995 | | 4.59 | https://www.genecards.org/cgi-bin/carddisp.pl?gene=MVP |
| EPB41 | Erythrocyte Membrane Protein Band 4.1 | Protein Coding |  | 51 | GC01P028887 | | 4.57 | https://www.genecards.org/cgi-bin/carddisp.pl?gene=EPB41 |
| CCR3 | C-C Motif Chemokine Receptor 3 | Protein Coding |  | 53 | GC03P046227 | | 4.54 | https://www.genecards.org/cgi-bin/carddisp.pl?gene=CCR3 |
| TK2 | Thymidine Kinase 2 | Protein Coding |  | 50 | GC16M066542 | | 4.53 | https://www.genecards.org/cgi-bin/carddisp.pl?gene=TK2 |
| MPL | MPL Proto-Oncogene, Thrombopoietin Receptor | Protein Coding |  | 54 | GC01P043337 | | 4.53 | https://www.genecards.org/cgi-bin/carddisp.pl?gene=MPL |
| CDC42 | Cell Division Cycle 42 | Protein Coding |  | 58 | GC01P022028 | | 4.51 | https://www.genecards.org/cgi-bin/carddisp.pl?gene=CDC42 |
| IKZF1 | IKAROS Family Zinc Finger 1 | Protein Coding |  | 53 | GC07P050343 | | 4.51 | https://www.genecards.org/cgi-bin/carddisp.pl?gene=IKZF1 |
| PMAIP1 | Phorbol-12-Myristate-13-Acetate-Induced Protein 1 | Protein Coding |  | 45 | GC18P059899 | | 4.51 | https://www.genecards.org/cgi-bin/carddisp.pl?gene=PMAIP1 |
| FLNA | Filamin A | Protein Coding |  | 54 | GC0XM154348 | | 4.51 | https://www.genecards.org/cgi-bin/carddisp.pl?gene=FLNA |
| MT-CYB | Mitochondrially Encoded Cytochrome B | Protein Coding |  | 35 | GCMTP014749 | | 4.5 | https://www.genecards.org/cgi-bin/carddisp.pl?gene=MT-CYB |
| HSPA5 | Heat Shock Protein Family A (Hsp70) Member 5 | Protein Coding |  | 53 | GC09M125234 | | 4.49 | https://www.genecards.org/cgi-bin/carddisp.pl?gene=HSPA5 |
| ACHE | Acetylcholinesterase (Cartwright Blood Group) | Protein Coding |  | 52 | GC07M100889 | | 4.48 | https://www.genecards.org/cgi-bin/carddisp.pl?gene=ACHE |
| SMPD1 | Sphingomyelin Phosphodiesterase 1 | Protein Coding |  | 53 | GC11P006390 | | 4.48 | https://www.genecards.org/cgi-bin/carddisp.pl?gene=SMPD1 |
| CCK | Cholecystokinin | Protein Coding |  | 47 | GC03M042274 | | 4.48 | https://www.genecards.org/cgi-bin/carddisp.pl?gene=CCK |
| TLR3 | Toll Like Receptor 3 | Protein Coding |  | 58 | GC04P186059 | | 4.48 | https://www.genecards.org/cgi-bin/carddisp.pl?gene=TLR3 |
| CTAG1B | Cancer/Testis Antigen 1B | Protein Coding |  | 35 | GC0XM154617 | | 4.47 | https://www.genecards.org/cgi-bin/carddisp.pl?gene=CTAG1B |
| MIRLET7F1 | MicroRNA Let-7f-1 | RNA Gene |  | 20 | GC09P094197 | | 4.47 | https://www.genecards.org/cgi-bin/carddisp.pl?gene=MIRLET7F1 |
| LEP | Leptin | Protein Coding |  | 54 | GC07P128241 | | 4.47 | https://www.genecards.org/cgi-bin/carddisp.pl?gene=LEP |
| AREG | Amphiregulin | Protein Coding |  | 50 | GC04P074445 | | 4.45 | https://www.genecards.org/cgi-bin/carddisp.pl?gene=AREG |
| TPO | Thyroid Peroxidase | Protein Coding |  | 54 | GC02P001396 | | 4.45 | https://www.genecards.org/cgi-bin/carddisp.pl?gene=TPO |
| STAT5B | Signal Transducer And Activator Of Transcription 5B | Protein Coding |  | 56 | GC17M042199 | | 4.45 | https://www.genecards.org/cgi-bin/carddisp.pl?gene=STAT5B |
| RRM1 | Ribonucleotide Reductase Catalytic Subunit M1 | Protein Coding |  | 54 | GC11P004115 | | 4.44 | https://www.genecards.org/cgi-bin/carddisp.pl?gene=RRM1 |
| CD1E | CD1e Molecule | Protein Coding |  | 46 | GC01P158323 | | 4.43 | https://www.genecards.org/cgi-bin/carddisp.pl?gene=CD1E |
| MAGEA4 | MAGE Family Member A4 | Protein Coding |  | 44 | GC0XP151912 | | 4.43 | https://www.genecards.org/cgi-bin/carddisp.pl?gene=MAGEA4 |
| DGCR5 | DiGeorge Syndrome Critical Region Gene 5 | RNA Gene |  | 22 | GC22P019274 | | 4.43 | https://www.genecards.org/cgi-bin/carddisp.pl?gene=DGCR5 |
| LINC00673 | Long Intergenic Non-Protein Coding RNA 673 | RNA Gene |  | 17 | GC17M072290 | | 4.43 | https://www.genecards.org/cgi-bin/carddisp.pl?gene=LINC00673 |
| CASC9 | Cancer Susceptibility 9 | RNA Gene |  | 15 | GC08M075223 | | 4.43 | https://www.genecards.org/cgi-bin/carddisp.pl?gene=CASC9 |
| NKILA | NF-KappaB Interacting LncRNA | RNA Gene |  | 13 | GC20P057711 | | 4.43 | https://www.genecards.org/cgi-bin/carddisp.pl?gene=NKILA |
| SHBG | Sex Hormone Binding Globulin | Protein Coding |  | 47 | GC17P007613 | | 4.42 | https://www.genecards.org/cgi-bin/carddisp.pl?gene=SHBG |
| CD59 | CD59 Molecule (CD59 Blood Group) | Protein Coding |  | 53 | GC11M033699 | | 4.41 | https://www.genecards.org/cgi-bin/carddisp.pl?gene=CD59 |
| SPI1 | Spi-1 Proto-Oncogene | Protein Coding |  | 50 | GC11M056039 | | 4.41 | https://www.genecards.org/cgi-bin/carddisp.pl?gene=SPI1 |
| BMP2 | Bone Morphogenetic Protein 2 | Protein Coding |  | 53 | GC20P006696 | | 4.41 | https://www.genecards.org/cgi-bin/carddisp.pl?gene=BMP2 |
| MIR199A1 | MicroRNA 199a-1 | RNA Gene |  | 20 | GC19M010792 | | 4.37 | https://www.genecards.org/cgi-bin/carddisp.pl?gene=MIR199A1 |
| IKBKG | Inhibitor Of Nuclear Factor Kappa B Kinase Regulatory Subunit Gamma | Protein Coding |  | 54 | GC0XP154541 | | 4.37 | https://www.genecards.org/cgi-bin/carddisp.pl?gene=IKBKG |
| CD81 | CD81 Molecule | Protein Coding |  | 53 | GC11P002397 | | 4.36 | https://www.genecards.org/cgi-bin/carddisp.pl?gene=CD81 |
| LINC01116 | Long Intergenic Non-Protein Coding RNA 1116 | RNA Gene |  | 17 | GC02M176629 | | 4.36 | https://www.genecards.org/cgi-bin/carddisp.pl?gene=LINC01116 |
| G6PD | Glucose-6-Phosphate Dehydrogenase | Protein Coding |  | 56 | GC0XM154531 | | 4.35 | https://www.genecards.org/cgi-bin/carddisp.pl?gene=G6PD |
| APTX | Aprataxin | Protein Coding |  | 50 | GC09M032962 | | 4.34 | https://www.genecards.org/cgi-bin/carddisp.pl?gene=APTX |
| GFAP | Glial Fibrillary Acidic Protein | Protein Coding |  | 54 | GC17M044905 | | 4.34 | https://www.genecards.org/cgi-bin/carddisp.pl?gene=GFAP |
| PGR | Progesterone Receptor | Protein Coding |  | 57 | GC11M100943 | | 4.31 | https://www.genecards.org/cgi-bin/carddisp.pl?gene=PGR |
| TK1 | Thymidine Kinase 1 | Protein Coding |  | 52 | GC17M078175 | | 4.31 | https://www.genecards.org/cgi-bin/carddisp.pl?gene=TK1 |
| LY96 | Lymphocyte Antigen 96 | Protein Coding |  | 49 | GC08P073991 | | 4.3 | https://www.genecards.org/cgi-bin/carddisp.pl?gene=LY96 |
| PDCD1 | Programmed Cell Death 1 | Protein Coding |  | 53 | GC02M241849 | | 4.3 | https://www.genecards.org/cgi-bin/carddisp.pl?gene=PDCD1 |
| MIR95 | MicroRNA 95 | RNA Gene |  | 18 | GC04M008007 | | 4.3 | https://www.genecards.org/cgi-bin/carddisp.pl?gene=MIR95 |
| REN | Renin | Protein Coding |  | 54 | GC01M204123 | | 4.28 | https://www.genecards.org/cgi-bin/carddisp.pl?gene=REN |
| CCNE1 | Cyclin E1 | Protein Coding |  | 54 | GC19P029811 | | 4.28 | https://www.genecards.org/cgi-bin/carddisp.pl?gene=CCNE1 |
| PIGA | Phosphatidylinositol Glycan Anchor Biosynthesis Class A | Protein Coding |  | 51 | GC0XM015319 | | 4.27 | https://www.genecards.org/cgi-bin/carddisp.pl?gene=PIGA |
| LMNA | Lamin A/C | Protein Coding |  | 53 | GC01P156082 | | 4.26 | https://www.genecards.org/cgi-bin/carddisp.pl?gene=LMNA |
| MAGEA3 | MAGE Family Member A3 | Protein Coding |  | 42 | GC0XP152698 | | 4.25 | https://www.genecards.org/cgi-bin/carddisp.pl?gene=MAGEA3 |
| RIOX2 | Ribosomal Oxygenase 2 | Protein Coding |  | 36 | GC03M097942 | | 4.25 | https://www.genecards.org/cgi-bin/carddisp.pl?gene=RIOX2 |
| SEPTIN4 | Septin 4 | Protein Coding |  | 34 | GC17M058522 | | 4.25 | https://www.genecards.org/cgi-bin/carddisp.pl?gene=SEPTIN4 |
| RPS19 | Ribosomal Protein S19 | Protein Coding |  | 54 | GC19P041859 | | 4.22 | https://www.genecards.org/cgi-bin/carddisp.pl?gene=RPS19 |
| CST3 | Cystatin C | Protein Coding |  | 51 | GC20M023608 | | 4.22 | https://www.genecards.org/cgi-bin/carddisp.pl?gene=CST3 |
| GAPDH | Glyceraldehyde-3-Phosphate Dehydrogenase | Protein Coding |  | 54 | GC12P006533 | | 4.21 | https://www.genecards.org/cgi-bin/carddisp.pl?gene=GAPDH |
| CARTPT | CART Prepropeptide | Protein Coding |  | 49 | GC05P071719 | | 4.2 | https://www.genecards.org/cgi-bin/carddisp.pl?gene=CARTPT |
| ICOSLG | Inducible T Cell Costimulator Ligand | Protein Coding |  | 45 | GC21M044222 | | 4.2 | https://www.genecards.org/cgi-bin/carddisp.pl?gene=ICOSLG |
| MAGEA1 | MAGE Family Member A1 | Protein Coding |  | 43 | GC0XP153179 | | 4.2 | https://www.genecards.org/cgi-bin/carddisp.pl?gene=MAGEA1 |
| LINC00312 | Long Intergenic Non-Protein Coding RNA 312 | RNA Gene |  | 22 | GC03P008613 | | 4.2 | https://www.genecards.org/cgi-bin/carddisp.pl?gene=LINC00312 |
| SNHG7 | Small Nucleolar RNA Host Gene 7 | RNA Gene |  | 20 | GC09M136721 | | 4.2 | https://www.genecards.org/cgi-bin/carddisp.pl?gene=SNHG7 |
| MIR497 | MicroRNA 497 | RNA Gene |  | 19 | GC17M007022 | | 4.2 | https://www.genecards.org/cgi-bin/carddisp.pl?gene=MIR497 |
| FOXD2-AS1 | FOXD2 Adjacent Opposite Strand RNA 1 | RNA Gene |  | 18 | GC01M047432 | | 4.2 | https://www.genecards.org/cgi-bin/carddisp.pl?gene=FOXD2-AS1 |
| LINC00460 | Long Intergenic Non-Protein Coding RNA 460 | RNA Gene |  | 17 | GC13P106376 | | 4.2 | https://www.genecards.org/cgi-bin/carddisp.pl?gene=LINC00460 |
| FEZF1-AS1 | FEZF1 Antisense RNA 1 | RNA Gene |  | 17 | GC07P122303 | | 4.2 | https://www.genecards.org/cgi-bin/carddisp.pl?gene=FEZF1-AS1 |
| CASC8 | Cancer Susceptibility 8 | RNA Gene |  | 16 | GC08M127289 | | 4.2 | https://www.genecards.org/cgi-bin/carddisp.pl?gene=CASC8 |
| EPB41L4A-DT | EPB41L4A Divergent Transcript | RNA Gene |  | 15 | GC05P112421 | | 4.2 | https://www.genecards.org/cgi-bin/carddisp.pl?gene=EPB41L4A-DT |
| GACAT2 | Gastric Cancer Associated Transcript 2 | RNA Gene |  | 15 | GC18M008695 | | 4.2 | https://www.genecards.org/cgi-bin/carddisp.pl?gene=GACAT2 |
| TLR1 | Toll Like Receptor 1 | Protein Coding |  | 54 | GC04M038797 | | 4.2 | https://www.genecards.org/cgi-bin/carddisp.pl?gene=TLR1 |
| ACTC1 | Actin Alpha Cardiac Muscle 1 | Protein Coding |  | 48 | GC15M034788 | | 4.19 | https://www.genecards.org/cgi-bin/carddisp.pl?gene=ACTC1 |
| PNP | Purine Nucleoside Phosphorylase | Protein Coding |  | 54 | GC14P020468 | | 4.17 | https://www.genecards.org/cgi-bin/carddisp.pl?gene=PNP |
| ELAVL4 | ELAV Like RNA Binding Protein 4 | Protein Coding |  | 45 | GC01P050048 | | 4.16 | https://www.genecards.org/cgi-bin/carddisp.pl?gene=ELAVL4 |
| PDE4A | Phosphodiesterase 4A | Protein Coding |  | 52 | GC19P010392 | | 4.15 | https://www.genecards.org/cgi-bin/carddisp.pl?gene=PDE4A |
| MYCL | MYCL Proto-Oncogene, BHLH Transcription Factor | Protein Coding |  | 42 | GC01M039895 | | 4.13 | https://www.genecards.org/cgi-bin/carddisp.pl?gene=MYCL |
| MIR212 | MicroRNA 212 | RNA Gene |  | 20 | GC17M002050 | | 4.13 | https://www.genecards.org/cgi-bin/carddisp.pl?gene=MIR212 |
| MMP3 | Matrix Metallopeptidase 3 | Protein Coding |  | 57 | GC11M102835 | | 4.13 | https://www.genecards.org/cgi-bin/carddisp.pl?gene=MMP3 |
| BSG | Basigin (Ok Blood Group) | Protein Coding |  | 50 | GC19P000571 | | 4.12 | https://www.genecards.org/cgi-bin/carddisp.pl?gene=BSG |
| DKK1 | Dickkopf WNT Signaling Pathway Inhibitor 1 | Protein Coding |  | 51 | GC10P052314 | | 4.1 | https://www.genecards.org/cgi-bin/carddisp.pl?gene=DKK1 |
| MIR338 | MicroRNA 338 | RNA Gene |  | 20 | GC17M081126 | | 4.1 | https://www.genecards.org/cgi-bin/carddisp.pl?gene=MIR338 |
| IL22 | Interleukin 22 | Protein Coding |  | 47 | GC12M068248 | | 4.1 | https://www.genecards.org/cgi-bin/carddisp.pl?gene=IL22 |
| CRYAA | Crystallin Alpha A | Protein Coding |  | 50 | GC21P043169 | | 4.09 | https://www.genecards.org/cgi-bin/carddisp.pl?gene=CRYAA |
| RAG2 | Recombination Activating 2 | Protein Coding |  | 48 | GC11M036575 | | 4.08 | https://www.genecards.org/cgi-bin/carddisp.pl?gene=RAG2 |
| MTHFR | Methylenetetrahydrofolate Reductase | Protein Coding |  | 54 | GC01M011845 | | 4.06 | https://www.genecards.org/cgi-bin/carddisp.pl?gene=MTHFR |
| IL3RA | Interleukin 3 Receptor Subunit Alpha | Protein Coding |  | 49 | GC0XP001336 | | 4.06 | https://www.genecards.org/cgi-bin/carddisp.pl?gene=IL3RA |
| HDAC6 | Histone Deacetylase 6 | Protein Coding |  | 58 | GC0XP048801 | | 4.05 | https://www.genecards.org/cgi-bin/carddisp.pl?gene=HDAC6 |
| HDAC9 | Histone Deacetylase 9 | Protein Coding |  | 53 | GC07P018179 | | 4.05 | https://www.genecards.org/cgi-bin/carddisp.pl?gene=HDAC9 |
| CP | Ceruloplasmin | Protein Coding |  | 54 | GC03M149162 | | 4.05 | https://www.genecards.org/cgi-bin/carddisp.pl?gene=CP |
| ZNRD1ASP | Zinc Ribbon Domain Containing 1 Antisense, Pseudogene | Pseudogene |  | 17 | GC06M030480 | | 4.03 | https://www.genecards.org/cgi-bin/carddisp.pl?gene=ZNRD1ASP |
| PDGFRA | Platelet Derived Growth Factor Receptor Alpha | Protein Coding |  | 62 | GC04P054229 | | 4.02 | https://www.genecards.org/cgi-bin/carddisp.pl?gene=PDGFRA |
| MAPK9 | Mitogen-Activated Protein Kinase 9 | Protein Coding |  | 54 | GC05M180234 | | 4.02 | https://www.genecards.org/cgi-bin/carddisp.pl?gene=MAPK9 |
| IL12B | Interleukin 12B | Protein Coding |  | 50 | GC05M159314 | | 4.01 | https://www.genecards.org/cgi-bin/carddisp.pl?gene=IL12B |
| MAP2 | Microtubule Associated Protein 2 | Protein Coding |  | 49 | GC02P209424 | | 4.01 | https://www.genecards.org/cgi-bin/carddisp.pl?gene=MAP2 |
| TCF7 | Transcription Factor 7 | Protein Coding |  | 50 | GC05P134114 | | 4 | https://www.genecards.org/cgi-bin/carddisp.pl?gene=TCF7 |
| CFAP410 | Cilia And Flagella Associated Protein 410 | Protein Coding |  | 35 | GC21M044330 | | 4 | https://www.genecards.org/cgi-bin/carddisp.pl?gene=CFAP410 |
| TSC2 | TSC Complex Subunit 2 | Protein Coding |  | 56 | GC16P002323 | | 3.98 | https://www.genecards.org/cgi-bin/carddisp.pl?gene=TSC2 |
| STAT6 | Signal Transducer And Activator Of Transcription 6 | Protein Coding |  | 55 | GC12M057095 | | 3.98 | https://www.genecards.org/cgi-bin/carddisp.pl?gene=STAT6 |
| XBP1 | X-Box Binding Protein 1 | Protein Coding |  | 52 | GC22M028794 | | 3.97 | https://www.genecards.org/cgi-bin/carddisp.pl?gene=XBP1 |
| CYP19A1 | Cytochrome P450 Family 19 Subfamily A Member 1 | Protein Coding |  | 56 | GC15M051208 | | 3.97 | https://www.genecards.org/cgi-bin/carddisp.pl?gene=CYP19A1 |
| PPARA | Peroxisome Proliferator Activated Receptor Alpha | Protein Coding |  | 51 | GC22P046150 | | 3.97 | https://www.genecards.org/cgi-bin/carddisp.pl?gene=PPARA |
| CRBN | Cereblon | Protein Coding |  | 49 | GC03M003166 | | 3.96 | https://www.genecards.org/cgi-bin/carddisp.pl?gene=CRBN |
| TP53TG1 | TP53 Target 1 | RNA Gene |  | 25 | GC07M087325 | | 3.96 | https://www.genecards.org/cgi-bin/carddisp.pl?gene=TP53TG1 |
| ZEB2-AS1 | ZEB2 Antisense RNA 1 | RNA Gene |  | 20 | GC02P144519 | | 3.96 | https://www.genecards.org/cgi-bin/carddisp.pl?gene=ZEB2-AS1 |
| LINC00511 | Long Intergenic Non-Protein Coding RNA 511 | RNA Gene |  | 18 | GC17M072323 | | 3.96 | https://www.genecards.org/cgi-bin/carddisp.pl?gene=LINC00511 |
| LINC01133 | Long Intergenic Non-Protein Coding RNA 1133 | RNA Gene |  | 16 | GC01P159933 | | 3.96 | https://www.genecards.org/cgi-bin/carddisp.pl?gene=LINC01133 |
| HIF1A-AS1 | HIF1A Antisense RNA 1 | RNA Gene |  | 15 | GC14M061681 | | 3.96 | https://www.genecards.org/cgi-bin/carddisp.pl?gene=HIF1A-AS1 |
| TGM4 | Transglutaminase 4 | Protein Coding |  | 45 | GC03P044874 | | 3.96 | https://www.genecards.org/cgi-bin/carddisp.pl?gene=TGM4 |
| IFNGR1 | Interferon Gamma Receptor 1 | Protein Coding |  | 56 | GC06M137197 | | 3.94 | https://www.genecards.org/cgi-bin/carddisp.pl?gene=IFNGR1 |
| MIR99B | MicroRNA 99b | RNA Gene |  | 19 | GC19P051692 | | 3.93 | https://www.genecards.org/cgi-bin/carddisp.pl?gene=MIR99B |
| GRN | Granulin Precursor | Protein Coding |  | 53 | GC17P044345 | | 3.92 | https://www.genecards.org/cgi-bin/carddisp.pl?gene=GRN |
| FER | FER Tyrosine Kinase | Protein Coding |  | 53 | GC05P108747 | | 3.91 | https://www.genecards.org/cgi-bin/carddisp.pl?gene=FER |
| PLOD1 | Procollagen-Lysine,2-Oxoglutarate 5-Dioxygenase 1 | Protein Coding |  | 48 | GC01P011934 | | 3.9 | https://www.genecards.org/cgi-bin/carddisp.pl?gene=PLOD1 |
| F5 | Coagulation Factor V | Protein Coding |  | 52 | GC01M169481 | | 3.9 | https://www.genecards.org/cgi-bin/carddisp.pl?gene=F5 |
| SLC52A3 | Solute Carrier Family 52 Member 3 | Protein Coding |  | 43 | GC20M000741 | | 3.89 | https://www.genecards.org/cgi-bin/carddisp.pl?gene=SLC52A3 |
| IRF4 | Interferon Regulatory Factor 4 | Protein Coding |  | 50 | GC06P000391 | | 3.89 | https://www.genecards.org/cgi-bin/carddisp.pl?gene=IRF4 |
| SDC1 | Syndecan 1 | Protein Coding |  | 50 | GC02M020200 | | 3.89 | https://www.genecards.org/cgi-bin/carddisp.pl?gene=SDC1 |
| ADAM12 | ADAM Metallopeptidase Domain 12 | Protein Coding |  | 52 | GC10M126012 | | 3.89 | https://www.genecards.org/cgi-bin/carddisp.pl?gene=ADAM12 |
| SAT1 | Spermidine/Spermine N1-Acetyltransferase 1 | Protein Coding |  | 51 | GC0XP023784 | | 3.86 | https://www.genecards.org/cgi-bin/carddisp.pl?gene=SAT1 |
| DIABLO | Diablo IAP-Binding Mitochondrial Protein | Protein Coding |  | 53 | GC12M122208 | | 3.86 | https://www.genecards.org/cgi-bin/carddisp.pl?gene=DIABLO |
| CHKB | Choline Kinase Beta | Protein Coding |  | 51 | GC22M050578 | | 3.84 | https://www.genecards.org/cgi-bin/carddisp.pl?gene=CHKB |
| SMARCB1 | SWI/SNF Related, Matrix Associated, Actin Dependent Regulator Of Chromatin, Subfamily B, Member 1 | Protein Coding |  | 51 | GC22P023786 | | 3.84 | https://www.genecards.org/cgi-bin/carddisp.pl?gene=SMARCB1 |
| MIR33A | MicroRNA 33a | RNA Gene |  | 20 | GC22P041900 | | 3.83 | https://www.genecards.org/cgi-bin/carddisp.pl?gene=MIR33A |
| RCVRN | Recoverin | Protein Coding |  | 44 | GC17M009896 | | 3.83 | https://www.genecards.org/cgi-bin/carddisp.pl?gene=RCVRN |
| MIR124-3 | MicroRNA 124-3 | RNA Gene |  | 18 | GC20P063180 | | 3.82 | https://www.genecards.org/cgi-bin/carddisp.pl?gene=MIR124-3 |
| S100A8 | S100 Calcium Binding Protein A8 | Protein Coding |  | 48 | GC01M153391 | | 3.82 | https://www.genecards.org/cgi-bin/carddisp.pl?gene=S100A8 |
| SST | Somatostatin | Protein Coding |  | 48 | GC03M187668 | | 3.81 | https://www.genecards.org/cgi-bin/carddisp.pl?gene=SST |
| FADD | Fas Associated Via Death Domain | Protein Coding |  | 53 | GC11P070203 | | 3.79 | https://www.genecards.org/cgi-bin/carddisp.pl?gene=FADD |
| MIR216A | MicroRNA 216a | RNA Gene |  | 21 | GC02M055988 | | 3.79 | https://www.genecards.org/cgi-bin/carddisp.pl?gene=MIR216A |
| VDAC1 | Voltage Dependent Anion Channel 1 | Protein Coding |  | 53 | GC05M133975 | | 3.79 | https://www.genecards.org/cgi-bin/carddisp.pl?gene=VDAC1 |
| NOX4 | NADPH Oxidase 4 | Protein Coding |  | 48 | GC11M089324 | | 3.78 | https://www.genecards.org/cgi-bin/carddisp.pl?gene=NOX4 |
| SOD3 | Superoxide Dismutase 3 | Protein Coding |  | 45 | GC04P024798 | | 3.78 | https://www.genecards.org/cgi-bin/carddisp.pl?gene=SOD3 |
| HNRNPA2B1 | Heterogeneous Nuclear Ribonucleoprotein A2/B1 | Protein Coding |  | 51 | GC07M026174 | | 3.78 | https://www.genecards.org/cgi-bin/carddisp.pl?gene=HNRNPA2B1 |
| NLRP3 | NLR Family Pyrin Domain Containing 3 | Protein Coding |  | 53 | GC01P247415 | | 3.78 | https://www.genecards.org/cgi-bin/carddisp.pl?gene=NLRP3 |
| LRP1B | LDL Receptor Related Protein 1B | Protein Coding |  | 45 | GC02M140231 | | 3.78 | https://www.genecards.org/cgi-bin/carddisp.pl?gene=LRP1B |
| GRB2 | Growth Factor Receptor Bound Protein 2 | Protein Coding |  | 56 | GC17M075318 | | 3.78 | https://www.genecards.org/cgi-bin/carddisp.pl?gene=GRB2 |
| CKS1B | CDC28 Protein Kinase Regulatory Subunit 1B | Protein Coding |  | 47 | GC01P154974 | | 3.76 | https://www.genecards.org/cgi-bin/carddisp.pl?gene=CKS1B |
| CSF3R | Colony Stimulating Factor 3 Receptor | Protein Coding |  | 54 | GC01M036466 | | 3.76 | https://www.genecards.org/cgi-bin/carddisp.pl?gene=CSF3R |
| MIR125B2 | MicroRNA 125b-2 | RNA Gene |  | 20 | GC21P016590 | | 3.75 | https://www.genecards.org/cgi-bin/carddisp.pl?gene=MIR125B2 |
| MIR339 | MicroRNA 339 | RNA Gene |  | 20 | GC07M001022 | | 3.75 | https://www.genecards.org/cgi-bin/carddisp.pl?gene=MIR339 |
| CD24 | CD24 Molecule | Protein Coding |  | 37 | GC06M106969 | | 3.74 | https://www.genecards.org/cgi-bin/carddisp.pl?gene=CD24 |
| C1QBP | Complement C1q Binding Protein | Protein Coding |  | 52 | GC17M005432 | | 3.73 | https://www.genecards.org/cgi-bin/carddisp.pl?gene=C1QBP |
| GATA2 | GATA Binding Protein 2 | Protein Coding |  | 54 | GC03M128479 | | 3.73 | https://www.genecards.org/cgi-bin/carddisp.pl?gene=GATA2 |
| ACTA1 | Actin Alpha 1, Skeletal Muscle | Protein Coding |  | 53 | GC01M229431 | | 3.72 | https://www.genecards.org/cgi-bin/carddisp.pl?gene=ACTA1 |
| CD7 | CD7 Molecule | Protein Coding |  | 45 | GC17M082314 | | 3.72 | https://www.genecards.org/cgi-bin/carddisp.pl?gene=CD7 |
| SQSTM1 | Sequestosome 1 | Protein Coding |  | 55 | GC05P179806 | | 3.72 | https://www.genecards.org/cgi-bin/carddisp.pl?gene=SQSTM1 |
| SLC26A4 | Solute Carrier Family 26 Member 4 | Protein Coding |  | 49 | GC07P107660 | | 3.71 | https://www.genecards.org/cgi-bin/carddisp.pl?gene=SLC26A4 |
| GHRL | Ghrelin And Obestatin Prepropeptide | Protein Coding |  | 48 | GC03M010285 | | 3.7 | https://www.genecards.org/cgi-bin/carddisp.pl?gene=GHRL |
| COL18A1 | Collagen Type XVIII Alpha 1 Chain | Protein Coding |  | 51 | GC21P045405 | | 3.7 | https://www.genecards.org/cgi-bin/carddisp.pl?gene=COL18A1 |
| KLF4 | Kruppel Like Factor 4 | Protein Coding |  | 52 | GC09M107484 | | 3.69 | https://www.genecards.org/cgi-bin/carddisp.pl?gene=KLF4 |
| MYOM2 | Myomesin 2 | Protein Coding |  | 44 | GC08P002045 | | 3.69 | https://www.genecards.org/cgi-bin/carddisp.pl?gene=MYOM2 |
| MAGEC1 | MAGE Family Member C1 | Protein Coding |  | 39 | GC0XP141905 | | 3.69 | https://www.genecards.org/cgi-bin/carddisp.pl?gene=MAGEC1 |
| MIR22HG | MIR22 Host Gene | RNA Gene |  | 29 | GC17M001711 | | 3.69 | https://www.genecards.org/cgi-bin/carddisp.pl?gene=MIR22HG |
| IGF2-AS | IGF2 Antisense RNA | RNA Gene |  | 28 | GC11P002140 | | 3.69 | https://www.genecards.org/cgi-bin/carddisp.pl?gene=IGF2-AS |
| FAM83H-AS1 | FAM83H Antisense RNA 1 (Head To Head) | RNA Gene |  | 17 | GC08P143734 | | 3.69 | https://www.genecards.org/cgi-bin/carddisp.pl?gene=FAM83H-AS1 |
| CPS1-IT1 | CPS1 Intronic Transcript 1 | RNA Gene |  | 17 | GC02P210617 | | 3.69 | https://www.genecards.org/cgi-bin/carddisp.pl?gene=CPS1-IT1 |
| SBF2-AS1 | SBF2 Antisense RNA 1 | RNA Gene |  | 17 | GC11P009781 | | 3.69 | https://www.genecards.org/cgi-bin/carddisp.pl?gene=SBF2-AS1 |
| LINC00858 | Long Intergenic Non-Protein Coding RNA 858 | RNA Gene |  | 16 | GC10P084279 | | 3.69 | https://www.genecards.org/cgi-bin/carddisp.pl?gene=LINC00858 |
| MIR4435-2HG | MIR4435-2 Host Gene | RNA Gene |  | 15 | GC02M111037 | | 3.69 | https://www.genecards.org/cgi-bin/carddisp.pl?gene=MIR4435-2HG |
| AGAP2-AS1 | AGAP2 Antisense RNA 1 | RNA Gene |  | 15 | GC12P057726 | | 3.69 | https://www.genecards.org/cgi-bin/carddisp.pl?gene=AGAP2-AS1 |
| RGMB-AS1 | RGMB Antisense RNA 1 | RNA Gene |  | 15 | GC05M098769 | | 3.69 | https://www.genecards.org/cgi-bin/carddisp.pl?gene=RGMB-AS1 |
| PDIA3P1 | Protein Disulfide Isomerase Family A Member 3 Pseudogene 1 | Pseudogene |  | 15 | GC01P147184 | | 3.69 | https://www.genecards.org/cgi-bin/carddisp.pl?gene=PDIA3P1 |
| MAFA-AS1 | MAFA Antisense RNA 1 | RNA Gene |  | 14 | GC08P143417 | | 3.69 | https://www.genecards.org/cgi-bin/carddisp.pl?gene=MAFA-AS1 |
| LINC01852 | Long Intergenic Non-Protein Coding RNA 1852 | RNA Gene |  | 11 | GC15M038070 | | 3.69 | https://www.genecards.org/cgi-bin/carddisp.pl?gene=LINC01852 |
| AGT | Angiotensinogen | Protein Coding |  | 56 | GC01M230702 | | 3.68 | https://www.genecards.org/cgi-bin/carddisp.pl?gene=AGT |
| SPINK1 | Serine Peptidase Inhibitor, Kazal Type 1 | Protein Coding |  | 50 | GC05M147785 | | 3.68 | https://www.genecards.org/cgi-bin/carddisp.pl?gene=SPINK1 |
| JAK1 | Janus Kinase 1 | Protein Coding |  | 56 | GC01M064833 | | 3.68 | https://www.genecards.org/cgi-bin/carddisp.pl?gene=JAK1 |
| HSPA4 | Heat Shock Protein Family A (Hsp70) Member 4 | Protein Coding |  | 48 | GC05P133051 | | 3.68 | https://www.genecards.org/cgi-bin/carddisp.pl?gene=HSPA4 |
| NSD2 | Nuclear Receptor Binding SET Domain Protein 2 | Protein Coding |  | 40 | GC04P001872 | | 3.67 | https://www.genecards.org/cgi-bin/carddisp.pl?gene=NSD2 |
| SIRT1 | Sirtuin 1 | Protein Coding |  | 56 | GC10P067884 | | 3.67 | https://www.genecards.org/cgi-bin/carddisp.pl?gene=SIRT1 |
| TUBB4A | Tubulin Beta 4A Class IVa | Protein Coding |  | 51 | GC19M006496 | | 3.66 | https://www.genecards.org/cgi-bin/carddisp.pl?gene=TUBB4A |
| ANXA2 | Annexin A2 | Protein Coding |  | 53 | GC15M060347 | | 3.65 | https://www.genecards.org/cgi-bin/carddisp.pl?gene=ANXA2 |
| UCHL1 | Ubiquitin C-Terminal Hydrolase L1 | Protein Coding |  | 58 | GC04P041174 | | 3.65 | https://www.genecards.org/cgi-bin/carddisp.pl?gene=UCHL1 |
| NFIC | Nuclear Factor I C | Protein Coding |  | 46 | GC19P003359 | | 3.65 | https://www.genecards.org/cgi-bin/carddisp.pl?gene=NFIC |
| LAMC2 | Laminin Subunit Gamma 2 | Protein Coding |  | 51 | GC01P183155 | | 3.65 | https://www.genecards.org/cgi-bin/carddisp.pl?gene=LAMC2 |
| EHMT2 | Euchromatic Histone Lysine Methyltransferase 2 | Protein Coding |  | 52 | GC06M031879 | | 3.64 | https://www.genecards.org/cgi-bin/carddisp.pl?gene=EHMT2 |
| TOLLIP | Toll Interacting Protein | Protein Coding |  | 50 | GC11M001274 | | 3.64 | https://www.genecards.org/cgi-bin/carddisp.pl?gene=TOLLIP |
| CASP7 | Caspase 7 | Protein Coding |  | 56 | GC10P113679 | | 3.64 | https://www.genecards.org/cgi-bin/carddisp.pl?gene=CASP7 |
| HBB | Hemoglobin Subunit Beta | Protein Coding |  | 52 | GC11M005238 | | 3.63 | https://www.genecards.org/cgi-bin/carddisp.pl?gene=HBB |
| DPP4 | Dipeptidyl Peptidase 4 | Protein Coding |  | 56 | GC02M161992 | | 3.63 | https://www.genecards.org/cgi-bin/carddisp.pl?gene=DPP4 |
| NPPB | Natriuretic Peptide B | Protein Coding |  | 50 | GC01M011918 | | 3.62 | https://www.genecards.org/cgi-bin/carddisp.pl?gene=NPPB |
| IMMT | Inner Membrane Mitochondrial Protein | Protein Coding |  | 45 | GC02M086144 | | 3.62 | https://www.genecards.org/cgi-bin/carddisp.pl?gene=IMMT |
| CASC3 | CASC3 Exon Junction Complex Subunit | Protein Coding |  | 42 | GC17P040140 | | 3.62 | https://www.genecards.org/cgi-bin/carddisp.pl?gene=CASC3 |
| C14orf132 | Chromosome 14 Open Reading Frame 132 | Protein Coding |  | 28 | GC14P096040 | | 3.62 | https://www.genecards.org/cgi-bin/carddisp.pl?gene=C14orf132 |
| AGTR1 | Angiotensin II Receptor Type 1 | Protein Coding |  | 56 | GC03P148697 | | 3.61 | https://www.genecards.org/cgi-bin/carddisp.pl?gene=AGTR1 |
| PDPK1 | 3-Phosphoinositide Dependent Protein Kinase 1 | Protein Coding |  | 54 | GC16P002537 | | 3.61 | https://www.genecards.org/cgi-bin/carddisp.pl?gene=PDPK1 |
| ARG1 | Arginase 1 | Protein Coding |  | 56 | GC06P131553 | | 3.6 | https://www.genecards.org/cgi-bin/carddisp.pl?gene=ARG1 |
| NPPA | Natriuretic Peptide A | Protein Coding |  | 53 | GC01M011909 | | 3.6 | https://www.genecards.org/cgi-bin/carddisp.pl?gene=NPPA |
| CD86 | CD86 Molecule | Protein Coding |  | 50 | GC03P122055 | | 3.59 | https://www.genecards.org/cgi-bin/carddisp.pl?gene=CD86 |
| PAX5 | Paired Box 5 | Protein Coding |  | 53 | GC09M036828 | | 3.59 | https://www.genecards.org/cgi-bin/carddisp.pl?gene=PAX5 |
| MIR124-1 | MicroRNA 124-1 | RNA Gene |  | 21 | GC08M009901 | | 3.58 | https://www.genecards.org/cgi-bin/carddisp.pl?gene=MIR124-1 |
| CD2 | CD2 Molecule | Protein Coding |  | 50 | GC01P116754 | | 3.58 | https://www.genecards.org/cgi-bin/carddisp.pl?gene=CD2 |
| CASP1 | Caspase 1 | Protein Coding |  | 56 | GC11M105025 | | 3.58 | https://www.genecards.org/cgi-bin/carddisp.pl?gene=CASP1 |
| XRCC2 | X-Ray Repair Cross Complementing 2 | Protein Coding |  | 47 | GC07M152644 | | 3.58 | https://www.genecards.org/cgi-bin/carddisp.pl?gene=XRCC2 |
| AQP5 | Aquaporin 5 | Protein Coding |  | 52 | GC12P049961 | | 3.58 | https://www.genecards.org/cgi-bin/carddisp.pl?gene=AQP5 |
| ZBTB24 | Zinc Finger And BTB Domain Containing 24 | Protein Coding |  | 45 | GC06M109462 | | 3.57 | https://www.genecards.org/cgi-bin/carddisp.pl?gene=ZBTB24 |
| PSAP | Prosaposin | Protein Coding |  | 52 | GC10M071816 | | 3.56 | https://www.genecards.org/cgi-bin/carddisp.pl?gene=PSAP |
| ALOX12 | Arachidonate 12-Lipoxygenase, 12S Type | Protein Coding |  | 50 | GC17P006995 | | 3.56 | https://www.genecards.org/cgi-bin/carddisp.pl?gene=ALOX12 |
| POLA1 | DNA Polymerase Alpha 1, Catalytic Subunit | Protein Coding |  | 53 | GC0XP024712 | | 3.56 | https://www.genecards.org/cgi-bin/carddisp.pl?gene=POLA1 |
| ALMS1 | ALMS1 Centrosome And Basal Body Associated Protein | Protein Coding |  | 46 | GC02P073385 | | 3.56 | https://www.genecards.org/cgi-bin/carddisp.pl?gene=ALMS1 |
| HSPA1A | Heat Shock Protein Family A (Hsp70) Member 1A | Protein Coding |  | 49 | GC06P032520 | | 3.55 | https://www.genecards.org/cgi-bin/carddisp.pl?gene=HSPA1A |
| APOA1 | Apolipoprotein A1 | Protein Coding |  | 56 | GC11M116835 | | 3.55 | https://www.genecards.org/cgi-bin/carddisp.pl?gene=APOA1 |
| EP300 | E1A Binding Protein P300 | Protein Coding |  | 57 | GC22P041091 | | 3.55 | https://www.genecards.org/cgi-bin/carddisp.pl?gene=EP300 |
| ATF2 | Activating Transcription Factor 2 | Protein Coding |  | 52 | GC02M175072 | | 3.54 | https://www.genecards.org/cgi-bin/carddisp.pl?gene=ATF2 |
| TRAF6 | TNF Receptor Associated Factor 6 | Protein Coding |  | 53 | GC11M036467 | | 3.54 | https://www.genecards.org/cgi-bin/carddisp.pl?gene=TRAF6 |
| POSTN | Periostin | Protein Coding |  | 50 | GC13M037562 | | 3.54 | https://www.genecards.org/cgi-bin/carddisp.pl?gene=POSTN |
| DUSP1 | Dual Specificity Phosphatase 1 | Protein Coding |  | 53 | GC05M172768 | | 3.52 | https://www.genecards.org/cgi-bin/carddisp.pl?gene=DUSP1 |
| ADRB2 | Adrenoceptor Beta 2 | Protein Coding |  | 56 | GC05P148825 | | 3.52 | https://www.genecards.org/cgi-bin/carddisp.pl?gene=ADRB2 |
| LIF | LIF Interleukin 6 Family Cytokine | Protein Coding |  | 49 | GC22M030240 | | 3.51 | https://www.genecards.org/cgi-bin/carddisp.pl?gene=LIF |
| SP1 | Sp1 Transcription Factor | Protein Coding |  | 50 | GC12P053380 | | 3.5 | https://www.genecards.org/cgi-bin/carddisp.pl?gene=SP1 |
| DRD2 | Dopamine Receptor D2 | Protein Coding |  | 56 | GC11M113314 | | 3.49 | https://www.genecards.org/cgi-bin/carddisp.pl?gene=DRD2 |
| GDF15 | Growth Differentiation Factor 15 | Protein Coding |  | 48 | GC19P020641 | | 3.49 | https://www.genecards.org/cgi-bin/carddisp.pl?gene=GDF15 |
| PMM2 | Phosphomannomutase 2 | Protein Coding |  | 53 | GC16P008788 | | 3.49 | https://www.genecards.org/cgi-bin/carddisp.pl?gene=PMM2 |
| GRPR | Gastrin Releasing Peptide Receptor | Protein Coding |  | 49 | GC0XP016141 | | 3.49 | https://www.genecards.org/cgi-bin/carddisp.pl?gene=GRPR |
| GSN | Gelsolin | Protein Coding |  | 55 | GC09P121207 | | 3.48 | https://www.genecards.org/cgi-bin/carddisp.pl?gene=GSN |
| LAMA5-AS1 | LAMA5 Antisense RNA 1 | RNA Gene |  | 14 | GC20P062352 | | 3.48 | https://www.genecards.org/cgi-bin/carddisp.pl?gene=LAMA5-AS1 |
| TIMP3 | TIMP Metallopeptidase Inhibitor 3 | Protein Coding |  | 51 | GC22P032800 | | 3.48 | https://www.genecards.org/cgi-bin/carddisp.pl?gene=TIMP3 |
| MICA | MHC Class I Polypeptide-Related Sequence A | Protein Coding |  | 45 | GC06P031399 | | 3.47 | https://www.genecards.org/cgi-bin/carddisp.pl?gene=MICA |
| PRL | Prolactin | Protein Coding |  | 49 | GC06M022230 | | 3.47 | https://www.genecards.org/cgi-bin/carddisp.pl?gene=PRL |
| IL15 | Interleukin 15 | Protein Coding |  | 47 | GC04P141636 | | 3.47 | https://www.genecards.org/cgi-bin/carddisp.pl?gene=IL15 |
| NTRK3 | Neurotrophic Receptor Tyrosine Kinase 3 | Protein Coding |  | 57 | GC15M087859 | | 3.46 | https://www.genecards.org/cgi-bin/carddisp.pl?gene=NTRK3 |
| RBM38 | RNA Binding Motif Protein 38 | Protein Coding |  | 43 | GC20P057391 | | 3.45 | https://www.genecards.org/cgi-bin/carddisp.pl?gene=RBM38 |
| MAP3K7 | Mitogen-Activated Protein Kinase Kinase Kinase 7 | Protein Coding |  | 57 | GC06M090513 | | 3.45 | https://www.genecards.org/cgi-bin/carddisp.pl?gene=MAP3K7 |
| PSMB5 | Proteasome Subunit Beta 5 | Protein Coding |  | 51 | GC14M023016 | | 3.44 | https://www.genecards.org/cgi-bin/carddisp.pl?gene=PSMB5 |
| S100A2 | S100 Calcium Binding Protein A2 | Protein Coding |  | 47 | GC01M153533 | | 3.44 | https://www.genecards.org/cgi-bin/carddisp.pl?gene=S100A2 |
| MYOD1 | Myogenic Differentiation 1 | Protein Coding |  | 50 | GC11P017741 | | 3.43 | https://www.genecards.org/cgi-bin/carddisp.pl?gene=MYOD1 |
| BRAT1 | BRCA1 Associated ATM Activator 1 | Protein Coding |  | 43 | GC07M002577 | | 3.43 | https://www.genecards.org/cgi-bin/carddisp.pl?gene=BRAT1 |
| PRKCI | Protein Kinase C Iota | Protein Coding |  | 56 | GC03P170222 | | 3.43 | https://www.genecards.org/cgi-bin/carddisp.pl?gene=PRKCI |
| CTNND1 | Catenin Delta 1 | Protein Coding |  | 52 | GC11P057761 | | 3.43 | https://www.genecards.org/cgi-bin/carddisp.pl?gene=CTNND1 |
| EML4 | EMAP Like 4 | Protein Coding |  | 44 | GC02P042131 | | 3.43 | https://www.genecards.org/cgi-bin/carddisp.pl?gene=EML4 |
| MAEA | Macrophage Erythroblast Attacher | Protein Coding |  | 43 | GC04P001289 | | 3.43 | https://www.genecards.org/cgi-bin/carddisp.pl?gene=MAEA |
| MIR101-1 | MicroRNA 101-1 | RNA Gene |  | 19 | GC01M065058 | | 3.43 | https://www.genecards.org/cgi-bin/carddisp.pl?gene=MIR101-1 |
| ASAP1-IT1 | ASAP1 Intronic Transcript 1 | RNA Gene |  | 15 | GC08M130295 | | 3.43 | https://www.genecards.org/cgi-bin/carddisp.pl?gene=ASAP1-IT1 |
| AIRE | Autoimmune Regulator | Protein Coding |  | 51 | GC21P044285 | | 3.42 | https://www.genecards.org/cgi-bin/carddisp.pl?gene=AIRE |
| CD70 | CD70 Molecule | Protein Coding |  | 49 | GC19M006583 | | 3.42 | https://www.genecards.org/cgi-bin/carddisp.pl?gene=CD70 |
| RARA | Retinoic Acid Receptor Alpha | Protein Coding |  | 57 | GC17P040309 | | 3.42 | https://www.genecards.org/cgi-bin/carddisp.pl?gene=RARA |
| CACNA1A | Calcium Voltage-Gated Channel Subunit Alpha1 A | Protein Coding |  | 56 | GC19M013178 | | 3.41 | https://www.genecards.org/cgi-bin/carddisp.pl?gene=CACNA1A |
| TGFB2 | Transforming Growth Factor Beta 2 | Protein Coding |  | 55 | GC01P218345 | | 3.41 | https://www.genecards.org/cgi-bin/carddisp.pl?gene=TGFB2 |
| SOCS1 | Suppressor Of Cytokine Signaling 1 | Protein Coding |  | 50 | GC16M011255 | | 3.41 | https://www.genecards.org/cgi-bin/carddisp.pl?gene=SOCS1 |
| CHUK | Component Of Inhibitor Of Nuclear Factor Kappa B Kinase Complex | Protein Coding |  | 59 | GC10M100188 | | 3.41 | https://www.genecards.org/cgi-bin/carddisp.pl?gene=CHUK |
| OMP | Olfactory Marker Protein | Protein Coding |  | 43 | GC11P077102 | | 3.41 | https://www.genecards.org/cgi-bin/carddisp.pl?gene=OMP |
| IL1RAPL2 | Interleukin 1 Receptor Accessory Protein Like 2 | Protein Coding |  | 43 | GC0XP104566 | | 3.39 | https://www.genecards.org/cgi-bin/carddisp.pl?gene=IL1RAPL2 |
| HSPA8 | Heat Shock Protein Family A (Hsp70) Member 8 | Protein Coding |  | 54 | GC11M123057 | | 3.39 | https://www.genecards.org/cgi-bin/carddisp.pl?gene=HSPA8 |
| MAF | MAF BZIP Transcription Factor | Protein Coding |  | 52 | GC16M079212 | | 3.38 | https://www.genecards.org/cgi-bin/carddisp.pl?gene=MAF |
| MAFB | MAF BZIP Transcription Factor B | Protein Coding |  | 50 | GC20M040685 | | 3.38 | https://www.genecards.org/cgi-bin/carddisp.pl?gene=MAFB |
| CDH13 | Cadherin 13 | Protein Coding |  | 48 | GC16P082660 | | 3.38 | https://www.genecards.org/cgi-bin/carddisp.pl?gene=CDH13 |
| SLAMF7 | SLAM Family Member 7 | Protein Coding |  | 48 | GC01P160709 | | 3.38 | https://www.genecards.org/cgi-bin/carddisp.pl?gene=SLAMF7 |
| DIS3 | DIS3 Homolog, Exosome Endoribonuclease And 3'-5' Exoribonuclease | Protein Coding |  | 46 | GC13M072752 | | 3.38 | https://www.genecards.org/cgi-bin/carddisp.pl?gene=DIS3 |
| BST2 | Bone Marrow Stromal Cell Antigen 2 | Protein Coding |  | 45 | GC19M017403 | | 3.38 | https://www.genecards.org/cgi-bin/carddisp.pl?gene=BST2 |
| DAZAP2 | DAZ Associated Protein 2 | Protein Coding |  | 43 | GC12P051238 | | 3.38 | https://www.genecards.org/cgi-bin/carddisp.pl?gene=DAZAP2 |
| FAM135A | Family With Sequence Similarity 135 Member A | Protein Coding |  | 40 | GC06P070412 | | 3.38 | https://www.genecards.org/cgi-bin/carddisp.pl?gene=FAM135A |
| CCDC54 | Coiled-Coil Domain Containing 54 | Protein Coding |  | 35 | GC03P107377 | | 3.38 | https://www.genecards.org/cgi-bin/carddisp.pl?gene=CCDC54 |
| TENT5C | Terminal Nucleotidyltransferase 5C | Protein Coding |  | 32 | GC01P117606 | | 3.38 | https://www.genecards.org/cgi-bin/carddisp.pl?gene=TENT5C |
| GAS8-AS1 | GAS8 Antisense RNA 1 | RNA Gene |  | 26 | GC16M090028 | | 3.38 | https://www.genecards.org/cgi-bin/carddisp.pl?gene=GAS8-AS1 |
| MIRLET7A2 | MicroRNA Let-7a-2 | RNA Gene |  | 21 | GC11M122146 | | 3.38 | https://www.genecards.org/cgi-bin/carddisp.pl?gene=MIRLET7A2 |
| LINC00473 | Long Intergenic Non-Protein Coding RNA 473 | RNA Gene |  | 21 | GC06M165328 | | 3.38 | https://www.genecards.org/cgi-bin/carddisp.pl?gene=LINC00473 |
| APELA | Apelin Receptor Early Endogenous Ligand | Protein Coding |  | 21 | GC04P164877 | | 3.38 | https://www.genecards.org/cgi-bin/carddisp.pl?gene=APELA |
| MIR198 | MicroRNA 198 | RNA Gene |  | 20 | GC03M120395 | | 3.38 | https://www.genecards.org/cgi-bin/carddisp.pl?gene=MIR198 |
| MIR219A1 | MicroRNA 219a-1 | RNA Gene |  | 20 | GC06P033207 | | 3.38 | https://www.genecards.org/cgi-bin/carddisp.pl?gene=MIR219A1 |
| MIR423 | MicroRNA 423 | RNA Gene |  | 18 | GC17P030117 | | 3.38 | https://www.genecards.org/cgi-bin/carddisp.pl?gene=MIR423 |
| ADAMTS9-AS2 | ADAMTS9 Antisense RNA 2 | RNA Gene |  | 18 | GC03P064671 | | 3.38 | https://www.genecards.org/cgi-bin/carddisp.pl?gene=ADAMTS9-AS2 |
| SPAAR | Small Regulatory Polypeptide Of Amino Acid Response | Protein Coding |  | 18 | GC09P035910 | | 3.38 | https://www.genecards.org/cgi-bin/carddisp.pl?gene=SPAAR |
| MIR561 | MicroRNA 561 | RNA Gene |  | 17 | GC02P188297 | | 3.38 | https://www.genecards.org/cgi-bin/carddisp.pl?gene=MIR561 |
| ST3GAL6-AS1 | ST3GAL6 Antisense RNA 1 | RNA Gene |  | 16 | GC03M098714 | | 3.38 | https://www.genecards.org/cgi-bin/carddisp.pl?gene=ST3GAL6-AS1 |
| LINC00210 | Long Intergenic Non-Protein Coding RNA 210 | RNA Gene |  | 15 | GC01P217892 | | 3.38 | https://www.genecards.org/cgi-bin/carddisp.pl?gene=LINC00210 |
| SLC16A1-AS1 | SLC16A1 Antisense RNA 1 | RNA Gene |  | 15 | GC01P112956 | | 3.38 | https://www.genecards.org/cgi-bin/carddisp.pl?gene=SLC16A1-AS1 |
| PCAT6 | Prostate Cancer Associated Transcript 6 | RNA Gene |  | 15 | GC01P202810 | | 3.38 | https://www.genecards.org/cgi-bin/carddisp.pl?gene=PCAT6 |
| TRPM2-AS | TRPM2 Antisense RNA | RNA Gene |  | 15 | GC21M044414 | | 3.38 | https://www.genecards.org/cgi-bin/carddisp.pl?gene=TRPM2-AS |
| GAS5-AS1 | GAS5 Antisense RNA 1 | RNA Gene |  | 14 | GC01P173863 | | 3.38 | https://www.genecards.org/cgi-bin/carddisp.pl?gene=GAS5-AS1 |
| MALINC1 | Mitosis Associated Long Intergenic Non-Coding RNA 1 | RNA Gene |  | 13 | GC05M140073 | | 3.38 | https://www.genecards.org/cgi-bin/carddisp.pl?gene=MALINC1 |
| PCAT7 | Prostate Cancer Associated Transcript 7 | RNA Gene |  | 13 | GC09P094555 | | 3.38 | https://www.genecards.org/cgi-bin/carddisp.pl?gene=PCAT7 |
| PCBP2-OT1 | PCBP2 Overlapping Transcript 1 | RNA Gene |  | 12 | GC12P053464 | | 3.38 | https://www.genecards.org/cgi-bin/carddisp.pl?gene=PCBP2-OT1 |
| LOC730101 | Uncharacterized LOC730101 | RNA Gene |  | 11 | GC06P052664 | | 3.38 | https://www.genecards.org/cgi-bin/carddisp.pl?gene=LOC730101 |
| PRAL | P53 Regulation Associated LncRNA | RNA Gene |  | 10 | GC17M006773 | | 3.38 | https://www.genecards.org/cgi-bin/carddisp.pl?gene=PRAL |
| IGHV4-38-2 | Immunoglobulin Heavy Variable 4-38-2 | Protein Coding |  | 9 | GC14U901616 | | 3.38 | https://www.genecards.org/cgi-bin/carddisp.pl?gene=IGHV4-38-2 |
| FCER2 | Fc Fragment Of IgE Receptor II | Protein Coding |  | 51 | GC19M007689 | | 3.37 | https://www.genecards.org/cgi-bin/carddisp.pl?gene=FCER2 |
| BCOR | BCL6 Corepressor | Protein Coding |  | 47 | GC0XM040049 | | 3.37 | https://www.genecards.org/cgi-bin/carddisp.pl?gene=BCOR |
| CYP21A2 | Cytochrome P450 Family 21 Subfamily A Member 2 | Protein Coding |  | 51 | GC06P032526 | | 3.36 | https://www.genecards.org/cgi-bin/carddisp.pl?gene=CYP21A2 |
| CCL7 | C-C Motif Chemokine Ligand 7 | Protein Coding |  | 48 | GC17P034270 | | 3.36 | https://www.genecards.org/cgi-bin/carddisp.pl?gene=CCL7 |
| APOB | Apolipoprotein B | Protein Coding |  | 51 | GC02M020956 | | 3.36 | https://www.genecards.org/cgi-bin/carddisp.pl?gene=APOB |
| GPX1 | Glutathione Peroxidase 1 | Protein Coding |  | 54 | GC03M049374 | | 3.34 | https://www.genecards.org/cgi-bin/carddisp.pl?gene=GPX1 |
| RAP1A | RAP1A, Member Of RAS Oncogene Family | Protein Coding |  | 53 | GC01P111542 | | 3.33 | https://www.genecards.org/cgi-bin/carddisp.pl?gene=RAP1A |
| IL16 | Interleukin 16 | Protein Coding |  | 49 | GC15P081159 | | 3.33 | https://www.genecards.org/cgi-bin/carddisp.pl?gene=IL16 |
| PABPN1 | Poly(A) Binding Protein Nuclear 1 | Protein Coding |  | 52 | GC14P024684 | | 3.32 | https://www.genecards.org/cgi-bin/carddisp.pl?gene=PABPN1 |
| DNAH5 | Dynein Axonemal Heavy Chain 5 | Protein Coding |  | 46 | GC05M013745 | | 3.32 | https://www.genecards.org/cgi-bin/carddisp.pl?gene=DNAH5 |
| STK4 | Serine/Threonine Kinase 4 | Protein Coding |  | 55 | GC20P044966 | | 3.31 | https://www.genecards.org/cgi-bin/carddisp.pl?gene=STK4 |
| RFWD3 | Ring Finger And WD Repeat Domain 3 | Protein Coding |  | 46 | GC16M074656 | | 3.31 | https://www.genecards.org/cgi-bin/carddisp.pl?gene=RFWD3 |
| CHD7 | Chromodomain Helicase DNA Binding Protein 7 | Protein Coding |  | 52 | GC08P060678 | | 3.31 | https://www.genecards.org/cgi-bin/carddisp.pl?gene=CHD7 |
| RHOB | Ras Homolog Family Member B | Protein Coding |  | 48 | GC02P020447 | | 3.27 | https://www.genecards.org/cgi-bin/carddisp.pl?gene=RHOB |
| PRF1 | Perforin 1 | Protein Coding |  | 51 | GC10M070597 | | 3.27 | https://www.genecards.org/cgi-bin/carddisp.pl?gene=PRF1 |
| ALOX5 | Arachidonate 5-Lipoxygenase | Protein Coding |  | 54 | GC10P045338 | | 3.27 | https://www.genecards.org/cgi-bin/carddisp.pl?gene=ALOX5 |
| TATDN1 | TatD DNase Domain Containing 1 | Protein Coding |  | 42 | GC08M124488 | | 3.26 | https://www.genecards.org/cgi-bin/carddisp.pl?gene=TATDN1 |
| FCRL6 | Fc Receptor Like 6 | Protein Coding |  | 40 | GC01P159771 | | 3.26 | https://www.genecards.org/cgi-bin/carddisp.pl?gene=FCRL6 |
| SGK1 | Serum/Glucocorticoid Regulated Kinase 1 | Protein Coding |  | 55 | GC06M134169 | | 3.26 | https://www.genecards.org/cgi-bin/carddisp.pl?gene=SGK1 |
| BMP7 | Bone Morphogenetic Protein 7 | Protein Coding |  | 51 | GC20M057168 | | 3.26 | https://www.genecards.org/cgi-bin/carddisp.pl?gene=BMP7 |
| MALT1 | MALT1 Paracaspase | Protein Coding |  | 54 | GC18P058671 | | 3.25 | https://www.genecards.org/cgi-bin/carddisp.pl?gene=MALT1 |
| PIGR | Polymeric Immunoglobulin Receptor | Protein Coding |  | 47 | GC01M206928 | | 3.24 | https://www.genecards.org/cgi-bin/carddisp.pl?gene=PIGR |
| FANCD2 | FA Complementation Group D2 | Protein Coding |  | 52 | GC03P010026 | | 3.24 | https://www.genecards.org/cgi-bin/carddisp.pl?gene=FANCD2 |
| CGA | Glycoprotein Hormones, Alpha Polypeptide | Protein Coding |  | 49 | GC06M087085 | | 3.23 | https://www.genecards.org/cgi-bin/carddisp.pl?gene=CGA |
| PAXIP1 | PAX Interacting Protein 1 | Protein Coding |  | 43 | GC07M154943 | | 3.23 | https://www.genecards.org/cgi-bin/carddisp.pl?gene=PAXIP1 |
| IKBKB | Inhibitor Of Nuclear Factor Kappa B Kinase Subunit Beta | Protein Coding |  | 59 | GC08P042247 | | 3.23 | https://www.genecards.org/cgi-bin/carddisp.pl?gene=IKBKB |
| TNFSF13 | TNF Superfamily Member 13 | Protein Coding |  | 50 | GC17P007558 | | 3.23 | https://www.genecards.org/cgi-bin/carddisp.pl?gene=TNFSF13 |
| FASN | Fatty Acid Synthase | Protein Coding |  | 56 | GC17M082078 | | 3.22 | https://www.genecards.org/cgi-bin/carddisp.pl?gene=FASN |
| GJC2 | Gap Junction Protein Gamma 2 | Protein Coding |  | 47 | GC01P228150 | | 3.21 | https://www.genecards.org/cgi-bin/carddisp.pl?gene=GJC2 |
| DHDH | Dihydrodiol Dehydrogenase | Protein Coding |  | 42 | GC19P048933 | | 3.21 | https://www.genecards.org/cgi-bin/carddisp.pl?gene=DHDH |
| PLOD2 | Procollagen-Lysine,2-Oxoglutarate 5-Dioxygenase 2 | Protein Coding |  | 52 | GC03M146069 | | 3.17 | https://www.genecards.org/cgi-bin/carddisp.pl?gene=PLOD2 |
| OSM | Oncostatin M | Protein Coding |  | 49 | GC22M030262 | | 3.17 | https://www.genecards.org/cgi-bin/carddisp.pl?gene=OSM |
| HLA-DRB1 | Major Histocompatibility Complex, Class II, DR Beta 1 | Protein Coding |  | 52 | GC06M032578 | | 3.17 | https://www.genecards.org/cgi-bin/carddisp.pl?gene=HLA-DRB1 |
| HDGF | Heparin Binding Growth Factor | Protein Coding |  | 46 | GC01M156743 | | 3.16 | https://www.genecards.org/cgi-bin/carddisp.pl?gene=HDGF |
| TUSC2 | Tumor Suppressor 2, Mitochondrial Calcium Regulator | Protein Coding |  | 40 | GC03M050357 | | 3.16 | https://www.genecards.org/cgi-bin/carddisp.pl?gene=TUSC2 |
| CCR5 | C-C Motif Chemokine Receptor 5 (Gene/Pseudogene) | Protein Coding |  | 53 | GC03P046389 | | 3.16 | https://www.genecards.org/cgi-bin/carddisp.pl?gene=CCR5 |
| PI3 | Peptidase Inhibitor 3 | Protein Coding |  | 45 | GC20P045174 | | 3.15 | https://www.genecards.org/cgi-bin/carddisp.pl?gene=PI3 |
| IL9 | Interleukin 9 | Protein Coding |  | 50 | GC05M135891 | | 3.15 | https://www.genecards.org/cgi-bin/carddisp.pl?gene=IL9 |
| RAD51C | RAD51 Paralog C | Protein Coding |  | 50 | GC17P058692 | | 3.14 | https://www.genecards.org/cgi-bin/carddisp.pl?gene=RAD51C |
| BIRC7 | Baculoviral IAP Repeat Containing 7 | Protein Coding |  | 46 | GC20P063235 | | 3.14 | https://www.genecards.org/cgi-bin/carddisp.pl?gene=BIRC7 |
| BRIP1 | BRCA1 Interacting Protein C-Terminal Helicase 1 | Protein Coding |  | 54 | GC17M061679 | | 3.14 | https://www.genecards.org/cgi-bin/carddisp.pl?gene=BRIP1 |
| CD4 | CD4 Molecule | Protein Coding |  | 56 | GC12P006786 | | 3.13 | https://www.genecards.org/cgi-bin/carddisp.pl?gene=CD4 |
| EEF1A2 | Eukaryotic Translation Elongation Factor 1 Alpha 2 | Protein Coding |  | 52 | GC20M063488 | | 3.13 | https://www.genecards.org/cgi-bin/carddisp.pl?gene=EEF1A2 |
| DPH1 | Diphthamide Biosynthesis 1 | Protein Coding |  | 46 | GC17P002030 | | 3.13 | https://www.genecards.org/cgi-bin/carddisp.pl?gene=DPH1 |
| KMT2B | Lysine Methyltransferase 2B | Protein Coding |  | 45 | GC19P037189 | | 3.13 | https://www.genecards.org/cgi-bin/carddisp.pl?gene=KMT2B |
| FANCA | FA Complementation Group A | Protein Coding |  | 54 | GC16M089740 | | 3.13 | https://www.genecards.org/cgi-bin/carddisp.pl?gene=FANCA |
| TNFSF12 | TNF Superfamily Member 12 | Protein Coding |  | 46 | GC17P007829 | | 3.12 | https://www.genecards.org/cgi-bin/carddisp.pl?gene=TNFSF12 |
| ANO1 | Anoctamin 1 | Protein Coding |  | 47 | GC11P070078 | | 3.12 | https://www.genecards.org/cgi-bin/carddisp.pl?gene=ANO1 |
| MIR511 | MicroRNA 511 | RNA Gene |  | 15 | GC10P017845 | | 3.12 | https://www.genecards.org/cgi-bin/carddisp.pl?gene=MIR511 |
| GUSB | Glucuronidase Beta | Protein Coding |  | 54 | GC07M065960 | | 3.1 | https://www.genecards.org/cgi-bin/carddisp.pl?gene=GUSB |
| GUCY2C | Guanylate Cyclase 2C | Protein Coding |  | 52 | GC12M014612 | | 3.1 | https://www.genecards.org/cgi-bin/carddisp.pl?gene=GUCY2C |
| GCG | Glucagon | Protein Coding |  | 46 | GC02M162142 | | 3.09 | https://www.genecards.org/cgi-bin/carddisp.pl?gene=GCG |
| PRSS1 | Serine Protease 1 | Protein Coding |  | 52 | GC07P144338 | | 3.08 | https://www.genecards.org/cgi-bin/carddisp.pl?gene=PRSS1 |
| PROP1 | PROP Paired-Like Homeobox 1 | Protein Coding |  | 45 | GC05M177992 | | 3.08 | https://www.genecards.org/cgi-bin/carddisp.pl?gene=PROP1 |
| GGPS1 | Geranylgeranyl Diphosphate Synthase 1 | Protein Coding |  | 51 | GC01P235327 | | 3.06 | https://www.genecards.org/cgi-bin/carddisp.pl?gene=GGPS1 |
| NMBR | Neuromedin B Receptor | Protein Coding |  | 48 | GC06M142019 | | 3.06 | https://www.genecards.org/cgi-bin/carddisp.pl?gene=NMBR |
| TUBA4B | Tubulin Alpha 4b | Protein Coding |  | 31 | GC02P219253 | | 3.06 | https://www.genecards.org/cgi-bin/carddisp.pl?gene=TUBA4B |
| PKD1 | Polycystin 1, Transient Receptor Potential Channel Interacting | Protein Coding |  | 51 | GC16M002117 | | 3.05 | https://www.genecards.org/cgi-bin/carddisp.pl?gene=PKD1 |
| CHI3L1 | Chitinase 3 Like 1 | Protein Coding |  | 49 | GC01M203148 | | 3.04 | https://www.genecards.org/cgi-bin/carddisp.pl?gene=CHI3L1 |
| CEACAM3 | Carcinoembryonic Antigen Related Cell Adhesion Molecule 3 | Protein Coding |  | 46 | GC19P041796 | | 3.04 | https://www.genecards.org/cgi-bin/carddisp.pl?gene=CEACAM3 |
| CHRNA5 | Cholinergic Receptor Nicotinic Alpha 5 Subunit | Protein Coding |  | 51 | GC15P078565 | | 3.02 | https://www.genecards.org/cgi-bin/carddisp.pl?gene=CHRNA5 |
| COL5A1 | Collagen Type V Alpha 1 Chain | Protein Coding |  | 51 | GC09P134641 | | 3.02 | https://www.genecards.org/cgi-bin/carddisp.pl?gene=COL5A1 |
| CHRNA3 | Cholinergic Receptor Nicotinic Alpha 3 Subunit | Protein Coding |  | 50 | GC15M078594 | | 3.02 | https://www.genecards.org/cgi-bin/carddisp.pl?gene=CHRNA3 |
| BRS3 | Bombesin Receptor Subtype 3 | Protein Coding |  | 49 | GC0XP136482 | | 3.02 | https://www.genecards.org/cgi-bin/carddisp.pl?gene=BRS3 |
| NMB | Neuromedin B | Protein Coding |  | 48 | GC15M084655 | | 3.02 | https://www.genecards.org/cgi-bin/carddisp.pl?gene=NMB |
| GPSM2 | G Protein Signaling Modulator 2 | Protein Coding |  | 47 | GC01P108875 | | 3.02 | https://www.genecards.org/cgi-bin/carddisp.pl?gene=GPSM2 |
| INSL3 | Insulin Like 3 | Protein Coding |  | 46 | GC19M017788 | | 3.02 | https://www.genecards.org/cgi-bin/carddisp.pl?gene=INSL3 |
| COTL1 | Coactosin Like F-Actin Binding Protein 1 | Protein Coding |  | 46 | GC16M084599 | | 3.02 | https://www.genecards.org/cgi-bin/carddisp.pl?gene=COTL1 |
| MYO18B | Myosin XVIIIB | Protein Coding |  | 45 | GC22P025742 | | 3.02 | https://www.genecards.org/cgi-bin/carddisp.pl?gene=MYO18B |
| SEMA3B | Semaphorin 3B | Protein Coding |  | 45 | GC03P050267 | | 3.02 | https://www.genecards.org/cgi-bin/carddisp.pl?gene=SEMA3B |
| RBM5 | RNA Binding Motif Protein 5 | Protein Coding |  | 45 | GC03P050101 | | 3.02 | https://www.genecards.org/cgi-bin/carddisp.pl?gene=RBM5 |
| SCUBE3 | Signal Peptide, CUB Domain And EGF Like Domain Containing 3 | Protein Coding |  | 44 | GC06P043119 | | 3.02 | https://www.genecards.org/cgi-bin/carddisp.pl?gene=SCUBE3 |
| GUCA2A | Guanylate Cyclase Activator 2A | Protein Coding |  | 43 | GC01M042162 | | 3.02 | https://www.genecards.org/cgi-bin/carddisp.pl?gene=GUCA2A |
| MRAP | Melanocortin 2 Receptor Accessory Protein | Protein Coding |  | 43 | GC21P032291 | | 3.02 | https://www.genecards.org/cgi-bin/carddisp.pl?gene=MRAP |
| KLHL40 | Kelch Like Family Member 40 | Protein Coding |  | 43 | GC03P042685 | | 3.02 | https://www.genecards.org/cgi-bin/carddisp.pl?gene=KLHL40 |
| DLGAP2 | DLG Associated Protein 2 | Protein Coding |  | 43 | GC08P000739 | | 3.02 | https://www.genecards.org/cgi-bin/carddisp.pl?gene=DLGAP2 |
| MYEF2 | Myelin Expression Factor 2 | Protein Coding |  | 41 | GC15M048134 | | 3.02 | https://www.genecards.org/cgi-bin/carddisp.pl?gene=MYEF2 |
| ZNF461 | Zinc Finger Protein 461 | Protein Coding |  | 41 | GC19M039145 | | 3.02 | https://www.genecards.org/cgi-bin/carddisp.pl?gene=ZNF461 |
| ZNF793 | Zinc Finger Protein 793 | Protein Coding |  | 38 | GC19P037506 | | 3.02 | https://www.genecards.org/cgi-bin/carddisp.pl?gene=ZNF793 |
| GUCY1B2 | Guanylate Cyclase 1 Soluble Subunit Beta 2 (Pseudogene) | Pseudogene |  | 37 | GC13M050994 | | 3.02 | https://www.genecards.org/cgi-bin/carddisp.pl?gene=GUCY1B2 |
| C20orf85 | Chromosome 20 Open Reading Frame 85 | Protein Coding |  | 33 | GC20P058150 | | 3.02 | https://www.genecards.org/cgi-bin/carddisp.pl?gene=C20orf85 |
| CT83 | Cancer/Testis Antigen 83 | Protein Coding |  | 32 | GC0XM116461 | | 3.02 | https://www.genecards.org/cgi-bin/carddisp.pl?gene=CT83 |
| NEXN-AS1 | NEXN Antisense RNA 1 | RNA Gene |  | 25 | GC01M077881 | | 3.02 | https://www.genecards.org/cgi-bin/carddisp.pl?gene=NEXN-AS1 |
| LINC00313 | Long Intergenic Non-Protein Coding RNA 313 | RNA Gene |  | 23 | GC21M043462 | | 3.02 | https://www.genecards.org/cgi-bin/carddisp.pl?gene=LINC00313 |
| GNAS-AS1 | GNAS Antisense RNA 1 | RNA Gene |  | 23 | GC20M058838 | | 3.02 | https://www.genecards.org/cgi-bin/carddisp.pl?gene=GNAS-AS1 |
| MIR218-2 | MicroRNA 218-2 | RNA Gene |  | 20 | GC05M168768 | | 3.02 | https://www.genecards.org/cgi-bin/carddisp.pl?gene=MIR218-2 |
| LINC00342 | Long Intergenic Non-Protein Coding RNA 342 | RNA Gene |  | 18 | GC02M095807 | | 3.02 | https://www.genecards.org/cgi-bin/carddisp.pl?gene=LINC00342 |
| CACNA1G-AS1 | CACNA1G Antisense RNA 1 | RNA Gene |  | 18 | GC17M050556 | | 3.02 | https://www.genecards.org/cgi-bin/carddisp.pl?gene=CACNA1G-AS1 |
| GAS6-AS1 | GAS6 Antisense RNA 1 | RNA Gene |  | 18 | GC13P113815 | | 3.02 | https://www.genecards.org/cgi-bin/carddisp.pl?gene=GAS6-AS1 |
| LINC00857 | Long Intergenic Non-Protein Coding RNA 857 | RNA Gene |  | 17 | GC10P080207 | | 3.02 | https://www.genecards.org/cgi-bin/carddisp.pl?gene=LINC00857 |
| LINC00880 | Long Intergenic Non-Protein Coding RNA 880 | RNA Gene |  | 17 | GC03M157081 | | 3.02 | https://www.genecards.org/cgi-bin/carddisp.pl?gene=LINC00880 |
| PPIEL | Peptidylprolyl Isomerase E Like Pseudogene | Pseudogene |  | 17 | GC01M039522 | | 3.02 | https://www.genecards.org/cgi-bin/carddisp.pl?gene=PPIEL |
| LINC01433 | Long Intergenic Non-Protein Coding RNA 1433 | RNA Gene |  | 15 | GC20P004193 | | 3.02 | https://www.genecards.org/cgi-bin/carddisp.pl?gene=LINC01433 |
| LINC01186 | Long Intergenic Non-Protein Coding RNA 1186 | RNA Gene |  | 15 | GC0XM046325 | | 3.02 | https://www.genecards.org/cgi-bin/carddisp.pl?gene=LINC01186 |
| TTN-AS1 | TTN Antisense RNA 1 | RNA Gene |  | 15 | GC02P178521 | | 3.02 | https://www.genecards.org/cgi-bin/carddisp.pl?gene=TTN-AS1 |
| LINC00968 | Long Intergenic Non-Protein Coding RNA 968 | RNA Gene |  | 14 | GC08M056496 | | 3.02 | https://www.genecards.org/cgi-bin/carddisp.pl?gene=LINC00968 |
| KCNMB2-AS1 | KCNMB2 Antisense RNA 1 | RNA Gene |  | 13 | GC03M178525 | | 3.02 | https://www.genecards.org/cgi-bin/carddisp.pl?gene=KCNMB2-AS1 |
| LINC01589 | Long Intergenic Non-Protein Coding RNA 1589 | RNA Gene |  | 13 | GC22M045604 | | 3.02 | https://www.genecards.org/cgi-bin/carddisp.pl?gene=LINC01589 |
| SGO1-AS1 | SGO1 Antisense RNA 1 | RNA Gene |  | 13 | GC03P020174 | | 3.02 | https://www.genecards.org/cgi-bin/carddisp.pl?gene=SGO1-AS1 |
| LINC01627 | Long Intergenic Non-Protein Coding RNA 1627 | RNA Gene |  | 12 | GC09M037384 | | 3.02 | https://www.genecards.org/cgi-bin/carddisp.pl?gene=LINC01627 |
| LINC02042 | Long Intergenic Non-Protein Coding RNA 2042 | RNA Gene |  | 12 | GC03M112737 | | 3.02 | https://www.genecards.org/cgi-bin/carddisp.pl?gene=LINC02042 |
| LINC02412 | Long Intergenic Non-Protein Coding RNA 2412 | RNA Gene |  | 12 | GC12P093175 | | 3.02 | https://www.genecards.org/cgi-bin/carddisp.pl?gene=LINC02412 |
| LCAL1 | Lung Cancer Associated LncRNA 1 | RNA Gene |  | 11 | GC06M079307 | | 3.02 | https://www.genecards.org/cgi-bin/carddisp.pl?gene=LCAL1 |
| LINC01502 | Long Intergenic Non-Protein Coding RNA 1502 | RNA Gene |  | 11 | GC09P135574 | | 3.02 | https://www.genecards.org/cgi-bin/carddisp.pl?gene=LINC01502 |
| ENSG00000255224 |  | RNA Gene |  | 10 | GC08M144078 | | 3.02 | https://www.genecards.org/cgi-bin/carddisp.pl?gene=ENSG00000255224 |
| MUC20-OT1 | MUC20 Overlapping Transcript | RNA Gene |  | 9 | GC03P195660 | | 3.02 | https://www.genecards.org/cgi-bin/carddisp.pl?gene=MUC20-OT1 |
| ENSG00000223859 |  | RNA Gene |  | 9 | GC02P067041 | | 3.02 | https://www.genecards.org/cgi-bin/carddisp.pl?gene=ENSG00000223859 |
| ENSG00000279080 |  | RNA Gene |  | 7 | GC22P038130 | | 3.02 | https://www.genecards.org/cgi-bin/carddisp.pl?gene=ENSG00000279080 |
| ENSG00000285095 |  | RNA Gene |  | 7 | GC18P032470 | | 3.02 | https://www.genecards.org/cgi-bin/carddisp.pl?gene=ENSG00000285095 |
| ENSG00000249592 |  | RNA Gene |  | 7 | GC04M000757 | | 3.02 | https://www.genecards.org/cgi-bin/carddisp.pl?gene=ENSG00000249592 |
| ENSG00000257337 |  | RNA Gene |  | 7 | GC12M053015 | | 3.02 | https://www.genecards.org/cgi-bin/carddisp.pl?gene=ENSG00000257337 |
| ENSG00000249738 |  | RNA Gene |  | 6 | GC05P159311 | | 3.02 | https://www.genecards.org/cgi-bin/carddisp.pl?gene=ENSG00000249738 |
| SMARCA4 | SWI/SNF Related, Matrix Associated, Actin Dependent Regulator Of Chromatin, Subfamily A, Member 4 | Protein Coding |  | 57 | GC19P010932 | | 3.02 | https://www.genecards.org/cgi-bin/carddisp.pl?gene=SMARCA4 |
| LDLR | Low Density Lipoprotein Receptor | Protein Coding |  | 56 | GC19P011061 | | 3.02 | https://www.genecards.org/cgi-bin/carddisp.pl?gene=LDLR |
| IRF3 | Interferon Regulatory Factor 3 | Protein Coding |  | 53 | GC19M049659 | | 3.01 | https://www.genecards.org/cgi-bin/carddisp.pl?gene=IRF3 |
| RPS3 | Ribosomal Protein S3 | Protein Coding |  | 49 | GC11P075596 | | 3 | https://www.genecards.org/cgi-bin/carddisp.pl?gene=RPS3 |
| HDAC1 | Histone Deacetylase 1 | Protein Coding |  | 56 | GC01P032260 | | 2.98 | https://www.genecards.org/cgi-bin/carddisp.pl?gene=HDAC1 |
| ATRX | ATRX Chromatin Remodeler | Protein Coding |  | 52 | GC0XM077504 | | 2.97 | https://www.genecards.org/cgi-bin/carddisp.pl?gene=ATRX |
| RPS27A | Ribosomal Protein S27a | Protein Coding |  | 50 | GC02P055231 | | 2.96 | https://www.genecards.org/cgi-bin/carddisp.pl?gene=RPS27A |
| MVK | Mevalonate Kinase | Protein Coding |  | 55 | GC12P109573 | | 2.96 | https://www.genecards.org/cgi-bin/carddisp.pl?gene=MVK |
| BPI | Bactericidal Permeability Increasing Protein | Protein Coding |  | 47 | GC20P038304 | | 2.95 | https://www.genecards.org/cgi-bin/carddisp.pl?gene=BPI |
| MMP13 | Matrix Metallopeptidase 13 | Protein Coding |  | 57 | GC11M102942 | | 2.94 | https://www.genecards.org/cgi-bin/carddisp.pl?gene=MMP13 |
| TFPI | Tissue Factor Pathway Inhibitor | Protein Coding |  | 51 | GC02M187464 | | 2.94 | https://www.genecards.org/cgi-bin/carddisp.pl?gene=TFPI |
| RAD21 | RAD21 Cohesin Complex Component | Protein Coding |  | 51 | GC08M116846 | | 2.94 | https://www.genecards.org/cgi-bin/carddisp.pl?gene=RAD21 |
| XDH | Xanthine Dehydrogenase | Protein Coding |  | 54 | GC02M031294 | | 2.93 | https://www.genecards.org/cgi-bin/carddisp.pl?gene=XDH |
| KRT10 | Keratin 10 | Protein Coding |  | 48 | GC17M040818 | | 2.93 | https://www.genecards.org/cgi-bin/carddisp.pl?gene=KRT10 |
| TNFRSF8 | TNF Receptor Superfamily Member 8 | Protein Coding |  | 50 | GC01P012123 | | 2.92 | https://www.genecards.org/cgi-bin/carddisp.pl?gene=TNFRSF8 |
| PRDX5 | Peroxiredoxin 5 | Protein Coding |  | 52 | GC11P064317 | | 2.91 | https://www.genecards.org/cgi-bin/carddisp.pl?gene=PRDX5 |
| LYN | LYN Proto-Oncogene, Src Family Tyrosine Kinase | Protein Coding |  | 57 | GC08P055879 | | 2.9 | https://www.genecards.org/cgi-bin/carddisp.pl?gene=LYN |
| ALPP | Alkaline Phosphatase, Placental | Protein Coding |  | 51 | GC02P232378 | | 2.9 | https://www.genecards.org/cgi-bin/carddisp.pl?gene=ALPP |
| CRYAB | Crystallin Alpha B | Protein Coding |  | 51 | GC11M111908 | | 2.89 | https://www.genecards.org/cgi-bin/carddisp.pl?gene=CRYAB |
| LCN2 | Lipocalin 2 | Protein Coding |  | 50 | GC09P128149 | | 2.89 | https://www.genecards.org/cgi-bin/carddisp.pl?gene=LCN2 |
| ITGAV | Integrin Subunit Alpha V | Protein Coding |  | 53 | GC02P186589 | | 2.87 | https://www.genecards.org/cgi-bin/carddisp.pl?gene=ITGAV |
| HNRNPA1 | Heterogeneous Nuclear Ribonucleoprotein A1 | Protein Coding |  | 52 | GC12P054280 | | 2.86 | https://www.genecards.org/cgi-bin/carddisp.pl?gene=HNRNPA1 |
| EPAS1 | Endothelial PAS Domain Protein 1 | Protein Coding |  | 54 | GC02P046293 | | 2.85 | https://www.genecards.org/cgi-bin/carddisp.pl?gene=EPAS1 |
| RPL4 | Ribosomal Protein L4 | Protein Coding |  | 46 | GC15M066498 | | 2.85 | https://www.genecards.org/cgi-bin/carddisp.pl?gene=RPL4 |
| PRTN3 | Proteinase 3 | Protein Coding |  | 51 | GC19P000840 | | 2.83 | https://www.genecards.org/cgi-bin/carddisp.pl?gene=PRTN3 |
| CLTC | Clathrin Heavy Chain | Protein Coding |  | 54 | GC17P059619 | | 2.83 | https://www.genecards.org/cgi-bin/carddisp.pl?gene=CLTC |
| PRKCE | Protein Kinase C Epsilon | Protein Coding |  | 57 | GC02P045617 | | 2.83 | https://www.genecards.org/cgi-bin/carddisp.pl?gene=PRKCE |
| CFP | Complement Factor Properdin | Protein Coding |  | 50 | GC0XM047624 | | 2.82 | https://www.genecards.org/cgi-bin/carddisp.pl?gene=CFP |
| MBP | Myelin Basic Protein | Protein Coding |  | 51 | GC18M076978 | | 2.82 | https://www.genecards.org/cgi-bin/carddisp.pl?gene=MBP |
| CASP4 | Caspase 4 | Protein Coding |  | 52 | GC11M104942 | | 2.81 | https://www.genecards.org/cgi-bin/carddisp.pl?gene=CASP4 |
| FOXM1 | Forkhead Box M1 | Protein Coding |  | 50 | GC12M002857 | | 2.81 | https://www.genecards.org/cgi-bin/carddisp.pl?gene=FOXM1 |
| ITGB2 | Integrin Subunit Beta 2 | Protein Coding |  | 57 | GC21M044885 | | 2.81 | https://www.genecards.org/cgi-bin/carddisp.pl?gene=ITGB2 |
| SMAD7 | SMAD Family Member 7 | Protein Coding |  | 49 | GC18M048919 | | 2.8 | https://www.genecards.org/cgi-bin/carddisp.pl?gene=SMAD7 |
| SLC35C1 | Solute Carrier Family 35 Member C1 | Protein Coding |  | 45 | GC11P045926 | | 2.8 | https://www.genecards.org/cgi-bin/carddisp.pl?gene=SLC35C1 |
| MSH5 | MutS Homolog 5 | Protein Coding |  | 47 | GC06P032514 | | 2.79 | https://www.genecards.org/cgi-bin/carddisp.pl?gene=MSH5 |
| PALB2 | Partner And Localizer Of BRCA2 | Protein Coding |  | 49 | GC16M023614 | | 2.79 | https://www.genecards.org/cgi-bin/carddisp.pl?gene=PALB2 |
| S100A1 | S100 Calcium Binding Protein A1 | Protein Coding |  | 48 | GC01P153627 | | 2.79 | https://www.genecards.org/cgi-bin/carddisp.pl?gene=S100A1 |
| DEFB1 | Defensin Beta 1 | Protein Coding |  | 45 | GC08M006870 | | 2.79 | https://www.genecards.org/cgi-bin/carddisp.pl?gene=DEFB1 |
| CCR1 | C-C Motif Chemokine Receptor 1 | Protein Coding |  | 52 | GC03M046218 | | 2.78 | https://www.genecards.org/cgi-bin/carddisp.pl?gene=CCR1 |
| FOSL1 | FOS Like 1, AP-1 Transcription Factor Subunit | Protein Coding |  | 50 | GC11M065909 | | 2.78 | https://www.genecards.org/cgi-bin/carddisp.pl?gene=FOSL1 |
| CCR2 | C-C Motif Chemokine Receptor 2 | Protein Coding |  | 51 | GC03P046356 | | 2.77 | https://www.genecards.org/cgi-bin/carddisp.pl?gene=CCR2 |
| PIK3CD | Phosphatidylinositol-4,5-Bisphosphate 3-Kinase Catalytic Subunit Delta | Protein Coding |  | 60 | GC01P009711 | | 2.76 | https://www.genecards.org/cgi-bin/carddisp.pl?gene=PIK3CD |
| DNASE1 | Deoxyribonuclease 1 | Protein Coding |  | 47 | GC16P003611 | | 2.75 | https://www.genecards.org/cgi-bin/carddisp.pl?gene=DNASE1 |
| F8 | Coagulation Factor VIII | Protein Coding |  | 52 | GC0XM154835 | | 2.75 | https://www.genecards.org/cgi-bin/carddisp.pl?gene=F8 |
| SLC9A3R1 | SLC9A3 Regulator 1 | Protein Coding |  | 52 | GC17P074749 | | 2.75 | https://www.genecards.org/cgi-bin/carddisp.pl?gene=SLC9A3R1 |
| SERPINA7 | Serpin Family A Member 7 | Protein Coding |  | 45 | GC0XM106032 | | 2.75 | https://www.genecards.org/cgi-bin/carddisp.pl?gene=SERPINA7 |
| EDNRA | Endothelin Receptor Type A | Protein Coding |  | 56 | GC04P147480 | | 2.73 | https://www.genecards.org/cgi-bin/carddisp.pl?gene=EDNRA |
| WAS | WASP Actin Nucleation Promoting Factor | Protein Coding |  | 55 | GC0XP048676 | | 2.73 | https://www.genecards.org/cgi-bin/carddisp.pl?gene=WAS |
| RUNX3 | RUNX Family Transcription Factor 3 | Protein Coding |  | 49 | GC01M024899 | | 2.73 | https://www.genecards.org/cgi-bin/carddisp.pl?gene=RUNX3 |
| FLI1 | Fli-1 Proto-Oncogene, ETS Transcription Factor | Protein Coding |  | 56 | GC11P128686 | | 2.73 | https://www.genecards.org/cgi-bin/carddisp.pl?gene=FLI1 |
| TGIF1 | TGFB Induced Factor Homeobox 1 | Protein Coding |  | 52 | GC18P003411 | | 2.73 | https://www.genecards.org/cgi-bin/carddisp.pl?gene=TGIF1 |
| TPM3 | Tropomyosin 3 | Protein Coding |  | 53 | GC01M154127 | | 2.71 | https://www.genecards.org/cgi-bin/carddisp.pl?gene=TPM3 |
| CD80 | CD80 Molecule | Protein Coding |  | 48 | GC03M119524 | | 2.71 | https://www.genecards.org/cgi-bin/carddisp.pl?gene=CD80 |
| AFF4 | AF4/FMR2 Family Member 4 | Protein Coding |  | 47 | GC05M132875 | | 2.71 | https://www.genecards.org/cgi-bin/carddisp.pl?gene=AFF4 |
| MIRLET7A1 | MicroRNA Let-7a-1 | RNA Gene |  | 21 | GC09P094175 | | 2.7 | https://www.genecards.org/cgi-bin/carddisp.pl?gene=MIRLET7A1 |
| GREM1 | Gremlin 1, DAN Family BMP Antagonist | Protein Coding |  | 51 | GC15P032717 | | 2.7 | https://www.genecards.org/cgi-bin/carddisp.pl?gene=GREM1 |
| ADCY10 | Adenylate Cyclase 10 | Protein Coding |  | 51 | GC01M167778 | | 2.69 | https://www.genecards.org/cgi-bin/carddisp.pl?gene=ADCY10 |
| LPL | Lipoprotein Lipase | Protein Coding |  | 57 | GC08P019841 | | 2.69 | https://www.genecards.org/cgi-bin/carddisp.pl?gene=LPL |
| TRG | T Cell Receptor Gamma Locus | Protein Coding |  | 15 | GC07M038240 | | 2.69 | https://www.genecards.org/cgi-bin/carddisp.pl?gene=TRG |
| APOH | Apolipoprotein H | Protein Coding |  | 50 | GC17M066212 | | 2.69 | https://www.genecards.org/cgi-bin/carddisp.pl?gene=APOH |
| MYH2 | Myosin Heavy Chain 2 | Protein Coding |  | 51 | GC17M010521 | | 2.68 | https://www.genecards.org/cgi-bin/carddisp.pl?gene=MYH2 |
| DUOX2 | Dual Oxidase 2 | Protein Coding |  | 49 | GC15M045092 | | 2.68 | https://www.genecards.org/cgi-bin/carddisp.pl?gene=DUOX2 |
| IL13RA2 | Interleukin 13 Receptor Subunit Alpha 2 | Protein Coding |  | 46 | GC0XM115003 | | 2.66 | https://www.genecards.org/cgi-bin/carddisp.pl?gene=IL13RA2 |
| SMARCE1 | SWI/SNF Related, Matrix Associated, Actin Dependent Regulator Of Chromatin, Subfamily E, Member 1 | Protein Coding |  | 51 | GC17M040624 | | 2.65 | https://www.genecards.org/cgi-bin/carddisp.pl?gene=SMARCE1 |
| IDO1 | Indoleamine 2,3-Dioxygenase 1 | Protein Coding |  | 51 | GC08P039891 | | 2.64 | https://www.genecards.org/cgi-bin/carddisp.pl?gene=IDO1 |
| NIPBL | NIPBL Cohesin Loading Factor | Protein Coding |  | 46 | GC05P036876 | | 2.64 | https://www.genecards.org/cgi-bin/carddisp.pl?gene=NIPBL |
| UMPS | Uridine Monophosphate Synthetase | Protein Coding |  | 51 | GC03P124730 | | 2.64 | https://www.genecards.org/cgi-bin/carddisp.pl?gene=UMPS |
| S100A9 | S100 Calcium Binding Protein A9 | Protein Coding |  | 50 | GC01P153357 | | 2.63 | https://www.genecards.org/cgi-bin/carddisp.pl?gene=S100A9 |
| NGF | Nerve Growth Factor | Protein Coding |  | 57 | GC01M115285 | | 2.63 | https://www.genecards.org/cgi-bin/carddisp.pl?gene=NGF |
| NAT2 | N-Acetyltransferase 2 | Protein Coding |  | 49 | GC08P018391 | | 2.62 | https://www.genecards.org/cgi-bin/carddisp.pl?gene=NAT2 |
| ANPEP | Alanyl Aminopeptidase, Membrane | Protein Coding |  | 54 | GC15M089784 | | 2.62 | https://www.genecards.org/cgi-bin/carddisp.pl?gene=ANPEP |
| RAC2 | Rac Family Small GTPase 2 | Protein Coding |  | 57 | GC22M037227 | | 2.62 | https://www.genecards.org/cgi-bin/carddisp.pl?gene=RAC2 |
| SBDS | SBDS Ribosome Maturation Factor | Protein Coding |  | 48 | GC07M066987 | | 2.62 | https://www.genecards.org/cgi-bin/carddisp.pl?gene=SBDS |
| LPO | Lactoperoxidase | Protein Coding |  | 45 | GC17P058218 | | 2.61 | https://www.genecards.org/cgi-bin/carddisp.pl?gene=LPO |
| ADAMTS13 | ADAM Metallopeptidase With Thrombospondin Type 1 Motif 13 | Protein Coding |  | 53 | GC09P133414 | | 2.61 | https://www.genecards.org/cgi-bin/carddisp.pl?gene=ADAMTS13 |
| GYS1 | Glycogen Synthase 1 | Protein Coding |  | 56 | GC19M048970 | | 2.61 | https://www.genecards.org/cgi-bin/carddisp.pl?gene=GYS1 |
| COL17A1 | Collagen Type XVII Alpha 1 Chain | Protein Coding |  | 50 | GC10M104031 | | 2.61 | https://www.genecards.org/cgi-bin/carddisp.pl?gene=COL17A1 |
| MC3R | Melanocortin 3 Receptor | Protein Coding |  | 48 | GC20P056248 | | 2.61 | https://www.genecards.org/cgi-bin/carddisp.pl?gene=MC3R |
| ITGA5 | Integrin Subunit Alpha 5 | Protein Coding |  | 54 | GC12M054396 | | 2.6 | https://www.genecards.org/cgi-bin/carddisp.pl?gene=ITGA5 |
| RECQL4 | RecQ Like Helicase 4 | Protein Coding |  | 48 | GC08M144512 | | 2.6 | https://www.genecards.org/cgi-bin/carddisp.pl?gene=RECQL4 |
| CFL1 | Cofilin 1 | Protein Coding |  | 51 | GC11M065823 | | 2.6 | https://www.genecards.org/cgi-bin/carddisp.pl?gene=CFL1 |
| BCR | BCR Activator Of RhoGEF And GTPase | Protein Coding |  | 59 | GC22P023179 | | 2.59 | https://www.genecards.org/cgi-bin/carddisp.pl?gene=BCR |
| IRF8 | Interferon Regulatory Factor 8 | Protein Coding |  | 51 | GC16P085898 | | 2.59 | https://www.genecards.org/cgi-bin/carddisp.pl?gene=IRF8 |
| F7 | Coagulation Factor VII | Protein Coding |  | 53 | GC13P113105 | | 2.59 | https://www.genecards.org/cgi-bin/carddisp.pl?gene=F7 |
| LCK | LCK Proto-Oncogene, Src Family Tyrosine Kinase | Protein Coding |  | 60 | GC01P032251 | | 2.57 | https://www.genecards.org/cgi-bin/carddisp.pl?gene=LCK |
| GAST | Gastrin | Protein Coding |  | 46 | GC17P041712 | | 2.57 | https://www.genecards.org/cgi-bin/carddisp.pl?gene=GAST |
| CRNDE | Colorectal Neoplasia Differentially Expressed | Protein Coding |  | 23 | GC16M054845 | | 2.57 | https://www.genecards.org/cgi-bin/carddisp.pl?gene=CRNDE |
| HNRNPK | Heterogeneous Nuclear Ribonucleoprotein K | Protein Coding |  | 51 | GC09M083969 | | 2.56 | https://www.genecards.org/cgi-bin/carddisp.pl?gene=HNRNPK |
| KPTN | Kaptin, Actin Binding Protein | Protein Coding |  | 43 | GC19M047475 | | 2.55 | https://www.genecards.org/cgi-bin/carddisp.pl?gene=KPTN |
| JAG1 | Jagged Canonical Notch Ligand 1 | Protein Coding |  | 56 | GC20M010637 | | 2.55 | https://www.genecards.org/cgi-bin/carddisp.pl?gene=JAG1 |
| AVPR2 | Arginine Vasopressin Receptor 2 | Protein Coding |  | 55 | GC0XP153902 | | 2.55 | https://www.genecards.org/cgi-bin/carddisp.pl?gene=AVPR2 |
| NEFL | Neurofilament Light | Protein Coding |  | 52 | GC08M024950 | | 2.55 | https://www.genecards.org/cgi-bin/carddisp.pl?gene=NEFL |
| DAB2 | DAB Adaptor Protein 2 | Protein Coding |  | 49 | GC05M039371 | | 2.55 | https://www.genecards.org/cgi-bin/carddisp.pl?gene=DAB2 |
| PYY | Peptide YY | Protein Coding |  | 49 | GC17M043952 | | 2.55 | https://www.genecards.org/cgi-bin/carddisp.pl?gene=PYY |
| GOPC | Golgi Associated PDZ And Coiled-Coil Motif Containing | Protein Coding |  | 48 | GC06M117560 | | 2.55 | https://www.genecards.org/cgi-bin/carddisp.pl?gene=GOPC |
| CDCA7L | Cell Division Cycle Associated 7 Like | Protein Coding |  | 46 | GC07M021900 | | 2.55 | https://www.genecards.org/cgi-bin/carddisp.pl?gene=CDCA7L |
| TRD | T Cell Receptor Delta Locus | Protein Coding |  | 14 | GC14P022425 | | 2.55 | https://www.genecards.org/cgi-bin/carddisp.pl?gene=TRD |
| FOXF1 | Forkhead Box F1 | Protein Coding |  | 48 | GC16P086510 | | 2.54 | https://www.genecards.org/cgi-bin/carddisp.pl?gene=FOXF1 |
| CYP3A4 | Cytochrome P450 Family 3 Subfamily A Member 4 | Protein Coding |  | 56 | GC07M099759 | | 2.54 | https://www.genecards.org/cgi-bin/carddisp.pl?gene=CYP3A4 |
| OFD1 | OFD1 Centriole And Centriolar Satellite Protein | Protein Coding |  | 48 | GC0XP013752 | | 2.54 | https://www.genecards.org/cgi-bin/carddisp.pl?gene=OFD1 |
| CD209 | CD209 Molecule | Protein Coding |  | 48 | GC19M007804 | | 2.53 | https://www.genecards.org/cgi-bin/carddisp.pl?gene=CD209 |
| MAP3K20 | Mitogen-Activated Protein Kinase Kinase Kinase 20 | Protein Coding |  | 43 | GC02P173076 | | 2.53 | https://www.genecards.org/cgi-bin/carddisp.pl?gene=MAP3K20 |
| ENG | Endoglin | Protein Coding |  | 53 | GC09M127815 | | 2.53 | https://www.genecards.org/cgi-bin/carddisp.pl?gene=ENG |
| KRT20 | Keratin 20 | Protein Coding |  | 47 | GC17M040875 | | 2.52 | https://www.genecards.org/cgi-bin/carddisp.pl?gene=KRT20 |
| CD3D | CD3d Molecule | Protein Coding |  | 54 | GC11M118338 | | 2.52 | https://www.genecards.org/cgi-bin/carddisp.pl?gene=CD3D |
| UNG | Uracil DNA Glycosylase | Protein Coding |  | 53 | GC12P109097 | | 2.52 | https://www.genecards.org/cgi-bin/carddisp.pl?gene=UNG |
| EEF1A1 | Eukaryotic Translation Elongation Factor 1 Alpha 1 | Protein Coding |  | 50 | GC06M073515 | | 2.51 | https://www.genecards.org/cgi-bin/carddisp.pl?gene=EEF1A1 |
| CTSG | Cathepsin G | Protein Coding |  | 51 | GC14M024573 | | 2.51 | https://www.genecards.org/cgi-bin/carddisp.pl?gene=CTSG |
| CYP1B1 | Cytochrome P450 Family 1 Subfamily B Member 1 | Protein Coding |  | 56 | GC02M038034 | | 2.5 | https://www.genecards.org/cgi-bin/carddisp.pl?gene=CYP1B1 |
| IFITM3 | Interferon Induced Transmembrane Protein 3 | Protein Coding |  | 47 | GC11M000319 | | 2.5 | https://www.genecards.org/cgi-bin/carddisp.pl?gene=IFITM3 |
| KRT8 | Keratin 8 | Protein Coding |  | 53 | GC12M052897 | | 2.49 | https://www.genecards.org/cgi-bin/carddisp.pl?gene=KRT8 |
| LGALS1 | Galectin 1 | Protein Coding |  | 49 | GC22P037675 | | 2.48 | https://www.genecards.org/cgi-bin/carddisp.pl?gene=LGALS1 |
| CD276 | CD276 Molecule | Protein Coding |  | 46 | GC15P073683 | | 2.48 | https://www.genecards.org/cgi-bin/carddisp.pl?gene=CD276 |
| RELB | RELB Proto-Oncogene, NF-KB Subunit | Protein Coding |  | 50 | GC19P045002 | | 2.48 | https://www.genecards.org/cgi-bin/carddisp.pl?gene=RELB |
| CAMK2G | Calcium/Calmodulin Dependent Protein Kinase II Gamma | Protein Coding |  | 53 | GC10M073812 | | 2.48 | https://www.genecards.org/cgi-bin/carddisp.pl?gene=CAMK2G |
| LTBP1 | Latent Transforming Growth Factor Beta Binding Protein 1 | Protein Coding |  | 50 | GC02P032946 | | 2.47 | https://www.genecards.org/cgi-bin/carddisp.pl?gene=LTBP1 |
| UBC | Ubiquitin C | Protein Coding |  | 48 | GC12M124911 | | 2.47 | https://www.genecards.org/cgi-bin/carddisp.pl?gene=UBC |
| IRAK1 | Interleukin 1 Receptor Associated Kinase 1 | Protein Coding |  | 55 | GC0XM154010 | | 2.47 | https://www.genecards.org/cgi-bin/carddisp.pl?gene=IRAK1 |
| SPARC | Secreted Protein Acidic And Cysteine Rich | Protein Coding |  | 57 | GC05M151639 | | 2.47 | https://www.genecards.org/cgi-bin/carddisp.pl?gene=SPARC |
| IL32 | Interleukin 32 | Protein Coding |  | 46 | GC16P003831 | | 2.47 | https://www.genecards.org/cgi-bin/carddisp.pl?gene=IL32 |
| MTAP | Methylthioadenosine Phosphorylase | Protein Coding |  | 54 | GC09P021792 | | 2.46 | https://www.genecards.org/cgi-bin/carddisp.pl?gene=MTAP |
| FLT3LG | Fms Related Tyrosine Kinase 3 Ligand | Protein Coding |  | 46 | GC19P049475 | | 2.46 | https://www.genecards.org/cgi-bin/carddisp.pl?gene=FLT3LG |
| ALOX5AP | Arachidonate 5-Lipoxygenase Activating Protein | Protein Coding |  | 50 | GC13P030713 | | 2.45 | https://www.genecards.org/cgi-bin/carddisp.pl?gene=ALOX5AP |
| PTPN6 | Protein Tyrosine Phosphatase Non-Receptor Type 6 | Protein Coding |  | 56 | GC12P007636 | | 2.45 | https://www.genecards.org/cgi-bin/carddisp.pl?gene=PTPN6 |
| BCHE | Butyrylcholinesterase | Protein Coding |  | 55 | GC03M165772 | | 2.45 | https://www.genecards.org/cgi-bin/carddisp.pl?gene=BCHE |
| RNF125 | Ring Finger Protein 125 | Protein Coding |  | 46 | GC18P032020 | | 2.44 | https://www.genecards.org/cgi-bin/carddisp.pl?gene=RNF125 |
| PPARD | Peroxisome Proliferator Activated Receptor Delta | Protein Coding |  | 54 | GC06P043121 | | 2.44 | https://www.genecards.org/cgi-bin/carddisp.pl?gene=PPARD |
| CREBBP | CREB Binding Protein | Protein Coding |  | 59 | GC16M003726 | | 2.44 | https://www.genecards.org/cgi-bin/carddisp.pl?gene=CREBBP |
| TLR7 | Toll Like Receptor 7 | Protein Coding |  | 52 | GC0XP012867 | | 2.42 | https://www.genecards.org/cgi-bin/carddisp.pl?gene=TLR7 |
| MAP2K3 | Mitogen-Activated Protein Kinase Kinase 3 | Protein Coding |  | 56 | GC17P025236 | | 2.42 | https://www.genecards.org/cgi-bin/carddisp.pl?gene=MAP2K3 |
| MIP | Major Intrinsic Protein Of Lens Fiber | Protein Coding |  | 48 | GC12M056449 | | 2.4 | https://www.genecards.org/cgi-bin/carddisp.pl?gene=MIP |
| P2RX7 | Purinergic Receptor P2X 7 | Protein Coding |  | 51 | GC12P122396 | | 2.4 | https://www.genecards.org/cgi-bin/carddisp.pl?gene=P2RX7 |
| DNTT | DNA Nucleotidylexotransferase | Protein Coding |  | 47 | GC10P096304 | | 2.38 | https://www.genecards.org/cgi-bin/carddisp.pl?gene=DNTT |
| MYLK | Myosin Light Chain Kinase | Protein Coding |  | 59 | GC03M123610 | | 2.37 | https://www.genecards.org/cgi-bin/carddisp.pl?gene=MYLK |
| CCL27 | C-C Motif Chemokine Ligand 27 | Protein Coding |  | 43 | GC09M034662 | | 2.37 | https://www.genecards.org/cgi-bin/carddisp.pl?gene=CCL27 |
| DEFB103B | Defensin Beta 103B | Protein Coding |  | 35 | GC08M007430 | | 2.36 | https://www.genecards.org/cgi-bin/carddisp.pl?gene=DEFB103B |
| TMPO | Thymopoietin | Protein Coding |  | 51 | GC12P098515 | | 2.35 | https://www.genecards.org/cgi-bin/carddisp.pl?gene=TMPO |
| PPIG | Peptidylprolyl Isomerase G | Protein Coding |  | 47 | GC02P169584 | | 2.35 | https://www.genecards.org/cgi-bin/carddisp.pl?gene=PPIG |
| C4BPA | Complement Component 4 Binding Protein Alpha | Protein Coding |  | 46 | GC01P207105 | | 2.34 | https://www.genecards.org/cgi-bin/carddisp.pl?gene=C4BPA |
| TNPO1 | Transportin 1 | Protein Coding |  | 45 | GC05P072816 | | 2.34 | https://www.genecards.org/cgi-bin/carddisp.pl?gene=TNPO1 |
| TINF2 | TERF1 Interacting Nuclear Factor 2 | Protein Coding |  | 46 | GC14M024234 | | 2.33 | https://www.genecards.org/cgi-bin/carddisp.pl?gene=TINF2 |
| PAFAH1B1 | Platelet Activating Factor Acetylhydrolase 1b Regulatory Subunit 1 | Protein Coding |  | 53 | GC17P002593 | | 2.33 | https://www.genecards.org/cgi-bin/carddisp.pl?gene=PAFAH1B1 |
| GCLC | Glutamate-Cysteine Ligase Catalytic Subunit | Protein Coding |  | 51 | GC06M053497 | | 2.33 | https://www.genecards.org/cgi-bin/carddisp.pl?gene=GCLC |
| MAP2K6 | Mitogen-Activated Protein Kinase Kinase 6 | Protein Coding |  | 53 | GC17P069414 | | 2.32 | https://www.genecards.org/cgi-bin/carddisp.pl?gene=MAP2K6 |
| CDH5 | Cadherin 5 | Protein Coding |  | 55 | GC16P066366 | | 2.32 | https://www.genecards.org/cgi-bin/carddisp.pl?gene=CDH5 |
| NTS | Neurotensin | Protein Coding |  | 45 | GC12P085876 | | 2.31 | https://www.genecards.org/cgi-bin/carddisp.pl?gene=NTS |
| CCL19 | C-C Motif Chemokine Ligand 19 | Protein Coding |  | 48 | GC09M034692 | | 2.31 | https://www.genecards.org/cgi-bin/carddisp.pl?gene=CCL19 |
| CCL20 | C-C Motif Chemokine Ligand 20 | Protein Coding |  | 50 | GC02P227813 | | 2.3 | https://www.genecards.org/cgi-bin/carddisp.pl?gene=CCL20 |
| ITGAL | Integrin Subunit Alpha L | Protein Coding |  | 52 | GC16P030483 | | 2.3 | https://www.genecards.org/cgi-bin/carddisp.pl?gene=ITGAL |
| SGCG | Sarcoglycan Gamma | Protein Coding |  | 49 | GC13P023160 | | 2.3 | https://www.genecards.org/cgi-bin/carddisp.pl?gene=SGCG |
| HBG2 | Hemoglobin Subunit Gamma 2 | Protein Coding |  | 49 | GC11M005280 | | 2.3 | https://www.genecards.org/cgi-bin/carddisp.pl?gene=HBG2 |
| ERG | ETS Transcription Factor ERG | Protein Coding |  | 52 | GC21M038367 | | 2.27 | https://www.genecards.org/cgi-bin/carddisp.pl?gene=ERG |
| DNAJC21 | DnaJ Heat Shock Protein Family (Hsp40) Member C21 | Protein Coding |  | 42 | GC05P034929 | | 2.27 | https://www.genecards.org/cgi-bin/carddisp.pl?gene=DNAJC21 |
| KEAP1 | Kelch Like ECH Associated Protein 1 | Protein Coding |  | 53 | GC19M010457 | | 2.24 | https://www.genecards.org/cgi-bin/carddisp.pl?gene=KEAP1 |
| PLCG1 | Phospholipase C Gamma 1 | Protein Coding |  | 53 | GC20P041136 | | 2.24 | https://www.genecards.org/cgi-bin/carddisp.pl?gene=PLCG1 |
| IL12A | Interleukin 12A | Protein Coding |  | 51 | GC03P159988 | | 2.24 | https://www.genecards.org/cgi-bin/carddisp.pl?gene=IL12A |
| PIM1 | Pim-1 Proto-Oncogene, Serine/Threonine Kinase | Protein Coding |  | 57 | GC06P043184 | | 2.23 | https://www.genecards.org/cgi-bin/carddisp.pl?gene=PIM1 |
| NR3C1 | Nuclear Receptor Subfamily 3 Group C Member 1 | Protein Coding |  | 57 | GC05M143241 | | 2.22 | https://www.genecards.org/cgi-bin/carddisp.pl?gene=NR3C1 |
| EPG5 | Ectopic P-Granules Autophagy Protein 5 Homolog | Protein Coding |  | 42 | GC18M045800 | | 2.21 | https://www.genecards.org/cgi-bin/carddisp.pl?gene=EPG5 |
| MAOB | Monoamine Oxidase B | Protein Coding |  | 49 | GC0XM043766 | | 2.21 | https://www.genecards.org/cgi-bin/carddisp.pl?gene=MAOB |
| HLA-G | Major Histocompatibility Complex, Class I, G | Protein Coding |  | 50 | GC06P032416 | | 2.21 | https://www.genecards.org/cgi-bin/carddisp.pl?gene=HLA-G |
| ENTPD1 | Ectonucleoside Triphosphate Diphosphohydrolase 1 | Protein Coding |  | 53 | GC10P095711 | | 2.21 | https://www.genecards.org/cgi-bin/carddisp.pl?gene=ENTPD1 |
| ITGB3 | Integrin Subunit Beta 3 | Protein Coding |  | 56 | GC17P047254 | | 2.2 | https://www.genecards.org/cgi-bin/carddisp.pl?gene=ITGB3 |
| CDH17 | Cadherin 17 | Protein Coding |  | 47 | GC08M094127 | | 2.19 | https://www.genecards.org/cgi-bin/carddisp.pl?gene=CDH17 |
| ABCB7 | ATP Binding Cassette Subfamily B Member 7 | Protein Coding |  | 48 | GC0XM075053 | | 2.19 | https://www.genecards.org/cgi-bin/carddisp.pl?gene=ABCB7 |
| PDCD4 | Programmed Cell Death 4 | Protein Coding |  | 49 | GC10P110871 | | 2.18 | https://www.genecards.org/cgi-bin/carddisp.pl?gene=PDCD4 |
| SIGLEC5 | Sialic Acid Binding Ig Like Lectin 5 | Protein Coding |  | 43 | GC19M051611 | | 2.18 | https://www.genecards.org/cgi-bin/carddisp.pl?gene=SIGLEC5 |
| AHR | Aryl Hydrocarbon Receptor | Protein Coding |  | 56 | GC07P016916 | | 2.17 | https://www.genecards.org/cgi-bin/carddisp.pl?gene=AHR |
| CSN1S1 | Casein Alpha S1 | Protein Coding |  | 39 | GC04P069932 | | 2.17 | https://www.genecards.org/cgi-bin/carddisp.pl?gene=CSN1S1 |
| CSRP1 | Cysteine And Glycine Rich Protein 1 | Protein Coding |  | 46 | GC01M201452 | | 2.17 | https://www.genecards.org/cgi-bin/carddisp.pl?gene=CSRP1 |
| FSCN1 | Fascin Actin-Bundling Protein 1 | Protein Coding |  | 50 | GC07P005632 | | 2.16 | https://www.genecards.org/cgi-bin/carddisp.pl?gene=FSCN1 |
| DHODH | Dihydroorotate Dehydrogenase (Quinone) | Protein Coding |  | 51 | GC16P072042 | | 2.16 | https://www.genecards.org/cgi-bin/carddisp.pl?gene=DHODH |
| IL21 | Interleukin 21 | Protein Coding |  | 50 | GC04M122612 | | 2.16 | https://www.genecards.org/cgi-bin/carddisp.pl?gene=IL21 |
| SYK | Spleen Associated Tyrosine Kinase | Protein Coding |  | 56 | GC09P091055 | | 2.15 | https://www.genecards.org/cgi-bin/carddisp.pl?gene=SYK |
| UCK2 | Uridine-Cytidine Kinase 2 | Protein Coding |  | 48 | GC01P165796 | | 2.15 | https://www.genecards.org/cgi-bin/carddisp.pl?gene=UCK2 |
| MUC7 | Mucin 7, Secreted | Protein Coding |  | 43 | GC04P070430 | | 2.15 | https://www.genecards.org/cgi-bin/carddisp.pl?gene=MUC7 |
| TTC7A | Tetratricopeptide Repeat Domain 7A | Protein Coding |  | 45 | GC02P046906 | | 2.15 | https://www.genecards.org/cgi-bin/carddisp.pl?gene=TTC7A |
| FABP2 | Fatty Acid Binding Protein 2 | Protein Coding |  | 49 | GC04M119317 | | 2.14 | https://www.genecards.org/cgi-bin/carddisp.pl?gene=FABP2 |
| NTF3 | Neurotrophin 3 | Protein Coding |  | 50 | GC12P005432 | | 2.14 | https://www.genecards.org/cgi-bin/carddisp.pl?gene=NTF3 |
| TRIM21 | Tripartite Motif Containing 21 | Protein Coding |  | 48 | GC11M004406 | | 2.14 | https://www.genecards.org/cgi-bin/carddisp.pl?gene=TRIM21 |
| OAS1 | 2'-5'-Oligoadenylate Synthetase 1 | Protein Coding |  | 52 | GC12P112906 | | 2.14 | https://www.genecards.org/cgi-bin/carddisp.pl?gene=OAS1 |
| BMP4 | Bone Morphogenetic Protein 4 | Protein Coding |  | 55 | GC14M053949 | | 2.14 | https://www.genecards.org/cgi-bin/carddisp.pl?gene=BMP4 |
| HDAC8 | Histone Deacetylase 8 | Protein Coding |  | 53 | GC0XM072329 | | 2.14 | https://www.genecards.org/cgi-bin/carddisp.pl?gene=HDAC8 |
| MAPK13 | Mitogen-Activated Protein Kinase 13 | Protein Coding |  | 54 | GC06P043145 | | 2.13 | https://www.genecards.org/cgi-bin/carddisp.pl?gene=MAPK13 |
| CCR8 | C-C Motif Chemokine Receptor 8 | Protein Coding |  | 50 | GC03P039330 | | 2.13 | https://www.genecards.org/cgi-bin/carddisp.pl?gene=CCR8 |
| IRF5 | Interferon Regulatory Factor 5 | Protein Coding |  | 54 | GC07P128937 | | 2.12 | https://www.genecards.org/cgi-bin/carddisp.pl?gene=IRF5 |
| NAMPT | Nicotinamide Phosphoribosyltransferase | Protein Coding |  | 54 | GC07M106248 | | 2.12 | https://www.genecards.org/cgi-bin/carddisp.pl?gene=NAMPT |
| PLA2G2A | Phospholipase A2 Group IIA | Protein Coding |  | 52 | GC01M019975 | | 2.12 | https://www.genecards.org/cgi-bin/carddisp.pl?gene=PLA2G2A |
| FCN2 | Ficolin 2 | Protein Coding |  | 47 | GC09P134864 | | 2.12 | https://www.genecards.org/cgi-bin/carddisp.pl?gene=FCN2 |
| NCF4 | Neutrophil Cytosolic Factor 4 | Protein Coding |  | 54 | GC22P036860 | | 2.11 | https://www.genecards.org/cgi-bin/carddisp.pl?gene=NCF4 |
| CASP6 | Caspase 6 | Protein Coding |  | 54 | GC04M109688 | | 2.11 | https://www.genecards.org/cgi-bin/carddisp.pl?gene=CASP6 |
| MUC16 | Mucin 16, Cell Surface Associated | Protein Coding |  | 42 | GC19M008904 | | 2.11 | https://www.genecards.org/cgi-bin/carddisp.pl?gene=MUC16 |
| RBX1 | Ring-Box 1 | Protein Coding |  | 48 | GC22P040951 | | 2.11 | https://www.genecards.org/cgi-bin/carddisp.pl?gene=RBX1 |
| ABCA1 | ATP Binding Cassette Subfamily A Member 1 | Protein Coding |  | 55 | GC09M104781 | | 2.1 | https://www.genecards.org/cgi-bin/carddisp.pl?gene=ABCA1 |
| KNG1 | Kininogen 1 | Protein Coding |  | 50 | GC03P186717 | | 2.09 | https://www.genecards.org/cgi-bin/carddisp.pl?gene=KNG1 |
| PHOX2B | Paired Like Homeobox 2B | Protein Coding |  | 50 | GC04M041746 | | 2.09 | https://www.genecards.org/cgi-bin/carddisp.pl?gene=PHOX2B |
| MAPKAPK2 | MAPK Activated Protein Kinase 2 | Protein Coding |  | 54 | GC01P206684 | | 2.09 | https://www.genecards.org/cgi-bin/carddisp.pl?gene=MAPKAPK2 |
| IL33 | Interleukin 33 | Protein Coding |  | 45 | GC09P006206 | | 2.09 | https://www.genecards.org/cgi-bin/carddisp.pl?gene=IL33 |
| S100A12 | S100 Calcium Binding Protein A12 | Protein Coding |  | 46 | GC01M153346 | | 2.09 | https://www.genecards.org/cgi-bin/carddisp.pl?gene=S100A12 |
| GPX2 | Glutathione Peroxidase 2 | Protein Coding |  | 51 | GC14M064939 | | 2.08 | https://www.genecards.org/cgi-bin/carddisp.pl?gene=GPX2 |
| COL3A1 | Collagen Type III Alpha 1 Chain | Protein Coding |  | 53 | GC02P188974 | | 2.07 | https://www.genecards.org/cgi-bin/carddisp.pl?gene=COL3A1 |
| FLAD1 | Flavin Adenine Dinucleotide Synthetase 1 | Protein Coding |  | 47 | GC01P154983 | | 2.05 | https://www.genecards.org/cgi-bin/carddisp.pl?gene=FLAD1 |
| LALBA | Lactalbumin Alpha | Protein Coding |  | 45 | GC12M048567 | | 2.05 | https://www.genecards.org/cgi-bin/carddisp.pl?gene=LALBA |
| MCAM | Melanoma Cell Adhesion Molecule | Protein Coding |  | 46 | GC11M119308 | | 2.05 | https://www.genecards.org/cgi-bin/carddisp.pl?gene=MCAM |
| ANXA6 | Annexin A6 | Protein Coding |  | 49 | GC05M151077 | | 2.04 | https://www.genecards.org/cgi-bin/carddisp.pl?gene=ANXA6 |
| C5 | Complement C5 | Protein Coding |  | 53 | GC09M120952 | | 2.04 | https://www.genecards.org/cgi-bin/carddisp.pl?gene=C5 |
| NUMB | NUMB Endocytic Adaptor Protein | Protein Coding |  | 53 | GC14M073275 | | 2.04 | https://www.genecards.org/cgi-bin/carddisp.pl?gene=NUMB |
| NDN | Necdin, MAGE Family Member | Protein Coding |  | 47 | GC15M023686 | | 2.03 | https://www.genecards.org/cgi-bin/carddisp.pl?gene=NDN |
| PAK4 | P21 (RAC1) Activated Kinase 4 | Protein Coding |  | 55 | GC19P039125 | | 2.03 | https://www.genecards.org/cgi-bin/carddisp.pl?gene=PAK4 |
| PLCG2 | Phospholipase C Gamma 2 | Protein Coding |  | 57 | GC16P081773 | | 2.03 | https://www.genecards.org/cgi-bin/carddisp.pl?gene=PLCG2 |
| TARDBP | TAR DNA Binding Protein | Protein Coding |  | 52 | GC01P011013 | | 2.03 | https://www.genecards.org/cgi-bin/carddisp.pl?gene=TARDBP |
| IFNAR2 | Interferon Alpha And Beta Receptor Subunit 2 | Protein Coding |  | 54 | GC21P033229 | | 2.02 | https://www.genecards.org/cgi-bin/carddisp.pl?gene=IFNAR2 |
| NCOR2 | Nuclear Receptor Corepressor 2 | Protein Coding |  | 49 | GC12M124324 | | 2 | https://www.genecards.org/cgi-bin/carddisp.pl?gene=NCOR2 |
| NEU3 | Neuraminidase 3 | Protein Coding |  | 45 | GC11P074988 | | 2 | https://www.genecards.org/cgi-bin/carddisp.pl?gene=NEU3 |
| NLRP12 | NLR Family Pyrin Domain Containing 12 | Protein Coding |  | 50 | GC19M053793 | | 2 | https://www.genecards.org/cgi-bin/carddisp.pl?gene=NLRP12 |
| AGER | Advanced Glycosylation End-Product Specific Receptor | Protein Coding |  | 50 | GC06M032180 | | 2 | https://www.genecards.org/cgi-bin/carddisp.pl?gene=AGER |
| ALDOA | Aldolase, Fructose-Bisphosphate A | Protein Coding |  | 54 | GC16P030064 | | 2 | https://www.genecards.org/cgi-bin/carddisp.pl?gene=ALDOA |
| CCL21 | C-C Motif Chemokine Ligand 21 | Protein Coding |  | 48 | GC09M034709 | | 1.99 | https://www.genecards.org/cgi-bin/carddisp.pl?gene=CCL21 |
| LMAN1 | Lectin, Mannose Binding 1 | Protein Coding |  | 51 | GC18M059327 | | 1.99 | https://www.genecards.org/cgi-bin/carddisp.pl?gene=LMAN1 |
| TGM1 | Transglutaminase 1 | Protein Coding |  | 51 | GC14M024249 | | 1.98 | https://www.genecards.org/cgi-bin/carddisp.pl?gene=TGM1 |
| HBA1 | Hemoglobin Subunit Alpha 1 | Protein Coding |  | 48 | GC16P000574 | | 1.97 | https://www.genecards.org/cgi-bin/carddisp.pl?gene=HBA1 |
| ITGA3 | Integrin Subunit Alpha 3 | Protein Coding |  | 53 | GC17P050055 | | 1.96 | https://www.genecards.org/cgi-bin/carddisp.pl?gene=ITGA3 |
| ADK | Adenosine Kinase | Protein Coding |  | 57 | GC10P074152 | | 1.96 | https://www.genecards.org/cgi-bin/carddisp.pl?gene=ADK |
| EIF2AK2 | Eukaryotic Translation Initiation Factor 2 Alpha Kinase 2 | Protein Coding |  | 51 | GC02M037073 | | 1.96 | https://www.genecards.org/cgi-bin/carddisp.pl?gene=EIF2AK2 |
| DKC1 | Dyskerin Pseudouridine Synthase 1 | Protein Coding |  | 53 | GC0XP154762 | | 1.96 | https://www.genecards.org/cgi-bin/carddisp.pl?gene=DKC1 |
| HELLS | Helicase, Lymphoid Specific | Protein Coding |  | 51 | GC10P094501 | | 1.95 | https://www.genecards.org/cgi-bin/carddisp.pl?gene=HELLS |
| PDPN | Podoplanin | Protein Coding |  | 46 | GC01P013656 | | 1.95 | https://www.genecards.org/cgi-bin/carddisp.pl?gene=PDPN |
| PSMB8 | Proteasome Subunit Beta 8 | Protein Coding |  | 57 | GC06M032840 | | 1.95 | https://www.genecards.org/cgi-bin/carddisp.pl?gene=PSMB8 |
| CTSH | Cathepsin H | Protein Coding |  | 53 | GC15M078925 | | 1.95 | https://www.genecards.org/cgi-bin/carddisp.pl?gene=CTSH |
| YWHAZ | Tyrosine 3-Monooxygenase/Tryptophan 5-Monooxygenase Activation Protein Zeta | Protein Coding |  | 53 | GC08M100917 | | 1.94 | https://www.genecards.org/cgi-bin/carddisp.pl?gene=YWHAZ |
| DCTN4 | Dynactin Subunit 4 | Protein Coding |  | 45 | GC05M150708 | | 1.94 | https://www.genecards.org/cgi-bin/carddisp.pl?gene=DCTN4 |
| CFAP298 | Cilia And Flagella Associated Protein 298 | Protein Coding |  | 34 | GC21M032593 | | 1.94 | https://www.genecards.org/cgi-bin/carddisp.pl?gene=CFAP298 |
| CHM | CHM Rab Escort Protein | Protein Coding |  | 46 | GC0XM085861 | | 1.94 | https://www.genecards.org/cgi-bin/carddisp.pl?gene=CHM |
| PRPF8 | Pre-MRNA Processing Factor 8 | Protein Coding |  | 48 | GC17M001650 | | 1.94 | https://www.genecards.org/cgi-bin/carddisp.pl?gene=PRPF8 |
| IL10RA | Interleukin 10 Receptor Subunit Alpha | Protein Coding |  | 51 | GC11P117987 | | 1.93 | https://www.genecards.org/cgi-bin/carddisp.pl?gene=IL10RA |
| PON1 | Paraoxonase 1 | Protein Coding |  | 53 | GC07M095297 | | 1.93 | https://www.genecards.org/cgi-bin/carddisp.pl?gene=PON1 |
| PTGS1 | Prostaglandin-Endoperoxide Synthase 1 | Protein Coding |  | 53 | GC09P122370 | | 1.93 | https://www.genecards.org/cgi-bin/carddisp.pl?gene=PTGS1 |
| ACKR3 | Atypical Chemokine Receptor 3 | Protein Coding |  | 46 | GC02P236537 | | 1.91 | https://www.genecards.org/cgi-bin/carddisp.pl?gene=ACKR3 |
| POU5F1 | POU Class 5 Homeobox 1 | Protein Coding |  | 53 | GC06M031136 | | 1.9 | https://www.genecards.org/cgi-bin/carddisp.pl?gene=POU5F1 |
| CEP290 | Centrosomal Protein 290 | Protein Coding |  | 46 | GC12M088049 | | 1.89 | https://www.genecards.org/cgi-bin/carddisp.pl?gene=CEP290 |
| USH2A | Usherin | Protein Coding |  | 44 | GC01M215622 | | 1.89 | https://www.genecards.org/cgi-bin/carddisp.pl?gene=USH2A |
| CPS1 | Carbamoyl-Phosphate Synthase 1 | Protein Coding |  | 51 | GC02P210477 | | 1.88 | https://www.genecards.org/cgi-bin/carddisp.pl?gene=CPS1 |
| ITK | IL2 Inducible T Cell Kinase | Protein Coding |  | 59 | GC05P157158 | | 1.88 | https://www.genecards.org/cgi-bin/carddisp.pl?gene=ITK |
| CXCL11 | C-X-C Motif Chemokine Ligand 11 | Protein Coding |  | 46 | GC04M076033 | | 1.88 | https://www.genecards.org/cgi-bin/carddisp.pl?gene=CXCL11 |
| FURIN | Furin, Paired Basic Amino Acid Cleaving Enzyme | Protein Coding |  | 54 | GC15P090868 | | 1.88 | https://www.genecards.org/cgi-bin/carddisp.pl?gene=FURIN |
| LYZ | Lysozyme | Protein Coding |  | 53 | GC12P069348 | | 1.87 | https://www.genecards.org/cgi-bin/carddisp.pl?gene=LYZ |
| PMVK | Phosphomevalonate Kinase | Protein Coding |  | 51 | GC01M154924 | | 1.87 | https://www.genecards.org/cgi-bin/carddisp.pl?gene=PMVK |
| PLP1 | Proteolipid Protein 1 | Protein Coding |  | 49 | GC0XP103773 | | 1.87 | https://www.genecards.org/cgi-bin/carddisp.pl?gene=PLP1 |
| CEACAM7 | Carcinoembryonic Antigen Related Cell Adhesion Molecule 7 | Protein Coding |  | 43 | GC19M041673 | | 1.87 | https://www.genecards.org/cgi-bin/carddisp.pl?gene=CEACAM7 |
| TRB | T Cell Receptor Beta Locus | Protein Coding |  | 19 | GC07P144424 | | 1.86 | https://www.genecards.org/cgi-bin/carddisp.pl?gene=TRB |
| SMC5 | Structural Maintenance Of Chromosomes 5 | Protein Coding |  | 43 | GC09P070258 | | 1.86 | https://www.genecards.org/cgi-bin/carddisp.pl?gene=SMC5 |
| MSN | Moesin | Protein Coding |  | 53 | GC0XP065588 | | 1.86 | https://www.genecards.org/cgi-bin/carddisp.pl?gene=MSN |
| SERPINF1 | Serpin Family F Member 1 | Protein Coding |  | 51 | GC17P001761 | | 1.85 | https://www.genecards.org/cgi-bin/carddisp.pl?gene=SERPINF1 |
| CSNK2A1 | Casein Kinase 2 Alpha 1 | Protein Coding |  | 57 | GC20M000459 | | 1.85 | https://www.genecards.org/cgi-bin/carddisp.pl?gene=CSNK2A1 |
| CTSD | Cathepsin D | Protein Coding |  | 59 | GC11M001752 | | 1.85 | https://www.genecards.org/cgi-bin/carddisp.pl?gene=CTSD |
| GMNN | Geminin DNA Replication Inhibitor | Protein Coding |  | 51 | GC06P024779 | | 1.85 | https://www.genecards.org/cgi-bin/carddisp.pl?gene=GMNN |
| ARID1B | AT-Rich Interaction Domain 1B | Protein Coding |  | 51 | GC06P156777 | | 1.84 | https://www.genecards.org/cgi-bin/carddisp.pl?gene=ARID1B |
| S100A7 | S100 Calcium Binding Protein A7 | Protein Coding |  | 46 | GC01M153430 | | 1.84 | https://www.genecards.org/cgi-bin/carddisp.pl?gene=S100A7 |
| PLA2G4A | Phospholipase A2 Group IVA | Protein Coding |  | 54 | GC01P186798 | | 1.83 | https://www.genecards.org/cgi-bin/carddisp.pl?gene=PLA2G4A |
| PHKA2 | Phosphorylase Kinase Regulatory Subunit Alpha 2 | Protein Coding |  | 52 | GC0XM018892 | | 1.83 | https://www.genecards.org/cgi-bin/carddisp.pl?gene=PHKA2 |
| PIK3CB | Phosphatidylinositol-4,5-Bisphosphate 3-Kinase Catalytic Subunit Beta | Protein Coding |  | 54 | GC03M138652 | | 1.83 | https://www.genecards.org/cgi-bin/carddisp.pl?gene=PIK3CB |
| FCGR1A | Fc Fragment Of IgG Receptor Ia | Protein Coding |  | 48 | GC01P149754 | | 1.83 | https://www.genecards.org/cgi-bin/carddisp.pl?gene=FCGR1A |
| UQCRC1 | Ubiquinol-Cytochrome C Reductase Core Protein 1 | Protein Coding |  | 47 | GC03M048598 | | 1.82 | https://www.genecards.org/cgi-bin/carddisp.pl?gene=UQCRC1 |
| HDAC2 | Histone Deacetylase 2 | Protein Coding |  | 58 | GC06M113933 | | 1.81 | https://www.genecards.org/cgi-bin/carddisp.pl?gene=HDAC2 |
| PTPN3 | Protein Tyrosine Phosphatase Non-Receptor Type 3 | Protein Coding |  | 50 | GC09M109375 | | 1.81 | https://www.genecards.org/cgi-bin/carddisp.pl?gene=PTPN3 |
| SIVA1 | SIVA1 Apoptosis Inducing Factor | Protein Coding |  | 43 | GC14P104753 | | 1.81 | https://www.genecards.org/cgi-bin/carddisp.pl?gene=SIVA1 |
| DHX30 | DExH-Box Helicase 30 | Protein Coding |  | 45 | GC03P047802 | | 1.81 | https://www.genecards.org/cgi-bin/carddisp.pl?gene=DHX30 |
| ATP7A | ATPase Copper Transporting Alpha | Protein Coding |  | 53 | GC0XP077918 | | 1.8 | https://www.genecards.org/cgi-bin/carddisp.pl?gene=ATP7A |
| RPL22 | Ribosomal Protein L22 | Protein Coding |  | 49 | GC01M006179 | | 1.8 | https://www.genecards.org/cgi-bin/carddisp.pl?gene=RPL22 |
| STX1A | Syntaxin 1A | Protein Coding |  | 53 | GC07M073700 | | 1.8 | https://www.genecards.org/cgi-bin/carddisp.pl?gene=STX1A |
| HBM | Hemoglobin Subunit Mu | Protein Coding |  | 37 | GC16P000573 | | 1.8 | https://www.genecards.org/cgi-bin/carddisp.pl?gene=HBM |
| HSPA9 | Heat Shock Protein Family A (Hsp70) Member 9 | Protein Coding |  | 53 | GC05M138554 | | 1.8 | https://www.genecards.org/cgi-bin/carddisp.pl?gene=HSPA9 |
| GZMA | Granzyme A | Protein Coding |  | 48 | GC05P055102 | | 1.79 | https://www.genecards.org/cgi-bin/carddisp.pl?gene=GZMA |
| MED1 | Mediator Complex Subunit 1 | Protein Coding |  | 46 | GC17M039404 | | 1.79 | https://www.genecards.org/cgi-bin/carddisp.pl?gene=MED1 |
| VAPA | VAMP Associated Protein A | Protein Coding |  | 50 | GC18P009904 | | 1.78 | https://www.genecards.org/cgi-bin/carddisp.pl?gene=VAPA |
| APRT | Adenine Phosphoribosyltransferase | Protein Coding |  | 53 | GC16M088810 | | 1.78 | https://www.genecards.org/cgi-bin/carddisp.pl?gene=APRT |
| NOX1 | NADPH Oxidase 1 | Protein Coding |  | 47 | GC0XM100843 | | 1.77 | https://www.genecards.org/cgi-bin/carddisp.pl?gene=NOX1 |
| DMBT1 | Deleted In Malignant Brain Tumors 1 | Protein Coding |  | 47 | GC10P122560 | | 1.77 | https://www.genecards.org/cgi-bin/carddisp.pl?gene=DMBT1 |
| LY75 | Lymphocyte Antigen 75 | Protein Coding |  | 44 | GC02M159803 | | 1.77 | https://www.genecards.org/cgi-bin/carddisp.pl?gene=LY75 |
| BBC3 | BCL2 Binding Component 3 | Protein Coding |  | 46 | GC19M047220 | | 1.76 | https://www.genecards.org/cgi-bin/carddisp.pl?gene=BBC3 |
| GATA1 | GATA Binding Protein 1 | Protein Coding |  | 52 | GC0XP048786 | | 1.75 | https://www.genecards.org/cgi-bin/carddisp.pl?gene=GATA1 |
| CCL16 | C-C Motif Chemokine Ligand 16 | Protein Coding |  | 44 | GC17M035976 | | 1.74 | https://www.genecards.org/cgi-bin/carddisp.pl?gene=CCL16 |
| SMARCD2 | SWI/SNF Related, Matrix Associated, Actin Dependent Regulator Of Chromatin, Subfamily D, Member 2 | Protein Coding |  | 50 | GC17M063832 | | 1.74 | https://www.genecards.org/cgi-bin/carddisp.pl?gene=SMARCD2 |
| SRP54 | Signal Recognition Particle 54 | Protein Coding |  | 49 | GC14P034981 | | 1.74 | https://www.genecards.org/cgi-bin/carddisp.pl?gene=SRP54 |
| HAX1 | HCLS1 Associated Protein X-1 | Protein Coding |  | 48 | GC01P154273 | | 1.74 | https://www.genecards.org/cgi-bin/carddisp.pl?gene=HAX1 |
| CCNO | Cyclin O | Protein Coding |  | 48 | GC05M055231 | | 1.74 | https://www.genecards.org/cgi-bin/carddisp.pl?gene=CCNO |
| LAMTOR2 | Late Endosomal/Lysosomal Adaptor, MAPK And MTOR Activator 2 | Protein Coding |  | 46 | GC01P156054 | | 1.74 | https://www.genecards.org/cgi-bin/carddisp.pl?gene=LAMTOR2 |
| DNAH11 | Dynein Axonemal Heavy Chain 11 | Protein Coding |  | 46 | GC07P021582 | | 1.74 | https://www.genecards.org/cgi-bin/carddisp.pl?gene=DNAH11 |
| SPAG1 | Sperm Associated Antigen 1 | Protein Coding |  | 45 | GC08P100157 | | 1.74 | https://www.genecards.org/cgi-bin/carddisp.pl?gene=SPAG1 |
| DNAH1 | Dynein Axonemal Heavy Chain 1 | Protein Coding |  | 42 | GC03P052350 | | 1.74 | https://www.genecards.org/cgi-bin/carddisp.pl?gene=DNAH1 |
| ACP1 | Acid Phosphatase 1 | Protein Coding |  | 50 | GC02P000254 | | 1.74 | https://www.genecards.org/cgi-bin/carddisp.pl?gene=ACP1 |
| BCL6 | BCL6 Transcription Repressor | Protein Coding |  | 51 | GC03M187721 | | 1.73 | https://www.genecards.org/cgi-bin/carddisp.pl?gene=BCL6 |
| HAVCR2 | Hepatitis A Virus Cellular Receptor 2 | Protein Coding |  | 48 | GC05M157063 | | 1.73 | https://www.genecards.org/cgi-bin/carddisp.pl?gene=HAVCR2 |
| NME4 | NME/NM23 Nucleoside Diphosphate Kinase 4 | Protein Coding |  | 48 | GC16P000396 | | 1.73 | https://www.genecards.org/cgi-bin/carddisp.pl?gene=NME4 |
| FANCC | FA Complementation Group C | Protein Coding |  | 52 | GC09M095099 | | 1.72 | https://www.genecards.org/cgi-bin/carddisp.pl?gene=FANCC |
| EBI3 | Epstein-Barr Virus Induced 3 | Protein Coding |  | 45 | GC19P004231 | | 1.72 | https://www.genecards.org/cgi-bin/carddisp.pl?gene=EBI3 |
| ANXA1 | Annexin A1 | Protein Coding |  | 55 | GC09P073151 | | 1.71 | https://www.genecards.org/cgi-bin/carddisp.pl?gene=ANXA1 |
| NAGA | Alpha-N-Acetylgalactosaminidase | Protein Coding |  | 51 | GC22M042058 | | 1.71 | https://www.genecards.org/cgi-bin/carddisp.pl?gene=NAGA |
| NR1H2 | Nuclear Receptor Subfamily 1 Group H Member 2 | Protein Coding |  | 54 | GC19P050329 | | 1.7 | https://www.genecards.org/cgi-bin/carddisp.pl?gene=NR1H2 |
| ADM | Adrenomedullin | Protein Coding |  | 50 | GC11P010304 | | 1.69 | https://www.genecards.org/cgi-bin/carddisp.pl?gene=ADM |
| VAPB | VAMP Associated Protein B And C | Protein Coding |  | 51 | GC20P058389 | | 1.69 | https://www.genecards.org/cgi-bin/carddisp.pl?gene=VAPB |
| NOS1 | Nitric Oxide Synthase 1 | Protein Coding |  | 56 | GC12M117169 | | 1.69 | https://www.genecards.org/cgi-bin/carddisp.pl?gene=NOS1 |
| NR1H3 | Nuclear Receptor Subfamily 1 Group H Member 3 | Protein Coding |  | 53 | GC11P047269 | | 1.68 | https://www.genecards.org/cgi-bin/carddisp.pl?gene=NR1H3 |
| VCP | Valosin Containing Protein | Protein Coding |  | 55 | GC09M035056 | | 1.67 | https://www.genecards.org/cgi-bin/carddisp.pl?gene=VCP |
| SELPLG | Selectin P Ligand | Protein Coding |  | 48 | GC12M108621 | | 1.67 | https://www.genecards.org/cgi-bin/carddisp.pl?gene=SELPLG |
| CD33 | CD33 Molecule | Protein Coding |  | 50 | GC19P051225 | | 1.67 | https://www.genecards.org/cgi-bin/carddisp.pl?gene=CD33 |
| FCGR3A | Fc Fragment Of IgG Receptor IIIa | Protein Coding |  | 51 | GC01M161541 | | 1.67 | https://www.genecards.org/cgi-bin/carddisp.pl?gene=FCGR3A |
| ASAH1 | N-Acylsphingosine Amidohydrolase 1 | Protein Coding |  | 53 | GC08M017958 | | 1.66 | https://www.genecards.org/cgi-bin/carddisp.pl?gene=ASAH1 |
| MUC2 | Mucin 2, Oligomeric Mucus/Gel-Forming | Protein Coding |  | 45 | GC11P001074 | | 1.64 | https://www.genecards.org/cgi-bin/carddisp.pl?gene=MUC2 |
| USP9X | Ubiquitin Specific Peptidase 9 X-Linked | Protein Coding |  | 54 | GC0XP041085 | | 1.64 | https://www.genecards.org/cgi-bin/carddisp.pl?gene=USP9X |
| RUNX1T1 | RUNX1 Partner Transcriptional Co-Repressor 1 | Protein Coding |  | 46 | GC08M091954 | | 1.63 | https://www.genecards.org/cgi-bin/carddisp.pl?gene=RUNX1T1 |
| IGFBP2 | Insulin Like Growth Factor Binding Protein 2 | Protein Coding |  | 50 | GC02P216632 | | 1.63 | https://www.genecards.org/cgi-bin/carddisp.pl?gene=IGFBP2 |
| ETS1 | ETS Proto-Oncogene 1, Transcription Factor | Protein Coding |  | 55 | GC11M128458 | | 1.62 | https://www.genecards.org/cgi-bin/carddisp.pl?gene=ETS1 |
| ADPRH | ADP-Ribosylarginine Hydrolase | Protein Coding |  | 43 | GC03P119579 | | 1.62 | https://www.genecards.org/cgi-bin/carddisp.pl?gene=ADPRH |
| MSRA | Methionine Sulfoxide Reductase A | Protein Coding |  | 48 | GC08P010054 | | 1.61 | https://www.genecards.org/cgi-bin/carddisp.pl?gene=MSRA |
| RUNX2 | RUNX Family Transcription Factor 2 | Protein Coding |  | 53 | GC06P045327 | | 1.6 | https://www.genecards.org/cgi-bin/carddisp.pl?gene=RUNX2 |
| RANBP2 | RAN Binding Protein 2 | Protein Coding |  | 52 | GC02P108719 | | 1.6 | https://www.genecards.org/cgi-bin/carddisp.pl?gene=RANBP2 |
| CD163 | CD163 Molecule | Protein Coding |  | 49 | GC12M007518 | | 1.6 | https://www.genecards.org/cgi-bin/carddisp.pl?gene=CD163 |
| TNFSF14 | TNF Superfamily Member 14 | Protein Coding |  | 46 | GC19M006663 | | 1.6 | https://www.genecards.org/cgi-bin/carddisp.pl?gene=TNFSF14 |
| PTX3 | Pentraxin 3 | Protein Coding |  | 48 | GC03P157436 | | 1.6 | https://www.genecards.org/cgi-bin/carddisp.pl?gene=PTX3 |
| MAPKAP1 | MAPK Associated Protein 1 | Protein Coding |  | 51 | GC09M125437 | | 1.59 | https://www.genecards.org/cgi-bin/carddisp.pl?gene=MAPKAP1 |
| TNNI3 | Troponin I3, Cardiac Type | Protein Coding |  | 55 | GC19M055151 | | 1.59 | https://www.genecards.org/cgi-bin/carddisp.pl?gene=TNNI3 |
| BLM | BLM RecQ Like Helicase | Protein Coding |  | 54 | GC15P090717 | | 1.59 | https://www.genecards.org/cgi-bin/carddisp.pl?gene=BLM |
| SULF1 | Sulfatase 1 | Protein Coding |  | 46 | GC08P069466 | | 1.59 | https://www.genecards.org/cgi-bin/carddisp.pl?gene=SULF1 |
| WDR26 | WD Repeat Domain 26 | Protein Coding |  | 47 | GC01M224385 | | 1.59 | https://www.genecards.org/cgi-bin/carddisp.pl?gene=WDR26 |
| WNT1 | Wnt Family Member 1 | Protein Coding |  | 53 | GC12P049026 | | 1.59 | https://www.genecards.org/cgi-bin/carddisp.pl?gene=WNT1 |
| NAT1 | N-Acetyltransferase 1 | Protein Coding |  | 51 | GC08P018183 | | 1.59 | https://www.genecards.org/cgi-bin/carddisp.pl?gene=NAT1 |
| PSEN1 | Presenilin 1 | Protein Coding |  | 58 | GC14P073136 | | 1.58 | https://www.genecards.org/cgi-bin/carddisp.pl?gene=PSEN1 |
| ADAM17 | ADAM Metallopeptidase Domain 17 | Protein Coding |  | 57 | GC02M009580 | | 1.57 | https://www.genecards.org/cgi-bin/carddisp.pl?gene=ADAM17 |
| EPRS | Glutamyl-Prolyl-TRNA Synthetase | Protein Coding |  | 51 | GC01M219968 | | 1.57 | https://www.genecards.org/cgi-bin/carddisp.pl?gene=EPRS |
| PPP3CA | Protein Phosphatase 3 Catalytic Subunit Alpha | Protein Coding |  | 59 | GC04M101024 | | 1.57 | https://www.genecards.org/cgi-bin/carddisp.pl?gene=PPP3CA |
| SAMD9L | Sterile Alpha Motif Domain Containing 9 Like | Protein Coding |  | 43 | GC07M093130 | | 1.57 | https://www.genecards.org/cgi-bin/carddisp.pl?gene=SAMD9L |
| DGKE | Diacylglycerol Kinase Epsilon | Protein Coding |  | 54 | GC17P056834 | | 1.56 | https://www.genecards.org/cgi-bin/carddisp.pl?gene=DGKE |
| CDC6 | Cell Division Cycle 6 | Protein Coding |  | 51 | GC17P040287 | | 1.56 | https://www.genecards.org/cgi-bin/carddisp.pl?gene=CDC6 |
| MARCO | Macrophage Receptor With Collagenous Structure | Protein Coding |  | 46 | GC02P118942 | | 1.56 | https://www.genecards.org/cgi-bin/carddisp.pl?gene=MARCO |
| SOX11 | SRY-Box 11 | Protein Coding |  | 46 | GC02P005707 | | 1.55 | https://www.genecards.org/cgi-bin/carddisp.pl?gene=SOX11 |
| HAGLROS | HAGLR Opposite Strand LncRNA | RNA Gene |  | 15 | GC02P176177 | | 1.55 | https://www.genecards.org/cgi-bin/carddisp.pl?gene=HAGLROS |
| DSG1 | Desmoglein 1 | Protein Coding |  | 50 | GC18P031318 | | 1.55 | https://www.genecards.org/cgi-bin/carddisp.pl?gene=DSG1 |
| DLD | Dihydrolipoamide Dehydrogenase | Protein Coding |  | 56 | GC07P107890 | | 1.55 | https://www.genecards.org/cgi-bin/carddisp.pl?gene=DLD |
| SLC9A3 | Solute Carrier Family 9 Member A3 | Protein Coding |  | 53 | GC05M000472 | | 1.55 | https://www.genecards.org/cgi-bin/carddisp.pl?gene=SLC9A3 |
| CUL1 | Cullin 1 | Protein Coding |  | 51 | GC07P148697 | | 1.55 | https://www.genecards.org/cgi-bin/carddisp.pl?gene=CUL1 |
| PDE4B | Phosphodiesterase 4B | Protein Coding |  | 51 | GC01P065792 | | 1.55 | https://www.genecards.org/cgi-bin/carddisp.pl?gene=PDE4B |
| KCNQ1 | Potassium Voltage-Gated Channel Subfamily Q Member 1 | Protein Coding |  | 56 | GC11P002444 | | 1.54 | https://www.genecards.org/cgi-bin/carddisp.pl?gene=KCNQ1 |
| DDX39B | DExD-Box Helicase 39B | Protein Coding |  | 45 | GC06M031530 | | 1.53 | https://www.genecards.org/cgi-bin/carddisp.pl?gene=DDX39B |
| FGG | Fibrinogen Gamma Chain | Protein Coding |  | 54 | GC04M154604 | | 1.53 | https://www.genecards.org/cgi-bin/carddisp.pl?gene=FGG |
| KPNB1 | Karyopherin Subunit Beta 1 | Protein Coding |  | 49 | GC17P047649 | | 1.52 | https://www.genecards.org/cgi-bin/carddisp.pl?gene=KPNB1 |
| RHOD | Ras Homolog Family Member D | Protein Coding |  | 46 | GC11P067057 | | 1.52 | https://www.genecards.org/cgi-bin/carddisp.pl?gene=RHOD |
| FGB | Fibrinogen Beta Chain | Protein Coding |  | 53 | GC04P154564 | | 1.52 | https://www.genecards.org/cgi-bin/carddisp.pl?gene=FGB |
| CBX5 | Chromobox 5 | Protein Coding |  | 50 | GC12M054230 | | 1.51 | https://www.genecards.org/cgi-bin/carddisp.pl?gene=CBX5 |
| ACVRL1 | Activin A Receptor Like Type 1 | Protein Coding |  | 56 | GC12P051906 | | 1.51 | https://www.genecards.org/cgi-bin/carddisp.pl?gene=ACVRL1 |
| GLUD1 | Glutamate Dehydrogenase 1 | Protein Coding |  | 57 | GC10M087050 | | 1.51 | https://www.genecards.org/cgi-bin/carddisp.pl?gene=GLUD1 |
| CCL24 | C-C Motif Chemokine Ligand 24 | Protein Coding |  | 42 | GC07M075811 | | 1.51 | https://www.genecards.org/cgi-bin/carddisp.pl?gene=CCL24 |
| COL4A2 | Collagen Type IV Alpha 2 Chain | Protein Coding |  | 51 | GC13P110305 | | 1.51 | https://www.genecards.org/cgi-bin/carddisp.pl?gene=COL4A2 |
| HSPE1 | Heat Shock Protein Family E (Hsp10) Member 1 | Protein Coding |  | 46 | GC02P197501 | | 1.51 | https://www.genecards.org/cgi-bin/carddisp.pl?gene=HSPE1 |
| OCRL | OCRL Inositol Polyphosphate-5-Phosphatase | Protein Coding |  | 50 | GC0XP129539 | | 1.5 | https://www.genecards.org/cgi-bin/carddisp.pl?gene=OCRL |
| SLC37A4 | Solute Carrier Family 37 Member 4 | Protein Coding |  | 48 | GC11M119024 | | 1.5 | https://www.genecards.org/cgi-bin/carddisp.pl?gene=SLC37A4 |
| PSTPIP1 | Proline-Serine-Threonine Phosphatase Interacting Protein 1 | Protein Coding |  | 51 | GC15P076993 | | 1.5 | https://www.genecards.org/cgi-bin/carddisp.pl?gene=PSTPIP1 |
| PRDX2 | Peroxiredoxin 2 | Protein Coding |  | 53 | GC19M012796 | | 1.5 | https://www.genecards.org/cgi-bin/carddisp.pl?gene=PRDX2 |
| TMBIM4 | Transmembrane BAX Inhibitor Motif Containing 4 | Protein Coding |  | 40 | GC12M066135 | | 1.5 | https://www.genecards.org/cgi-bin/carddisp.pl?gene=TMBIM4 |
| DROSHA | Drosha Ribonuclease III | Protein Coding |  | 47 | GC05M031401 | | 1.49 | https://www.genecards.org/cgi-bin/carddisp.pl?gene=DROSHA |
| DDX58 | DExD/H-Box Helicase 58 | Protein Coding |  | 54 | GC09M032455 | | 1.48 | https://www.genecards.org/cgi-bin/carddisp.pl?gene=DDX58 |
| ASH2L | ASH2 Like, Histone Lysine Methyltransferase Complex Subunit | Protein Coding |  | 48 | GC08P038104 | | 1.46 | https://www.genecards.org/cgi-bin/carddisp.pl?gene=ASH2L |
| CPA6 | Carboxypeptidase A6 | Protein Coding |  | 48 | GC08M067463 | | 1.46 | https://www.genecards.org/cgi-bin/carddisp.pl?gene=CPA6 |
| CLDN4 | Claudin 4 | Protein Coding |  | 47 | GC07P073799 | | 1.46 | https://www.genecards.org/cgi-bin/carddisp.pl?gene=CLDN4 |
| MC4R | Melanocortin 4 Receptor | Protein Coding |  | 50 | GC18M060371 | | 1.46 | https://www.genecards.org/cgi-bin/carddisp.pl?gene=MC4R |
| PHEX | Phosphate Regulating Endopeptidase Homolog X-Linked | Protein Coding |  | 47 | GC0XP022052 | | 1.46 | https://www.genecards.org/cgi-bin/carddisp.pl?gene=PHEX |
| NFKBIE | NFKB Inhibitor Epsilon | Protein Coding |  | 45 | GC06M044226 | | 1.45 | https://www.genecards.org/cgi-bin/carddisp.pl?gene=NFKBIE |
| FLOT1 | Flotillin 1 | Protein Coding |  | 46 | GC06M030729 | | 1.45 | https://www.genecards.org/cgi-bin/carddisp.pl?gene=FLOT1 |
| CSN3 | Casein Kappa | Protein Coding |  | 42 | GC04P070242 | | 1.45 | https://www.genecards.org/cgi-bin/carddisp.pl?gene=CSN3 |
| GJB2 | Gap Junction Protein Beta 2 | Protein Coding |  | 53 | GC13M020187 | | 1.45 | https://www.genecards.org/cgi-bin/carddisp.pl?gene=GJB2 |
| TPPP | Tubulin Polymerization Promoting Protein | Protein Coding |  | 46 | GC05M000659 | | 1.45 | https://www.genecards.org/cgi-bin/carddisp.pl?gene=TPPP |
| MYO1D | Myosin ID | Protein Coding |  | 45 | GC17M032492 | | 1.45 | https://www.genecards.org/cgi-bin/carddisp.pl?gene=MYO1D |
| SPEN | Spen Family Transcriptional Repressor | Protein Coding |  | 45 | GC01P015848 | | 1.45 | https://www.genecards.org/cgi-bin/carddisp.pl?gene=SPEN |
| LAMA2 | Laminin Subunit Alpha 2 | Protein Coding |  | 48 | GC06P128863 | | 1.45 | https://www.genecards.org/cgi-bin/carddisp.pl?gene=LAMA2 |
| RPGR | Retinitis Pigmentosa GTPase Regulator | Protein Coding |  | 47 | GC0XM038269 | | 1.45 | https://www.genecards.org/cgi-bin/carddisp.pl?gene=RPGR |
| HPSE | Heparanase | Protein Coding |  | 51 | GC04M083292 | | 1.44 | https://www.genecards.org/cgi-bin/carddisp.pl?gene=HPSE |
| ZEB2 | Zinc Finger E-Box Binding Homeobox 2 | Protein Coding |  | 54 | GC02M144384 | | 1.43 | https://www.genecards.org/cgi-bin/carddisp.pl?gene=ZEB2 |
| CDH23 | Cadherin Related 23 | Protein Coding |  | 48 | GC10P071396 | | 1.43 | https://www.genecards.org/cgi-bin/carddisp.pl?gene=CDH23 |
| CLEC4A | C-Type Lectin Domain Family 4 Member A | Protein Coding |  | 44 | GC12P008123 | | 1.43 | https://www.genecards.org/cgi-bin/carddisp.pl?gene=CLEC4A |
| CTSL | Cathepsin L | Protein Coding |  | 52 | GC09P087725 | | 1.42 | https://www.genecards.org/cgi-bin/carddisp.pl?gene=CTSL |
| MAG | Myelin Associated Glycoprotein | Protein Coding |  | 52 | GC19P035292 | | 1.42 | https://www.genecards.org/cgi-bin/carddisp.pl?gene=MAG |
| HLA-C | Major Histocompatibility Complex, Class I, C | Protein Coding |  | 49 | GC06M031272 | | 1.42 | https://www.genecards.org/cgi-bin/carddisp.pl?gene=HLA-C |
| VAV1 | Vav Guanine Nucleotide Exchange Factor 1 | Protein Coding |  | 53 | GC19P006772 | | 1.42 | https://www.genecards.org/cgi-bin/carddisp.pl?gene=VAV1 |
| FCAR | Fc Fragment Of IgA Receptor | Protein Coding |  | 47 | GC19P055134 | | 1.42 | https://www.genecards.org/cgi-bin/carddisp.pl?gene=FCAR |
| KCNH2 | Potassium Voltage-Gated Channel Subfamily H Member 2 | Protein Coding |  | 57 | GC07M150944 | | 1.41 | https://www.genecards.org/cgi-bin/carddisp.pl?gene=KCNH2 |
| BMPR1A | Bone Morphogenetic Protein Receptor Type 1A | Protein Coding |  | 57 | GC10P086756 | | 1.4 | https://www.genecards.org/cgi-bin/carddisp.pl?gene=BMPR1A |
| PLCB1 | Phospholipase C Beta 1 | Protein Coding |  | 54 | GC20P008061 | | 1.39 | https://www.genecards.org/cgi-bin/carddisp.pl?gene=PLCB1 |
| FOXA2 | Forkhead Box A2 | Protein Coding |  | 50 | GC20M022581 | | 1.39 | https://www.genecards.org/cgi-bin/carddisp.pl?gene=FOXA2 |
| P2RY1 | Purinergic Receptor P2Y1 | Protein Coding |  | 50 | GC03P152835 | | 1.39 | https://www.genecards.org/cgi-bin/carddisp.pl?gene=P2RY1 |
| FYN | FYN Proto-Oncogene, Src Family Tyrosine Kinase | Protein Coding |  | 55 | GC06M111660 | | 1.39 | https://www.genecards.org/cgi-bin/carddisp.pl?gene=FYN |
| GPC3 | Glypican 3 | Protein Coding |  | 52 | GC0XM133535 | | 1.39 | https://www.genecards.org/cgi-bin/carddisp.pl?gene=GPC3 |
| LCN1 | Lipocalin 1 | Protein Coding |  | 45 | GC09P135521 | | 1.39 | https://www.genecards.org/cgi-bin/carddisp.pl?gene=LCN1 |
| TNFRSF6B | TNF Receptor Superfamily Member 6b | Protein Coding |  | 48 | GC20P063696 | | 1.39 | https://www.genecards.org/cgi-bin/carddisp.pl?gene=TNFRSF6B |
| STK16 | Serine/Threonine Kinase 16 | Protein Coding |  | 46 | GC02P219248 | | 1.39 | https://www.genecards.org/cgi-bin/carddisp.pl?gene=STK16 |
| F2RL1 | F2R Like Trypsin Receptor 1 | Protein Coding |  | 52 | GC05P076818 | | 1.39 | https://www.genecards.org/cgi-bin/carddisp.pl?gene=F2RL1 |
| NUMA1 | Nuclear Mitotic Apparatus Protein 1 | Protein Coding |  | 50 | GC11M072002 | | 1.38 | https://www.genecards.org/cgi-bin/carddisp.pl?gene=NUMA1 |
| THRA | Thyroid Hormone Receptor Alpha | Protein Coding |  | 55 | GC17P040058 | | 1.38 | https://www.genecards.org/cgi-bin/carddisp.pl?gene=THRA |
| RFXANK | Regulatory Factor X Associated Ankyrin Containing Protein | Protein Coding |  | 48 | GC19P019192 | | 1.38 | https://www.genecards.org/cgi-bin/carddisp.pl?gene=RFXANK |
| FGA | Fibrinogen Alpha Chain | Protein Coding |  | 54 | GC04M154583 | | 1.38 | https://www.genecards.org/cgi-bin/carddisp.pl?gene=FGA |
| GLI3 | GLI Family Zinc Finger 3 | Protein Coding |  | 56 | GC07M042000 | | 1.38 | https://www.genecards.org/cgi-bin/carddisp.pl?gene=GLI3 |
| CHKA | Choline Kinase Alpha | Protein Coding |  | 48 | GC11M068052 | | 1.38 | https://www.genecards.org/cgi-bin/carddisp.pl?gene=CHKA |
| NOTCH2 | Notch Receptor 2 | Protein Coding |  | 56 | GC01M119911 | | 1.37 | https://www.genecards.org/cgi-bin/carddisp.pl?gene=NOTCH2 |
| TRADD | TNFRSF1A Associated Via Death Domain | Protein Coding |  | 49 | GC16M067154 | | 1.37 | https://www.genecards.org/cgi-bin/carddisp.pl?gene=TRADD |
| FABP5 | Fatty Acid Binding Protein 5 | Protein Coding |  | 47 | GC08P081282 | | 1.37 | https://www.genecards.org/cgi-bin/carddisp.pl?gene=FABP5 |
| TNNT2 | Troponin T2, Cardiac Type | Protein Coding |  | 54 | GC01M201359 | | 1.37 | https://www.genecards.org/cgi-bin/carddisp.pl?gene=TNNT2 |
| FST | Follistatin | Protein Coding |  | 53 | GC05P053480 | | 1.37 | https://www.genecards.org/cgi-bin/carddisp.pl?gene=FST |
| CTSC | Cathepsin C | Protein Coding |  | 51 | GC11M088211 | | 1.36 | https://www.genecards.org/cgi-bin/carddisp.pl?gene=CTSC |
| GOLGA5 | Golgin A5 | Protein Coding |  | 45 | GC14P092794 | | 1.36 | https://www.genecards.org/cgi-bin/carddisp.pl?gene=GOLGA5 |
| FANCG | FA Complementation Group G | Protein Coding |  | 49 | GC09M035073 | | 1.36 | https://www.genecards.org/cgi-bin/carddisp.pl?gene=FANCG |
| HSPG2 | Heparan Sulfate Proteoglycan 2 | Protein Coding |  | 53 | GC01M021822 | | 1.36 | https://www.genecards.org/cgi-bin/carddisp.pl?gene=HSPG2 |
| TRPS1 | Transcriptional Repressor GATA Binding 1 | Protein Coding |  | 51 | GC08M115408 | | 1.35 | https://www.genecards.org/cgi-bin/carddisp.pl?gene=TRPS1 |
| TEK | TEK Receptor Tyrosine Kinase | Protein Coding |  | 57 | GC09P027109 | | 1.35 | https://www.genecards.org/cgi-bin/carddisp.pl?gene=TEK |
| ZMPSTE24 | Zinc Metallopeptidase STE24 | Protein Coding |  | 49 | GC01P040258 | | 1.35 | https://www.genecards.org/cgi-bin/carddisp.pl?gene=ZMPSTE24 |
| LEPR | Leptin Receptor | Protein Coding |  | 55 | GC01P065421 | | 1.35 | https://www.genecards.org/cgi-bin/carddisp.pl?gene=LEPR |
| ORAI1 | ORAI Calcium Release-Activated Calcium Modulator 1 | Protein Coding |  | 50 | GC12P122415 | | 1.35 | https://www.genecards.org/cgi-bin/carddisp.pl?gene=ORAI1 |
| HSPB2 | Heat Shock Protein Family B (Small) Member 2 | Protein Coding |  | 46 | GC11P111913 | | 1.35 | https://www.genecards.org/cgi-bin/carddisp.pl?gene=HSPB2 |
| MAD2L2 | Mitotic Arrest Deficient 2 Like 2 | Protein Coding |  | 50 | GC01M011734 | | 1.34 | https://www.genecards.org/cgi-bin/carddisp.pl?gene=MAD2L2 |
| TREX1 | Three Prime Repair Exonuclease 1 | Protein Coding |  | 48 | GC03P048506 | | 1.34 | https://www.genecards.org/cgi-bin/carddisp.pl?gene=TREX1 |
| DEFA1 | Defensin Alpha 1 | Protein Coding |  | 45 | GC08M006977 | | 1.34 | https://www.genecards.org/cgi-bin/carddisp.pl?gene=DEFA1 |
| C4BPB | Complement Component 4 Binding Protein Beta | Protein Coding |  | 45 | GC01P207088 | | 1.34 | https://www.genecards.org/cgi-bin/carddisp.pl?gene=C4BPB |
| HIST1H4A | Histone Cluster 1 H4 Family Member A | Protein Coding |  | 41 | GC06P026021 | | 1.34 | https://www.genecards.org/cgi-bin/carddisp.pl?gene=HIST1H4A |
| PDP1 | Pyruvate Dehyrogenase Phosphatase Catalytic Subunit 1 | Protein Coding |  | 52 | GC08P093857 | | 1.33 | https://www.genecards.org/cgi-bin/carddisp.pl?gene=PDP1 |
| AARS | Alanyl-TRNA Synthetase | Protein Coding |  | 51 | GC16M070252 | | 1.33 | https://www.genecards.org/cgi-bin/carddisp.pl?gene=AARS |
| CGAS | Cyclic GMP-AMP Synthase | Protein Coding |  | 34 | GC06M073414 | | 1.32 | https://www.genecards.org/cgi-bin/carddisp.pl?gene=CGAS |
| HYAL1 | Hyaluronidase 1 | Protein Coding |  | 52 | GC03M050299 | | 1.32 | https://www.genecards.org/cgi-bin/carddisp.pl?gene=HYAL1 |
| CEBPE | CCAAT Enhancer Binding Protein Epsilon | Protein Coding |  | 48 | GC14M023117 | | 1.32 | https://www.genecards.org/cgi-bin/carddisp.pl?gene=CEBPE |
| SUMO1 | Small Ubiquitin Like Modifier 1 | Protein Coding |  | 53 | GC02M202206 | | 1.32 | https://www.genecards.org/cgi-bin/carddisp.pl?gene=SUMO1 |
| P2RY2 | Purinergic Receptor P2Y2 | Protein Coding |  | 51 | GC11P073217 | | 1.31 | https://www.genecards.org/cgi-bin/carddisp.pl?gene=P2RY2 |
| CNR2 | Cannabinoid Receptor 2 | Protein Coding |  | 51 | GC01M023870 | | 1.31 | https://www.genecards.org/cgi-bin/carddisp.pl?gene=CNR2 |
| SIK2 | Salt Inducible Kinase 2 | Protein Coding |  | 50 | GC11P111605 | | 1.31 | https://www.genecards.org/cgi-bin/carddisp.pl?gene=SIK2 |
| MGAM | Maltase-Glucoamylase | Protein Coding |  | 48 | GC07P144396 | | 1.31 | https://www.genecards.org/cgi-bin/carddisp.pl?gene=MGAM |
| MSI1 | Musashi RNA Binding Protein 1 | Protein Coding |  | 46 | GC12M120341 | | 1.3 | https://www.genecards.org/cgi-bin/carddisp.pl?gene=MSI1 |
| PVALB | Parvalbumin | Protein Coding |  | 45 | GC22M036800 | | 1.3 | https://www.genecards.org/cgi-bin/carddisp.pl?gene=PVALB |
| DIO2 | Iodothyronine Deiodinase 2 | Protein Coding |  | 45 | GC14M080197 | | 1.3 | https://www.genecards.org/cgi-bin/carddisp.pl?gene=DIO2 |
| CLIP2 | CAP-Gly Domain Containing Linker Protein 2 | Protein Coding |  | 43 | GC07P074289 | | 1.3 | https://www.genecards.org/cgi-bin/carddisp.pl?gene=CLIP2 |
| PLA2G6 | Phospholipase A2 Group VI | Protein Coding |  | 54 | GC22M044196 | | 1.29 | https://www.genecards.org/cgi-bin/carddisp.pl?gene=PLA2G6 |
| HCFC1 | Host Cell Factor C1 | Protein Coding |  | 52 | GC0XM153947 | | 1.29 | https://www.genecards.org/cgi-bin/carddisp.pl?gene=HCFC1 |
| FHL2 | Four And A Half LIM Domains 2 | Protein Coding |  | 52 | GC02M105343 | | 1.29 | https://www.genecards.org/cgi-bin/carddisp.pl?gene=FHL2 |
| CYP2D6 | Cytochrome P450 Family 2 Subfamily D Member 6 | Protein Coding |  | 55 | GC22M042916 | | 1.29 | https://www.genecards.org/cgi-bin/carddisp.pl?gene=CYP2D6 |
| KCNJ11 | Potassium Voltage-Gated Channel Subfamily J Member 11 | Protein Coding |  | 54 | GC11M017364 | | 1.29 | https://www.genecards.org/cgi-bin/carddisp.pl?gene=KCNJ11 |
| MAP1LC3B | Microtubule Associated Protein 1 Light Chain 3 Beta | Protein Coding |  | 49 | GC16P087425 | | 1.29 | https://www.genecards.org/cgi-bin/carddisp.pl?gene=MAP1LC3B |
| CPQ | Carboxypeptidase Q | Protein Coding |  | 40 | GC08P096645 | | 1.29 | https://www.genecards.org/cgi-bin/carddisp.pl?gene=CPQ |
| PGD | Phosphogluconate Dehydrogenase | Protein Coding |  | 53 | GC01P010398 | | 1.29 | https://www.genecards.org/cgi-bin/carddisp.pl?gene=PGD |
| DSG3 | Desmoglein 3 | Protein Coding |  | 45 | GC18P031447 | | 1.29 | https://www.genecards.org/cgi-bin/carddisp.pl?gene=DSG3 |
| STAT4 | Signal Transducer And Activator Of Transcription 4 | Protein Coding |  | 52 | GC02M191029 | | 1.28 | https://www.genecards.org/cgi-bin/carddisp.pl?gene=STAT4 |
| PSORS1C1 | Psoriasis Susceptibility 1 Candidate 1 | Protein Coding |  | 34 | GC06P031114 | | 1.28 | https://www.genecards.org/cgi-bin/carddisp.pl?gene=PSORS1C1 |
| ARID1A | AT-Rich Interaction Domain 1A | Protein Coding |  | 51 | GC01P026706 | | 1.28 | https://www.genecards.org/cgi-bin/carddisp.pl?gene=ARID1A |
| HOXA13 | Homeobox A13 | Protein Coding |  | 50 | GC07M027196 | | 1.28 | https://www.genecards.org/cgi-bin/carddisp.pl?gene=HOXA13 |
| LRIG2 | Leucine Rich Repeats And Immunoglobulin Like Domains 2 | Protein Coding |  | 46 | GC01P113073 | | 1.28 | https://www.genecards.org/cgi-bin/carddisp.pl?gene=LRIG2 |
| PWRN1 | Prader-Willi Region Non-Protein Coding RNA 1 | RNA Gene |  | 20 | GC15P024267 | | 1.28 | https://www.genecards.org/cgi-bin/carddisp.pl?gene=PWRN1 |
| BMX | BMX Non-Receptor Tyrosine Kinase | Protein Coding |  | 51 | GC0XP015392 | | 1.28 | https://www.genecards.org/cgi-bin/carddisp.pl?gene=BMX |
| CMA1 | Chymase 1 | Protein Coding |  | 50 | GC14M024506 | | 1.28 | https://www.genecards.org/cgi-bin/carddisp.pl?gene=CMA1 |
| SP100 | SP100 Nuclear Antigen | Protein Coding |  | 47 | GC02P230415 | | 1.28 | https://www.genecards.org/cgi-bin/carddisp.pl?gene=SP100 |
| AHCY | Adenosylhomocysteinase | Protein Coding |  | 57 | GC20M034237 | | 1.28 | https://www.genecards.org/cgi-bin/carddisp.pl?gene=AHCY |
| HADHA | Hydroxyacyl-CoA Dehydrogenase Trifunctional Multienzyme Complex Subunit Alpha | Protein Coding |  | 53 | GC02M026190 | | 1.28 | https://www.genecards.org/cgi-bin/carddisp.pl?gene=HADHA |
| CDC5L | Cell Division Cycle 5 Like | Protein Coding |  | 44 | GC06P044387 | | 1.28 | https://www.genecards.org/cgi-bin/carddisp.pl?gene=CDC5L |
| PLRG1 | Pleiotropic Regulator 1 | Protein Coding |  | 43 | GC04M154534 | | 1.28 | https://www.genecards.org/cgi-bin/carddisp.pl?gene=PLRG1 |
| TRIP4 | Thyroid Hormone Receptor Interactor 4 | Protein Coding |  | 46 | GC15P069586 | | 1.28 | https://www.genecards.org/cgi-bin/carddisp.pl?gene=TRIP4 |
| STUB1 | STIP1 Homology And U-Box Containing Protein 1 | Protein Coding |  | 52 | GC16P001074 | | 1.28 | https://www.genecards.org/cgi-bin/carddisp.pl?gene=STUB1 |
| PIAS1 | Protein Inhibitor Of Activated STAT 1 | Protein Coding |  | 51 | GC15P068054 | | 1.27 | https://www.genecards.org/cgi-bin/carddisp.pl?gene=PIAS1 |
| SLX4 | SLX4 Structure-Specific Endonuclease Subunit | Protein Coding |  | 45 | GC16M003581 | | 1.27 | https://www.genecards.org/cgi-bin/carddisp.pl?gene=SLX4 |
| CD82 | CD82 Molecule | Protein Coding |  | 49 | GC11P044586 | | 1.27 | https://www.genecards.org/cgi-bin/carddisp.pl?gene=CD82 |
| FANCF | FA Complementation Group F | Protein Coding |  | 48 | GC11M022600 | | 1.27 | https://www.genecards.org/cgi-bin/carddisp.pl?gene=FANCF |
| SIN3A | SIN3 Transcription Regulator Family Member A | Protein Coding |  | 52 | GC15M075369 | | 1.27 | https://www.genecards.org/cgi-bin/carddisp.pl?gene=SIN3A |
| PPM1F | Protein Phosphatase, Mg2+/Mn2+ Dependent 1F | Protein Coding |  | 45 | GC22M021919 | | 1.27 | https://www.genecards.org/cgi-bin/carddisp.pl?gene=PPM1F |
| PAG1 | Phosphoprotein Membrane Anchor With Glycosphingolipid Microdomains 1 | Protein Coding |  | 43 | GC08M080967 | | 1.27 | https://www.genecards.org/cgi-bin/carddisp.pl?gene=PAG1 |
| CBLB | Cbl Proto-Oncogene B | Protein Coding |  | 48 | GC03M105655 | | 1.26 | https://www.genecards.org/cgi-bin/carddisp.pl?gene=CBLB |
| ERVK-6 | Endogenous Retrovirus Group K Member 6, Envelope | Protein Coding |  | 20 | GC07U903184 | | 1.26 | https://www.genecards.org/cgi-bin/carddisp.pl?gene=ERVK-6 |
| SLC4A1 | Solute Carrier Family 4 Member 1 (Diego Blood Group) | Protein Coding |  | 54 | GC17M044249 | | 1.26 | https://www.genecards.org/cgi-bin/carddisp.pl?gene=SLC4A1 |
| ADIPOQ | Adiponectin, C1Q And Collagen Domain Containing | Protein Coding |  | 51 | GC03P186842 | | 1.26 | https://www.genecards.org/cgi-bin/carddisp.pl?gene=ADIPOQ |
| FOXC2 | Forkhead Box C2 | Protein Coding |  | 50 | GC16P086567 | | 1.26 | https://www.genecards.org/cgi-bin/carddisp.pl?gene=FOXC2 |
| POLE | DNA Polymerase Epsilon, Catalytic Subunit | Protein Coding |  | 55 | GC12M132624 | | 1.26 | https://www.genecards.org/cgi-bin/carddisp.pl?gene=POLE |
| FANCL | FA Complementation Group L | Protein Coding |  | 50 | GC02M058127 | | 1.26 | https://www.genecards.org/cgi-bin/carddisp.pl?gene=FANCL |
| SLC29A1 | Solute Carrier Family 29 Member 1 (Augustine Blood Group) | Protein Coding |  | 54 | GC06P044219 | | 1.25 | https://www.genecards.org/cgi-bin/carddisp.pl?gene=SLC29A1 |
| CHRNA7 | Cholinergic Receptor Nicotinic Alpha 7 Subunit | Protein Coding |  | 52 | GC15P031923 | | 1.25 | https://www.genecards.org/cgi-bin/carddisp.pl?gene=CHRNA7 |
| FKBPL | FKBP Prolyl Isomerase Like | Protein Coding |  | 40 | GC06M032209 | | 1.24 | https://www.genecards.org/cgi-bin/carddisp.pl?gene=FKBPL |
| CTSS | Cathepsin S | Protein Coding |  | 52 | GC01M150702 | | 1.24 | https://www.genecards.org/cgi-bin/carddisp.pl?gene=CTSS |
| DYRK1A | Dual Specificity Tyrosine Phosphorylation Regulated Kinase 1A | Protein Coding |  | 57 | GC21P037365 | | 1.24 | https://www.genecards.org/cgi-bin/carddisp.pl?gene=DYRK1A |
| CLEC12A | C-Type Lectin Domain Family 12 Member A | Protein Coding |  | 43 | GC12P009951 | | 1.24 | https://www.genecards.org/cgi-bin/carddisp.pl?gene=CLEC12A |
| IL15RA | Interleukin 15 Receptor Subunit Alpha | Protein Coding |  | 47 | GC10M005991 | | 1.24 | https://www.genecards.org/cgi-bin/carddisp.pl?gene=IL15RA |
| MDK | Midkine | Protein Coding |  | 50 | GC11P046402 | | 1.24 | https://www.genecards.org/cgi-bin/carddisp.pl?gene=MDK |
| AHSG | Alpha 2-HS Glycoprotein | Protein Coding |  | 50 | GC03P186612 | | 1.24 | https://www.genecards.org/cgi-bin/carddisp.pl?gene=AHSG |
| FTSJ1 | FtsJ RNA 2'-O-Methyltransferase 1 | Protein Coding |  | 47 | GC0XP048476 | | 1.23 | https://www.genecards.org/cgi-bin/carddisp.pl?gene=FTSJ1 |
| REEP1 | Receptor Accessory Protein 1 | Protein Coding |  | 46 | GC02M086213 | | 1.23 | https://www.genecards.org/cgi-bin/carddisp.pl?gene=REEP1 |
| UHMK1 | U2AF Homology Motif Kinase 1 | Protein Coding |  | 45 | GC01P162467 | | 1.23 | https://www.genecards.org/cgi-bin/carddisp.pl?gene=UHMK1 |
| SGTA | Small Glutamine Rich Tetratricopeptide Repeat Containing Alpha | Protein Coding |  | 44 | GC19M002754 | | 1.23 | https://www.genecards.org/cgi-bin/carddisp.pl?gene=SGTA |
| DAND5 | DAN Domain BMP Antagonist Family Member 5 | Protein Coding |  | 42 | GC19P012965 | | 1.23 | https://www.genecards.org/cgi-bin/carddisp.pl?gene=DAND5 |
| SMN1 | Survival Of Motor Neuron 1, Telomeric | Protein Coding |  | 47 | GC05P070924 | | 1.23 | https://www.genecards.org/cgi-bin/carddisp.pl?gene=SMN1 |
| NEDD4 | NEDD4 E3 Ubiquitin Protein Ligase | Protein Coding |  | 52 | GC15M055826 | | 1.23 | https://www.genecards.org/cgi-bin/carddisp.pl?gene=NEDD4 |
| WNT5A | Wnt Family Member 5A | Protein Coding |  | 56 | GC03M055474 | | 1.23 | https://www.genecards.org/cgi-bin/carddisp.pl?gene=WNT5A |
| NEDD8 | NEDD8 Ubiquitin Like Modifier | Protein Coding |  | 46 | GC14M024216 | | 1.23 | https://www.genecards.org/cgi-bin/carddisp.pl?gene=NEDD8 |
| IGH | Immunoglobulin Heavy Locus | Protein Coding |  | 20 | GC14M105761 | | 1.23 | https://www.genecards.org/cgi-bin/carddisp.pl?gene=IGH |
| DMD | Dystrophin | Protein Coding |  | 52 | GC0XM031047 | | 1.23 | https://www.genecards.org/cgi-bin/carddisp.pl?gene=DMD |
| HMGCR | 3-Hydroxy-3-Methylglutaryl-CoA Reductase | Protein Coding |  | 52 | GC05P075336 | | 1.23 | https://www.genecards.org/cgi-bin/carddisp.pl?gene=HMGCR |
| HPR | Haptoglobin-Related Protein | Protein Coding |  | 46 | GC16P072097 | | 1.22 | https://www.genecards.org/cgi-bin/carddisp.pl?gene=HPR |
| MSH3 | MutS Homolog 3 | Protein Coding |  | 48 | GC05P080654 | | 1.22 | https://www.genecards.org/cgi-bin/carddisp.pl?gene=MSH3 |
| CD3E | CD3e Molecule | Protein Coding |  | 53 | GC11P118304 | | 1.21 | https://www.genecards.org/cgi-bin/carddisp.pl?gene=CD3E |
| CDK5 | Cyclin Dependent Kinase 5 | Protein Coding |  | 59 | GC07M151053 | | 1.2 | https://www.genecards.org/cgi-bin/carddisp.pl?gene=CDK5 |
| HSPA2 | Heat Shock Protein Family A (Hsp70) Member 2 | Protein Coding |  | 50 | GC14P064535 | | 1.2 | https://www.genecards.org/cgi-bin/carddisp.pl?gene=HSPA2 |
| CD1A | CD1a Molecule | Protein Coding |  | 48 | GC01P158255 | | 1.2 | https://www.genecards.org/cgi-bin/carddisp.pl?gene=CD1A |
| MIR92A1 | MicroRNA 92a-1 | RNA Gene |  | 19 | GC13P091384 | | 1.2 | https://www.genecards.org/cgi-bin/carddisp.pl?gene=MIR92A1 |
| FABP3 | Fatty Acid Binding Protein 3 | Protein Coding |  | 50 | GC01M031365 | | 1.2 | https://www.genecards.org/cgi-bin/carddisp.pl?gene=FABP3 |
| ATXN3 | Ataxin 3 | Protein Coding |  | 51 | GC14M092430 | | 1.2 | https://www.genecards.org/cgi-bin/carddisp.pl?gene=ATXN3 |
| PGAM1 | Phosphoglycerate Mutase 1 | Protein Coding |  | 50 | GC10P097426 | | 1.2 | https://www.genecards.org/cgi-bin/carddisp.pl?gene=PGAM1 |
| PRKCZ | Protein Kinase C Zeta | Protein Coding |  | 55 | GC01P002050 | | 1.19 | https://www.genecards.org/cgi-bin/carddisp.pl?gene=PRKCZ |
| GSS | Glutathione Synthetase | Protein Coding |  | 54 | GC20M034928 | | 1.19 | https://www.genecards.org/cgi-bin/carddisp.pl?gene=GSS |
| OLFM4 | Olfactomedin 4 | Protein Coding |  | 44 | GC13P053028 | | 1.19 | https://www.genecards.org/cgi-bin/carddisp.pl?gene=OLFM4 |
| NOTCH4 | Notch Receptor 4 | Protein Coding |  | 51 | GC06M032203 | | 1.19 | https://www.genecards.org/cgi-bin/carddisp.pl?gene=NOTCH4 |
| HES1 | Hes Family BHLH Transcription Factor 1 | Protein Coding |  | 50 | GC03P194136 | | 1.19 | https://www.genecards.org/cgi-bin/carddisp.pl?gene=HES1 |
| ACTR3 | Actin Related Protein 3 | Protein Coding |  | 48 | GC02P113889 | | 1.19 | https://www.genecards.org/cgi-bin/carddisp.pl?gene=ACTR3 |
| ERLIN1 | ER Lipid Raft Associated 1 | Protein Coding |  | 47 | GC10M100150 | | 1.19 | https://www.genecards.org/cgi-bin/carddisp.pl?gene=ERLIN1 |
| CPT1A | Carnitine Palmitoyltransferase 1A | Protein Coding |  | 55 | GC11M068772 | | 1.18 | https://www.genecards.org/cgi-bin/carddisp.pl?gene=CPT1A |
| SLC5A6 | Solute Carrier Family 5 Member 6 | Protein Coding |  | 50 | GC02M027164 | | 1.18 | https://www.genecards.org/cgi-bin/carddisp.pl?gene=SLC5A6 |
| OPN1LW | Opsin 1, Long Wave Sensitive | Protein Coding |  | 42 | GC0XP154144 | | 1.18 | https://www.genecards.org/cgi-bin/carddisp.pl?gene=OPN1LW |
| BTLA | B And T Lymphocyte Associated | Protein Coding |  | 44 | GC03M112463 | | 1.18 | https://www.genecards.org/cgi-bin/carddisp.pl?gene=BTLA |
| HSPB8 | Heat Shock Protein Family B (Small) Member 8 | Protein Coding |  | 51 | GC12P119178 | | 1.18 | https://www.genecards.org/cgi-bin/carddisp.pl?gene=HSPB8 |
| HIST4H4 | Histone Cluster 4 H4 | Protein Coding |  | 45 | GC12M014767 | | 1.17 | https://www.genecards.org/cgi-bin/carddisp.pl?gene=HIST4H4 |
| AKR1B10 | Aldo-Keto Reductase Family 1 Member B10 | Protein Coding |  | 50 | GC07P134527 | | 1.17 | https://www.genecards.org/cgi-bin/carddisp.pl?gene=AKR1B10 |
| GJB1 | Gap Junction Protein Beta 1 | Protein Coding |  | 54 | GC0XP071215 | | 1.17 | https://www.genecards.org/cgi-bin/carddisp.pl?gene=GJB1 |
| SNRPD3 | Small Nuclear Ribonucleoprotein D3 Polypeptide | Protein Coding |  | 42 | GC22P024555 | | 1.17 | https://www.genecards.org/cgi-bin/carddisp.pl?gene=SNRPD3 |
| C1QC | Complement C1q C Chain | Protein Coding |  | 51 | GC01P022643 | | 1.16 | https://www.genecards.org/cgi-bin/carddisp.pl?gene=C1QC |
| HABP2 | Hyaluronan Binding Protein 2 | Protein Coding |  | 50 | GC10P113550 | | 1.16 | https://www.genecards.org/cgi-bin/carddisp.pl?gene=HABP2 |
| ANG | Angiogenin | Protein Coding |  | 51 | GC14P020789 | | 1.16 | https://www.genecards.org/cgi-bin/carddisp.pl?gene=ANG |
| AMD1 | Adenosylmethionine Decarboxylase 1 | Protein Coding |  | 51 | GC06P110814 | | 1.15 | https://www.genecards.org/cgi-bin/carddisp.pl?gene=AMD1 |
| PIDD1 | P53-Induced Death Domain Protein 1 | Protein Coding |  | 40 | GC11M000800 | | 1.15 | https://www.genecards.org/cgi-bin/carddisp.pl?gene=PIDD1 |
| RPS6KA5 | Ribosomal Protein S6 Kinase A5 | Protein Coding |  | 50 | GC14M090847 | | 1.15 | https://www.genecards.org/cgi-bin/carddisp.pl?gene=RPS6KA5 |
| CXCL6 | C-X-C Motif Chemokine Ligand 6 | Protein Coding |  | 46 | GC04P073837 | | 1.15 | https://www.genecards.org/cgi-bin/carddisp.pl?gene=CXCL6 |
| ST2 | Suppression Of Tumorigenicity 2 | Genetic Locus |  | 12 | GC11U990127 | | 1.15 | https://www.genecards.org/cgi-bin/carddisp.pl?gene=ST2 |
| ATF6 | Activating Transcription Factor 6 | Protein Coding |  | 53 | GC01P161766 | | 1.15 | https://www.genecards.org/cgi-bin/carddisp.pl?gene=ATF6 |
| RPSA | Ribosomal Protein SA | Protein Coding |  | 51 | GC03P039406 | | 1.15 | https://www.genecards.org/cgi-bin/carddisp.pl?gene=RPSA |
| TNXA | Tenascin XA (Pseudogene) | Pseudogene |  | 29 | GC06M032182 | | 1.15 | https://www.genecards.org/cgi-bin/carddisp.pl?gene=TNXA |
| UBE2T | Ubiquitin Conjugating Enzyme E2 T | Protein Coding |  | 50 | GC01M202300 | | 1.14 | https://www.genecards.org/cgi-bin/carddisp.pl?gene=UBE2T |
| NHP2 | NHP2 Ribonucleoprotein | Protein Coding |  | 49 | GC05M178149 | | 1.14 | https://www.genecards.org/cgi-bin/carddisp.pl?gene=NHP2 |
| CD3G | CD3g Molecule | Protein Coding |  | 53 | GC11P118344 | | 1.13 | https://www.genecards.org/cgi-bin/carddisp.pl?gene=CD3G |
| CLEC4E | C-Type Lectin Domain Family 4 Member E | Protein Coding |  | 43 | GC12M008535 | | 1.13 | https://www.genecards.org/cgi-bin/carddisp.pl?gene=CLEC4E |
| TRAF2 | TNF Receptor Associated Factor 2 | Protein Coding |  | 51 | GC09P136881 | | 1.13 | https://www.genecards.org/cgi-bin/carddisp.pl?gene=TRAF2 |
| GLRX | Glutaredoxin | Protein Coding |  | 51 | GC05M095752 | | 1.13 | https://www.genecards.org/cgi-bin/carddisp.pl?gene=GLRX |
| SOAT1 | Sterol O-Acyltransferase 1 | Protein Coding |  | 50 | GC01P179262 | | 1.13 | https://www.genecards.org/cgi-bin/carddisp.pl?gene=SOAT1 |
| FCN1 | Ficolin 1 | Protein Coding |  | 45 | GC09M134942 | | 1.13 | https://www.genecards.org/cgi-bin/carddisp.pl?gene=FCN1 |
| NDUFS4 | NADH:Ubiquinone Oxidoreductase Subunit S4 | Protein Coding |  | 49 | GC05P053560 | | 1.12 | https://www.genecards.org/cgi-bin/carddisp.pl?gene=NDUFS4 |
| DBH | Dopamine Beta-Hydroxylase | Protein Coding |  | 56 | GC09P133636 | | 1.12 | https://www.genecards.org/cgi-bin/carddisp.pl?gene=DBH |
| PNMT | Phenylethanolamine N-Methyltransferase | Protein Coding |  | 51 | GC17P039667 | | 1.12 | https://www.genecards.org/cgi-bin/carddisp.pl?gene=PNMT |
| EREG | Epiregulin | Protein Coding |  | 48 | GC04P074366 | | 1.12 | https://www.genecards.org/cgi-bin/carddisp.pl?gene=EREG |
| SERPINB2 | Serpin Family B Member 2 | Protein Coding |  | 50 | GC18P063871 | | 1.12 | https://www.genecards.org/cgi-bin/carddisp.pl?gene=SERPINB2 |
| HPS1 | HPS1 Biogenesis Of Lysosomal Organelles Complex 3 Subunit 1 | Protein Coding |  | 47 | GC10M098416 | | 1.12 | https://www.genecards.org/cgi-bin/carddisp.pl?gene=HPS1 |
| ICAM3 | Intercellular Adhesion Molecule 3 | Protein Coding |  | 47 | GC19M010335 | | 1.12 | https://www.genecards.org/cgi-bin/carddisp.pl?gene=ICAM3 |
| MPIG6B | Megakaryocyte And Platelet Inhibitory Receptor G6b | Protein Coding |  | 37 | GC06P032543 | | 1.11 | https://www.genecards.org/cgi-bin/carddisp.pl?gene=MPIG6B |
| BTN1A1 | Butyrophilin Subfamily 1 Member A1 | Protein Coding |  | 45 | GC06P026499 | | 1.11 | https://www.genecards.org/cgi-bin/carddisp.pl?gene=BTN1A1 |
| GPI | Glucose-6-Phosphate Isomerase | Protein Coding |  | 53 | GC19P034359 | | 1.11 | https://www.genecards.org/cgi-bin/carddisp.pl?gene=GPI |
| BTRC | Beta-Transducin Repeat Containing E3 Ubiquitin Protein Ligase | Protein Coding |  | 52 | GC10P101354 | | 1.11 | https://www.genecards.org/cgi-bin/carddisp.pl?gene=BTRC |
| IRS2 | Insulin Receptor Substrate 2 | Protein Coding |  | 51 | GC13M109752 | | 1.1 | https://www.genecards.org/cgi-bin/carddisp.pl?gene=IRS2 |
| FANCI | FA Complementation Group I | Protein Coding |  | 48 | GC15P089243 | | 1.1 | https://www.genecards.org/cgi-bin/carddisp.pl?gene=FANCI |
| SCGB2A1 | Secretoglobin Family 2A Member 1 | Protein Coding |  | 39 | GC11P062227 | | 1.1 | https://www.genecards.org/cgi-bin/carddisp.pl?gene=SCGB2A1 |
| SCN10A | Sodium Voltage-Gated Channel Alpha Subunit 10 | Protein Coding |  | 53 | GC03M038713 | | 1.1 | https://www.genecards.org/cgi-bin/carddisp.pl?gene=SCN10A |
| PSENEN | Presenilin Enhancer, Gamma-Secretase Subunit | Protein Coding |  | 51 | GC19P037195 | | 1.1 | https://www.genecards.org/cgi-bin/carddisp.pl?gene=PSENEN |
| FANCE | FA Complementation Group E | Protein Coding |  | 49 | GC06P043125 | | 1.1 | https://www.genecards.org/cgi-bin/carddisp.pl?gene=FANCE |
| NOP10 | NOP10 Ribonucleoprotein | Protein Coding |  | 46 | GC15M034341 | | 1.1 | https://www.genecards.org/cgi-bin/carddisp.pl?gene=NOP10 |
| CYP4F22 | Cytochrome P450 Family 4 Subfamily F Member 22 | Protein Coding |  | 45 | GC19P015484 | | 1.1 | https://www.genecards.org/cgi-bin/carddisp.pl?gene=CYP4F22 |
| FAH | Fumarylacetoacetate Hydrolase | Protein Coding |  | 53 | GC15P080152 | | 1.1 | https://www.genecards.org/cgi-bin/carddisp.pl?gene=FAH |
| SKP1 | S-Phase Kinase Associated Protein 1 | Protein Coding |  | 48 | GC05M134148 | | 1.09 | https://www.genecards.org/cgi-bin/carddisp.pl?gene=SKP1 |
| BLK | BLK Proto-Oncogene, Src Family Tyrosine Kinase | Protein Coding |  | 57 | GC08P011388 | | 1.09 | https://www.genecards.org/cgi-bin/carddisp.pl?gene=BLK |
| LRRK2 | Leucine Rich Repeat Kinase 2 | Protein Coding |  | 56 | GC12P040196 | | 1.09 | https://www.genecards.org/cgi-bin/carddisp.pl?gene=LRRK2 |
| TLR8 | Toll Like Receptor 8 | Protein Coding |  | 53 | GC0XP012924 | | 1.09 | https://www.genecards.org/cgi-bin/carddisp.pl?gene=TLR8 |
| IFRD1 | Interferon Related Developmental Regulator 1 | Protein Coding |  | 46 | GC07P112422 | | 1.09 | https://www.genecards.org/cgi-bin/carddisp.pl?gene=IFRD1 |
| FKTN | Fukutin | Protein Coding |  | 45 | GC09P105558 | | 1.09 | https://www.genecards.org/cgi-bin/carddisp.pl?gene=FKTN |
| STAB2 | Stabilin 2 | Protein Coding |  | 44 | GC12P103587 | | 1.09 | https://www.genecards.org/cgi-bin/carddisp.pl?gene=STAB2 |
| IRF2BP2 | Interferon Regulatory Factor 2 Binding Protein 2 | Protein Coding |  | 42 | GC01M234604 | | 1.09 | https://www.genecards.org/cgi-bin/carddisp.pl?gene=IRF2BP2 |
| RNF5 | Ring Finger Protein 5 | Protein Coding |  | 46 | GC06P032530 | | 1.09 | https://www.genecards.org/cgi-bin/carddisp.pl?gene=RNF5 |
| HTT | Huntingtin | Protein Coding |  | 50 | GC04P003041 | | 1.08 | https://www.genecards.org/cgi-bin/carddisp.pl?gene=HTT |
| PAPPA | Pappalysin 1 | Protein Coding |  | 47 | GC09P116162 | | 1.08 | https://www.genecards.org/cgi-bin/carddisp.pl?gene=PAPPA |
| WDR5 | WD Repeat Domain 5 | Protein Coding |  | 50 | GC09P134135 | | 1.08 | https://www.genecards.org/cgi-bin/carddisp.pl?gene=WDR5 |
| ARX | Aristaless Related Homeobox | Protein Coding |  | 48 | GC0XM025021 | | 1.08 | https://www.genecards.org/cgi-bin/carddisp.pl?gene=ARX |
| SLC1A2 | Solute Carrier Family 1 Member 2 | Protein Coding |  | 56 | GC11M035272 | | 1.08 | https://www.genecards.org/cgi-bin/carddisp.pl?gene=SLC1A2 |
| FANCM | FA Complementation Group M | Protein Coding |  | 46 | GC14P045135 | | 1.07 | https://www.genecards.org/cgi-bin/carddisp.pl?gene=FANCM |
| CYB5R3 | Cytochrome B5 Reductase 3 | Protein Coding |  | 51 | GC22M042617 | | 1.07 | https://www.genecards.org/cgi-bin/carddisp.pl?gene=CYB5R3 |
| LAMP1 | Lysosomal Associated Membrane Protein 1 | Protein Coding |  | 49 | GC13P113297 | | 1.07 | https://www.genecards.org/cgi-bin/carddisp.pl?gene=LAMP1 |
| DEFA3 | Defensin Alpha 3 | Protein Coding |  | 42 | GC08M007015 | | 1.07 | https://www.genecards.org/cgi-bin/carddisp.pl?gene=DEFA3 |
| PRPF19 | Pre-MRNA Processing Factor 19 | Protein Coding |  | 43 | GC11M060890 | | 1.07 | https://www.genecards.org/cgi-bin/carddisp.pl?gene=PRPF19 |
| TMPRSS2 | Transmembrane Serine Protease 2 | Protein Coding |  | 48 | GC21M041464 | | 1.07 | https://www.genecards.org/cgi-bin/carddisp.pl?gene=TMPRSS2 |
| RIPK1 | Receptor Interacting Serine/Threonine Kinase 1 | Protein Coding |  | 55 | GC06P003064 | | 1.07 | https://www.genecards.org/cgi-bin/carddisp.pl?gene=RIPK1 |
| BMPR1B | Bone Morphogenetic Protein Receptor Type 1B | Protein Coding |  | 56 | GC04P094757 | | 1.07 | https://www.genecards.org/cgi-bin/carddisp.pl?gene=BMPR1B |
| ALDH9A1 | Aldehyde Dehydrogenase 9 Family Member A1 | Protein Coding |  | 48 | GC01M165632 | | 1.06 | https://www.genecards.org/cgi-bin/carddisp.pl?gene=ALDH9A1 |
| WRAP53 | WD Repeat Containing Antisense To TP53 | Protein Coding |  | 47 | GC17P007847 | | 1.06 | https://www.genecards.org/cgi-bin/carddisp.pl?gene=WRAP53 |
| GPRC5A | G Protein-Coupled Receptor Class C Group 5 Member A | Protein Coding |  | 46 | GC12P012890 | | 1.06 | https://www.genecards.org/cgi-bin/carddisp.pl?gene=GPRC5A |
| LYVE1 | Lymphatic Vessel Endothelial Hyaluronan Receptor 1 | Protein Coding |  | 48 | GC11M010610 | | 1.06 | https://www.genecards.org/cgi-bin/carddisp.pl?gene=LYVE1 |
| FARSB | Phenylalanyl-TRNA Synthetase Subunit Beta | Protein Coding |  | 50 | GC02M222570 | | 1.06 | https://www.genecards.org/cgi-bin/carddisp.pl?gene=FARSB |
| CPVL | Carboxypeptidase Vitellogenic Like | Protein Coding |  | 46 | GC07M028995 | | 1.06 | https://www.genecards.org/cgi-bin/carddisp.pl?gene=CPVL |
| LGALS9 | Galectin 9 | Protein Coding |  | 44 | GC17P027629 | | 1.06 | https://www.genecards.org/cgi-bin/carddisp.pl?gene=LGALS9 |
| CLCN3 | Chloride Voltage-Gated Channel 3 | Protein Coding |  | 48 | GC04P169612 | | 1.06 | https://www.genecards.org/cgi-bin/carddisp.pl?gene=CLCN3 |
| TPMT | Thiopurine S-Methyltransferase | Protein Coding |  | 54 | GC06M018072 | | 1.05 | https://www.genecards.org/cgi-bin/carddisp.pl?gene=TPMT |
| B4GALT1 | Beta-1,4-Galactosyltransferase 1 | Protein Coding |  | 51 | GC09M033100 | | 1.05 | https://www.genecards.org/cgi-bin/carddisp.pl?gene=B4GALT1 |
| NGB | Neuroglobin | Protein Coding |  | 43 | GC14M077265 | | 1.05 | https://www.genecards.org/cgi-bin/carddisp.pl?gene=NGB |
| ATP7B | ATPase Copper Transporting Beta | Protein Coding |  | 54 | GC13M051905 | | 1.04 | https://www.genecards.org/cgi-bin/carddisp.pl?gene=ATP7B |
| DNAJB2 | DnaJ Heat Shock Protein Family (Hsp40) Member B2 | Protein Coding |  | 49 | GC02P219279 | | 1.04 | https://www.genecards.org/cgi-bin/carddisp.pl?gene=DNAJB2 |
| TUBA1A | Tubulin Alpha 1a | Protein Coding |  | 54 | GC12M049184 | | 1.04 | https://www.genecards.org/cgi-bin/carddisp.pl?gene=TUBA1A |
| ARF1 | ADP Ribosylation Factor 1 | Protein Coding |  | 52 | GC01P228082 | | 1.04 | https://www.genecards.org/cgi-bin/carddisp.pl?gene=ARF1 |
| PAFAH1B2 | Platelet Activating Factor Acetylhydrolase 1b Catalytic Subunit 2 | Protein Coding |  | 50 | GC11P117144 | | 1.04 | https://www.genecards.org/cgi-bin/carddisp.pl?gene=PAFAH1B2 |
| SCYL1 | SCY1 Like Pseudokinase 1 | Protein Coding |  | 48 | GC11P065525 | | 1.04 | https://www.genecards.org/cgi-bin/carddisp.pl?gene=SCYL1 |
| NUDC | Nuclear Distribution C, Dynein Complex Regulator | Protein Coding |  | 48 | GC01P026932 | | 1.04 | https://www.genecards.org/cgi-bin/carddisp.pl?gene=NUDC |
| MPC1 | Mitochondrial Pyruvate Carrier 1 | Protein Coding |  | 47 | GC06M166364 | | 1.04 | https://www.genecards.org/cgi-bin/carddisp.pl?gene=MPC1 |
| HBS1L | HBS1 Like Translational GTPase | Protein Coding |  | 46 | GC06M134904 | | 1.04 | https://www.genecards.org/cgi-bin/carddisp.pl?gene=HBS1L |
| MPP5 | Membrane Palmitoylated Protein 5 | Protein Coding |  | 45 | GC14P067241 | | 1.04 | https://www.genecards.org/cgi-bin/carddisp.pl?gene=MPP5 |
| RBBP5 | RB Binding Protein 5, Histone Lysine Methyltransferase Complex Subunit | Protein Coding |  | 45 | GC01M205055 | | 1.04 | https://www.genecards.org/cgi-bin/carddisp.pl?gene=RBBP5 |
| IFT80 | Intraflagellar Transport 80 | Protein Coding |  | 44 | GC03M160256 | | 1.04 | https://www.genecards.org/cgi-bin/carddisp.pl?gene=IFT80 |
| RPP30 | Ribonuclease P/MRP Subunit P30 | Protein Coding |  | 44 | GC10P090871 | | 1.04 | https://www.genecards.org/cgi-bin/carddisp.pl?gene=RPP30 |
| NOP2 | NOP2 Nucleolar Protein | Protein Coding |  | 42 | GC12M006556 | | 1.04 | https://www.genecards.org/cgi-bin/carddisp.pl?gene=NOP2 |
| TUBA3D | Tubulin Alpha 3d | Protein Coding |  | 42 | GC02P131892 | | 1.04 | https://www.genecards.org/cgi-bin/carddisp.pl?gene=TUBA3D |
| ZNF346 | Zinc Finger Protein 346 | Protein Coding |  | 42 | GC05P177022 | | 1.04 | https://www.genecards.org/cgi-bin/carddisp.pl?gene=ZNF346 |
| KIF1BP | KIF1 Binding Protein | Protein Coding |  | 41 | GC10P068988 | | 1.04 | https://www.genecards.org/cgi-bin/carddisp.pl?gene=KIF1BP |
| DUS2 | Dihydrouridine Synthase 2 | Protein Coding |  | 41 | GC16P068021 | | 1.04 | https://www.genecards.org/cgi-bin/carddisp.pl?gene=DUS2 |
| HRH1 | Histamine Receptor H1 | Protein Coding |  | 53 | GC03P011113 | | 1.04 | https://www.genecards.org/cgi-bin/carddisp.pl?gene=HRH1 |
| LAT | Linker For Activation Of T Cells | Protein Coding |  | 52 | GC16P028998 | | 1.04 | https://www.genecards.org/cgi-bin/carddisp.pl?gene=LAT |
| PTS | 6-Pyruvoyltetrahydropterin Synthase | Protein Coding |  | 53 | GC11P112226 | | 1.03 | https://www.genecards.org/cgi-bin/carddisp.pl?gene=PTS |
| LTB4R | Leukotriene B4 Receptor | Protein Coding |  | 50 | GC14P024311 | | 1.02 | https://www.genecards.org/cgi-bin/carddisp.pl?gene=LTB4R |
| SSTR4 | Somatostatin Receptor 4 | Protein Coding |  | 47 | GC20P023035 | | 1.02 | https://www.genecards.org/cgi-bin/carddisp.pl?gene=SSTR4 |
| PROZ | Protein Z, Vitamin K Dependent Plasma Glycoprotein | Protein Coding |  | 46 | GC13P113158 | | 1.02 | https://www.genecards.org/cgi-bin/carddisp.pl?gene=PROZ |
| POU2AF1 | POU Class 2 Homeobox Associating Factor 1 | Protein Coding |  | 43 | GC11M111352 | | 1.02 | https://www.genecards.org/cgi-bin/carddisp.pl?gene=POU2AF1 |
| EVPL | Envoplakin | Protein Coding |  | 42 | GC17M076004 | | 1.01 | https://www.genecards.org/cgi-bin/carddisp.pl?gene=EVPL |
| CEBPB | CCAAT Enhancer Binding Protein Beta | Protein Coding |  | 50 | GC20P050190 | | 1.01 | https://www.genecards.org/cgi-bin/carddisp.pl?gene=CEBPB |
| ICMT | Isoprenylcysteine Carboxyl Methyltransferase | Protein Coding |  | 48 | GC01M006282 | | 1.01 | https://www.genecards.org/cgi-bin/carddisp.pl?gene=ICMT |
| RLN1 | Relaxin 1 | Protein Coding |  | 40 | GC09M005324 | | 1 | https://www.genecards.org/cgi-bin/carddisp.pl?gene=RLN1 |
| ECM1 | Extracellular Matrix Protein 1 | Protein Coding |  | 50 | GC01P150508 | | 1 | https://www.genecards.org/cgi-bin/carddisp.pl?gene=ECM1 |
| P2RY12 | Purinergic Receptor P2Y12 | Protein Coding |  | 54 | GC03M151336 | | 0.99 | https://www.genecards.org/cgi-bin/carddisp.pl?gene=P2RY12 |
| DCTN1 | Dynactin Subunit 1 | Protein Coding |  | 53 | GC02M074361 | | 0.99 | https://www.genecards.org/cgi-bin/carddisp.pl?gene=DCTN1 |
| GNPTG | N-Acetylglucosamine-1-Phosphate Transferase Subunit Gamma | Protein Coding |  | 45 | GC16P001351 | | 0.99 | https://www.genecards.org/cgi-bin/carddisp.pl?gene=GNPTG |
| GYG1 | Glycogenin 1 | Protein Coding |  | 52 | GC03P148991 | | 0.99 | https://www.genecards.org/cgi-bin/carddisp.pl?gene=GYG1 |
| MAT2A | Methionine Adenosyltransferase 2A | Protein Coding |  | 52 | GC02P085538 | | 0.99 | https://www.genecards.org/cgi-bin/carddisp.pl?gene=MAT2A |
| FTO | FTO Alpha-Ketoglutarate Dependent Dioxygenase | Protein Coding |  | 51 | GC16P053737 | | 0.99 | https://www.genecards.org/cgi-bin/carddisp.pl?gene=FTO |
| ATXN1 | Ataxin 1 | Protein Coding |  | 49 | GC06M016299 | | 0.99 | https://www.genecards.org/cgi-bin/carddisp.pl?gene=ATXN1 |
| TRIP10 | Thyroid Hormone Receptor Interactor 10 | Protein Coding |  | 46 | GC19P006737 | | 0.99 | https://www.genecards.org/cgi-bin/carddisp.pl?gene=TRIP10 |
| SNX9 | Sorting Nexin 9 | Protein Coding |  | 45 | GC06P157685 | | 0.99 | https://www.genecards.org/cgi-bin/carddisp.pl?gene=SNX9 |
| PPL | Periplakin | Protein Coding |  | 46 | GC16M004872 | | 0.98 | https://www.genecards.org/cgi-bin/carddisp.pl?gene=PPL |
| OTUB1 | OTU Deubiquitinase, Ubiquitin Aldehyde Binding 1 | Protein Coding |  | 45 | GC11P063985 | | 0.98 | https://www.genecards.org/cgi-bin/carddisp.pl?gene=OTUB1 |
| IL17F | Interleukin 17F | Protein Coding |  | 47 | GC06M052209 | | 0.98 | https://www.genecards.org/cgi-bin/carddisp.pl?gene=IL17F |
| SLC11A1 | Solute Carrier Family 11 Member 1 | Protein Coding |  | 53 | GC02P218382 | | 0.98 | https://www.genecards.org/cgi-bin/carddisp.pl?gene=SLC11A1 |
| CD53 | CD53 Molecule | Protein Coding |  | 46 | GC01P110871 | | 0.98 | https://www.genecards.org/cgi-bin/carddisp.pl?gene=CD53 |
| NFATC1 | Nuclear Factor Of Activated T Cells 1 | Protein Coding |  | 54 | GC18P079395 | | 0.98 | https://www.genecards.org/cgi-bin/carddisp.pl?gene=NFATC1 |
| PRKAA2 | Protein Kinase AMP-Activated Catalytic Subunit Alpha 2 | Protein Coding |  | 56 | GC01P056645 | | 0.98 | https://www.genecards.org/cgi-bin/carddisp.pl?gene=PRKAA2 |
| AKR1A1 | Aldo-Keto Reductase Family 1 Member A1 | Protein Coding |  | 50 | GC01P045550 | | 0.98 | https://www.genecards.org/cgi-bin/carddisp.pl?gene=AKR1A1 |
| SLC7A2 | Solute Carrier Family 7 Member 2 | Protein Coding |  | 48 | GC08P017497 | | 0.97 | https://www.genecards.org/cgi-bin/carddisp.pl?gene=SLC7A2 |
| L3MBTL1 | L3MBTL Histone Methyl-Lysine Binding Protein 1 | Protein Coding |  | 44 | GC20P043507 | | 0.97 | https://www.genecards.org/cgi-bin/carddisp.pl?gene=L3MBTL1 |
| MICB | MHC Class I Polypeptide-Related Sequence B | Protein Coding |  | 48 | GC06P032494 | | 0.96 | https://www.genecards.org/cgi-bin/carddisp.pl?gene=MICB |
| PRMT1 | Protein Arginine Methyltransferase 1 | Protein Coding |  | 54 | GC19P049675 | | 0.96 | https://www.genecards.org/cgi-bin/carddisp.pl?gene=PRMT1 |
| ANGPT1 | Angiopoietin 1 | Protein Coding |  | 51 | GC08M107246 | | 0.96 | https://www.genecards.org/cgi-bin/carddisp.pl?gene=ANGPT1 |
| ACKR1 | Atypical Chemokine Receptor 1 (Duffy Blood Group) | Protein Coding |  | 41 | GC01P159203 | | 0.96 | https://www.genecards.org/cgi-bin/carddisp.pl?gene=ACKR1 |
| HLA-DQA1 | Major Histocompatibility Complex, Class II, DQ Alpha 1 | Protein Coding |  | 48 | GC06P032807 | | 0.95 | https://www.genecards.org/cgi-bin/carddisp.pl?gene=HLA-DQA1 |
| EGLN1 | Egl-9 Family Hypoxia Inducible Factor 1 | Protein Coding |  | 55 | GC01M231363 | | 0.95 | https://www.genecards.org/cgi-bin/carddisp.pl?gene=EGLN1 |
| PTMA | Prothymosin Alpha | Protein Coding |  | 43 | GC02P231707 | | 0.95 | https://www.genecards.org/cgi-bin/carddisp.pl?gene=PTMA |
| PARK7 | Parkinsonism Associated Deglycase | Protein Coding |  | 52 | GC01P007957 | | 0.95 | https://www.genecards.org/cgi-bin/carddisp.pl?gene=PARK7 |
| CLCN2 | Chloride Voltage-Gated Channel 2 | Protein Coding |  | 52 | GC03M184346 | | 0.94 | https://www.genecards.org/cgi-bin/carddisp.pl?gene=CLCN2 |
| OGDH | Oxoglutarate Dehydrogenase | Protein Coding |  | 51 | GC07P044646 | | 0.94 | https://www.genecards.org/cgi-bin/carddisp.pl?gene=OGDH |
| NPHP1 | Nephrocystin 1 | Protein Coding |  | 50 | GC02M110122 | | 0.94 | https://www.genecards.org/cgi-bin/carddisp.pl?gene=NPHP1 |
| MGAT1 | Mannosyl (Alpha-1,3-)-Glycoprotein Beta-1,2-N-Acetylglucosaminyltransferase | Protein Coding |  | 49 | GC05M180790 | | 0.94 | https://www.genecards.org/cgi-bin/carddisp.pl?gene=MGAT1 |
| MICAL1 | Microtubule Associated Monooxygenase, Calponin And LIM Domain Containing 1 | Protein Coding |  | 47 | GC06M109444 | | 0.94 | https://www.genecards.org/cgi-bin/carddisp.pl?gene=MICAL1 |
| LAIR1 | Leukocyte Associated Immunoglobulin Like Receptor 1 | Protein Coding |  | 47 | GC19M054351 | | 0.94 | https://www.genecards.org/cgi-bin/carddisp.pl?gene=LAIR1 |
| FMO2 | Flavin Containing Dimethylaniline Monoxygenase 2 | Protein Coding |  | 46 | GC01P171154 | | 0.94 | https://www.genecards.org/cgi-bin/carddisp.pl?gene=FMO2 |
| FKBP14 | FKBP Prolyl Isomerase 14 | Protein Coding |  | 46 | GC07M030050 | | 0.94 | https://www.genecards.org/cgi-bin/carddisp.pl?gene=FKBP14 |
| WDR45 | WD Repeat Domain 45 | Protein Coding |  | 46 | GC0XM049074 | | 0.94 | https://www.genecards.org/cgi-bin/carddisp.pl?gene=WDR45 |
| PPP1R15B | Protein Phosphatase 1 Regulatory Subunit 15B | Protein Coding |  | 44 | GC01M204372 | | 0.94 | https://www.genecards.org/cgi-bin/carddisp.pl?gene=PPP1R15B |
| ALMS1P1 | ALMS1 Pseudogene 1 | Pseudogene |  | 17 | GC02P073644 | | 0.94 | https://www.genecards.org/cgi-bin/carddisp.pl?gene=ALMS1P1 |
| CRISP3 | Cysteine Rich Secretory Protein 3 | Protein Coding |  | 43 | GC06M049727 | | 0.94 | https://www.genecards.org/cgi-bin/carddisp.pl?gene=CRISP3 |
| CKB | Creatine Kinase B | Protein Coding |  | 52 | GC14M103519 | | 0.94 | https://www.genecards.org/cgi-bin/carddisp.pl?gene=CKB |
| NTN1 | Netrin 1 | Protein Coding |  | 50 | GC17P009021 | | 0.94 | https://www.genecards.org/cgi-bin/carddisp.pl?gene=NTN1 |
| ADAM10 | ADAM Metallopeptidase Domain 10 | Protein Coding |  | 59 | GC15M058588 | | 0.94 | https://www.genecards.org/cgi-bin/carddisp.pl?gene=ADAM10 |
| LTB4R2 | Leukotriene B4 Receptor 2 | Protein Coding |  | 47 | GC14P024633 | | 0.93 | https://www.genecards.org/cgi-bin/carddisp.pl?gene=LTB4R2 |
| CRHR1 | Corticotropin Releasing Hormone Receptor 1 | Protein Coding |  | 51 | GC17P045784 | | 0.93 | https://www.genecards.org/cgi-bin/carddisp.pl?gene=CRHR1 |
| TRPV4 | Transient Receptor Potential Cation Channel Subfamily V Member 4 | Protein Coding |  | 56 | GC12M109783 | | 0.93 | https://www.genecards.org/cgi-bin/carddisp.pl?gene=TRPV4 |
| ADAMTSL1 | ADAMTS Like 1 | Protein Coding |  | 48 | GC09P017906 | | 0.92 | https://www.genecards.org/cgi-bin/carddisp.pl?gene=ADAMTSL1 |
| TONSL | Tonsoku Like, DNA Repair Protein | Protein Coding |  | 41 | GC08M144428 | | 0.92 | https://www.genecards.org/cgi-bin/carddisp.pl?gene=TONSL |
| KPNA2 | Karyopherin Subunit Alpha 2 | Protein Coding |  | 52 | GC17P068035 | | 0.92 | https://www.genecards.org/cgi-bin/carddisp.pl?gene=KPNA2 |
| CD83 | CD83 Molecule | Protein Coding |  | 44 | GC06P014117 | | 0.92 | https://www.genecards.org/cgi-bin/carddisp.pl?gene=CD83 |
| PAX2 | Paired Box 2 | Protein Coding |  | 53 | GC10P100735 | | 0.92 | https://www.genecards.org/cgi-bin/carddisp.pl?gene=PAX2 |
| YWHAQ | Tyrosine 3-Monooxygenase/Tryptophan 5-Monooxygenase Activation Protein Theta | Protein Coding |  | 53 | GC02M009641 | | 0.92 | https://www.genecards.org/cgi-bin/carddisp.pl?gene=YWHAQ |
| ARHGEF2 | Rho/Rac Guanine Nucleotide Exchange Factor 2 | Protein Coding |  | 51 | GC01M155946 | | 0.91 | https://www.genecards.org/cgi-bin/carddisp.pl?gene=ARHGEF2 |
| SPDEF | SAM Pointed Domain Containing ETS Transcription Factor | Protein Coding |  | 45 | GC06M039640 | | 0.91 | https://www.genecards.org/cgi-bin/carddisp.pl?gene=SPDEF |
| DNASE1L3 | Deoxyribonuclease 1 Like 3 | Protein Coding |  | 50 | GC03M058192 | | 0.91 | https://www.genecards.org/cgi-bin/carddisp.pl?gene=DNASE1L3 |
| KRT17 | Keratin 17 | Protein Coding |  | 52 | GC17M041619 | | 0.91 | https://www.genecards.org/cgi-bin/carddisp.pl?gene=KRT17 |
| ISL1 | ISL LIM Homeobox 1 | Protein Coding |  | 51 | GC05P051383 | | 0.9 | https://www.genecards.org/cgi-bin/carddisp.pl?gene=ISL1 |
| PSMB4 | Proteasome Subunit Beta 4 | Protein Coding |  | 51 | GC01P151372 | | 0.9 | https://www.genecards.org/cgi-bin/carddisp.pl?gene=PSMB4 |
| CCBE1 | Collagen And Calcium Binding EGF Domains 1 | Protein Coding |  | 46 | GC18M059430 | | 0.9 | https://www.genecards.org/cgi-bin/carddisp.pl?gene=CCBE1 |
| ELP1 | Elongator Complex Protein 1 | Protein Coding |  | 39 | GC09M108868 | | 0.9 | https://www.genecards.org/cgi-bin/carddisp.pl?gene=ELP1 |
| DLK1 | Delta Like Non-Canonical Notch Ligand 1 | Protein Coding |  | 50 | GC14P103771 | | 0.9 | https://www.genecards.org/cgi-bin/carddisp.pl?gene=DLK1 |
| HSPA14 | Heat Shock Protein Family A (Hsp70) Member 14 | Protein Coding |  | 44 | GC10P014790 | | 0.89 | https://www.genecards.org/cgi-bin/carddisp.pl?gene=HSPA14 |
| WFDC2 | WAP Four-Disulfide Core Domain 2 | Protein Coding |  | 43 | GC20P045469 | | 0.89 | https://www.genecards.org/cgi-bin/carddisp.pl?gene=WFDC2 |
| YWHAG | Tyrosine 3-Monooxygenase/Tryptophan 5-Monooxygenase Activation Protein Gamma | Protein Coding |  | 54 | GC07M076327 | | 0.89 | https://www.genecards.org/cgi-bin/carddisp.pl?gene=YWHAG |
| EPX | Eosinophil Peroxidase | Protein Coding |  | 48 | GC17P058192 | | 0.88 | https://www.genecards.org/cgi-bin/carddisp.pl?gene=EPX |
| THY1 | Thy-1 Cell Surface Antigen | Protein Coding |  | 50 | GC11M119417 | | 0.88 | https://www.genecards.org/cgi-bin/carddisp.pl?gene=THY1 |
| HIST1H1C | Histone Cluster 1 H1 Family Member C | Protein Coding |  | 46 | GC06M026055 | | 0.88 | https://www.genecards.org/cgi-bin/carddisp.pl?gene=HIST1H1C |
| HYOU1 | Hypoxia Up-Regulated 1 | Protein Coding |  | 48 | GC11M119045 | | 0.87 | https://www.genecards.org/cgi-bin/carddisp.pl?gene=HYOU1 |
| CUL2 | Cullin 2 | Protein Coding |  | 47 | GC10M035046 | | 0.87 | https://www.genecards.org/cgi-bin/carddisp.pl?gene=CUL2 |
| AKAP13 | A-Kinase Anchoring Protein 13 | Protein Coding |  | 50 | GC15P085381 | | 0.87 | https://www.genecards.org/cgi-bin/carddisp.pl?gene=AKAP13 |
| CD207 | CD207 Molecule | Protein Coding |  | 45 | GC02M070830 | | 0.87 | https://www.genecards.org/cgi-bin/carddisp.pl?gene=CD207 |
| NCR1 | Natural Cytotoxicity Triggering Receptor 1 | Protein Coding |  | 43 | GC19P055136 | | 0.87 | https://www.genecards.org/cgi-bin/carddisp.pl?gene=NCR1 |
| S100A5 | S100 Calcium Binding Protein A5 | Protein Coding |  | 40 | GC01M153509 | | 0.87 | https://www.genecards.org/cgi-bin/carddisp.pl?gene=S100A5 |
| CTSK | Cathepsin K | Protein Coding |  | 55 | GC01M150768 | | 0.87 | https://www.genecards.org/cgi-bin/carddisp.pl?gene=CTSK |
| TBK1 | TANK Binding Kinase 1 | Protein Coding |  | 56 | GC12P064451 | | 0.87 | https://www.genecards.org/cgi-bin/carddisp.pl?gene=TBK1 |
| CFHR3 | Complement Factor H Related 3 | Protein Coding |  | 43 | GC01P196743 | | 0.86 | https://www.genecards.org/cgi-bin/carddisp.pl?gene=CFHR3 |
| IL18R1 | Interleukin 18 Receptor 1 | Protein Coding |  | 49 | GC02P102345 | | 0.86 | https://www.genecards.org/cgi-bin/carddisp.pl?gene=IL18R1 |
| FUT1 | Fucosyltransferase 1 (H Blood Group) | Protein Coding |  | 47 | GC19M048748 | | 0.86 | https://www.genecards.org/cgi-bin/carddisp.pl?gene=FUT1 |
| UPF1 | UPF1 RNA Helicase And ATPase | Protein Coding |  | 46 | GC19P018831 | | 0.86 | https://www.genecards.org/cgi-bin/carddisp.pl?gene=UPF1 |
| FARSA | Phenylalanyl-TRNA Synthetase Subunit Alpha | Protein Coding |  | 46 | GC19M012922 | | 0.86 | https://www.genecards.org/cgi-bin/carddisp.pl?gene=FARSA |
| SRXN1 | Sulfiredoxin 1 | Protein Coding |  | 40 | GC20M000647 | | 0.86 | https://www.genecards.org/cgi-bin/carddisp.pl?gene=SRXN1 |
| PTPN22 | Protein Tyrosine Phosphatase Non-Receptor Type 22 | Protein Coding |  | 53 | GC01M113813 | | 0.86 | https://www.genecards.org/cgi-bin/carddisp.pl?gene=PTPN22 |
| PSMB9 | Proteasome Subunit Beta 9 | Protein Coding |  | 53 | GC06P032845 | | 0.86 | https://www.genecards.org/cgi-bin/carddisp.pl?gene=PSMB9 |
| ADAMTS3 | ADAM Metallopeptidase With Thrombospondin Type 1 Motif 3 | Protein Coding |  | 48 | GC04M072280 | | 0.86 | https://www.genecards.org/cgi-bin/carddisp.pl?gene=ADAMTS3 |
| ARID2 | AT-Rich Interaction Domain 2 | Protein Coding |  | 47 | GC12P045729 | | 0.86 | https://www.genecards.org/cgi-bin/carddisp.pl?gene=ARID2 |
| HPS6 | HPS6 Biogenesis Of Lysosomal Organelles Complex 2 Subunit 3 | Protein Coding |  | 45 | GC10P102065 | | 0.86 | https://www.genecards.org/cgi-bin/carddisp.pl?gene=HPS6 |
| LIPN | Lipase Family Member N | Protein Coding |  | 43 | GC10P088760 | | 0.86 | https://www.genecards.org/cgi-bin/carddisp.pl?gene=LIPN |
| LBR | Lamin B Receptor | Protein Coding |  | 53 | GC01M225401 | | 0.86 | https://www.genecards.org/cgi-bin/carddisp.pl?gene=LBR |
| ELOC | Elongin C | Protein Coding |  | 38 | GC08M073939 | | 0.85 | https://www.genecards.org/cgi-bin/carddisp.pl?gene=ELOC |
| GBE1 | 1,4-Alpha-Glucan Branching Enzyme 1 | Protein Coding |  | 50 | GC03M081489 | | 0.84 | https://www.genecards.org/cgi-bin/carddisp.pl?gene=GBE1 |
| ACTR2 | Actin Related Protein 2 | Protein Coding |  | 49 | GC02P065227 | | 0.84 | https://www.genecards.org/cgi-bin/carddisp.pl?gene=ACTR2 |
| GBP1 | Guanylate Binding Protein 1 | Protein Coding |  | 47 | GC01M089052 | | 0.84 | https://www.genecards.org/cgi-bin/carddisp.pl?gene=GBP1 |
| SMC4 | Structural Maintenance Of Chromosomes 4 | Protein Coding |  | 47 | GC03P160399 | | 0.84 | https://www.genecards.org/cgi-bin/carddisp.pl?gene=SMC4 |
| STMN2 | Stathmin 2 | Protein Coding |  | 45 | GC08P079610 | | 0.84 | https://www.genecards.org/cgi-bin/carddisp.pl?gene=STMN2 |
| MCM4 | Minichromosome Maintenance Complex Component 4 | Protein Coding |  | 53 | GC08P047965 | | 0.84 | https://www.genecards.org/cgi-bin/carddisp.pl?gene=MCM4 |
| SOS1 | SOS Ras/Rac Guanine Nucleotide Exchange Factor 1 | Protein Coding |  | 54 | GC02M038981 | | 0.84 | https://www.genecards.org/cgi-bin/carddisp.pl?gene=SOS1 |
| CSNK2B | Casein Kinase 2 Beta | Protein Coding |  | 53 | GC06P032503 | | 0.83 | https://www.genecards.org/cgi-bin/carddisp.pl?gene=CSNK2B |
| FGF23 | Fibroblast Growth Factor 23 | Protein Coding |  | 51 | GC12M004347 | | 0.83 | https://www.genecards.org/cgi-bin/carddisp.pl?gene=FGF23 |
| TRPA1 | Transient Receptor Potential Cation Channel Subfamily A Member 1 | Protein Coding |  | 52 | GC08M072019 | | 0.82 | https://www.genecards.org/cgi-bin/carddisp.pl?gene=TRPA1 |
| FCGR3B | Fc Fragment Of IgG Receptor IIIb | Protein Coding |  | 49 | GC01M161623 | | 0.82 | https://www.genecards.org/cgi-bin/carddisp.pl?gene=FCGR3B |
| ESM1 | Endothelial Cell Specific Molecule 1 | Protein Coding |  | 45 | GC05M054977 | | 0.81 | https://www.genecards.org/cgi-bin/carddisp.pl?gene=ESM1 |
| SCN9A | Sodium Voltage-Gated Channel Alpha Subunit 9 | Protein Coding |  | 53 | GC02M166195 | | 0.81 | https://www.genecards.org/cgi-bin/carddisp.pl?gene=SCN9A |
| ID1 | Inhibitor Of DNA Binding 1, HLH Protein | Protein Coding |  | 48 | GC20P031605 | | 0.81 | https://www.genecards.org/cgi-bin/carddisp.pl?gene=ID1 |
| CBL | Cbl Proto-Oncogene | Protein Coding |  | 57 | GC11P119206 | | 0.81 | https://www.genecards.org/cgi-bin/carddisp.pl?gene=CBL |
| DNM1 | Dynamin 1 | Protein Coding |  | 57 | GC09P128203 | | 0.8 | https://www.genecards.org/cgi-bin/carddisp.pl?gene=DNM1 |
| ROCK1 | Rho Associated Coiled-Coil Containing Protein Kinase 1 | Protein Coding |  | 56 | GC18M020946 | | 0.8 | https://www.genecards.org/cgi-bin/carddisp.pl?gene=ROCK1 |
| CSTB | Cystatin B | Protein Coding |  | 52 | GC21M043772 | | 0.8 | https://www.genecards.org/cgi-bin/carddisp.pl?gene=CSTB |
| GP1BA | Glycoprotein Ib Platelet Subunit Alpha | Protein Coding |  | 51 | GC17P004932 | | 0.8 | https://www.genecards.org/cgi-bin/carddisp.pl?gene=GP1BA |
| DNAJC5 | DnaJ Heat Shock Protein Family (Hsp40) Member C5 | Protein Coding |  | 50 | GC20P063895 | | 0.8 | https://www.genecards.org/cgi-bin/carddisp.pl?gene=DNAJC5 |
| AVPR1B | Arginine Vasopressin Receptor 1B | Protein Coding |  | 49 | GC01M206109 | | 0.8 | https://www.genecards.org/cgi-bin/carddisp.pl?gene=AVPR1B |
| GM2A | GM2 Ganglioside Activator | Protein Coding |  | 49 | GC05P151229 | | 0.8 | https://www.genecards.org/cgi-bin/carddisp.pl?gene=GM2A |
| WASF2 | WASP Family Member 2 | Protein Coding |  | 48 | GC01M027404 | | 0.8 | https://www.genecards.org/cgi-bin/carddisp.pl?gene=WASF2 |
| TMSB4X | Thymosin Beta 4 X-Linked | Protein Coding |  | 46 | GC0XP012993 | | 0.8 | https://www.genecards.org/cgi-bin/carddisp.pl?gene=TMSB4X |
| PCOLCE | Procollagen C-Endopeptidase Enhancer | Protein Coding |  | 45 | GC07P100602 | | 0.8 | https://www.genecards.org/cgi-bin/carddisp.pl?gene=PCOLCE |
| REG3A | Regenerating Family Member 3 Alpha | Protein Coding |  | 45 | GC02M079157 | | 0.8 | https://www.genecards.org/cgi-bin/carddisp.pl?gene=REG3A |
| F10 | Coagulation Factor X | Protein Coding |  | 55 | GC13P113122 | | 0.8 | https://www.genecards.org/cgi-bin/carddisp.pl?gene=F10 |
| CX3CR1 | C-X3-C Motif Chemokine Receptor 1 | Protein Coding |  | 51 | GC03M039279 | | 0.79 | https://www.genecards.org/cgi-bin/carddisp.pl?gene=CX3CR1 |
| ING4 | Inhibitor Of Growth Family Member 4 | Protein Coding |  | 42 | GC12M006650 | | 0.79 | https://www.genecards.org/cgi-bin/carddisp.pl?gene=ING4 |
| HTR1A | 5-Hydroxytryptamine Receptor 1A | Protein Coding |  | 54 | GC05M063960 | | 0.79 | https://www.genecards.org/cgi-bin/carddisp.pl?gene=HTR1A |
| ITGA1 | Integrin Subunit Alpha 1 | Protein Coding |  | 47 | GC05P052788 | | 0.79 | https://www.genecards.org/cgi-bin/carddisp.pl?gene=ITGA1 |
| ANKH | ANKH Inorganic Pyrophosphate Transport Regulator | Protein Coding |  | 46 | GC05M014706 | | 0.79 | https://www.genecards.org/cgi-bin/carddisp.pl?gene=ANKH |
| ARG2 | Arginase 2 | Protein Coding |  | 52 | GC14P067619 | | 0.78 | https://www.genecards.org/cgi-bin/carddisp.pl?gene=ARG2 |
| DCN | Decorin | Protein Coding |  | 53 | GC12M091140 | | 0.78 | https://www.genecards.org/cgi-bin/carddisp.pl?gene=DCN |
| HCRT | Hypocretin Neuropeptide Precursor | Protein Coding |  | 48 | GC17M042185 | | 0.78 | https://www.genecards.org/cgi-bin/carddisp.pl?gene=HCRT |
| RASSF6 | Ras Association Domain Family Member 6 | Protein Coding |  | 44 | GC04M073571 | | 0.78 | https://www.genecards.org/cgi-bin/carddisp.pl?gene=RASSF6 |
| GTF3C1 | General Transcription Factor IIIC Subunit 1 | Protein Coding |  | 43 | GC16M027471 | | 0.78 | https://www.genecards.org/cgi-bin/carddisp.pl?gene=GTF3C1 |
| INTS8 | Integrator Complex Subunit 8 | Protein Coding |  | 42 | GC08P094813 | | 0.78 | https://www.genecards.org/cgi-bin/carddisp.pl?gene=INTS8 |
| MLXIPL | MLX Interacting Protein Like | Protein Coding |  | 48 | GC07M073593 | | 0.77 | https://www.genecards.org/cgi-bin/carddisp.pl?gene=MLXIPL |
| EIF2S1 | Eukaryotic Translation Initiation Factor 2 Subunit Alpha | Protein Coding |  | 51 | GC14P067359 | | 0.77 | https://www.genecards.org/cgi-bin/carddisp.pl?gene=EIF2S1 |
| SERPINF2 | Serpin Family F Member 2 | Protein Coding |  | 50 | GC17P001742 | | 0.77 | https://www.genecards.org/cgi-bin/carddisp.pl?gene=SERPINF2 |
| PSEN2 | Presenilin 2 | Protein Coding |  | 56 | GC01P226870 | | 0.77 | https://www.genecards.org/cgi-bin/carddisp.pl?gene=PSEN2 |
| PCSK1 | Proprotein Convertase Subtilisin/Kexin Type 1 | Protein Coding |  | 53 | GC05M096391 | | 0.77 | https://www.genecards.org/cgi-bin/carddisp.pl?gene=PCSK1 |
| SCO2 | SCO Cytochrome C Oxidase Assembly Protein 2 | Protein Coding |  | 50 | GC22M050523 | | 0.77 | https://www.genecards.org/cgi-bin/carddisp.pl?gene=SCO2 |
| PRSS8 | Serine Protease 8 | Protein Coding |  | 50 | GC16M031142 | | 0.77 | https://www.genecards.org/cgi-bin/carddisp.pl?gene=PRSS8 |
| VPS35 | VPS35 Retromer Complex Component | Protein Coding |  | 50 | GC16M046661 | | 0.77 | https://www.genecards.org/cgi-bin/carddisp.pl?gene=VPS35 |
| PSMB3 | Proteasome Subunit Beta 3 | Protein Coding |  | 47 | GC17P038752 | | 0.77 | https://www.genecards.org/cgi-bin/carddisp.pl?gene=PSMB3 |
| TNFAIP6 | TNF Alpha Induced Protein 6 | Protein Coding |  | 46 | GC02P151357 | | 0.77 | https://www.genecards.org/cgi-bin/carddisp.pl?gene=TNFAIP6 |
| ID3 | Inhibitor Of DNA Binding 3, HLH Protein | Protein Coding |  | 48 | GC01M023557 | | 0.76 | https://www.genecards.org/cgi-bin/carddisp.pl?gene=ID3 |
| DNAH8 | Dynein Axonemal Heavy Chain 8 | Protein Coding |  | 41 | GC06P043204 | | 0.76 | https://www.genecards.org/cgi-bin/carddisp.pl?gene=DNAH8 |
| FCGR2B | Fc Fragment Of IgG Receptor IIb | Protein Coding |  | 54 | GC01P161663 | | 0.76 | https://www.genecards.org/cgi-bin/carddisp.pl?gene=FCGR2B |
| SULF2 | Sulfatase 2 | Protein Coding |  | 46 | GC20M047656 | | 0.76 | https://www.genecards.org/cgi-bin/carddisp.pl?gene=SULF2 |
| TAZ | Tafazzin | Protein Coding |  | 50 | GC0XP154411 | | 0.75 | https://www.genecards.org/cgi-bin/carddisp.pl?gene=TAZ |
| RBPJ | Recombination Signal Binding Protein For Immunoglobulin Kappa J Region | Protein Coding |  | 53 | GC04P026165 | | 0.75 | https://www.genecards.org/cgi-bin/carddisp.pl?gene=RBPJ |
| PYGM | Glycogen Phosphorylase, Muscle Associated | Protein Coding |  | 53 | GC11M064763 | | 0.75 | https://www.genecards.org/cgi-bin/carddisp.pl?gene=PYGM |
| ROCK2 | Rho Associated Coiled-Coil Containing Protein Kinase 2 | Protein Coding |  | 53 | GC02M011180 | | 0.75 | https://www.genecards.org/cgi-bin/carddisp.pl?gene=ROCK2 |
| RDH11 | Retinol Dehydrogenase 11 | Protein Coding |  | 51 | GC14M067676 | | 0.75 | https://www.genecards.org/cgi-bin/carddisp.pl?gene=RDH11 |
| ADRB3 | Adrenoceptor Beta 3 | Protein Coding |  | 50 | GC08M037962 | | 0.75 | https://www.genecards.org/cgi-bin/carddisp.pl?gene=ADRB3 |
| FUT2 | Fucosyltransferase 2 | Protein Coding |  | 50 | GC19P048695 | | 0.75 | https://www.genecards.org/cgi-bin/carddisp.pl?gene=FUT2 |
| FUT8 | Fucosyltransferase 8 | Protein Coding |  | 50 | GC14P065411 | | 0.75 | https://www.genecards.org/cgi-bin/carddisp.pl?gene=FUT8 |
| PSMB1 | Proteasome Subunit Beta 1 | Protein Coding |  | 50 | GC06M170535 | | 0.75 | https://www.genecards.org/cgi-bin/carddisp.pl?gene=PSMB1 |
| TUFM | Tu Translation Elongation Factor, Mitochondrial | Protein Coding |  | 50 | GC16M028853 | | 0.75 | https://www.genecards.org/cgi-bin/carddisp.pl?gene=TUFM |
| SECISBP2 | SECIS Binding Protein 2 | Protein Coding |  | 49 | GC09P089318 | | 0.75 | https://www.genecards.org/cgi-bin/carddisp.pl?gene=SECISBP2 |
| SPG7 | SPG7 Matrix AAA Peptidase Subunit, Paraplegin | Protein Coding |  | 49 | GC16P089492 | | 0.75 | https://www.genecards.org/cgi-bin/carddisp.pl?gene=SPG7 |
| HSPH1 | Heat Shock Protein Family H (Hsp110) Member 1 | Protein Coding |  | 48 | GC13M031134 | | 0.75 | https://www.genecards.org/cgi-bin/carddisp.pl?gene=HSPH1 |
| EHD1 | EH Domain Containing 1 | Protein Coding |  | 48 | GC11M064870 | | 0.75 | https://www.genecards.org/cgi-bin/carddisp.pl?gene=EHD1 |
| NRL | Neural Retina Leucine Zipper | Protein Coding |  | 48 | GC14M024078 | | 0.75 | https://www.genecards.org/cgi-bin/carddisp.pl?gene=NRL |
| LMO2 | LIM Domain Only 2 | Protein Coding |  | 48 | GC11M033880 | | 0.75 | https://www.genecards.org/cgi-bin/carddisp.pl?gene=LMO2 |
| VAMP7 | Vesicle Associated Membrane Protein 7 | Protein Coding |  | 48 | GC0XP155881 | | 0.75 | https://www.genecards.org/cgi-bin/carddisp.pl?gene=VAMP7 |
| TGM5 | Transglutaminase 5 | Protein Coding |  | 48 | GC15M043234 | | 0.75 | https://www.genecards.org/cgi-bin/carddisp.pl?gene=TGM5 |
| TIMM8A | Translocase Of Inner Mitochondrial Membrane 8A | Protein Coding |  | 48 | GC0XM101345 | | 0.75 | https://www.genecards.org/cgi-bin/carddisp.pl?gene=TIMM8A |
| PIGT | Phosphatidylinositol Glycan Anchor Biosynthesis Class T | Protein Coding |  | 47 | GC20P045416 | | 0.75 | https://www.genecards.org/cgi-bin/carddisp.pl?gene=PIGT |
| PITX3 | Paired Like Homeodomain 3 | Protein Coding |  | 46 | GC10M102230 | | 0.75 | https://www.genecards.org/cgi-bin/carddisp.pl?gene=PITX3 |
| HAS1 | Hyaluronan Synthase 1 | Protein Coding |  | 45 | GC19M051714 | | 0.75 | https://www.genecards.org/cgi-bin/carddisp.pl?gene=HAS1 |
| HBD | Hemoglobin Subunit Delta | Protein Coding |  | 45 | GC11M005232 | | 0.75 | https://www.genecards.org/cgi-bin/carddisp.pl?gene=HBD |
| SPG11 | SPG11 Vesicle Trafficking Associated, Spatacsin | Protein Coding |  | 45 | GC15M044562 | | 0.75 | https://www.genecards.org/cgi-bin/carddisp.pl?gene=SPG11 |
| SNRPD1 | Small Nuclear Ribonucleoprotein D1 Polypeptide | Protein Coding |  | 45 | GC18P021612 | | 0.75 | https://www.genecards.org/cgi-bin/carddisp.pl?gene=SNRPD1 |
| NME5 | NME/NM23 Family Member 5 | Protein Coding |  | 44 | GC05M138115 | | 0.75 | https://www.genecards.org/cgi-bin/carddisp.pl?gene=NME5 |
| SRRT | Serrate, RNA Effector Molecule | Protein Coding |  | 43 | GC07P100875 | | 0.75 | https://www.genecards.org/cgi-bin/carddisp.pl?gene=SRRT |
| TRMT10C | TRNA Methyltransferase 10C, Mitochondrial RNase P Subunit | Protein Coding |  | 42 | GC03P101561 | | 0.75 | https://www.genecards.org/cgi-bin/carddisp.pl?gene=TRMT10C |
| TPPP3 | Tubulin Polymerization Promoting Protein Family Member 3 | Protein Coding |  | 42 | GC16M067423 | | 0.75 | https://www.genecards.org/cgi-bin/carddisp.pl?gene=TPPP3 |
| WASHC1 | WASH Complex Subunit 1 | Protein Coding |  | 28 | GC09M000016 | | 0.75 | https://www.genecards.org/cgi-bin/carddisp.pl?gene=WASHC1 |
| GLRA1 | Glycine Receptor Alpha 1 | Protein Coding |  | 52 | GC05M151799 | | 0.75 | https://www.genecards.org/cgi-bin/carddisp.pl?gene=GLRA1 |
| CFHR4 | Complement Factor H Related 4 | Protein Coding |  | 41 | GC01P196857 | | 0.74 | https://www.genecards.org/cgi-bin/carddisp.pl?gene=CFHR4 |
| RAB27A | RAB27A, Member RAS Oncogene Family | Protein Coding |  | 54 | GC15M055202 | | 0.74 | https://www.genecards.org/cgi-bin/carddisp.pl?gene=RAB27A |
| MAOA | Monoamine Oxidase A | Protein Coding |  | 55 | GC0XP043654 | | 0.74 | https://www.genecards.org/cgi-bin/carddisp.pl?gene=MAOA |
| PPP6R2 | Protein Phosphatase 6 Regulatory Subunit 2 | Protein Coding |  | 40 | GC22P050343 | | 0.74 | https://www.genecards.org/cgi-bin/carddisp.pl?gene=PPP6R2 |
| CCRL2 | C-C Motif Chemokine Receptor Like 2 | Protein Coding |  | 45 | GC03P046448 | | 0.73 | https://www.genecards.org/cgi-bin/carddisp.pl?gene=CCRL2 |
| INSR | Insulin Receptor | Protein Coding |  | 60 | GC19M007112 | | 0.73 | https://www.genecards.org/cgi-bin/carddisp.pl?gene=INSR |
| F11 | Coagulation Factor XI | Protein Coding |  | 51 | GC04P186265 | | 0.72 | https://www.genecards.org/cgi-bin/carddisp.pl?gene=F11 |
| FABP1 | Fatty Acid Binding Protein 1 | Protein Coding |  | 50 | GC02M088122 | | 0.72 | https://www.genecards.org/cgi-bin/carddisp.pl?gene=FABP1 |
| DAXX | Death Domain Associated Protein | Protein Coding |  | 50 | GC06M033318 | | 0.72 | https://www.genecards.org/cgi-bin/carddisp.pl?gene=DAXX |
| ACE2 | Angiotensin I Converting Enzyme 2 | Protein Coding |  | 55 | GC0XM015494 | | 0.72 | https://www.genecards.org/cgi-bin/carddisp.pl?gene=ACE2 |
| IGKC | Immunoglobulin Kappa Constant | Protein Coding |  | 37 | GC02M088975 | | 0.71 | https://www.genecards.org/cgi-bin/carddisp.pl?gene=IGKC |
| GJB6 | Gap Junction Protein Beta 6 | Protein Coding |  | 51 | GC13M020221 | | 0.7 | https://www.genecards.org/cgi-bin/carddisp.pl?gene=GJB6 |
| PLA2G7 | Phospholipase A2 Group VII | Protein Coding |  | 56 | GC06M046704 | | 0.7 | https://www.genecards.org/cgi-bin/carddisp.pl?gene=PLA2G7 |
| CD63 | CD63 Molecule | Protein Coding |  | 48 | GC12M055725 | | 0.7 | https://www.genecards.org/cgi-bin/carddisp.pl?gene=CD63 |
| ITGB8 | Integrin Subunit Beta 8 | Protein Coding |  | 47 | GC07P020370 | | 0.7 | https://www.genecards.org/cgi-bin/carddisp.pl?gene=ITGB8 |
| SNAP25 | Synaptosome Associated Protein 25 | Protein Coding |  | 56 | GC20P010218 | | 0.7 | https://www.genecards.org/cgi-bin/carddisp.pl?gene=SNAP25 |
| PRDX1 | Peroxiredoxin 1 | Protein Coding |  | 56 | GC01M045511 | | 0.69 | https://www.genecards.org/cgi-bin/carddisp.pl?gene=PRDX1 |
| MAP3K5 | Mitogen-Activated Protein Kinase Kinase Kinase 5 | Protein Coding |  | 54 | GC06M136500 | | 0.69 | https://www.genecards.org/cgi-bin/carddisp.pl?gene=MAP3K5 |
| ANXA4 | Annexin A4 | Protein Coding |  | 50 | GC02P069705 | | 0.68 | https://www.genecards.org/cgi-bin/carddisp.pl?gene=ANXA4 |
| MC2R | Melanocortin 2 Receptor | Protein Coding |  | 54 | GC18M015731 | | 0.68 | https://www.genecards.org/cgi-bin/carddisp.pl?gene=MC2R |
| PACSIN1 | Protein Kinase C And Casein Kinase Substrate In Neurons 1 | Protein Coding |  | 46 | GC06P043094 | | 0.68 | https://www.genecards.org/cgi-bin/carddisp.pl?gene=PACSIN1 |
| KCNJ5 | Potassium Voltage-Gated Channel Subfamily J Member 5 | Protein Coding |  | 54 | GC11P128891 | | 0.67 | https://www.genecards.org/cgi-bin/carddisp.pl?gene=KCNJ5 |
| TNFRSF14 | TNF Receptor Superfamily Member 14 | Protein Coding |  | 50 | GC01P002555 | | 0.67 | https://www.genecards.org/cgi-bin/carddisp.pl?gene=TNFRSF14 |
| LSM4 | LSM4 Homolog, U6 Small Nuclear RNA And MRNA Degradation Associated | Protein Coding |  | 45 | GC19M018306 | | 0.67 | https://www.genecards.org/cgi-bin/carddisp.pl?gene=LSM4 |
| CLPX | Caseinolytic Mitochondrial Matrix Peptidase Chaperone Subunit | Protein Coding |  | 45 | GC15M065148 | | 0.67 | https://www.genecards.org/cgi-bin/carddisp.pl?gene=CLPX |
| HUWE1 | HECT, UBA And WWE Domain Containing E3 Ubiquitin Protein Ligase 1 | Protein Coding |  | 51 | GC0XM053532 | | 0.67 | https://www.genecards.org/cgi-bin/carddisp.pl?gene=HUWE1 |
| TSPO | Translocator Protein | Protein Coding |  | 48 | GC22P043151 | | 0.67 | https://www.genecards.org/cgi-bin/carddisp.pl?gene=TSPO |
| CLIC1 | Chloride Intracellular Channel 1 | Protein Coding |  | 48 | GC06M032146 | | 0.67 | https://www.genecards.org/cgi-bin/carddisp.pl?gene=CLIC1 |
| USP8 | Ubiquitin Specific Peptidase 8 | Protein Coding |  | 54 | GC15P050424 | | 0.67 | https://www.genecards.org/cgi-bin/carddisp.pl?gene=USP8 |
| PINK1 | PTEN Induced Kinase 1 | Protein Coding |  | 54 | GC01P020634 | | 0.67 | https://www.genecards.org/cgi-bin/carddisp.pl?gene=PINK1 |
| FERMT3 | Fermitin Family Member 3 | Protein Coding |  | 49 | GC11P064206 | | 0.66 | https://www.genecards.org/cgi-bin/carddisp.pl?gene=FERMT3 |
| USP10 | Ubiquitin Specific Peptidase 10 | Protein Coding |  | 51 | GC16P084734 | | 0.66 | https://www.genecards.org/cgi-bin/carddisp.pl?gene=USP10 |
| PIWIL4 | Piwi Like RNA-Mediated Gene Silencing 4 | Protein Coding |  | 43 | GC11P094543 | | 0.66 | https://www.genecards.org/cgi-bin/carddisp.pl?gene=PIWIL4 |
| MARS | Methionyl-TRNA Synthetase | Protein Coding |  | 54 | GC12P057475 | | 0.65 | https://www.genecards.org/cgi-bin/carddisp.pl?gene=MARS |
| HAS2 | Hyaluronan Synthase 2 | Protein Coding |  | 45 | GC08M121594 | | 0.63 | https://www.genecards.org/cgi-bin/carddisp.pl?gene=HAS2 |
| NR0B1 | Nuclear Receptor Subfamily 0 Group B Member 1 | Protein Coding |  | 52 | GC0XM030322 | | 0.63 | https://www.genecards.org/cgi-bin/carddisp.pl?gene=NR0B1 |
| DGUOK | Deoxyguanosine Kinase | Protein Coding |  | 51 | GC02P073926 | | 0.63 | https://www.genecards.org/cgi-bin/carddisp.pl?gene=DGUOK |
| TRIM25 | Tripartite Motif Containing 25 | Protein Coding |  | 51 | GC17M056836 | | 0.63 | https://www.genecards.org/cgi-bin/carddisp.pl?gene=TRIM25 |
| ITIH4 | Inter-Alpha-Trypsin Inhibitor Heavy Chain 4 | Protein Coding |  | 48 | GC03M052812 | | 0.63 | https://www.genecards.org/cgi-bin/carddisp.pl?gene=ITIH4 |
| KLK5 | Kallikrein Related Peptidase 5 | Protein Coding |  | 48 | GC19M050943 | | 0.63 | https://www.genecards.org/cgi-bin/carddisp.pl?gene=KLK5 |
| DPYSL5 | Dihydropyrimidinase Like 5 | Protein Coding |  | 43 | GC02P026847 | | 0.63 | https://www.genecards.org/cgi-bin/carddisp.pl?gene=DPYSL5 |
| AMOTL2 | Angiomotin Like 2 | Protein Coding |  | 42 | GC03M134355 | | 0.63 | https://www.genecards.org/cgi-bin/carddisp.pl?gene=AMOTL2 |
| CTC1 | CST Telomere Replication Complex Component 1 | Protein Coding |  | 41 | GC17M008657 | | 0.63 | https://www.genecards.org/cgi-bin/carddisp.pl?gene=CTC1 |
| FBLN5 | Fibulin 5 | Protein Coding |  | 51 | GC14M091869 | | 0.63 | https://www.genecards.org/cgi-bin/carddisp.pl?gene=FBLN5 |
| VDAC2 | Voltage Dependent Anion Channel 2 | Protein Coding |  | 48 | GC10P075210 | | 0.62 | https://www.genecards.org/cgi-bin/carddisp.pl?gene=VDAC2 |
| BRD2 | Bromodomain Containing 2 | Protein Coding |  | 51 | GC06P032958 | | 0.62 | https://www.genecards.org/cgi-bin/carddisp.pl?gene=BRD2 |
| SRSF6 | Serine And Arginine Rich Splicing Factor 6 | Protein Coding |  | 45 | GC20P043457 | | 0.62 | https://www.genecards.org/cgi-bin/carddisp.pl?gene=SRSF6 |
| HMOX2 | Heme Oxygenase 2 | Protein Coding |  | 54 | GC16P004474 | | 0.61 | https://www.genecards.org/cgi-bin/carddisp.pl?gene=HMOX2 |
| GRK2 | G Protein-Coupled Receptor Kinase 2 | Protein Coding |  | 42 | GC11P067266 | | 0.61 | https://www.genecards.org/cgi-bin/carddisp.pl?gene=GRK2 |
| PPIA | Peptidylprolyl Isomerase A | Protein Coding |  | 53 | GC07P044811 | | 0.61 | https://www.genecards.org/cgi-bin/carddisp.pl?gene=PPIA |
| ISG15 | ISG15 Ubiquitin Like Modifier | Protein Coding |  | 54 | GC01P001001 | | 0.61 | https://www.genecards.org/cgi-bin/carddisp.pl?gene=ISG15 |
| OPLAH | 5-Oxoprolinase, ATP-Hydrolysing | Protein Coding |  | 47 | GC08M144051 | | 0.6 | https://www.genecards.org/cgi-bin/carddisp.pl?gene=OPLAH |
| NAGLU | N-Acetyl-Alpha-Glucosaminidase | Protein Coding |  | 50 | GC17P042535 | | 0.59 | https://www.genecards.org/cgi-bin/carddisp.pl?gene=NAGLU |
| RPL10 | Ribosomal Protein L10 | Protein Coding |  | 52 | GC0XP154389 | | 0.59 | https://www.genecards.org/cgi-bin/carddisp.pl?gene=RPL10 |
| ST3GAL4 | ST3 Beta-Galactoside Alpha-2,3-Sialyltransferase 4 | Protein Coding |  | 48 | GC11P126355 | | 0.59 | https://www.genecards.org/cgi-bin/carddisp.pl?gene=ST3GAL4 |
| ACVR1 | Activin A Receptor Type 1 | Protein Coding |  | 57 | GC02M157736 | | 0.59 | https://www.genecards.org/cgi-bin/carddisp.pl?gene=ACVR1 |
| UFD1 | Ubiquitin Recognition Factor In ER Associated Degradation 1 | Protein Coding |  | 40 | GC22M019450 | | 0.59 | https://www.genecards.org/cgi-bin/carddisp.pl?gene=UFD1 |
| FAM20B | FAM20B Glycosaminoglycan Xylosylkinase | Protein Coding |  | 42 | GC01P179025 | | 0.59 | https://www.genecards.org/cgi-bin/carddisp.pl?gene=FAM20B |
| JUP | Junction Plakoglobin | Protein Coding |  | 53 | GC17M041754 | | 0.59 | https://www.genecards.org/cgi-bin/carddisp.pl?gene=JUP |
| CDK20 | Cyclin Dependent Kinase 20 | Protein Coding |  | 46 | GC09M087966 | | 0.58 | https://www.genecards.org/cgi-bin/carddisp.pl?gene=CDK20 |
| TSPAN32 | Tetraspanin 32 | Protein Coding |  | 42 | GC11P002302 | | 0.58 | https://www.genecards.org/cgi-bin/carddisp.pl?gene=TSPAN32 |
| RXRB | Retinoid X Receptor Beta | Protein Coding |  | 54 | GC06M033193 | | 0.58 | https://www.genecards.org/cgi-bin/carddisp.pl?gene=RXRB |
| NCR3 | Natural Cytotoxicity Triggering Receptor 3 | Protein Coding |  | 45 | GC06M031588 | | 0.58 | https://www.genecards.org/cgi-bin/carddisp.pl?gene=NCR3 |
| LTB | Lymphotoxin Beta | Protein Coding |  | 45 | GC06M032132 | | 0.58 | https://www.genecards.org/cgi-bin/carddisp.pl?gene=LTB |
| TRIM39 | Tripartite Motif Containing 39 | Protein Coding |  | 43 | GC06P032443 | | 0.58 | https://www.genecards.org/cgi-bin/carddisp.pl?gene=TRIM39 |
| MUC21 | Mucin 21, Cell Surface Associated | Protein Coding |  | 37 | GC06P032483 | | 0.58 | https://www.genecards.org/cgi-bin/carddisp.pl?gene=MUC21 |
| TRMT9B | TRNA Methyltransferase 9B (Putative) | Protein Coding |  | 30 | GC08P012945 | | 0.58 | https://www.genecards.org/cgi-bin/carddisp.pl?gene=TRMT9B |
| SRY | Sex Determining Region Y | Protein Coding |  | 41 | GC0YM002698 | | 0.57 | https://www.genecards.org/cgi-bin/carddisp.pl?gene=SRY |
| GLB1 | Galactosidase Beta 1 | Protein Coding |  | 54 | GC03M033013 | | 0.57 | https://www.genecards.org/cgi-bin/carddisp.pl?gene=GLB1 |
| ABCA12 | ATP Binding Cassette Subfamily A Member 12 | Protein Coding |  | 50 | GC02M214931 | | 0.57 | https://www.genecards.org/cgi-bin/carddisp.pl?gene=ABCA12 |
| PSMD12 | Proteasome 26S Subunit, Non-ATPase 12 | Protein Coding |  | 48 | GC17M067337 | | 0.57 | https://www.genecards.org/cgi-bin/carddisp.pl?gene=PSMD12 |
| FLVCR1 | Feline Leukemia Virus Subgroup C Cellular Receptor 1 | Protein Coding |  | 46 | GC01P212858 | | 0.57 | https://www.genecards.org/cgi-bin/carddisp.pl?gene=FLVCR1 |
| TNFSF9 | TNF Superfamily Member 9 | Protein Coding |  | 43 | GC19P006531 | | 0.57 | https://www.genecards.org/cgi-bin/carddisp.pl?gene=TNFSF9 |
| CCL26 | C-C Motif Chemokine Ligand 26 | Protein Coding |  | 43 | GC07M075769 | | 0.57 | https://www.genecards.org/cgi-bin/carddisp.pl?gene=CCL26 |
| KLK11 | Kallikrein Related Peptidase 11 | Protein Coding |  | 45 | GC19M051023 | | 0.57 | https://www.genecards.org/cgi-bin/carddisp.pl?gene=KLK11 |
| CYP2C19 | Cytochrome P450 Family 2 Subfamily C Member 19 | Protein Coding |  | 54 | GC10P094762 | | 0.57 | https://www.genecards.org/cgi-bin/carddisp.pl?gene=CYP2C19 |
| PRELP | Proline And Arginine Rich End Leucine Rich Repeat Protein | Protein Coding |  | 45 | GC01P203444 | | 0.56 | https://www.genecards.org/cgi-bin/carddisp.pl?gene=PRELP |
| IGHA1 | Immunoglobulin Heavy Constant Alpha 1 | Protein Coding |  | 32 | GC14M105768 | | 0.56 | https://www.genecards.org/cgi-bin/carddisp.pl?gene=IGHA1 |
| TPI1 | Triosephosphate Isomerase 1 | Protein Coding |  | 54 | GC12P006910 | | 0.55 | https://www.genecards.org/cgi-bin/carddisp.pl?gene=TPI1 |
| CD151 | CD151 Molecule (Raph Blood Group) | Protein Coding |  | 51 | GC11P000861 | | 0.55 | https://www.genecards.org/cgi-bin/carddisp.pl?gene=CD151 |
| ABCG1 | ATP Binding Cassette Subfamily G Member 1 | Protein Coding |  | 50 | GC21P042199 | | 0.55 | https://www.genecards.org/cgi-bin/carddisp.pl?gene=ABCG1 |
| C3AR1 | Complement C3a Receptor 1 | Protein Coding |  | 49 | GC12M008058 | | 0.55 | https://www.genecards.org/cgi-bin/carddisp.pl?gene=C3AR1 |
| GABPA | GA Binding Protein Transcription Factor Subunit Alpha | Protein Coding |  | 45 | GC21P025734 | | 0.55 | https://www.genecards.org/cgi-bin/carddisp.pl?gene=GABPA |
| SOX9 | SRY-Box 9 | Protein Coding |  | 53 | GC17P072121 | | 0.54 | https://www.genecards.org/cgi-bin/carddisp.pl?gene=SOX9 |
| ASGR2 | Asialoglycoprotein Receptor 2 | Protein Coding |  | 46 | GC17M007101 | | 0.54 | https://www.genecards.org/cgi-bin/carddisp.pl?gene=ASGR2 |
| IREB2 | Iron Responsive Element Binding Protein 2 | Protein Coding |  | 48 | GC15P078437 | | 0.54 | https://www.genecards.org/cgi-bin/carddisp.pl?gene=IREB2 |
| TRIM26 | Tripartite Motif Containing 26 | Protein Coding |  | 43 | GC06M030184 | | 0.54 | https://www.genecards.org/cgi-bin/carddisp.pl?gene=TRIM26 |
| ARL3 | ADP Ribosylation Factor Like GTPase 3 | Protein Coding |  | 51 | GC10M102673 | | 0.53 | https://www.genecards.org/cgi-bin/carddisp.pl?gene=ARL3 |
| MGP | Matrix Gla Protein | Protein Coding |  | 48 | GC12M014881 | | 0.53 | https://www.genecards.org/cgi-bin/carddisp.pl?gene=MGP |
| INPPL1 | Inositol Polyphosphate Phosphatase Like 1 | Protein Coding |  | 55 | GC11P072223 | | 0.53 | https://www.genecards.org/cgi-bin/carddisp.pl?gene=INPPL1 |
| IFNGR2 | Interferon Gamma Receptor 2 | Protein Coding |  | 50 | GC21P033402 | | 0.53 | https://www.genecards.org/cgi-bin/carddisp.pl?gene=IFNGR2 |
| MBTPS2 | Membrane Bound Transcription Factor Peptidase, Site 2 | Protein Coding |  | 49 | GC0XP021839 | | 0.53 | https://www.genecards.org/cgi-bin/carddisp.pl?gene=MBTPS2 |
| BSCL2 | BSCL2 Lipid Droplet Biogenesis Associated, Seipin | Protein Coding |  | 48 | GC11M062983 | | 0.53 | https://www.genecards.org/cgi-bin/carddisp.pl?gene=BSCL2 |
| SEC61A1 | SEC61 Translocon Alpha 1 Subunit | Protein Coding |  | 48 | GC03P128051 | | 0.53 | https://www.genecards.org/cgi-bin/carddisp.pl?gene=SEC61A1 |
| BAZ1B | Bromodomain Adjacent To Zinc Finger Domain 1B | Protein Coding |  | 47 | GC07M073440 | | 0.53 | https://www.genecards.org/cgi-bin/carddisp.pl?gene=BAZ1B |
| BPTF | Bromodomain PHD Finger Transcription Factor | Protein Coding |  | 46 | GC17P067825 | | 0.53 | https://www.genecards.org/cgi-bin/carddisp.pl?gene=BPTF |
| FBXL4 | F-Box And Leucine Rich Repeat Protein 4 | Protein Coding |  | 46 | GC06M098868 | | 0.53 | https://www.genecards.org/cgi-bin/carddisp.pl?gene=FBXL4 |
| FANCB | FA Complementation Group B | Protein Coding |  | 45 | GC0XM014796 | | 0.53 | https://www.genecards.org/cgi-bin/carddisp.pl?gene=FANCB |
| PRDM12 | PR/SET Domain 12 | Protein Coding |  | 43 | GC09P130664 | | 0.53 | https://www.genecards.org/cgi-bin/carddisp.pl?gene=PRDM12 |
| ESS2 | Ess-2 Splicing Factor Homolog | Protein Coding |  | 32 | GC22M019130 | | 0.53 | https://www.genecards.org/cgi-bin/carddisp.pl?gene=ESS2 |
| TNFAIP3 | TNF Alpha Induced Protein 3 | Protein Coding |  | 54 | GC06P137866 | | 0.53 | https://www.genecards.org/cgi-bin/carddisp.pl?gene=TNFAIP3 |
| NSMCE1 | NSE1 Homolog, SMC5-SMC6 Complex Component | Protein Coding |  | 40 | GC16M027236 | | 0.53 | https://www.genecards.org/cgi-bin/carddisp.pl?gene=NSMCE1 |
| CCN4 | Cellular Communication Network Factor 4 | Protein Coding |  | 36 | GC08P133192 | | 0.53 | https://www.genecards.org/cgi-bin/carddisp.pl?gene=CCN4 |
| APOC1 | Apolipoprotein C1 | Protein Coding |  | 46 | GC19P044914 | | 0.52 | https://www.genecards.org/cgi-bin/carddisp.pl?gene=APOC1 |
| ABCB11 | ATP Binding Cassette Subfamily B Member 11 | Protein Coding |  | 53 | GC02M168922 | | 0.52 | https://www.genecards.org/cgi-bin/carddisp.pl?gene=ABCB11 |
| RPS18 | Ribosomal Protein S18 | Protein Coding |  | 45 | GC06P033323 | | 0.51 | https://www.genecards.org/cgi-bin/carddisp.pl?gene=RPS18 |
| DUSP13 | Dual Specificity Phosphatase 13 | Protein Coding |  | 43 | GC10M075094 | | 0.51 | https://www.genecards.org/cgi-bin/carddisp.pl?gene=DUSP13 |
| ARPC1B | Actin Related Protein 2/3 Complex Subunit 1B | Protein Coding |  | 49 | GC07P099374 | | 0.51 | https://www.genecards.org/cgi-bin/carddisp.pl?gene=ARPC1B |
| RFC2 | Replication Factor C Subunit 2 | Protein Coding |  | 50 | GC07M074231 | | 0.5 | https://www.genecards.org/cgi-bin/carddisp.pl?gene=RFC2 |
| PPP1R10 | Protein Phosphatase 1 Regulatory Subunit 10 | Protein Coding |  | 45 | GC06M030568 | | 0.5 | https://www.genecards.org/cgi-bin/carddisp.pl?gene=PPP1R10 |
| AHSP | Alpha Hemoglobin Stabilizing Protein | Protein Coding |  | 42 | GC16P031540 | | 0.5 | https://www.genecards.org/cgi-bin/carddisp.pl?gene=AHSP |
| HYAL2 | Hyaluronidase 2 | Protein Coding |  | 48 | GC03M050317 | | 0.5 | https://www.genecards.org/cgi-bin/carddisp.pl?gene=HYAL2 |
| GC06M032189 |  | RNA Gene |  | 4 | GC06M032189 | | 0.48 | https://www.genecards.org/cgi-bin/carddisp.pl?gene=GC06M032189 |
| GK | Glycerol Kinase | Protein Coding |  | 53 | GC0XP030671 | | 0.48 | https://www.genecards.org/cgi-bin/carddisp.pl?gene=GK |
| GATA6 | GATA Binding Protein 6 | Protein Coding |  | 53 | GC18P022169 | | 0.48 | https://www.genecards.org/cgi-bin/carddisp.pl?gene=GATA6 |
| TRAF1 | TNF Receptor Associated Factor 1 | Protein Coding |  | 49 | GC09M120902 | | 0.48 | https://www.genecards.org/cgi-bin/carddisp.pl?gene=TRAF1 |
| EDAR | Ectodysplasin A Receptor | Protein Coding |  | 48 | GC02M108894 | | 0.48 | https://www.genecards.org/cgi-bin/carddisp.pl?gene=EDAR |
| DPEP1 | Dipeptidase 1 | Protein Coding |  | 48 | GC16P089613 | | 0.48 | https://www.genecards.org/cgi-bin/carddisp.pl?gene=DPEP1 |
| CD180 | CD180 Molecule | Protein Coding |  | 44 | GC05M067181 | | 0.48 | https://www.genecards.org/cgi-bin/carddisp.pl?gene=CD180 |
| EXTL3 | Exostosin Like Glycosyltransferase 3 | Protein Coding |  | 51 | GC08P028615 | | 0.48 | https://www.genecards.org/cgi-bin/carddisp.pl?gene=EXTL3 |
| BTD | Biotinidase | Protein Coding |  | 50 | GC03P015621 | | 0.48 | https://www.genecards.org/cgi-bin/carddisp.pl?gene=BTD |
| DPAGT1 | Dolichyl-Phosphate N-Acetylglucosaminephosphotransferase 1 | Protein Coding |  | 51 | GC11M119096 | | 0.47 | https://www.genecards.org/cgi-bin/carddisp.pl?gene=DPAGT1 |
| CGB7 | Chorionic Gonadotropin Subunit Beta 7 | Protein Coding |  | 35 | GC19M049054 | | 0.47 | https://www.genecards.org/cgi-bin/carddisp.pl?gene=CGB7 |
| F2R | Coagulation Factor II Thrombin Receptor | Protein Coding |  | 53 | GC05P076716 | | 0.46 | https://www.genecards.org/cgi-bin/carddisp.pl?gene=F2R |
| PYCARD | PYD And CARD Domain Containing | Protein Coding |  | 49 | GC16M031212 | | 0.46 | https://www.genecards.org/cgi-bin/carddisp.pl?gene=PYCARD |
| MYO18A | Myosin XVIIIA | Protein Coding |  | 42 | GC17M029392 | | 0.46 | https://www.genecards.org/cgi-bin/carddisp.pl?gene=MYO18A |
| CD247 | CD247 Molecule | Protein Coding |  | 55 | GC01M167399 | | 0.46 | https://www.genecards.org/cgi-bin/carddisp.pl?gene=CD247 |
| CD46 | CD46 Molecule | Protein Coding |  | 53 | GC01P207752 | | 0.46 | https://www.genecards.org/cgi-bin/carddisp.pl?gene=CD46 |
| PTGDS | Prostaglandin D2 Synthase | Protein Coding |  | 51 | GC09P136977 | | 0.46 | https://www.genecards.org/cgi-bin/carddisp.pl?gene=PTGDS |
| NFS1 | NFS1 Cysteine Desulfurase | Protein Coding |  | 51 | GC20M035668 | | 0.46 | https://www.genecards.org/cgi-bin/carddisp.pl?gene=NFS1 |
| ADD1 | Adducin 1 | Protein Coding |  | 50 | GC04P002859 | | 0.46 | https://www.genecards.org/cgi-bin/carddisp.pl?gene=ADD1 |
| TNFRSF18 | TNF Receptor Superfamily Member 18 | Protein Coding |  | 49 | GC01M001203 | | 0.46 | https://www.genecards.org/cgi-bin/carddisp.pl?gene=TNFRSF18 |
| MBD2 | Methyl-CpG Binding Domain Protein 2 | Protein Coding |  | 46 | GC18M054151 | | 0.46 | https://www.genecards.org/cgi-bin/carddisp.pl?gene=MBD2 |
| APCS | Amyloid P Component, Serum | Protein Coding |  | 46 | GC01P159557 | | 0.46 | https://www.genecards.org/cgi-bin/carddisp.pl?gene=APCS |
| RNPC3 | RNA Binding Region (RNP1, RRM) Containing 3 | Protein Coding |  | 41 | GC01P103525 | | 0.46 | https://www.genecards.org/cgi-bin/carddisp.pl?gene=RNPC3 |
| KPNA4 | Karyopherin Subunit Alpha 4 | Protein Coding |  | 47 | GC03M160494 | | 0.45 | https://www.genecards.org/cgi-bin/carddisp.pl?gene=KPNA4 |
| TPP2 | Tripeptidyl Peptidase 2 | Protein Coding |  | 47 | GC13P102596 | | 0.45 | https://www.genecards.org/cgi-bin/carddisp.pl?gene=TPP2 |
| IER3IP1 | Immediate Early Response 3 Interacting Protein 1 | Protein Coding |  | 45 | GC18M047152 | | 0.45 | https://www.genecards.org/cgi-bin/carddisp.pl?gene=IER3IP1 |
| MYO9A | Myosin IXA | Protein Coding |  | 45 | GC15M071822 | | 0.45 | https://www.genecards.org/cgi-bin/carddisp.pl?gene=MYO9A |
| PRKAG2 | Protein Kinase AMP-Activated Non-Catalytic Subunit Gamma 2 | Protein Coding |  | 56 | GC07M151556 | | 0.45 | https://www.genecards.org/cgi-bin/carddisp.pl?gene=PRKAG2 |
| YWHAB | Tyrosine 3-Monooxygenase/Tryptophan 5-Monooxygenase Activation Protein Beta | Protein Coding |  | 55 | GC20P044885 | | 0.45 | https://www.genecards.org/cgi-bin/carddisp.pl?gene=YWHAB |
| L1CAM | L1 Cell Adhesion Molecule | Protein Coding |  | 53 | GC0XM153864 | | 0.45 | https://www.genecards.org/cgi-bin/carddisp.pl?gene=L1CAM |
| COL4A6 | Collagen Type IV Alpha 6 Chain | Protein Coding |  | 50 | GC0XM108155 | | 0.45 | https://www.genecards.org/cgi-bin/carddisp.pl?gene=COL4A6 |
| CDA | Cytidine Deaminase | Protein Coding |  | 50 | GC01P020588 | | 0.45 | https://www.genecards.org/cgi-bin/carddisp.pl?gene=CDA |
| RAB1A | RAB1A, Member RAS Oncogene Family | Protein Coding |  | 48 | GC02M065048 | | 0.45 | https://www.genecards.org/cgi-bin/carddisp.pl?gene=RAB1A |
| TNFSF4 | TNF Superfamily Member 4 | Protein Coding |  | 46 | GC01M173152 | | 0.45 | https://www.genecards.org/cgi-bin/carddisp.pl?gene=TNFSF4 |
| SLC2A6 | Solute Carrier Family 2 Member 6 | Protein Coding |  | 45 | GC09M133471 | | 0.44 | https://www.genecards.org/cgi-bin/carddisp.pl?gene=SLC2A6 |
| DNAH14 | Dynein Axonemal Heavy Chain 14 | Protein Coding |  | 37 | GC01P224896 | | 0.44 | https://www.genecards.org/cgi-bin/carddisp.pl?gene=DNAH14 |
| ALAD | Aminolevulinate Dehydratase | Protein Coding |  | 53 | GC09M113386 | | 0.44 | https://www.genecards.org/cgi-bin/carddisp.pl?gene=ALAD |
| TAT | Tyrosine Aminotransferase | Protein Coding |  | 51 | GC16M071565 | | 0.43 | https://www.genecards.org/cgi-bin/carddisp.pl?gene=TAT |
| GPR15 | G Protein-Coupled Receptor 15 | Protein Coding |  | 44 | GC03P098531 | | 0.43 | https://www.genecards.org/cgi-bin/carddisp.pl?gene=GPR15 |
| TMEM154 | Transmembrane Protein 154 | Protein Coding |  | 37 | GC04M152618 | | 0.43 | https://www.genecards.org/cgi-bin/carddisp.pl?gene=TMEM154 |
| CFD | Complement Factor D | Protein Coding |  | 51 | GC19P000859 | | 0.43 | https://www.genecards.org/cgi-bin/carddisp.pl?gene=CFD |
| SLC5A7 | Solute Carrier Family 5 Member 7 | Protein Coding |  | 50 | GC02P107969 | | 0.43 | https://www.genecards.org/cgi-bin/carddisp.pl?gene=SLC5A7 |
| NGLY1 | N-Glycanase 1 | Protein Coding |  | 50 | GC03M025718 | | 0.43 | https://www.genecards.org/cgi-bin/carddisp.pl?gene=NGLY1 |
| MANBA | Mannosidase Beta | Protein Coding |  | 48 | GC04M102631 | | 0.43 | https://www.genecards.org/cgi-bin/carddisp.pl?gene=MANBA |
| MTR | 5-Methyltetrahydrofolate-Homocysteine Methyltransferase | Protein Coding |  | 53 | GC01P236795 | | 0.42 | https://www.genecards.org/cgi-bin/carddisp.pl?gene=MTR |
| TACR1 | Tachykinin Receptor 1 | Protein Coding |  | 52 | GC02M075010 | | 0.42 | https://www.genecards.org/cgi-bin/carddisp.pl?gene=TACR1 |
| USP14 | Ubiquitin Specific Peptidase 14 | Protein Coding |  | 51 | GC18P000158 | | 0.42 | https://www.genecards.org/cgi-bin/carddisp.pl?gene=USP14 |
| TCN1 | Transcobalamin 1 | Protein Coding |  | 46 | GC11M059871 | | 0.42 | https://www.genecards.org/cgi-bin/carddisp.pl?gene=TCN1 |
| MAPK11 | Mitogen-Activated Protein Kinase 11 | Protein Coding |  | 54 | GC22M050263 | | 0.42 | https://www.genecards.org/cgi-bin/carddisp.pl?gene=MAPK11 |
| ADSS | Adenylosuccinate Synthase | Protein Coding |  | 50 | GC01M244409 | | 0.41 | https://www.genecards.org/cgi-bin/carddisp.pl?gene=ADSS |
| RERE | Arginine-Glutamic Acid Dipeptide Repeats | Protein Coding |  | 47 | GC01M008412 | | 0.41 | https://www.genecards.org/cgi-bin/carddisp.pl?gene=RERE |
| ASGR1 | Asialoglycoprotein Receptor 1 | Protein Coding |  | 46 | GC17M007173 | | 0.41 | https://www.genecards.org/cgi-bin/carddisp.pl?gene=ASGR1 |
| NEK3 | NIMA Related Kinase 3 | Protein Coding |  | 46 | GC13M052132 | | 0.41 | https://www.genecards.org/cgi-bin/carddisp.pl?gene=NEK3 |
| AZU1 | Azurocidin 1 | Protein Coding |  | 45 | GC19P000825 | | 0.41 | https://www.genecards.org/cgi-bin/carddisp.pl?gene=AZU1 |
| CCL8 | C-C Motif Chemokine Ligand 8 | Protein Coding |  | 44 | GC17P034319 | | 0.41 | https://www.genecards.org/cgi-bin/carddisp.pl?gene=CCL8 |
| HIST1H4F | Histone Cluster 1 H4 Family Member F | Protein Coding |  | 40 | GC06P026240 | | 0.41 | https://www.genecards.org/cgi-bin/carddisp.pl?gene=HIST1H4F |
| SMC6 | Structural Maintenance Of Chromosomes 6 | Protein Coding |  | 40 | GC02M017708 | | 0.41 | https://www.genecards.org/cgi-bin/carddisp.pl?gene=SMC6 |
| CSMD1 | CUB And Sushi Multiple Domains 1 | Protein Coding |  | 42 | GC08M002953 | | 0.41 | https://www.genecards.org/cgi-bin/carddisp.pl?gene=CSMD1 |
| TARS | Threonyl-TRNA Synthetase | Protein Coding |  | 48 | GC05P033476 | | 0.41 | https://www.genecards.org/cgi-bin/carddisp.pl?gene=TARS |
| VNN1 | Vanin 1 | Protein Coding |  | 50 | GC06M132680 | | 0.4 | https://www.genecards.org/cgi-bin/carddisp.pl?gene=VNN1 |
| RING1 | Ring Finger Protein 1 | Protein Coding |  | 47 | GC06P033208 | | 0.4 | https://www.genecards.org/cgi-bin/carddisp.pl?gene=RING1 |
| ABCF1 | ATP Binding Cassette Subfamily F Member 1 | Protein Coding |  | 44 | GC06P030571 | | 0.4 | https://www.genecards.org/cgi-bin/carddisp.pl?gene=ABCF1 |
| PELO | Pelota MRNA Surveillance And Ribosome Rescue Factor | Protein Coding |  | 43 | GC05P052787 | | 0.4 | https://www.genecards.org/cgi-bin/carddisp.pl?gene=PELO |
| MRPS18B | Mitochondrial Ribosomal Protein S18B | Protein Coding |  | 42 | GC06P030617 | | 0.4 | https://www.genecards.org/cgi-bin/carddisp.pl?gene=MRPS18B |
| BAG6 | BCL2 Associated Athanogene 6 | Protein Coding |  | 42 | GC06M031639 | | 0.4 | https://www.genecards.org/cgi-bin/carddisp.pl?gene=BAG6 |
| MX2 | MX Dynamin Like GTPase 2 | Protein Coding |  | 44 | GC21P041361 | | 0.4 | https://www.genecards.org/cgi-bin/carddisp.pl?gene=MX2 |
| GLRX2 | Glutaredoxin 2 | Protein Coding |  | 43 | GC01M193065 | | 0.4 | https://www.genecards.org/cgi-bin/carddisp.pl?gene=GLRX2 |
| NME2 | NME/NM23 Nucleoside Diphosphate Kinase 2 | Protein Coding |  | 53 | GC17P051155 | | 0.4 | https://www.genecards.org/cgi-bin/carddisp.pl?gene=NME2 |
| TRIO | Trio Rho Guanine Nucleotide Exchange Factor | Protein Coding |  | 52 | GC05P014143 | | 0.39 | https://www.genecards.org/cgi-bin/carddisp.pl?gene=TRIO |
| COL6A1 | Collagen Type VI Alpha 1 Chain | Protein Coding |  | 49 | GC21P045981 | | 0.39 | https://www.genecards.org/cgi-bin/carddisp.pl?gene=COL6A1 |
| NAA10 | N(Alpha)-Acetyltransferase 10, NatA Catalytic Subunit | Protein Coding |  | 48 | GC0XM153929 | | 0.39 | https://www.genecards.org/cgi-bin/carddisp.pl?gene=NAA10 |
| HPSE2 | Heparanase 2 (Inactive) | Protein Coding |  | 46 | GC10M098457 | | 0.39 | https://www.genecards.org/cgi-bin/carddisp.pl?gene=HPSE2 |
| PTPN1 | Protein Tyrosine Phosphatase Non-Receptor Type 1 | Protein Coding |  | 56 | GC20P050510 | | 0.39 | https://www.genecards.org/cgi-bin/carddisp.pl?gene=PTPN1 |
| FAAH | Fatty Acid Amide Hydrolase | Protein Coding |  | 53 | GC01P046394 | | 0.39 | https://www.genecards.org/cgi-bin/carddisp.pl?gene=FAAH |
| HSPA1L | Heat Shock Protein Family A (Hsp70) Member 1 Like | Protein Coding |  | 50 | GC06M031809 | | 0.39 | https://www.genecards.org/cgi-bin/carddisp.pl?gene=HSPA1L |
| RPS16 | Ribosomal Protein S16 | Protein Coding |  | 47 | GC19M039433 | | 0.39 | https://www.genecards.org/cgi-bin/carddisp.pl?gene=RPS16 |
| ZC3H12A | Zinc Finger CCCH-Type Containing 12A | Protein Coding |  | 42 | GC01P037474 | | 0.39 | https://www.genecards.org/cgi-bin/carddisp.pl?gene=ZC3H12A |
| SCFV | Single-Chain Fv Fragment | Uncategorized |  | 7 | GC14U900745 | | 0.39 | https://www.genecards.org/cgi-bin/carddisp.pl?gene=SCFV |
| UNC5C | Unc-5 Netrin Receptor C | Protein Coding |  | 46 | GC04M095162 | | 0.38 | https://www.genecards.org/cgi-bin/carddisp.pl?gene=UNC5C |
| LSM2 | LSM2 Homolog, U6 Small Nuclear RNA And MRNA Degradation Associated | Protein Coding |  | 45 | GC06M032172 | | 0.38 | https://www.genecards.org/cgi-bin/carddisp.pl?gene=LSM2 |
| ORMDL3 | ORMDL Sphingolipid Biosynthesis Regulator 3 | Protein Coding |  | 45 | GC17M039921 | | 0.38 | https://www.genecards.org/cgi-bin/carddisp.pl?gene=ORMDL3 |
| SUGT1 | SGT1 Homolog, MIS12 Kinetochore Complex Assembly Cochaperone | Protein Coding |  | 43 | GC13P052652 | | 0.38 | https://www.genecards.org/cgi-bin/carddisp.pl?gene=SUGT1 |
| MED24 | Mediator Complex Subunit 24 | Protein Coding |  | 42 | GC17M040019 | | 0.38 | https://www.genecards.org/cgi-bin/carddisp.pl?gene=MED24 |
| FUT5 | Fucosyltransferase 5 | Protein Coding |  | 42 | GC19M005865 | | 0.38 | https://www.genecards.org/cgi-bin/carddisp.pl?gene=FUT5 |
| MSL1 | MSL Complex Subunit 1 | Protein Coding |  | 40 | GC17P040122 | | 0.38 | https://www.genecards.org/cgi-bin/carddisp.pl?gene=MSL1 |
| PRRC2A | Proline Rich Coiled-Coil 2A | Protein Coding |  | 39 | GC06P032501 | | 0.38 | https://www.genecards.org/cgi-bin/carddisp.pl?gene=PRRC2A |
| RPAP3 | RNA Polymerase II Associated Protein 3 | Protein Coding |  | 37 | GC12M047661 | | 0.38 | https://www.genecards.org/cgi-bin/carddisp.pl?gene=RPAP3 |
| INPP5D | Inositol Polyphosphate-5-Phosphatase D | Protein Coding |  | 52 | GC02P233059 | | 0.37 | https://www.genecards.org/cgi-bin/carddisp.pl?gene=INPP5D |
| TXN2 | Thioredoxin 2 | Protein Coding |  | 52 | GC22M036467 | | 0.37 | https://www.genecards.org/cgi-bin/carddisp.pl?gene=TXN2 |
| TRPC4 | Transient Receptor Potential Cation Channel Subfamily C Member 4 | Protein Coding |  | 51 | GC13M037636 | | 0.37 | https://www.genecards.org/cgi-bin/carddisp.pl?gene=TRPC4 |
| MPI | Mannose Phosphate Isomerase | Protein Coding |  | 50 | GC15P074890 | | 0.37 | https://www.genecards.org/cgi-bin/carddisp.pl?gene=MPI |
| ORAI3 | ORAI Calcium Release-Activated Calcium Modulator 3 | Protein Coding |  | 38 | GC16P030949 | | 0.37 | https://www.genecards.org/cgi-bin/carddisp.pl?gene=ORAI3 |
| POLR3A | RNA Polymerase III Subunit A | Protein Coding |  | 50 | GC10M077969 | | 0.37 | https://www.genecards.org/cgi-bin/carddisp.pl?gene=POLR3A |
| PRPS1 | Phosphoribosyl Pyrophosphate Synthetase 1 | Protein Coding |  | 50 | GC0XP107628 | | 0.37 | https://www.genecards.org/cgi-bin/carddisp.pl?gene=PRPS1 |
| EXT1 | Exostosin Glycosyltransferase 1 | Protein Coding |  | 54 | GC08M117798 | | 0.35 | https://www.genecards.org/cgi-bin/carddisp.pl?gene=EXT1 |
| RPL11 | Ribosomal Protein L11 | Protein Coding |  | 54 | GC01P023691 | | 0.35 | https://www.genecards.org/cgi-bin/carddisp.pl?gene=RPL11 |
| CRKL | CRK Like Proto-Oncogene, Adaptor Protein | Protein Coding |  | 53 | GC22P020917 | | 0.35 | https://www.genecards.org/cgi-bin/carddisp.pl?gene=CRKL |
| DHCR7 | 7-Dehydrocholesterol Reductase | Protein Coding |  | 52 | GC11M071428 | | 0.35 | https://www.genecards.org/cgi-bin/carddisp.pl?gene=DHCR7 |
| MYO5A | Myosin VA | Protein Coding |  | 50 | GC15M057793 | | 0.35 | https://www.genecards.org/cgi-bin/carddisp.pl?gene=MYO5A |
| SMARCAL1 | SWI/SNF Related, Matrix Associated, Actin Dependent Regulator Of Chromatin, Subfamily A Like 1 | Protein Coding |  | 50 | GC02P216412 | | 0.35 | https://www.genecards.org/cgi-bin/carddisp.pl?gene=SMARCAL1 |
| GTF2I | General Transcription Factor IIi | Protein Coding |  | 48 | GC07P074658 | | 0.35 | https://www.genecards.org/cgi-bin/carddisp.pl?gene=GTF2I |
| DGCR8 | DGCR8 Microprocessor Complex Subunit | Protein Coding |  | 47 | GC22P020080 | | 0.35 | https://www.genecards.org/cgi-bin/carddisp.pl?gene=DGCR8 |
| SLC35A2 | Solute Carrier Family 35 Member A2 | Protein Coding |  | 46 | GC0XM048903 | | 0.35 | https://www.genecards.org/cgi-bin/carddisp.pl?gene=SLC35A2 |
| CKM | Creatine Kinase, M-Type | Protein Coding |  | 51 | GC19M045306 | | 0.35 | https://www.genecards.org/cgi-bin/carddisp.pl?gene=CKM |
| BLMH | Bleomycin Hydrolase | Protein Coding |  | 50 | GC17M030248 | | 0.35 | https://www.genecards.org/cgi-bin/carddisp.pl?gene=BLMH |
| CUL5 | Cullin 5 | Protein Coding |  | 50 | GC11P108008 | | 0.35 | https://www.genecards.org/cgi-bin/carddisp.pl?gene=CUL5 |
| ETS2 | ETS Proto-Oncogene 2, Transcription Factor | Protein Coding |  | 49 | GC21P038805 | | 0.35 | https://www.genecards.org/cgi-bin/carddisp.pl?gene=ETS2 |
| MAP1B | Microtubule Associated Protein 1B | Protein Coding |  | 48 | GC05P072107 | | 0.35 | https://www.genecards.org/cgi-bin/carddisp.pl?gene=MAP1B |
| SEMA7A | Semaphorin 7A (John Milton Hagen Blood Group) | Protein Coding |  | 48 | GC15M074409 | | 0.35 | https://www.genecards.org/cgi-bin/carddisp.pl?gene=SEMA7A |
| NFKBIB | NFKB Inhibitor Beta | Protein Coding |  | 46 | GC19P038899 | | 0.35 | https://www.genecards.org/cgi-bin/carddisp.pl?gene=NFKBIB |
| NUDT2 | Nudix Hydrolase 2 | Protein Coding |  | 46 | GC09P034329 | | 0.35 | https://www.genecards.org/cgi-bin/carddisp.pl?gene=NUDT2 |
| CST6 | Cystatin E/M | Protein Coding |  | 45 | GC11P066029 | | 0.35 | https://www.genecards.org/cgi-bin/carddisp.pl?gene=CST6 |
| NPLOC4 | NPL4 Homolog, Ubiquitin Recognition Factor | Protein Coding |  | 45 | GC17M081556 | | 0.35 | https://www.genecards.org/cgi-bin/carddisp.pl?gene=NPLOC4 |
| ROR2 | Receptor Tyrosine Kinase Like Orphan Receptor 2 | Protein Coding |  | 54 | GC09M091564 | | 0.33 | https://www.genecards.org/cgi-bin/carddisp.pl?gene=ROR2 |
| MTHFD1 | Methylenetetrahydrofolate Dehydrogenase, Cyclohydrolase And Formyltetrahydrofolate Synthetase 1 | Protein Coding |  | 53 | GC14P064388 | | 0.33 | https://www.genecards.org/cgi-bin/carddisp.pl?gene=MTHFD1 |
| ITGA7 | Integrin Subunit Alpha 7 | Protein Coding |  | 53 | GC12M055684 | | 0.33 | https://www.genecards.org/cgi-bin/carddisp.pl?gene=ITGA7 |
| PKD2 | Polycystin 2, Transient Receptor Potential Cation Channel | Protein Coding |  | 53 | GC04P088007 | | 0.33 | https://www.genecards.org/cgi-bin/carddisp.pl?gene=PKD2 |
| FOXP1 | Forkhead Box P1 | Protein Coding |  | 51 | GC03M070926 | | 0.33 | https://www.genecards.org/cgi-bin/carddisp.pl?gene=FOXP1 |
| CTPS1 | CTP Synthase 1 | Protein Coding |  | 51 | GC01P040979 | | 0.33 | https://www.genecards.org/cgi-bin/carddisp.pl?gene=CTPS1 |
| DDOST | Dolichyl-Diphosphooligosaccharide--Protein Glycosyltransferase Non-Catalytic Subunit | Protein Coding |  | 51 | GC01M020651 | | 0.33 | https://www.genecards.org/cgi-bin/carddisp.pl?gene=DDOST |
| ARSB | Arylsulfatase B | Protein Coding |  | 51 | GC05M078777 | | 0.33 | https://www.genecards.org/cgi-bin/carddisp.pl?gene=ARSB |
| AP3B1 | Adaptor Related Protein Complex 3 Subunit Beta 1 | Protein Coding |  | 51 | GC05M078000 | | 0.33 | https://www.genecards.org/cgi-bin/carddisp.pl?gene=AP3B1 |
| SNRPN | Small Nuclear Ribonucleoprotein Polypeptide N | Protein Coding |  | 51 | GC15P024823 | | 0.33 | https://www.genecards.org/cgi-bin/carddisp.pl?gene=SNRPN |
| TNFSF15 | TNF Superfamily Member 15 | Protein Coding |  | 51 | GC09M114784 | | 0.33 | https://www.genecards.org/cgi-bin/carddisp.pl?gene=TNFSF15 |
| TPM2 | Tropomyosin 2 | Protein Coding |  | 51 | GC09M035672 | | 0.33 | https://www.genecards.org/cgi-bin/carddisp.pl?gene=TPM2 |
| ALOX12B | Arachidonate 12-Lipoxygenase, 12R Type | Protein Coding |  | 50 | GC17M008619 | | 0.33 | https://www.genecards.org/cgi-bin/carddisp.pl?gene=ALOX12B |
| CLPB | ClpB Homolog, Mitochondrial AAA ATPase Chaperonin | Protein Coding |  | 49 | GC11M072292 | | 0.33 | https://www.genecards.org/cgi-bin/carddisp.pl?gene=CLPB |
| PLEC | Plectin | Protein Coding |  | 49 | GC08M143916 | | 0.33 | https://www.genecards.org/cgi-bin/carddisp.pl?gene=PLEC |
| IDUA | Iduronidase Alpha-L- | Protein Coding |  | 48 | GC04P000980 | | 0.33 | https://www.genecards.org/cgi-bin/carddisp.pl?gene=IDUA |
| ATP6AP1 | ATPase H+ Transporting Accessory Protein 1 | Protein Coding |  | 48 | GC0XP154428 | | 0.33 | https://www.genecards.org/cgi-bin/carddisp.pl?gene=ATP6AP1 |
| CLDN16 | Claudin 16 | Protein Coding |  | 48 | GC03P190322 | | 0.33 | https://www.genecards.org/cgi-bin/carddisp.pl?gene=CLDN16 |
| SDCCAG8 | Serologically Defined Colon Cancer Antigen 8 | Protein Coding |  | 48 | GC01P243255 | | 0.33 | https://www.genecards.org/cgi-bin/carddisp.pl?gene=SDCCAG8 |
| NXN | Nucleoredoxin | Protein Coding |  | 48 | GC17M000799 | | 0.33 | https://www.genecards.org/cgi-bin/carddisp.pl?gene=NXN |
| NSUN2 | NOP2/Sun RNA Methyltransferase 2 | Protein Coding |  | 48 | GC05M006599 | | 0.33 | https://www.genecards.org/cgi-bin/carddisp.pl?gene=NSUN2 |
| SLC25A22 | Solute Carrier Family 25 Member 22 | Protein Coding |  | 47 | GC11M000791 | | 0.33 | https://www.genecards.org/cgi-bin/carddisp.pl?gene=SLC25A22 |
| KCTD1 | Potassium Channel Tetramerization Domain Containing 1 | Protein Coding |  | 46 | GC18M026454 | | 0.33 | https://www.genecards.org/cgi-bin/carddisp.pl?gene=KCTD1 |
| EXOSC9 | Exosome Component 9 | Protein Coding |  | 46 | GC04P121801 | | 0.33 | https://www.genecards.org/cgi-bin/carddisp.pl?gene=EXOSC9 |
| ADNP | Activity Dependent Neuroprotector Homeobox | Protein Coding |  | 46 | GC20M050888 | | 0.33 | https://www.genecards.org/cgi-bin/carddisp.pl?gene=ADNP |
| VPS33A | VPS33A Core Subunit Of CORVET And HOPS Complexes | Protein Coding |  | 46 | GC12M122229 | | 0.33 | https://www.genecards.org/cgi-bin/carddisp.pl?gene=VPS33A |
| TNPO3 | Transportin 3 | Protein Coding |  | 46 | GC07M128954 | | 0.33 | https://www.genecards.org/cgi-bin/carddisp.pl?gene=TNPO3 |
| NME8 | NME/NM23 Family Member 8 | Protein Coding |  | 45 | GC07P037889 | | 0.33 | https://www.genecards.org/cgi-bin/carddisp.pl?gene=NME8 |
| CRIPT | CXXC Repeat Containing Interactor Of PDZ3 Domain | Protein Coding |  | 45 | GC02P046581 | | 0.33 | https://www.genecards.org/cgi-bin/carddisp.pl?gene=CRIPT |
| TBX6 | T-Box 6 | Protein Coding |  | 45 | GC16M030097 | | 0.33 | https://www.genecards.org/cgi-bin/carddisp.pl?gene=TBX6 |
| TBL2 | Transducin Beta Like 2 | Protein Coding |  | 45 | GC07M073568 | | 0.33 | https://www.genecards.org/cgi-bin/carddisp.pl?gene=TBL2 |
| NECTIN1 | Nectin Cell Adhesion Molecule 1 | Protein Coding |  | 43 | GC11M119624 | | 0.33 | https://www.genecards.org/cgi-bin/carddisp.pl?gene=NECTIN1 |
| PWAR1 | Prader Willi/Angelman Region RNA 1 | RNA Gene |  | 17 | GC15P025135 | | 0.33 | https://www.genecards.org/cgi-bin/carddisp.pl?gene=PWAR1 |
| SCN5A | Sodium Voltage-Gated Channel Alpha Subunit 5 | Protein Coding |  | 57 | GC03M038549 | | 0.32 | https://www.genecards.org/cgi-bin/carddisp.pl?gene=SCN5A |
| ACADS | Acyl-CoA Dehydrogenase Short Chain | Protein Coding |  | 53 | GC12P120798 | | 0.32 | https://www.genecards.org/cgi-bin/carddisp.pl?gene=ACADS |
| SERPINI1 | Serpin Family I Member 1 | Protein Coding |  | 52 | GC03P167735 | | 0.32 | https://www.genecards.org/cgi-bin/carddisp.pl?gene=SERPINI1 |
| MFGE8 | Milk Fat Globule-EGF Factor 8 Protein | Protein Coding |  | 49 | GC15M088898 | | 0.32 | https://www.genecards.org/cgi-bin/carddisp.pl?gene=MFGE8 |
| RAPGEF4 | Rap Guanine Nucleotide Exchange Factor 4 | Protein Coding |  | 49 | GC02P172735 | | 0.32 | https://www.genecards.org/cgi-bin/carddisp.pl?gene=RAPGEF4 |
| TACR2 | Tachykinin Receptor 2 | Protein Coding |  | 49 | GC10M069403 | | 0.32 | https://www.genecards.org/cgi-bin/carddisp.pl?gene=TACR2 |
| RNASE1 | Ribonuclease A Family Member 1, Pancreatic | Protein Coding |  | 46 | GC14M020801 | | 0.32 | https://www.genecards.org/cgi-bin/carddisp.pl?gene=RNASE1 |
| IL27 | Interleukin 27 | Protein Coding |  | 45 | GC16M028511 | | 0.32 | https://www.genecards.org/cgi-bin/carddisp.pl?gene=IL27 |
| FKRP | Fukutin Related Protein | Protein Coding |  | 45 | GC19P046746 | | 0.32 | https://www.genecards.org/cgi-bin/carddisp.pl?gene=FKRP |
| CARD8 | Caspase Recruitment Domain Family Member 8 | Protein Coding |  | 44 | GC19M048181 | | 0.32 | https://www.genecards.org/cgi-bin/carddisp.pl?gene=CARD8 |
| SAFB | Scaffold Attachment Factor B | Protein Coding |  | 43 | GC19P005574 | | 0.32 | https://www.genecards.org/cgi-bin/carddisp.pl?gene=SAFB |
| CDC42EP1 | CDC42 Effector Protein 1 | Protein Coding |  | 42 | GC22P037559 | | 0.32 | https://www.genecards.org/cgi-bin/carddisp.pl?gene=CDC42EP1 |
| DEFB103A | Defensin Beta 103A | Protein Coding |  | 31 | GC08P007881 | | 0.32 | https://www.genecards.org/cgi-bin/carddisp.pl?gene=DEFB103A |
| G3BP1 | G3BP Stress Granule Assembly Factor 1 | Protein Coding |  | 45 | GC05P151771 | | 0.3 | https://www.genecards.org/cgi-bin/carddisp.pl?gene=G3BP1 |
| RPS6KA3 | Ribosomal Protein S6 Kinase A3 | Protein Coding |  | 59 | GC0XM020149 | | 0.3 | https://www.genecards.org/cgi-bin/carddisp.pl?gene=RPS6KA3 |
| KPNA1 | Karyopherin Subunit Alpha 1 | Protein Coding |  | 47 | GC03M122421 | | 0.3 | https://www.genecards.org/cgi-bin/carddisp.pl?gene=KPNA1 |
| HIST2H4A | Histone Cluster 2 H4 Family Member A | Protein Coding |  | 38 | GC01P149804 | | 0.3 | https://www.genecards.org/cgi-bin/carddisp.pl?gene=HIST2H4A |
| GLRB | Glycine Receptor Beta | Protein Coding |  | 54 | GC04P157076 | | 0.3 | https://www.genecards.org/cgi-bin/carddisp.pl?gene=GLRB |
| CA8 | Carbonic Anhydrase 8 | Protein Coding |  | 52 | GC08M060187 | | 0.3 | https://www.genecards.org/cgi-bin/carddisp.pl?gene=CA8 |
| SLC12A1 | Solute Carrier Family 12 Member 1 | Protein Coding |  | 52 | GC15P048191 | | 0.3 | https://www.genecards.org/cgi-bin/carddisp.pl?gene=SLC12A1 |
| ZNF502 | Zinc Finger Protein 502 | Protein Coding |  | 37 | GC03P045203 | | 0.3 | https://www.genecards.org/cgi-bin/carddisp.pl?gene=ZNF502 |
| LDHB | Lactate Dehydrogenase B | Protein Coding |  | 53 | GC12M021635 | | 0.27 | https://www.genecards.org/cgi-bin/carddisp.pl?gene=LDHB |
| CAV2 | Caveolin 2 | Protein Coding |  | 48 | GC07P116287 | | 0.27 | https://www.genecards.org/cgi-bin/carddisp.pl?gene=CAV2 |
| IL22RA1 | Interleukin 22 Receptor Subunit Alpha 1 | Protein Coding |  | 43 | GC01M024119 | | 0.27 | https://www.genecards.org/cgi-bin/carddisp.pl?gene=IL22RA1 |
| EEF2 | Eukaryotic Translation Elongation Factor 2 | Protein Coding |  | 55 | GC19M003976 | | 0.25 | https://www.genecards.org/cgi-bin/carddisp.pl?gene=EEF2 |
| PHYH | Phytanoyl-CoA 2-Hydroxylase | Protein Coding |  | 51 | GC10M013277 | | 0.25 | https://www.genecards.org/cgi-bin/carddisp.pl?gene=PHYH |
| RAPGEF3 | Rap Guanine Nucleotide Exchange Factor 3 | Protein Coding |  | 51 | GC12M047736 | | 0.25 | https://www.genecards.org/cgi-bin/carddisp.pl?gene=RAPGEF3 |
| LONP1 | Lon Peptidase 1, Mitochondrial | Protein Coding |  | 49 | GC19M005691 | | 0.25 | https://www.genecards.org/cgi-bin/carddisp.pl?gene=LONP1 |
| EDA | Ectodysplasin A | Protein Coding |  | 48 | GC0XP069618 | | 0.25 | https://www.genecards.org/cgi-bin/carddisp.pl?gene=EDA |
| COL4A5 | Collagen Type IV Alpha 5 Chain | Protein Coding |  | 48 | GC0XP108439 | | 0.25 | https://www.genecards.org/cgi-bin/carddisp.pl?gene=COL4A5 |
| MRPL14 | Mitochondrial Ribosomal Protein L14 | Protein Coding |  | 42 | GC06M044189 | | 0.25 | https://www.genecards.org/cgi-bin/carddisp.pl?gene=MRPL14 |
| CES1 | Carboxylesterase 1 | Protein Coding |  | 53 | GC16M055836 | | 0.22 | https://www.genecards.org/cgi-bin/carddisp.pl?gene=CES1 |
| TPT1 | Tumor Protein, Translationally-Controlled 1 | Protein Coding |  | 53 | GC13M045333 | | 0.22 | https://www.genecards.org/cgi-bin/carddisp.pl?gene=TPT1 |
| TREH | Trehalase | Protein Coding |  | 49 | GC11M118562 | | 0.22 | https://www.genecards.org/cgi-bin/carddisp.pl?gene=TREH |
| HBA2 | Hemoglobin Subunit Alpha 2 | Protein Coding |  | 48 | GC16P000576 | | 0.22 | https://www.genecards.org/cgi-bin/carddisp.pl?gene=HBA2 |
| SUMO3 | Small Ubiquitin Like Modifier 3 | Protein Coding |  | 48 | GC21M044805 | | 0.22 | https://www.genecards.org/cgi-bin/carddisp.pl?gene=SUMO3 |
| HSPA1B | Heat Shock Protein Family A (Hsp70) Member 1B | Protein Coding |  | 46 | GC06P032517 | | 0.22 | https://www.genecards.org/cgi-bin/carddisp.pl?gene=HSPA1B |
| KPNA6 | Karyopherin Subunit Alpha 6 | Protein Coding |  | 45 | GC01P032108 | | 0.22 | https://www.genecards.org/cgi-bin/carddisp.pl?gene=KPNA6 |
| IGSF3 | Immunoglobulin Superfamily Member 3 | Protein Coding |  | 45 | GC01M116574 | | 0.22 | https://www.genecards.org/cgi-bin/carddisp.pl?gene=IGSF3 |
| GK2 | Glycerol Kinase 2 | Protein Coding |  | 45 | GC04M079406 | | 0.22 | https://www.genecards.org/cgi-bin/carddisp.pl?gene=GK2 |
| DDT | D-Dopachrome Tautomerase | Protein Coding |  | 45 | GC22M023971 | | 0.22 | https://www.genecards.org/cgi-bin/carddisp.pl?gene=DDT |
| CHST1 | Carbohydrate Sulfotransferase 1 | Protein Coding |  | 43 | GC11M045647 | | 0.22 | https://www.genecards.org/cgi-bin/carddisp.pl?gene=CHST1 |
| S100A16 | S100 Calcium Binding Protein A16 | Protein Coding |  | 43 | GC01M153606 | | 0.22 | https://www.genecards.org/cgi-bin/carddisp.pl?gene=S100A16 |
| FAIM2 | Fas Apoptotic Inhibitory Molecule 2 | Protein Coding |  | 42 | GC12M049866 | | 0.22 | https://www.genecards.org/cgi-bin/carddisp.pl?gene=FAIM2 |
| MALSU1 | Mitochondrial Assembly Of Ribosomal Large Subunit 1 | Protein Coding |  | 38 | GC07P023338 | | 0.22 | https://www.genecards.org/cgi-bin/carddisp.pl?gene=MALSU1 |
| LOC107372315 | OSGEP/APEX1 Bi-Directional Promoter Region | Biological Region |  | 3 | GC14U902072 | | 0.22 | https://www.genecards.org/cgi-bin/carddisp.pl?gene=LOC107372315 |

Table S3 Network of Traditional Chinese Medicine-Active Ingredient-Target Genes-Disease

| Node1 | Node2 | Net |
| --- | --- | --- |
| Radiation pneumonitis | MMP2 | disease |
| Radiation pneumonitis | XDH | disease |
| Radiation pneumonitis | EDN1 | disease |
| Radiation pneumonitis | ERG | disease |
| Radiation pneumonitis | MET | disease |
| Radiation pneumonitis | BIRC5 | disease |
| Radiation pneumonitis | BCL2 | disease |
| Radiation pneumonitis | F7 | disease |
| Radiation pneumonitis | CYP1A1 | disease |
| Radiation pneumonitis | GSTP1 | disease |
| Radiation pneumonitis | TNF | disease |
| Radiation pneumonitis | PTGS2 | disease |
| Radiation pneumonitis | CCND1 | disease |
| Radiation pneumonitis | VEGFA | disease |
| Radiation pneumonitis | MYC | disease |
| Radiation pneumonitis | CCNA2 | disease |
| Radiation pneumonitis | GSK3B | disease |
| Radiation pneumonitis | HMOX1 | disease |
| Radiation pneumonitis | HTR1A | disease |
| Radiation pneumonitis | IL10 | disease |
| Radiation pneumonitis | MAPK1 | disease |
| Radiation pneumonitis | MDM2 | disease |
| Radiation pneumonitis | EGFR | disease |
| Radiation pneumonitis | ERBB2 | disease |
| Radiation pneumonitis | IL4 | disease |
| Radiation pneumonitis | F10 | disease |
| Radiation pneumonitis | RB1 | disease |
| Radiation pneumonitis | PGR | disease |
| Radiation pneumonitis | TP53 | disease |
| Radiation pneumonitis | CASP9 | disease |
| Radiation pneumonitis | CDKN1A | disease |
| Radiation pneumonitis | NFKBIA | disease |
| Radiation pneumonitis | ESR2 | disease |
| Radiation pneumonitis | SLC6A3 | disease |
| Radiation pneumonitis | CHRNA7 | disease |
| Radiation pneumonitis | FASN | disease |
| Radiation pneumonitis | DPP4 | disease |
| Radiation pneumonitis | KCNH2 | disease |
| Radiation pneumonitis | NR3C1 | disease |
| Radiation pneumonitis | MAPK14 | disease |
| Radiation pneumonitis | DRD2 | disease |
| Radiation pneumonitis | PCNA | disease |
| Radiation pneumonitis | STAT3 | disease |
| Radiation pneumonitis | CASP7 | disease |
| Radiation pneumonitis | ADRB2 | disease |
| Radiation pneumonitis | ACHE | disease |
| Radiation pneumonitis | MCL1 | disease |
| Radiation pneumonitis | IL6 | disease |
| Radiation pneumonitis | CASP3 | disease |
| Radiation pneumonitis | PIK3CA | disease |
| Radiation pneumonitis | IL2 | disease |
| Radiation pneumonitis | FOS | disease |
| Radiation pneumonitis | IFNG | disease |
| Radiation pneumonitis | TOP2A | disease |
| Radiation pneumonitis | ICAM1 | disease |
| Radiation pneumonitis | BCL2L1 | disease |
| Radiation pneumonitis | RELA | disease |
| Radiation pneumonitis | NOS3 | disease |
| Radiation pneumonitis | INSR | disease |
| Radiation pneumonitis | AKT1 | disease |
| Radiation pneumonitis | ITGB3 | disease |
| Radiation pneumonitis | PTGS1 | disease |
| Radiation pneumonitis | JUN | disease |
| Radiation pneumonitis | AR | disease |
| Radiation pneumonitis | CYP3A4 | disease |
| Radiation pneumonitis | SCN5A | disease |
| Radiation pneumonitis | APP | disease |
| Radiation pneumonitis | PRSS1 | disease |
| Radiation pneumonitis | MMP9 | disease |
| Radiation pneumonitis | TOP1 | disease |
| Radix Salviae | MOL007145 | mol |
| Radix Salviae | MOL000006 | mol |
| Radix Salviae | MOL002222 | mol |
| Radix Salviae | MOL002651 | mol |
| Radix Salviae | MOL007036 | mol |
| Radix Salviae | MOL007045 | mol |
| Radix Salviae | MOL007059 | mol |
| Radix Salviae | MOL007061 | mol |
| Radix Salviae | MOL007069 | mol |
| Radix Salviae | MOL007070 | mol |
| Radix Salviae | MOL007079 | mol |
| Radix Salviae | MOL007093 | mol |
| Radix Salviae | MOL007094 | mol |
| Radix Salviae | MOL007100 | mol |
| Radix Salviae | MOL007107 | mol |
| Radix Salviae | MOL007108 | mol |
| Radix Salviae | MOL007111 | mol |
| Radix Salviae | MOL007120 | mol |
| Radix Salviae | MOL007121 | mol |
| Radix Salviae | MOL007127 | mol |
| Radix Salviae | MOL007143 | mol |
| Radix Salviae | MOL007150 | mol |
| Radix Salviae | MOL007151 | mol |
| Radix Salviae | MOL007152 | mol |
| Radix Salviae | MOL007154 | mol |
| Radix Salviae | MOL007155 | mol |
| Radix Salviae | MOL007088 | mol |
| Radix Salviae | MOL007041 | mol |
| Radix Salviae | MOL007049 | mol |
| Radix Salviae | MOL007050 | mol |
| Radix Salviae | MOL007058 | mol |
| Radix Salviae | MOL007085 | mol |
| Radix Salviae | MOL007098 | mol |
| Radix Salviae | MOL007119 | mol |
| Radix Salviae | MOL007122 | mol |
| Radix Salviae | MOL007124 | mol |
| Radix Salviae | MOL007130 | mol |
| Radix Salviae | MOL007132 | mol |
| Radix Salviae | MOL007156 | mol |
| Radix Salviae | MOL001601 | mol |
| Radix Salviae | MOL007101 | mol |
| Radix Salviae | MOL007105 | mol |
| Radix Salviae | MOL007125 | mol |
| Radix Salviae | MOL007142 | mol |
| Radix Salviae | MOL002776 | mol |
| Radix Salviae | MOL007082 | mol |
| Radix Salviae | MOL007068 | mol |
| Radix Salviae | MOL007071 | mol |
| Radix Salviae | MOL007063 | mol |
| Radix Salviae | MOL007064 | mol |
| Radix Salviae | MOL007081 | mol |
| Radix Salviae | MOL001659 | mol |
| Radix Salviae | MOL001771 | mol |
| Radix Salviae | MOL001942 | mol |
| Radix Salviae | MOL000569 | mol |
| Radix Salviae | MOL007048 | mol |
| Radix Salviae | MOL007077 | mol |
| Radix Salviae | MOL007141 | mol |
| MOL007145 | HTR1A | target |
| MOL000006 | MMP2 | target |
| MOL002222 | ACHE | target |
| MOL002651 | ACHE | target |
| MOL007036 | ACHE | target |
| MOL007045 | ACHE | target |
| MOL007059 | ACHE | target |
| MOL007061 | ACHE | target |
| MOL007069 | ACHE | target |
| MOL007070 | ACHE | target |
| MOL007079 | ACHE | target |
| MOL007093 | ACHE | target |
| MOL007094 | ACHE | target |
| MOL007100 | ACHE | target |
| MOL007107 | ACHE | target |
| MOL007108 | ACHE | target |
| MOL007111 | ACHE | target |
| MOL007120 | ACHE | target |
| MOL007121 | ACHE | target |
| MOL007127 | ACHE | target |
| MOL007143 | ACHE | target |
| MOL007145 | ACHE | target |
| MOL007150 | ACHE | target |
| MOL007151 | ACHE | target |
| MOL007152 | ACHE | target |
| MOL007154 | ACHE | target |
| MOL007155 | ACHE | target |
| MOL000006 | APP | target |
| MOL007088 | APP | target |
| MOL002651 | AR | target |
| MOL000006 | AR | target |
| MOL007041 | AR | target |
| MOL007049 | AR | target |
| MOL007050 | AR | target |
| MOL007058 | AR | target |
| MOL007085 | AR | target |
| MOL007093 | AR | target |
| MOL007098 | AR | target |
| MOL007100 | AR | target |
| MOL007108 | AR | target |
| MOL007111 | AR | target |
| MOL007119 | AR | target |
| MOL007122 | AR | target |
| MOL007124 | AR | target |
| MOL007130 | AR | target |
| MOL007132 | AR | target |
| MOL007156 | AR | target |
| MOL007154 | BCL2 | target |
| MOL000006 | BIRC5 | target |
| MOL007088 | BIRC5 | target |
| MOL000006 | BCL2L1 | target |
| MOL007088 | BCL2L1 | target |
| MOL001601 | ADRB2 | target |
| MOL002222 | ADRB2 | target |
| MOL002651 | ADRB2 | target |
| MOL007036 | ADRB2 | target |
| MOL007041 | ADRB2 | target |
| MOL007045 | ADRB2 | target |
| MOL007049 | ADRB2 | target |
| MOL007059 | ADRB2 | target |
| MOL007061 | ADRB2 | target |
| MOL007069 | ADRB2 | target |
| MOL007079 | ADRB2 | target |
| MOL007088 | ADRB2 | target |
| MOL007093 | ADRB2 | target |
| MOL007094 | ADRB2 | target |
| MOL007098 | ADRB2 | target |
| MOL007100 | ADRB2 | target |
| MOL007101 | ADRB2 | target |
| MOL007105 | ADRB2 | target |
| MOL007107 | ADRB2 | target |
| MOL007108 | ADRB2 | target |
| MOL007111 | ADRB2 | target |
| MOL007119 | ADRB2 | target |
| MOL007122 | ADRB2 | target |
| MOL007124 | ADRB2 | target |
| MOL007125 | ADRB2 | target |
| MOL007127 | ADRB2 | target |
| MOL007145 | ADRB2 | target |
| MOL007154 | ADRB2 | target |
| MOL007155 | ADRB2 | target |
| MOL000006 | CASP3 | target |
| MOL007154 | CASP3 | target |
| MOL000006 | CASP7 | target |
| MOL000006 | CASP9 | target |
| MOL000006 | TP53 | target |
| MOL007154 | TP53 | target |
| MOL007142 | F7 | target |
| MOL002776 | F10 | target |
| MOL007082 | F10 | target |
| MOL007093 | F10 | target |
| MOL007108 | F10 | target |
| MOL007119 | F10 | target |
| MOL007156 | F10 | target |
| MOL007041 | CCNA2 | target |
| MOL007050 | CCNA2 | target |
| MOL007093 | CCNA2 | target |
| MOL007100 | CCNA2 | target |
| MOL007111 | CCNA2 | target |
| MOL007119 | CCNA2 | target |
| MOL007124 | CCNA2 | target |
| MOL007132 | CCNA2 | target |
| MOL000006 | CDKN1A | target |
| MOL007154 | CDKN1A | target |
| MOL007154 | CYP1A1 | target |
| MOL007154 | CYP3A4 | target |
| MOL002222 | DRD2 | target |
| MOL007049 | DRD2 | target |
| MOL007108 | DRD2 | target |
| MOL007145 | DRD2 | target |
| MOL002651 | DPP4 | target |
| MOL000006 | DPP4 | target |
| MOL007045 | DPP4 | target |
| MOL007058 | DPP4 | target |
| MOL007059 | DPP4 | target |
| MOL007061 | DPP4 | target |
| MOL007068 | DPP4 | target |
| MOL007069 | DPP4 | target |
| MOL007070 | DPP4 | target |
| MOL007071 | DPP4 | target |
| MOL007079 | DPP4 | target |
| MOL007093 | DPP4 | target |
| MOL007094 | DPP4 | target |
| MOL007100 | DPP4 | target |
| MOL007111 | DPP4 | target |
| MOL007127 | DPP4 | target |
| MOL007132 | DPP4 | target |
| MOL007150 | DPP4 | target |
| MOL007151 | DPP4 | target |
| MOL007152 | DPP4 | target |
| MOL007154 | DPP4 | target |
| MOL007155 | DPP4 | target |
| MOL000006 | TOP1 | target |
| MOL000006 | TOP2A | target |
| MOL000006 | MDM2 | target |
| MOL007088 | EDN1 | target |
| MOL007154 | EDN1 | target |
| MOL000006 | EGFR | target |
| MOL007050 | ESR2 | target |
| MOL007085 | ESR2 | target |
| MOL007093 | ESR2 | target |
| MOL007111 | ESR2 | target |
| MOL007154 | FASN | target |
| MOL000006 | CCND1 | target |
| MOL007088 | CCND1 | target |
| MOL007063 | NR3C1 | target |
| MOL007064 | NR3C1 | target |
| MOL007081 | NR3C1 | target |
| MOL007119 | NR3C1 | target |
| MOL007120 | NR3C1 | target |
| MOL007143 | NR3C1 | target |
| MOL000006 | GSTP1 | target |
| MOL007050 | GSK3B | target |
| MOL007093 | GSK3B | target |
| MOL007098 | GSK3B | target |
| MOL007100 | GSK3B | target |
| MOL007111 | GSK3B | target |
| MOL007119 | GSK3B | target |
| MOL007124 | GSK3B | target |
| MOL000006 | HMOX1 | target |
| MOL000006 | MET | target |
| MOL000006 | MCL1 | target |
| MOL000006 | INSR | target |
| MOL007154 | ITGB3 | target |
| MOL000006 | ICAM1 | target |
| MOL000006 | IFNG | target |
| MOL000006 | IL10 | target |
| MOL000006 | IL2 | target |
| MOL000006 | IL4 | target |
| MOL000006 | IL6 | target |
| MOL000006 | MMP9 | target |
| MOL007154 | MMP9 | target |
| MOL000006 | MAPK1 | target |
| MOL007050 | MAPK14 | target |
| MOL007154 | MYC | target |
| MOL001601 | CHRNA7 | target |
| MOL002222 | CHRNA7 | target |
| MOL002651 | CHRNA7 | target |
| MOL007041 | CHRNA7 | target |
| MOL007045 | CHRNA7 | target |
| MOL007049 | CHRNA7 | target |
| MOL007059 | CHRNA7 | target |
| MOL007061 | CHRNA7 | target |
| MOL007069 | CHRNA7 | target |
| MOL007079 | CHRNA7 | target |
| MOL007088 | CHRNA7 | target |
| MOL007098 | CHRNA7 | target |
| MOL007100 | CHRNA7 | target |
| MOL007101 | CHRNA7 | target |
| MOL007105 | CHRNA7 | target |
| MOL007108 | CHRNA7 | target |
| MOL007111 | CHRNA7 | target |
| MOL007119 | CHRNA7 | target |
| MOL007122 | CHRNA7 | target |
| MOL007124 | CHRNA7 | target |
| MOL007125 | CHRNA7 | target |
| MOL007127 | CHRNA7 | target |
| MOL007145 | CHRNA7 | target |
| MOL007154 | CHRNA7 | target |
| MOL007155 | CHRNA7 | target |
| MOL000006 | NFKBIA | target |
| MOL007154 | NFKBIA | target |
| MOL007041 | NOS3 | target |
| MOL007049 | NOS3 | target |
| MOL007098 | NOS3 | target |
| MOL007105 | NOS3 | target |
| MOL007122 | NOS3 | target |
| MOL007124 | NOS3 | target |
| MOL000006 | PIK3CA | target |
| MOL007082 | KCNH2 | target |
| MOL007093 | KCNH2 | target |
| MOL001659 | PGR | target |
| MOL001771 | PGR | target |
| MOL007064 | PGR | target |
| MOL007081 | PGR | target |
| MOL007088 | PGR | target |
| MOL007120 | PGR | target |
| MOL007143 | PGR | target |
| MOL000006 | PCNA | target |
| MOL001601 | PTGS1 | target |
| MOL000006 | PTGS1 | target |
| MOL007036 | PTGS1 | target |
| MOL007041 | PTGS1 | target |
| MOL007049 | PTGS1 | target |
| MOL007069 | PTGS1 | target |
| MOL007082 | PTGS1 | target |
| MOL007085 | PTGS1 | target |
| MOL007088 | PTGS1 | target |
| MOL007093 | PTGS1 | target |
| MOL007094 | PTGS1 | target |
| MOL007098 | PTGS1 | target |
| MOL007100 | PTGS1 | target |
| MOL007101 | PTGS1 | target |
| MOL007105 | PTGS1 | target |
| MOL007108 | PTGS1 | target |
| MOL007119 | PTGS1 | target |
| MOL007122 | PTGS1 | target |
| MOL007124 | PTGS1 | target |
| MOL007125 | PTGS1 | target |
| MOL007127 | PTGS1 | target |
| MOL007130 | PTGS1 | target |
| MOL007145 | PTGS1 | target |
| MOL007156 | PTGS1 | target |
| MOL001601 | PTGS2 | target |
| MOL001942 | PTGS2 | target |
| MOL002222 | PTGS2 | target |
| MOL002651 | PTGS2 | target |
| MOL000569 | PTGS2 | target |
| MOL000006 | PTGS2 | target |
| MOL007036 | PTGS2 | target |
| MOL007041 | PTGS2 | target |
| MOL007045 | PTGS2 | target |
| MOL007048 | PTGS2 | target |
| MOL007049 | PTGS2 | target |
| MOL007058 | PTGS2 | target |
| MOL007059 | PTGS2 | target |
| MOL007061 | PTGS2 | target |
| MOL007064 | PTGS2 | target |
| MOL007068 | PTGS2 | target |
| MOL007069 | PTGS2 | target |
| MOL007070 | PTGS2 | target |
| MOL007071 | PTGS2 | target |
| MOL007077 | PTGS2 | target |
| MOL007079 | PTGS2 | target |
| MOL007081 | PTGS2 | target |
| MOL007082 | PTGS2 | target |
| MOL007085 | PTGS2 | target |
| MOL007088 | PTGS2 | target |
| MOL007093 | PTGS2 | target |
| MOL007094 | PTGS2 | target |
| MOL007098 | PTGS2 | target |
| MOL007100 | PTGS2 | target |
| MOL007101 | PTGS2 | target |
| MOL007105 | PTGS2 | target |
| MOL007108 | PTGS2 | target |
| MOL007111 | PTGS2 | target |
| MOL007119 | PTGS2 | target |
| MOL007120 | PTGS2 | target |
| MOL007122 | PTGS2 | target |
| MOL007124 | PTGS2 | target |
| MOL007125 | PTGS2 | target |
| MOL007127 | PTGS2 | target |
| MOL007130 | PTGS2 | target |
| MOL007132 | PTGS2 | target |
| MOL007141 | PTGS2 | target |
| MOL007143 | PTGS2 | target |
| MOL007145 | PTGS2 | target |
| MOL007150 | PTGS2 | target |
| MOL007151 | PTGS2 | target |
| MOL007152 | PTGS2 | target |
| MOL007154 | PTGS2 | target |
| MOL007155 | PTGS2 | target |
| MOL007156 | PTGS2 | target |
| MOL007154 | FOS | target |
| MOL000006 | AKT1 | target |
| MOL000006 | ERBB2 | target |
| MOL000006 | RB1 | target |
| MOL007088 | STAT3 | target |
| MOL001601 | SCN5A | target |
| MOL002222 | SCN5A | target |
| MOL002651 | SCN5A | target |
| MOL007036 | SCN5A | target |
| MOL007041 | SCN5A | target |
| MOL007045 | SCN5A | target |
| MOL007049 | SCN5A | target |
| MOL007061 | SCN5A | target |
| MOL007069 | SCN5A | target |
| MOL007082 | SCN5A | target |
| MOL007088 | SCN5A | target |
| MOL007093 | SCN5A | target |
| MOL007094 | SCN5A | target |
| MOL007098 | SCN5A | target |
| MOL007100 | SCN5A | target |
| MOL007101 | SCN5A | target |
| MOL007105 | SCN5A | target |
| MOL007107 | SCN5A | target |
| MOL007108 | SCN5A | target |
| MOL007111 | SCN5A | target |
| MOL007119 | SCN5A | target |
| MOL007122 | SCN5A | target |
| MOL007124 | SCN5A | target |
| MOL007125 | SCN5A | target |
| MOL007127 | SCN5A | target |
| MOL007145 | SCN5A | target |
| MOL007154 | SCN5A | target |
| MOL007155 | SCN5A | target |
| MOL007156 | SCN5A | target |
| MOL001601 | SLC6A3 | target |
| MOL007041 | SLC6A3 | target |
| MOL007049 | SLC6A3 | target |
| MOL007100 | SLC6A3 | target |
| MOL007122 | SLC6A3 | target |
| MOL007124 | SLC6A3 | target |
| MOL007145 | SLC6A3 | target |
| MOL000006 | JUN | target |
| MOL007154 | JUN | target |
| MOL000006 | RELA | target |
| MOL007088 | RELA | target |
| MOL007154 | RELA | target |
| MOL000006 | PRSS1 | target |
| MOL007045 | PRSS1 | target |
| MOL007059 | PRSS1 | target |
| MOL007061 | PRSS1 | target |
| MOL007068 | PRSS1 | target |
| MOL007070 | PRSS1 | target |
| MOL007071 | PRSS1 | target |
| MOL007079 | PRSS1 | target |
| MOL007093 | PRSS1 | target |
| MOL007100 | PRSS1 | target |
| MOL007108 | PRSS1 | target |
| MOL007130 | PRSS1 | target |
| MOL007132 | PRSS1 | target |
| MOL007142 | PRSS1 | target |
| MOL007150 | PRSS1 | target |
| MOL007155 | PRSS1 | target |
| MOL000006 | TNF | target |
| MOL007088 | TNF | target |
| MOL000006 | VEGFA | target |
| MOL000006 | XDH | target |
