# Supplementary material for: Exploring the Pharmacological Mechanism of Radix Salvia Miltiorrhizae in the Treatment of Radiation Pneumonia by Using Network Pharmacology
Source: Front Oncol. 2021 Jul 29;11:684315. doi: 10.3389/fonc.2021.684315 (PMC8358777; doi:10.3389/fonc.2021.684315)
Supplement: Supplementary file 3 [file Table_3.docx]

Table S3 Network of Traditional Chinese Medicine-Active Ingredient-Target Genes-Disease

| Node1 | Node2 | Net |
| --- | --- | --- |
| Radiation pneumonitis | MMP2 | disease |
| Radiation pneumonitis | XDH | disease |
| Radiation pneumonitis | EDN1 | disease |
| Radiation pneumonitis | ERG | disease |
| Radiation pneumonitis | MET | disease |
| Radiation pneumonitis | BIRC5 | disease |
| Radiation pneumonitis | BCL2 | disease |
| Radiation pneumonitis | F7 | disease |
| Radiation pneumonitis | CYP1A1 | disease |
| Radiation pneumonitis | GSTP1 | disease |
| Radiation pneumonitis | TNF | disease |
| Radiation pneumonitis | PTGS2 | disease |
| Radiation pneumonitis | CCND1 | disease |
| Radiation pneumonitis | VEGFA | disease |
| Radiation pneumonitis | MYC | disease |
| Radiation pneumonitis | CCNA2 | disease |
| Radiation pneumonitis | GSK3B | disease |
| Radiation pneumonitis | HMOX1 | disease |
| Radiation pneumonitis | HTR1A | disease |
| Radiation pneumonitis | IL10 | disease |
| Radiation pneumonitis | MAPK1 | disease |
| Radiation pneumonitis | MDM2 | disease |
| Radiation pneumonitis | EGFR | disease |
| Radiation pneumonitis | ERBB2 | disease |
| Radiation pneumonitis | IL4 | disease |
| Radiation pneumonitis | F10 | disease |
| Radiation pneumonitis | RB1 | disease |
| Radiation pneumonitis | PGR | disease |
| Radiation pneumonitis | TP53 | disease |
| Radiation pneumonitis | CASP9 | disease |
| Radiation pneumonitis | CDKN1A | disease |
| Radiation pneumonitis | NFKBIA | disease |
| Radiation pneumonitis | ESR2 | disease |
| Radiation pneumonitis | SLC6A3 | disease |
| Radiation pneumonitis | CHRNA7 | disease |
| Radiation pneumonitis | FASN | disease |
| Radiation pneumonitis | DPP4 | disease |
| Radiation pneumonitis | KCNH2 | disease |
| Radiation pneumonitis | NR3C1 | disease |
| Radiation pneumonitis | MAPK14 | disease |
| Radiation pneumonitis | DRD2 | disease |
| Radiation pneumonitis | PCNA | disease |
| Radiation pneumonitis | STAT3 | disease |
| Radiation pneumonitis | CASP7 | disease |
| Radiation pneumonitis | ADRB2 | disease |
| Radiation pneumonitis | ACHE | disease |
| Radiation pneumonitis | MCL1 | disease |
| Radiation pneumonitis | IL6 | disease |
| Radiation pneumonitis | CASP3 | disease |
| Radiation pneumonitis | PIK3CA | disease |
| Radiation pneumonitis | IL2 | disease |
| Radiation pneumonitis | FOS | disease |
| Radiation pneumonitis | IFNG | disease |
| Radiation pneumonitis | TOP2A | disease |
| Radiation pneumonitis | ICAM1 | disease |
| Radiation pneumonitis | BCL2L1 | disease |
| Radiation pneumonitis | RELA | disease |
| Radiation pneumonitis | NOS3 | disease |
| Radiation pneumonitis | INSR | disease |
| Radiation pneumonitis | AKT1 | disease |
| Radiation pneumonitis | ITGB3 | disease |
| Radiation pneumonitis | PTGS1 | disease |
| Radiation pneumonitis | JUN | disease |
| Radiation pneumonitis | AR | disease |
| Radiation pneumonitis | CYP3A4 | disease |
| Radiation pneumonitis | SCN5A | disease |
| Radiation pneumonitis | APP | disease |
| Radiation pneumonitis | PRSS1 | disease |
| Radiation pneumonitis | MMP9 | disease |
| Radiation pneumonitis | TOP1 | disease |
| Radix Salviae | MOL007145 | mol |
| Radix Salviae | MOL000006 | mol |
| Radix Salviae | MOL002222 | mol |
| Radix Salviae | MOL002651 | mol |
| Radix Salviae | MOL007036 | mol |
| Radix Salviae | MOL007045 | mol |
| Radix Salviae | MOL007059 | mol |
| Radix Salviae | MOL007061 | mol |
| Radix Salviae | MOL007069 | mol |
| Radix Salviae | MOL007070 | mol |
| Radix Salviae | MOL007079 | mol |
| Radix Salviae | MOL007093 | mol |
| Radix Salviae | MOL007094 | mol |
| Radix Salviae | MOL007100 | mol |
| Radix Salviae | MOL007107 | mol |
| Radix Salviae | MOL007108 | mol |
| Radix Salviae | MOL007111 | mol |
| Radix Salviae | MOL007120 | mol |
| Radix Salviae | MOL007121 | mol |
| Radix Salviae | MOL007127 | mol |
| Radix Salviae | MOL007143 | mol |
| Radix Salviae | MOL007150 | mol |
| Radix Salviae | MOL007151 | mol |
| Radix Salviae | MOL007152 | mol |
| Radix Salviae | MOL007154 | mol |
| Radix Salviae | MOL007155 | mol |
| Radix Salviae | MOL007088 | mol |
| Radix Salviae | MOL007041 | mol |
| Radix Salviae | MOL007049 | mol |
| Radix Salviae | MOL007050 | mol |
| Radix Salviae | MOL007058 | mol |
| Radix Salviae | MOL007085 | mol |
| Radix Salviae | MOL007098 | mol |
| Radix Salviae | MOL007119 | mol |
| Radix Salviae | MOL007122 | mol |
| Radix Salviae | MOL007124 | mol |
| Radix Salviae | MOL007130 | mol |
| Radix Salviae | MOL007132 | mol |
| Radix Salviae | MOL007156 | mol |
| Radix Salviae | MOL001601 | mol |
| Radix Salviae | MOL007101 | mol |
| Radix Salviae | MOL007105 | mol |
| Radix Salviae | MOL007125 | mol |
| Radix Salviae | MOL007142 | mol |
| Radix Salviae | MOL002776 | mol |
| Radix Salviae | MOL007082 | mol |
| Radix Salviae | MOL007068 | mol |
| Radix Salviae | MOL007071 | mol |
| Radix Salviae | MOL007063 | mol |
| Radix Salviae | MOL007064 | mol |
| Radix Salviae | MOL007081 | mol |
| Radix Salviae | MOL001659 | mol |
| Radix Salviae | MOL001771 | mol |
| Radix Salviae | MOL001942 | mol |
| Radix Salviae | MOL000569 | mol |
| Radix Salviae | MOL007048 | mol |
| Radix Salviae | MOL007077 | mol |
| Radix Salviae | MOL007141 | mol |
| MOL007145 | HTR1A | target |
| MOL000006 | MMP2 | target |
| MOL002222 | ACHE | target |
| MOL002651 | ACHE | target |
| MOL007036 | ACHE | target |
| MOL007045 | ACHE | target |
| MOL007059 | ACHE | target |
| MOL007061 | ACHE | target |
| MOL007069 | ACHE | target |
| MOL007070 | ACHE | target |
| MOL007079 | ACHE | target |
| MOL007093 | ACHE | target |
| MOL007094 | ACHE | target |
| MOL007100 | ACHE | target |
| MOL007107 | ACHE | target |
| MOL007108 | ACHE | target |
| MOL007111 | ACHE | target |
| MOL007120 | ACHE | target |
| MOL007121 | ACHE | target |
| MOL007127 | ACHE | target |
| MOL007143 | ACHE | target |
| MOL007145 | ACHE | target |
| MOL007150 | ACHE | target |
| MOL007151 | ACHE | target |
| MOL007152 | ACHE | target |
| MOL007154 | ACHE | target |
| MOL007155 | ACHE | target |
| MOL000006 | APP | target |
| MOL007088 | APP | target |
| MOL002651 | AR | target |
| MOL000006 | AR | target |
| MOL007041 | AR | target |
| MOL007049 | AR | target |
| MOL007050 | AR | target |
| MOL007058 | AR | target |
| MOL007085 | AR | target |
| MOL007093 | AR | target |
| MOL007098 | AR | target |
| MOL007100 | AR | target |
| MOL007108 | AR | target |
| MOL007111 | AR | target |
| MOL007119 | AR | target |
| MOL007122 | AR | target |
| MOL007124 | AR | target |
| MOL007130 | AR | target |
| MOL007132 | AR | target |
| MOL007156 | AR | target |
| MOL007154 | BCL2 | target |
| MOL000006 | BIRC5 | target |
| MOL007088 | BIRC5 | target |
| MOL000006 | BCL2L1 | target |
| MOL007088 | BCL2L1 | target |
| MOL001601 | ADRB2 | target |
| MOL002222 | ADRB2 | target |
| MOL002651 | ADRB2 | target |
| MOL007036 | ADRB2 | target |
| MOL007041 | ADRB2 | target |
| MOL007045 | ADRB2 | target |
| MOL007049 | ADRB2 | target |
| MOL007059 | ADRB2 | target |
| MOL007061 | ADRB2 | target |
| MOL007069 | ADRB2 | target |
| MOL007079 | ADRB2 | target |
| MOL007088 | ADRB2 | target |
| MOL007093 | ADRB2 | target |
| MOL007094 | ADRB2 | target |
| MOL007098 | ADRB2 | target |
| MOL007100 | ADRB2 | target |
| MOL007101 | ADRB2 | target |
| MOL007105 | ADRB2 | target |
| MOL007107 | ADRB2 | target |
| MOL007108 | ADRB2 | target |
| MOL007111 | ADRB2 | target |
| MOL007119 | ADRB2 | target |
| MOL007122 | ADRB2 | target |
| MOL007124 | ADRB2 | target |
| MOL007125 | ADRB2 | target |
| MOL007127 | ADRB2 | target |
| MOL007145 | ADRB2 | target |
| MOL007154 | ADRB2 | target |
| MOL007155 | ADRB2 | target |
| MOL000006 | CASP3 | target |
| MOL007154 | CASP3 | target |
| MOL000006 | CASP7 | target |
| MOL000006 | CASP9 | target |
| MOL000006 | TP53 | target |
| MOL007154 | TP53 | target |
| MOL007142 | F7 | target |
| MOL002776 | F10 | target |
| MOL007082 | F10 | target |
| MOL007093 | F10 | target |
| MOL007108 | F10 | target |
| MOL007119 | F10 | target |
| MOL007156 | F10 | target |
| MOL007041 | CCNA2 | target |
| MOL007050 | CCNA2 | target |
| MOL007093 | CCNA2 | target |
| MOL007100 | CCNA2 | target |
| MOL007111 | CCNA2 | target |
| MOL007119 | CCNA2 | target |
| MOL007124 | CCNA2 | target |
| MOL007132 | CCNA2 | target |
| MOL000006 | CDKN1A | target |
| MOL007154 | CDKN1A | target |
| MOL007154 | CYP1A1 | target |
| MOL007154 | CYP3A4 | target |
| MOL002222 | DRD2 | target |
| MOL007049 | DRD2 | target |
| MOL007108 | DRD2 | target |
| MOL007145 | DRD2 | target |
| MOL002651 | DPP4 | target |
| MOL000006 | DPP4 | target |
| MOL007045 | DPP4 | target |
| MOL007058 | DPP4 | target |
| MOL007059 | DPP4 | target |
| MOL007061 | DPP4 | target |
| MOL007068 | DPP4 | target |
| MOL007069 | DPP4 | target |
| MOL007070 | DPP4 | target |
| MOL007071 | DPP4 | target |
| MOL007079 | DPP4 | target |
| MOL007093 | DPP4 | target |
| MOL007094 | DPP4 | target |
| MOL007100 | DPP4 | target |
| MOL007111 | DPP4 | target |
| MOL007127 | DPP4 | target |
| MOL007132 | DPP4 | target |
| MOL007150 | DPP4 | target |
| MOL007151 | DPP4 | target |
| MOL007152 | DPP4 | target |
| MOL007154 | DPP4 | target |
| MOL007155 | DPP4 | target |
| MOL000006 | TOP1 | target |
| MOL000006 | TOP2A | target |
| MOL000006 | MDM2 | target |
| MOL007088 | EDN1 | target |
| MOL007154 | EDN1 | target |
| MOL000006 | EGFR | target |
| MOL007050 | ESR2 | target |
| MOL007085 | ESR2 | target |
| MOL007093 | ESR2 | target |
| MOL007111 | ESR2 | target |
| MOL007154 | FASN | target |
| MOL000006 | CCND1 | target |
| MOL007088 | CCND1 | target |
| MOL007063 | NR3C1 | target |
| MOL007064 | NR3C1 | target |
| MOL007081 | NR3C1 | target |
| MOL007119 | NR3C1 | target |
| MOL007120 | NR3C1 | target |
| MOL007143 | NR3C1 | target |
| MOL000006 | GSTP1 | target |
| MOL007050 | GSK3B | target |
| MOL007093 | GSK3B | target |
| MOL007098 | GSK3B | target |
| MOL007100 | GSK3B | target |
| MOL007111 | GSK3B | target |
| MOL007119 | GSK3B | target |
| MOL007124 | GSK3B | target |
| MOL000006 | HMOX1 | target |
| MOL000006 | MET | target |
| MOL000006 | MCL1 | target |
| MOL000006 | INSR | target |
| MOL007154 | ITGB3 | target |
| MOL000006 | ICAM1 | target |
| MOL000006 | IFNG | target |
| MOL000006 | IL10 | target |
| MOL000006 | IL2 | target |
| MOL000006 | IL4 | target |
| MOL000006 | IL6 | target |
| MOL000006 | MMP9 | target |
| MOL007154 | MMP9 | target |
| MOL000006 | MAPK1 | target |
| MOL007050 | MAPK14 | target |
| MOL007154 | MYC | target |
| MOL001601 | CHRNA7 | target |
| MOL002222 | CHRNA7 | target |
| MOL002651 | CHRNA7 | target |
| MOL007041 | CHRNA7 | target |
| MOL007045 | CHRNA7 | target |
| MOL007049 | CHRNA7 | target |
| MOL007059 | CHRNA7 | target |
| MOL007061 | CHRNA7 | target |
| MOL007069 | CHRNA7 | target |
| MOL007079 | CHRNA7 | target |
| MOL007088 | CHRNA7 | target |
| MOL007098 | CHRNA7 | target |
| MOL007100 | CHRNA7 | target |
| MOL007101 | CHRNA7 | target |
| MOL007105 | CHRNA7 | target |
| MOL007108 | CHRNA7 | target |
| MOL007111 | CHRNA7 | target |
| MOL007119 | CHRNA7 | target |
| MOL007122 | CHRNA7 | target |
| MOL007124 | CHRNA7 | target |
| MOL007125 | CHRNA7 | target |
| MOL007127 | CHRNA7 | target |
| MOL007145 | CHRNA7 | target |
| MOL007154 | CHRNA7 | target |
| MOL007155 | CHRNA7 | target |
| MOL000006 | NFKBIA | target |
| MOL007154 | NFKBIA | target |
| MOL007041 | NOS3 | target |
| MOL007049 | NOS3 | target |
| MOL007098 | NOS3 | target |
| MOL007105 | NOS3 | target |
| MOL007122 | NOS3 | target |
| MOL007124 | NOS3 | target |
| MOL000006 | PIK3CA | target |
| MOL007082 | KCNH2 | target |
| MOL007093 | KCNH2 | target |
| MOL001659 | PGR | target |
| MOL001771 | PGR | target |
| MOL007064 | PGR | target |
| MOL007081 | PGR | target |
| MOL007088 | PGR | target |
| MOL007120 | PGR | target |
| MOL007143 | PGR | target |
| MOL000006 | PCNA | target |
| MOL001601 | PTGS1 | target |
| MOL000006 | PTGS1 | target |
| MOL007036 | PTGS1 | target |
| MOL007041 | PTGS1 | target |
| MOL007049 | PTGS1 | target |
| MOL007069 | PTGS1 | target |
| MOL007082 | PTGS1 | target |
| MOL007085 | PTGS1 | target |
| MOL007088 | PTGS1 | target |
| MOL007093 | PTGS1 | target |
| MOL007094 | PTGS1 | target |
| MOL007098 | PTGS1 | target |
| MOL007100 | PTGS1 | target |
| MOL007101 | PTGS1 | target |
| MOL007105 | PTGS1 | target |
| MOL007108 | PTGS1 | target |
| MOL007119 | PTGS1 | target |
| MOL007122 | PTGS1 | target |
| MOL007124 | PTGS1 | target |
| MOL007125 | PTGS1 | target |
| MOL007127 | PTGS1 | target |
| MOL007130 | PTGS1 | target |
| MOL007145 | PTGS1 | target |
| MOL007156 | PTGS1 | target |
| MOL001601 | PTGS2 | target |
| MOL001942 | PTGS2 | target |
| MOL002222 | PTGS2 | target |
| MOL002651 | PTGS2 | target |
| MOL000569 | PTGS2 | target |
| MOL000006 | PTGS2 | target |
| MOL007036 | PTGS2 | target |
| MOL007041 | PTGS2 | target |
| MOL007045 | PTGS2 | target |
| MOL007048 | PTGS2 | target |
| MOL007049 | PTGS2 | target |
| MOL007058 | PTGS2 | target |
| MOL007059 | PTGS2 | target |
| MOL007061 | PTGS2 | target |
| MOL007064 | PTGS2 | target |
| MOL007068 | PTGS2 | target |
| MOL007069 | PTGS2 | target |
| MOL007070 | PTGS2 | target |
| MOL007071 | PTGS2 | target |
| MOL007077 | PTGS2 | target |
| MOL007079 | PTGS2 | target |
| MOL007081 | PTGS2 | target |
| MOL007082 | PTGS2 | target |
| MOL007085 | PTGS2 | target |
| MOL007088 | PTGS2 | target |
| MOL007093 | PTGS2 | target |
| MOL007094 | PTGS2 | target |
| MOL007098 | PTGS2 | target |
| MOL007100 | PTGS2 | target |
| MOL007101 | PTGS2 | target |
| MOL007105 | PTGS2 | target |
| MOL007108 | PTGS2 | target |
| MOL007111 | PTGS2 | target |
| MOL007119 | PTGS2 | target |
| MOL007120 | PTGS2 | target |
| MOL007122 | PTGS2 | target |
| MOL007124 | PTGS2 | target |
| MOL007125 | PTGS2 | target |
| MOL007127 | PTGS2 | target |
| MOL007130 | PTGS2 | target |
| MOL007132 | PTGS2 | target |
| MOL007141 | PTGS2 | target |
| MOL007143 | PTGS2 | target |
| MOL007145 | PTGS2 | target |
| MOL007150 | PTGS2 | target |
| MOL007151 | PTGS2 | target |
| MOL007152 | PTGS2 | target |
| MOL007154 | PTGS2 | target |
| MOL007155 | PTGS2 | target |
| MOL007156 | PTGS2 | target |
| MOL007154 | FOS | target |
| MOL000006 | AKT1 | target |
| MOL000006 | ERBB2 | target |
| MOL000006 | RB1 | target |
| MOL007088 | STAT3 | target |
| MOL001601 | SCN5A | target |
| MOL002222 | SCN5A | target |
| MOL002651 | SCN5A | target |
| MOL007036 | SCN5A | target |
| MOL007041 | SCN5A | target |
| MOL007045 | SCN5A | target |
| MOL007049 | SCN5A | target |
| MOL007061 | SCN5A | target |
| MOL007069 | SCN5A | target |
| MOL007082 | SCN5A | target |
| MOL007088 | SCN5A | target |
| MOL007093 | SCN5A | target |
| MOL007094 | SCN5A | target |
| MOL007098 | SCN5A | target |
| MOL007100 | SCN5A | target |
| MOL007101 | SCN5A | target |
| MOL007105 | SCN5A | target |
| MOL007107 | SCN5A | target |
| MOL007108 | SCN5A | target |
| MOL007111 | SCN5A | target |
| MOL007119 | SCN5A | target |
| MOL007122 | SCN5A | target |
| MOL007124 | SCN5A | target |
| MOL007125 | SCN5A | target |
| MOL007127 | SCN5A | target |
| MOL007145 | SCN5A | target |
| MOL007154 | SCN5A | target |
| MOL007155 | SCN5A | target |
| MOL007156 | SCN5A | target |
| MOL001601 | SLC6A3 | target |
| MOL007041 | SLC6A3 | target |
| MOL007049 | SLC6A3 | target |
| MOL007100 | SLC6A3 | target |
| MOL007122 | SLC6A3 | target |
| MOL007124 | SLC6A3 | target |
| MOL007145 | SLC6A3 | target |
| MOL000006 | JUN | target |
| MOL007154 | JUN | target |
| MOL000006 | RELA | target |
| MOL007088 | RELA | target |
| MOL007154 | RELA | target |
| MOL000006 | PRSS1 | target |
| MOL007045 | PRSS1 | target |
| MOL007059 | PRSS1 | target |
| MOL007061 | PRSS1 | target |
| MOL007068 | PRSS1 | target |
| MOL007070 | PRSS1 | target |
| MOL007071 | PRSS1 | target |
| MOL007079 | PRSS1 | target |
| MOL007093 | PRSS1 | target |
| MOL007100 | PRSS1 | target |
| MOL007108 | PRSS1 | target |
| MOL007130 | PRSS1 | target |
| MOL007132 | PRSS1 | target |
| MOL007142 | PRSS1 | target |
| MOL007150 | PRSS1 | target |
| MOL007155 | PRSS1 | target |
| MOL000006 | TNF | target |
| MOL007088 | TNF | target |
| MOL000006 | VEGFA | target |
| MOL000006 | XDH | target |
